# Supplementary material for: Carbene organic catalytic planar enantioselective macrolactonization
Source: Nat Commun. 2024 Feb 1;15:958. doi: 10.1038/s41467-024-45218-x (PMC10834540; doi:10.1038/s41467-024-45218-x)
Supplement: Supplementary file 1 — Supplimentary Information [file 41467_2024_45218_MOESM1_ESM.pdf]

# Supplementary Information

## Carbene Organic Catalytic Planar Enantioselective Macrolactonization

Xiaokang Lv,[1] Fen Su,[1] Hongyan Long,[1] Fengfei Lu,[1] Yukun Zeng,[1] Minghong Liao,[1] Fengrui Che,[1] Xingxing Wu[1],* and Yonggui Robin Chi[1,2],*

Affiliations:

[1]National Key Laboratory of Green Pesticide, Key Laboratory of Green Pesticide and Agricultural Bioengineering, Ministry of Education, Guizhou University, Huaxi District, Guiyang 550025, China.

[2]School of chemistry, chemical engineering, and biotechnology, Nanyang Technological University, Singapore 637371, Singapore

* Correspondence:

wuxx@gzu.edu.cn

robinchi@ntu.edu.sg.

# Table of Contents


**I. Supplementary Methods**……………………………………………Page S1

**1. General Information**…………………………………………………Page S1

**2. Experimental Section**…………………………………………...…...……Page S2

(1) Typical procedure for preparation of substrates…………………….……...Page S2

(2) Supplementary results of condition optimization.….......…………….........Page S6

(3) General procedure for the catalytic enantioselective macrolactonizations…Page S8

(4) Synthetic transformations of chiral products **2a** and **2c**.…………………...Page S9

(5) Plausible mechanism………...……………………………………….Page S11

(6) Limitations of the developed method……………………………………..Page S13

(7) Intermolecular macrolactonization between **S8** and **S9**…...………………....Page S27

(8) X-ray crystallography of chiral product ($R_p$)-**2p**………...………………....Page S30

**II. Supplementary Notes**……………………...………………………Page S31

**Characterization of substrates and planar chiral products**.....................Page S31

(1) Characterization of the prepared substrates…………………………..Page S31

(2) Characterization of the planar chiral products …………………………..Page S48

**III. Supplementary Figures**……………………...……...……………Page S63

$^{1}$H, $^{13}$C NMR spectra and HPLC traces..………..……………………..Page S63

**IV. Supplementary References**……….......…..........……………..Page S147

# I. Supplementary Methods

## 1. General information

Commercially available materials purchased from Energy Chemical and J&K were used as received. Unless otherwise specified, all reactions were carried under an atmosphere of Nitrogen in 100.0 mL Schlenk tube. NMR spectra were measured either on a JEOL-ECX-500 (500 MHz) or on a Bruker ASCEND 400 (400 MHz) Spectrometer. The chemical shift values were corrected to 7.26 ppm ($^1$H NMR), 1.60 ppm ($^1$H NMR) and 77.16 ppm ($^{13}$C NMR) for $CDCl_3$; $^1$H NMR splitting patterns are designated as singlet (s), double (d), triplet (t), quartet (q), doublet of doublets (dd), multiplets (m), and etc. All first-order splitting patterns were assigned on the base of the appearance of the multiplet. Splitting patterns that could not be easily interpreted are designated as multiplet (m) or broad (br). High resolution mass spectrometer analysis (HRMS) was performed on Thermo Fisher Q Exactive mass spectrometer. HPLC analyses were measured on Waters systems with Empower 3 system controller, Alliance column heater, and 2998 Diode Array Waters 2489 UV/Vis detector. Chiralcel brand chiral columns from Daicel Chemical Industries were used with models IA, IB, ID, IE, IG, IF, AD-H, AS-H, OD-H in 4.6 × 250 mm size. The racemic products used to determine the er values were synthesized using racemic catalyst. Optical rotations were measured on a Insmark IP-digi Polarimeter in a 1 dm cuvette. The concentration (*c*) is given in g/100 mL. Melting Point (MP): Melting points were measured on a Beijing Tech Instrument X-4 digital display micro melting point apparatus and are uncorrected. Analytical thin-layer chromatography (TLC) was carried out on pre-coated silica gel plate (0.2 mm thickness). Visualization was performed using a UV lamp.

## 2. Experimental Section

### (1) Typical procedure for preparation of substrates

**General procedure for the synthesis of 4a-4c**[1-4]

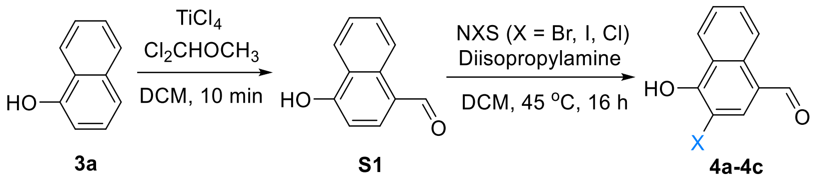


**Step 1**: Formylation reaction to prepare building block **S1**[1]

To a stirred solution of 1-naphthol (**3a**, 2.9 g, 20.0 mmol, 1.0 equiv.) in $CH_2Cl_2$ (25.0 mL) at 0 °C was dropwise added titanium tetrachloride (4.4 mL, 40.0 mmol, 2.0 equiv.). Dichloro(methoxy)methane (2.8 mL, 31.0 mmol, 1.5 equiv.) was then added to the resulting dark solution over 5 minutes at 0 °C. The solution was keeping stirring for additional 10 minutes, then ice water (5.6 mL) was added. The heterogeneous mixture was stirred vigorously at 0 °C until all titanium salts dissolved. The organic layer was separated and the aqueous layer was extracted with $CH_2Cl_2$ (3 x 50.0 mL). The combined organic extracts were washed with brine (50.0 mL), dried over $Na_2SO_4$, and concentrated in vacuo. The crude product was purified by column chromatography on silica gel (Petroleum ether / Ethyl acetate = 95:5 to 50:50) to afford 4-hydroxy-1-naphthaldehyde (**S1**, 2.8 g, 80% yield) as a white solid. Product **S1** matched literature characterization data.[1]

**Step 2**: Preparation of building block **4a-4c**[2-4]

Precursor **S1** (2.5 g, 17.36 mmol, 1.0 equiv.) and diisopropylamine (0.24 mL, 0.24 g, 3.47 mmol, 0.1 equiv.) were dissolved in $CH_2Cl_2$ (100.0 mL) and treated with NXS (X = Br, Cl, I, 33.28 mmol, 1.0 equiv.) for 16 h at reflux. After cooling to room temperature, the resulting mixture was treated with 2 M sulfuric acid (100.0 mL). The crude product was purified by flash chromatography (Cyclohexane / EtOAc 40:1) to afford **4a-c** as yellow solids. Product **4a-c** matched literature characterization data.[2-4]

## General procedure for the synthesis of 5a-5f[5-10]

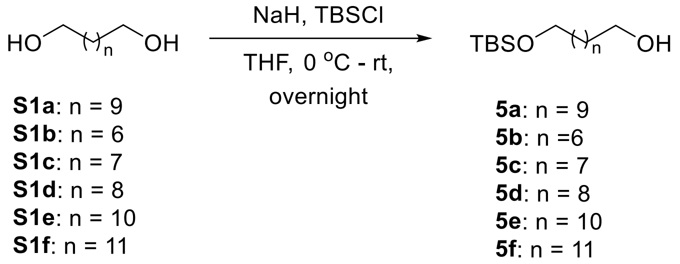


A 60% dispersion of NaH in mineral oil (0.9 g, 22.6 mmol, 1.1 equiv.) was suspended in distilled THF (40.0 mL), followed by the addition of diol **S1a-S1f** (20.5 mmol, 1.0 equiv.) in THF (10.0 mL). The mixture was stirred at room temperature for 2 h, at which time a large amount of white precipitates were formed. After the addition of *tert*-butyldimethylsilyl chloride (3.1 g, 20.5 mmol, 1.0 equiv.) in THF (5.0 mL), the mixture was stirred vigorously at room temperature overnight. The reaction was quenched with small ice pieces and extracted with ether. The organic layers were washed with brine, dried over $Na_2SO_4$, filtered, and concentrated in vacuum. Purification by flash chromatography on silica gel (Petroleum ether / EtOAc = 10:1) afforded the title compound **5a-f** as colorless oil.

Compounds **5a**, **5b**, **5c, 5d**, **5e** and **5f** were obtained in 70%, 61%, 66%, 81%, 65% and 51% yield, respectively, following the general procedure. All products **5a-f** matched literature characterization data.[5-10]

## General procedure A for the synthesis of substrate 1a-1c and 1p-1t[11]

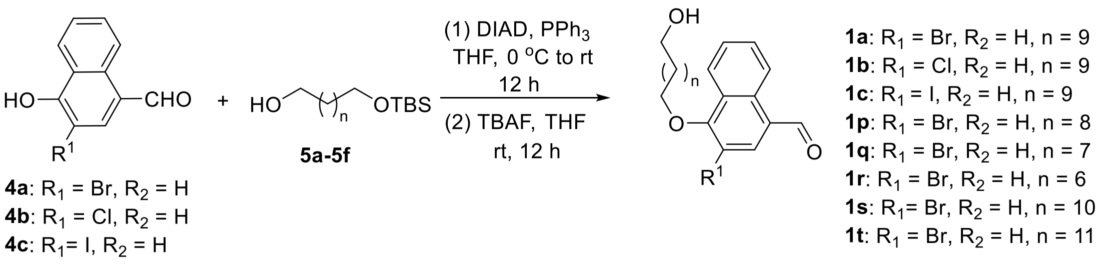


To a THF solution (5.0 mL) of **4a-c** (1.0 mmol, 1.0 equiv.), **5a-5f** (1.5 mmol, 1.5 equiv.), and triphenylphosphine (390.0 mg, 1.5 mmol, 1.5 equiv.), diisopropylazodicarboxylate (DIAD, 300.0 mg, 1.5 mmol, 1.5 equiv.) was added at 0 °C. After the resulting solution was stirred at 25 °C for 12 h, the reaction mixture was

evaporated to dryness under reduced pressure and extracted 3 times with ethyl acetate. The organic layers were washed with brine, dried over $Na_2SO_4$, filtered, and concentrated in *vacuo*. The crude product was dissolved in 10.0 mL THF, then 1.5 mL TBAF (1.0 M in THF) was added. The reaction mixture was stirred at 25 °C for 12 h. After consumption of the material, the solvent was removed in *vacuo*. Purification of the obtained residue by flash chromatography on silica gel (Petroleum ether / EtOAc = 5:1) would afford substrate **1**.

**General procedure B for the synthesis of substrate 1d-1l**[12-15]

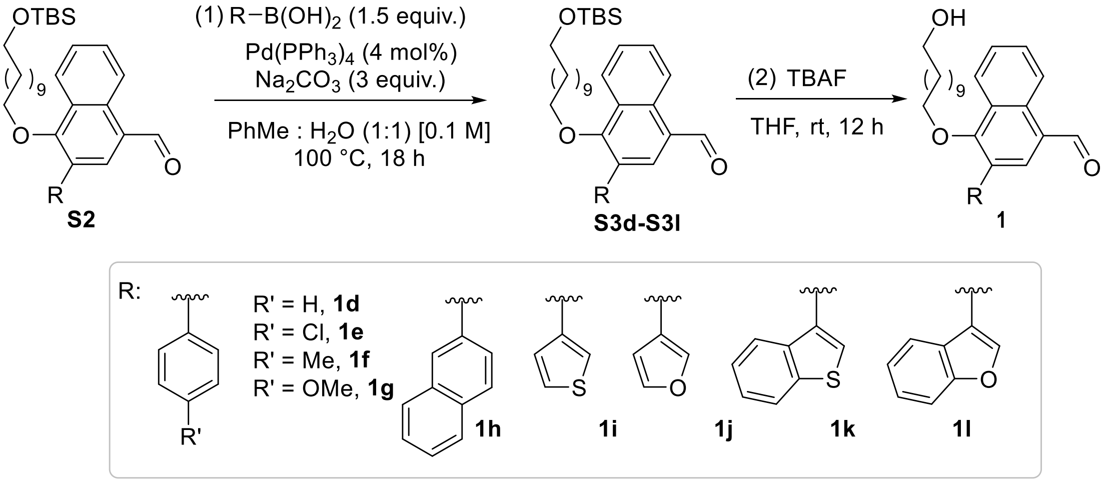


**Step 1**: Suzuki coupling reaction to prepare building block **S3**

Building block **S2** (534.0 mg, 1.0 mmol, 1.0 equiv.), phenylboronic acid (122.0 mg, 1.5 mmol, 1.5 equiv., prepared according to general procedure A), sodium carbonate (318.0 mg, 3.0 mmol, 3.0 equiv.) and $Pd(PPh_3)_4$ (46.2 mg, 0.04 mmol, 4 mol%) were dissolved in 10.0 mL of a toluene:water (1:1) mixture in an open sealed tube equipped with a stir bar under an atmosphere of nitrogen. The vessel was sealed with a screw cap and the solution was heated at 100 °C for 18 h. The reaction was cooled to room temperature and extracted 3 times with ethyl acetate. The organic layers were combined, dried over sodium sulfate and evaporated under reduced pressure. The crude product was purified by flash chromatography on silica gel (Petroleum ether / EtOAc = 20:1) to afford substrate **S3d-S3l**.

**Step 2**: deprotection of TBS group to prepare substrate **1d-1l**

Precursor **S3** (0.5 mmol, 1.0 equiv.) was dissolved in 5.0 mL THF, then 0.5 mL of TBAF (1.0 M in THF) was added. The reaction mixture was stirred at 25 °C for 12 h before the solvent was removed in *vacuo*. Purification by flash chromatography on silica gel (Petroleum ether / EtOAc = 5:1) would afford **1d-1l**.

## Preparation of additive HBD 3

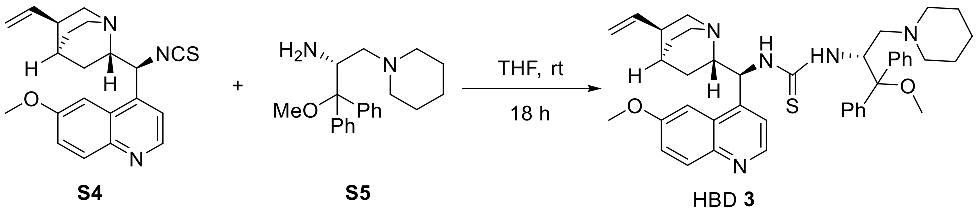


HBD **3** was prepared following literature procedure.[16,17] To the solution of **S4** (584.0 mg, 1.6 mmol, 1.0 equiv.) in 5.0 mL of THF, **S5** (570.0 mg, 1.765 mmol, 1.1 equiv.) was added and the reaction mixture was stirred at rt for 18 h. Reaction mixture was concentrated *in vacuo* to give a sticky liquid. Purification by column chromatography over silica gel using $CH_2Cl_2$ / $CH_3OH$ / $Et_3N$ (100: 5: 1) afforded HBD **3** as a white solid (562.0 mg, 51% yield).

## (2) Supplementary results of condition optimization

**Supplementary Table 1**. The effects of NHCs, bases, additive, solvents[a]

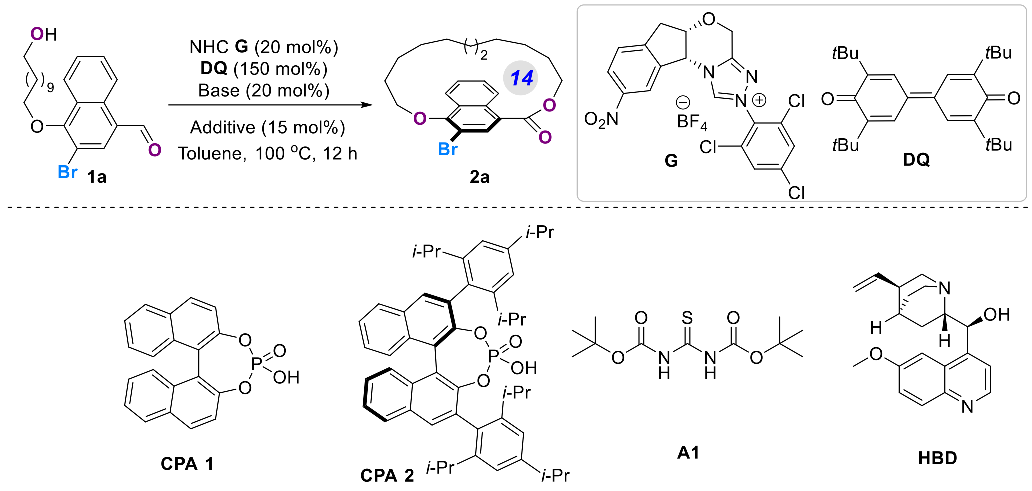


| Entry | NHC | Base | Solvent | Additives | Temp. | Yield (%)[b] | E.r. (%)[c] |
|---|---|---|---|---|---|---|---|
| 1 | **G** | $Na_2CO_3$ | Toluene | -- | 100 °C | 44 | 88:12 |
| 2 | **G** | KOAc | Toluene | -- | 100 °C | 45 | 92:8 |
| 3 | **G** | $K_3PO_4$ | Toluene | -- | 100 °C | 44 | 89:11 |
| 4 | **G** | DBU | Toluene | -- | 100 °C | 32 | 89:11 |
| 5 | **G** | DABCO | $CHCl_3$ | -- | 60 °C | 33 | 90:10 |
| 6 | **G** | DABCO | THF | -- | 60 °C | 43 | 89:11 |
| 7 | **G** | DABCO | PhCl | -- | 100 °C | 34 | 93:7 |
| 8 | **G** | DABCO | *n*-Heptane | -- | 100 °C | 13 | 91:9 |
| 9 | **G** | DABCO | Toluene | $Cu(acac)_2$ | 100 °C | 44 | 93:7 |
| 10 | **G** | DABCO | Toluene | $Sc(OTf)_3$ | 100 °C | 5 | 95:5 |
| 11 | **G** | DABCO | Toluene | $Cu(OTf)_2$ | 100 °C | 17 | 95:5 |
| 12 | **G** | DABCO | Toluene | $Mg(OTf)_2$ | 100 °C | 9 | 94:6 |
| 13 | **G** | DABCO | Toluene | PhCOOH | 100 °C | 40 | 93:7 |
| 14 | **G** | DABCO | Toluene | TsOH | 100 °C | -- | -- |
| 15 | **G** | DABCO | Toluene | $PhB(OH)_2$ | 100 °C | 47 | 92:8 |
| 16 | **G** | DABCO | Toluene | **CPA 1** | 100 °C | 37 | 94:6 |
| 17 | **G** | DABCO | Toluene | **CPA 2** | 100 °C | 6 | 91:9 |
| 18 | **G** | DABCO | Toluene | **A1** | 100 °C | 41 | 92:8 |
| 19 | **G** | DABCO | Toluene | HBD **1** | 100 °C | 45 | 92:8 |
| 20 | **G** | DABCO | $CHCl_3$ | HBD **3** | 60 °C | 35 | 92:8 |
| 21 | **G** | DABCO | THF | HBD **3** | 60 °C | 22 | 90:10 |
| 22 | **G** | DABCO | PhCl | HBD **3** | 100 °C | 59 | 91:9 |
| 23 | **G** | DABCO | *n*-Heptane | HBD **3** | 100 °C | 17 | 93:7 |
| 24[d] | **G** | DABCO | Toluene: *n*-Heptane 1.2:1 | HBD **3** | 100 °C | 24 | 95:5 |

| | | | | | | | |
|---|---|---|---|---|---|---|---|
| 25[e] | **G** | DABCO | Toluene: *n*-Heptane 1.2:1 | HBD **3** | 100 °C | 40 | 94:6 |

[a] General conditions (unless otherwise specified): **1a** (6.3 mg, 0.015 mmol, 1.0 equiv.), NHC **G** (20 mol%), **DQ** (9.2 mg, 150 mol%), and base (20 mol%) in toluene (1 mM) under $N_2$ atmosphere at 100 °C for 12 h. [b] Yields of **4a** were determined via $^1$H NMR analysis with 1,3,5-trimethoxy-benzene as an internal standard. [c] The e.r. values of **2a** were determined by HPLC using a chiral stationary phase. [d] The reaction was performed at a concentration of [10.0 mM] at 100 °C for 12 h. [e] The reaction was performed at a concentration of [2.5 mM] at 100 °C for 12 h. DIEA = *N, N*-Diisopropylethylamine, DABCO = Triethylenediamine, DBU = 1,8-Diazabicyclo [5,4,0] undec-7-ene, THF = Tetrahydrofuran.

**Supplementary Table 2.** Examination of the effects of reaction temperatures[a]

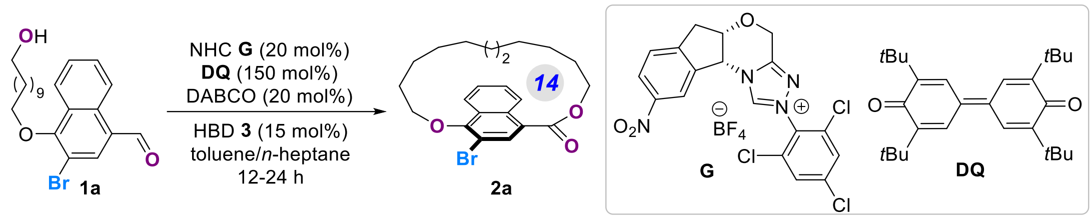


| Entry | Temp. | Yield (%)[b] | E.r. (%) |
|---|---|---|---|
| 1 | 25 | 37 | 95:5 |
| 2 | 50 | 33 | 95:5 |
| 3 | 70 | 42 | 96:4 |
| 4 | 100 | 82 | 96:4 |
| 5 | 110 | 76 | 96:4 |

[a] General conditions (unless otherwise specified): **1a** (6.3 mg, 0.015 mmol, 1.0 equiv.), NHC **G** (20 mol%), **DQ** (9.2 mg, 150 mol%), and DABCO (20 mol%) in toluene/*n*-heptane (11:9 v/v, 1 mM) under $N_2$ atmosphere at indicated temperature for 12-24 h (entries 1-3, 24 h; entries 4-5, 12 h). [b] Yields of **2a** were determined via $^1$H NMR analysis with 1,3,5-trimethoxybenzene as an internal standard.

## (3) General procedure for the catalytic enantioselective macrolactonizations

### General procedure C:

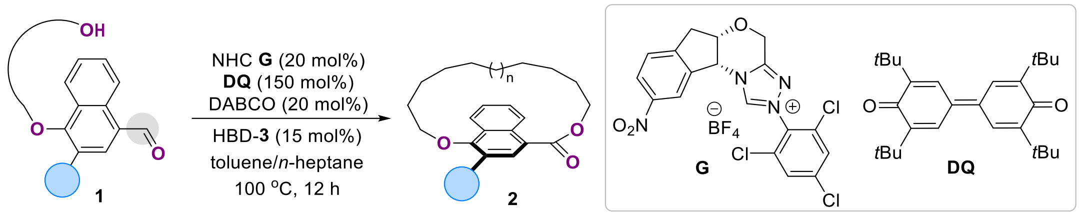


To a 100.0 mL Schlenk flask equipped with a magnetic stir bar was added chiral NHC pre-catalyst **G** (5.2 mg, 0.01 mmol, 20 mol%), **DQ** (0.075 mmol, 150 mol%, 30.6 mg), DABCO (1.12 mg, 0.01 mmol, 20 mol%), HBD **3** (5.17 mg, 0.0075 mmol, 15 mol%,) and aldehyde substrate **1** (0.05 mmol, 1.0 equiv.). After that, solvent toluene / *n*-heptane (11:9 *v*/*v*, 1 mM) was added and the reaction mixture was allowed to stir for 12 hours at 100 °C. Then the mixture was concentrated under reduced pressure. The resulting crude residue was purified by column chromatography on silica gel to afford the desired planar chiral product **2**.

## (4) Synthetic transformations of chiral products 2a and 2c

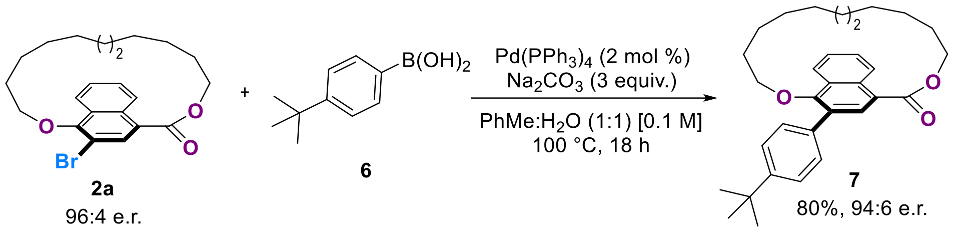


**Supplementary Figure 1**. Preparation of **7** from macrolactone **2a**

Compound ($R_p$)-**2a** (21.0 mg, 0.05 mmol, 1.0 equiv.), *tert*-butylbenzeneboronic acid **6** (13.4 mg, 0.075 mmol, 1.5 equiv.), sodium carbonate (15.9 mg, 0.15 mmol, 3 equiv.) and $Pd(PPh_3)_4$ (2.3 mg, 0.002 mmol, 4 mol%) were dissolved in 10.0 mL of a toluene:water (1:1) mixture in an open sealed tube equipped with a stir bar under an atmosphere of nitrogen. The vessel was sealed with a screw cap and the solution was heated at 100 °C for 18 h. The reaction was cooled to room temperature and extracted 3 times with ethyl acetate. The organic layers were combined, dried over sodium sulfate and evaporated under reduced pressure. The crude product was purified by flash chromatography on silica gel (Petroleum ether / EtOAc = 50:1) to afford **7** (light yellow oil, 18.9 mg, 80% yield, 94: 6 er)

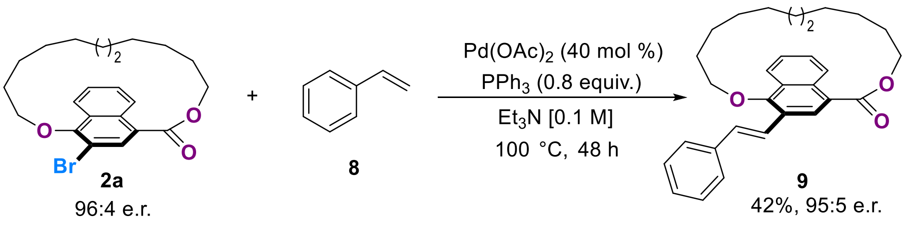


**Supplementary Figure 2**. Preparation of **9** from macrolactone **2a**

($R_p$)-**2a** (21.0 mg, 0.05 mol, 1.0 equiv.), $Pd(OAc)_2$ (4.5 mg, 0.02 mmol, 40 mol%), $PPh_3$ (10.5 mg, 0.04 mmol, 0.8 equiv.) and styrene **8** (31.0 uL, 0.25 mmol, 5.0 equiv.) were dissolved in 0.6 mL of dry triethylamine in an open dried sealed tube equipped with a stir bar under an atmosphere of nitrogen. The solution was bubbled with nitrogen, sealed with a screw cap and heated at 100 °C for 48 h. The reaction was cooled to room temperature and extracted 3 times with ethyl acetate. The organic layers were combined, dried over sodium sulfate and evaporated under reduced pressure. The crude product

was purified by flash chromatography on silica gel (Petroleum ether / EtOAc = 40:1) to give title compound **9** (light yellow oil, 9.3 mg, 42% yield, 95: 5 er).

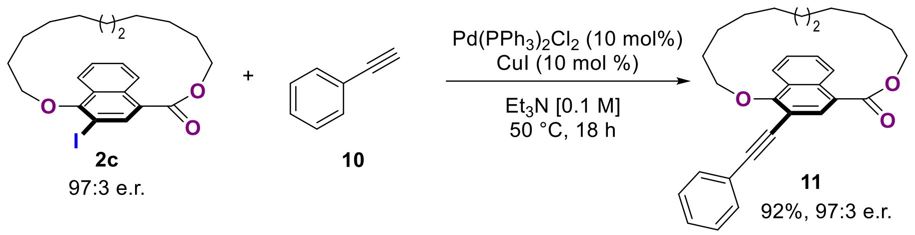


**Supplementary Figure 3**. Preparation of **11** from macrolactone **2c**

($R_p$)-**2c** (23.3 mg, 0.05 mmol, 1.0 equiv.), $Pd(PPh_3)_2Cl_2$ (3.51 mg, 0.005 mmol, 10 mol%), and copper iodide (0.97 mg, 0.005 mmol, 10 mol%) were dissolved in 1.0 mL of a triethylamine in an open dried sealed tube equipped with a stir bar under an atmosphere of nitrogen. The solution was bubbled with nitrogen and phenylacetylene (16.6 uL, 0.15 mmol, 3.0 equiv.) was finally added. The vessel was sealed with a screw cap and the solution was heated at 50 °C for 18 h. The reaction was cooled to room temperature and extracted 3 times with ethyl acetate. The organic layers were combined, dried over sodium sulfate and evaporated under reduced pressure. The crude product was purified by flash chromatography on silica gel (Petroleum ether / EtOAc = 40:1) to provide product **11** (light yellow oil, 20.2 mg, 92% yield, 97: 3 er).

### (5) Plausible mechanism

A plausible mechanism of the carbene-catalyzed planar enantioselective macrolactonization was proposed (Supplementary Figure 4). The initially formed carbene NHC **G** from the chiral triazolium salt underwent nucleophilic attack on the formyl group of **1**, followed by a rapid proton transfer that resulted in the formation of the Breslow intermediate **A**. Facile oxidation by the **DQ** oxidant generated the key acyl azolium intermediate **B/B'**. Considering the transition state (**TS**) of azolium intermediate, **B'** was unfavorable due to the significant steric repulsion between the carbene backbone and naphthalene ring. A subsequent nucleophilic addition of the hydroxyl group in transition state **B** would lead to the planar chiral macrolactone **2** in ***R*p** configuration and regenerate the NHC **G** catalyst for the next catalytic cycle. Notably, the intermolecular lactonization was the competing reaction pathway to give the dimer side product.

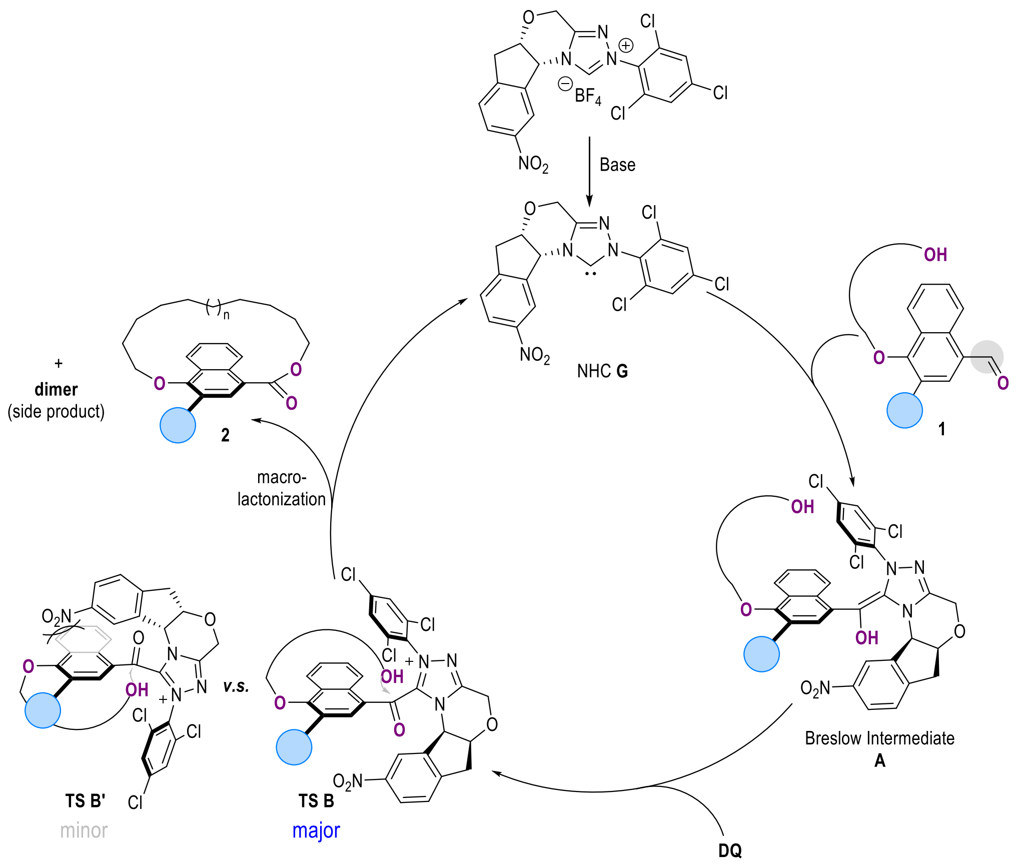


**Supplementary Figure 4**. Plausible reaction mechanism

**1,16-Dibromo-2,14,17,29-tetraoxa-1,16(1,4)-dinaphthalenacyclotriacontaphane-15,30-dione**

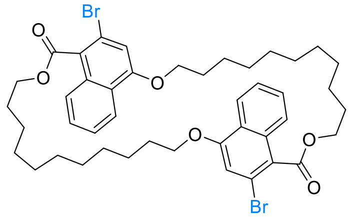

**$^{1}$H NMR** (400 MHz, $CDCl_3$) δ 8.94 (d, *J* = 8.6 Hz, 1H), 8.32 (s, 1H), 8.15 (d, *J* = 9.9 Hz, 1H), 7.60 (ddd, *J* = 8.6, 6.8, 1.5 Hz, 1H), 7.53 (ddd, *J* = 8.2, 6.8, 1.3 Hz, 1H), 4.41 (t, *J* = 5.8 Hz, 2H), 4.10 (t, *J* = 6.8 Hz, 2H), 1.93 (p, *J* = 6.8 Hz, 2H), 1.81 (dt, *J* = 11.8, 6.2 Hz, 2H), 1.59 – 1.50 (m, 6H), 1.41 – 1.33 (m, 8H).

**$^{13}$C NMR** (101 MHz, $CDCl_3$) δ 166.1, 156.5, 134.9, 132.2, 129.8, 128.2, 126.9, 126.3, 124.3, 122.6, 111.4, 74.8, 65.5, 30.1, 29.3, 29.1, 29.0, 28.9, 28.8, 28.6, 26.3, 25.6.

**HRMS** (ESI, m/z) Calcd. for $C_{44}H_{55}Br_2O_6^+$, [M+H] $^+$: 837.2365, found: 837.1969.

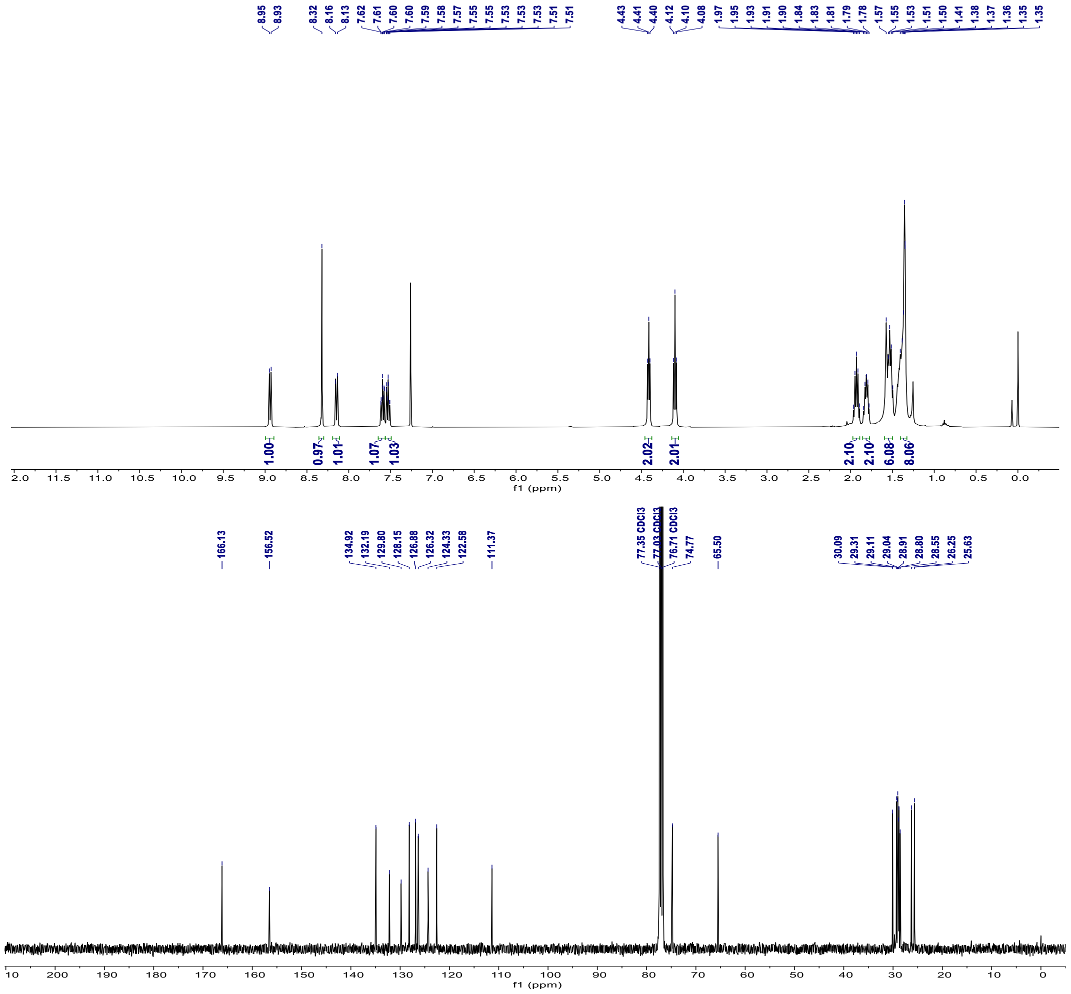

**Supplementary Figure 5.** $^{1}$H and $^{13}$C NMR spectra of 1,16-Dibromo-2,14,17,29-tetraoxa-1,16(1,4)-dinaphthalenacyclotriacontaphane-15,30-dione

## (6) Limitations of the developed method

We attempted to obtain the macrocyclization product **S4b** with thiol-based precursor **S4a**. Initial conditions screening with the optimal conditions failed to give any desired product **S4b** (Supplementary Table 3, entry 1). We then performed the reaction at elevated temperatures at 110-150 °C (Supplementary Table 3, entries 2-4). However, we were still not able to observe any product **S4b**, with a large amount of starting material remained in the reaction. Furthermore, we tested additional reaction conditions with various NHC catalysts, solvents and temperatures (Supplementary Table 4). While the product **S4b** was still not obtained, a side product **S4c** was isolated in several entries.

**3-Bromo-4-(3-mercaptopropoxy)-1-naphthaldehyde--methane (S4a)**

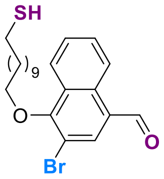


$^{1}$H NMR (400 MHz, $CDCl_3$) δ 10.24 (s, 1H), 9.21 (d, $J$ = 10.4 Hz, 1H), 8.19 (d, $J$ = 8.4 Hz, 1H), 8.08 (s, 1H), 7.74 – 7.67 (m, 1H), 7.63 (d, $J$ = 8.4 Hz, 1H), 4.16 (t, $J$ = 6.6 Hz, 2H), 2.75 – 2.62 (m, 2H), 2.03 – 1.93 (m, 2H), 1.73 – 1.54 (m, 5H), 1.42 – 1.30 (m, 11H).

$^{13}$C NMR (101 MHz, $CDCl_3$) δ 190.3, 157.3, 140.2, 128.8, 128.5, 127.7, 126.7, 124.2, 110.6, 74.1, 29.2, 28.5, 28.5, 28.5, 28.4, 28.2, 28.2, 27.5, 24.9.

**HRMS** (ESI, m/z) Calcd. for $C_{22}H_{29}ClO_3Na^+$, [M+Na] $^+$: 399.1697, found: 399.1691.

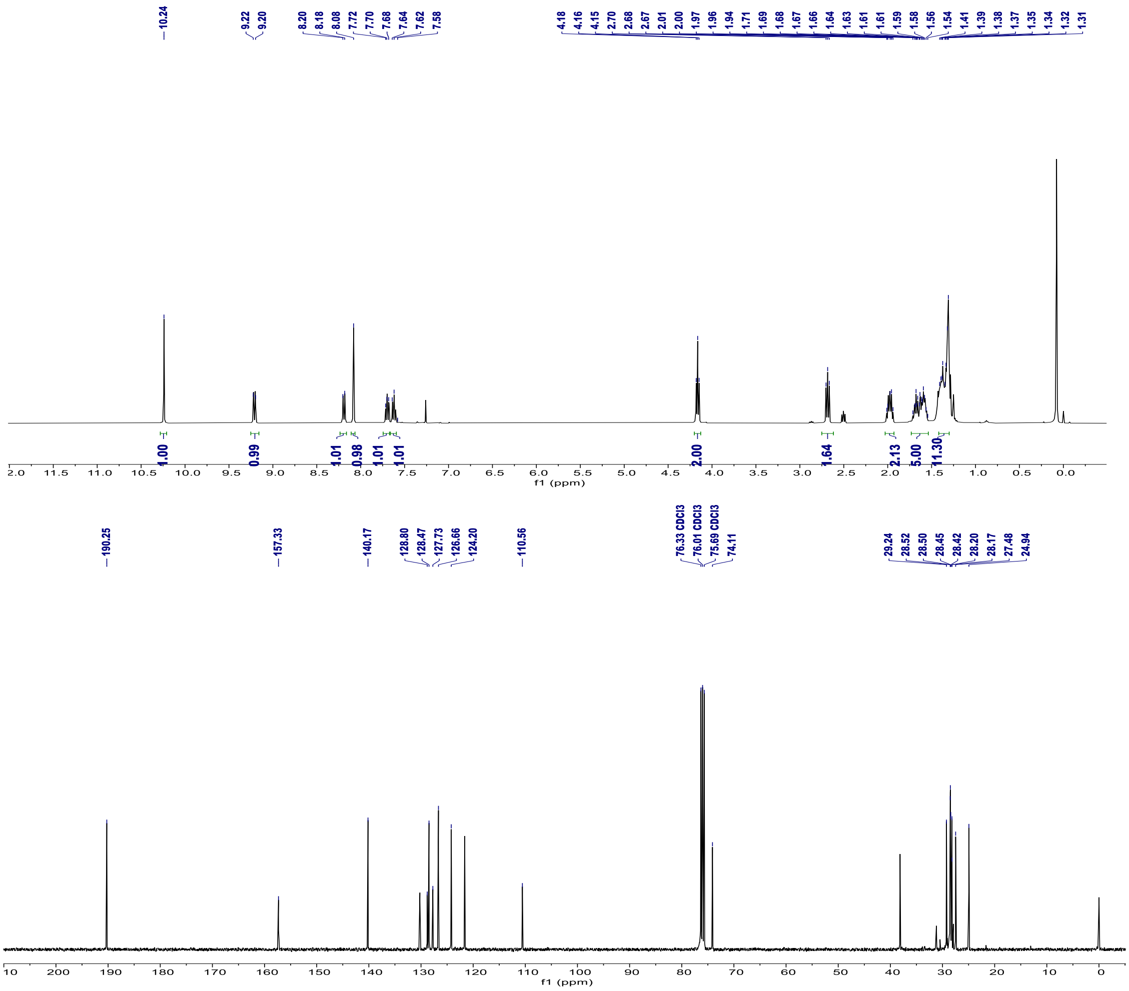


**Supplementary Figure 6.** $^{1}H$ and $^{13}C$ NMR spectra of **S4a**

**Supplementary Table 3.** Macrolactonization of thiol-based substrate **S4a** with NHC **G**

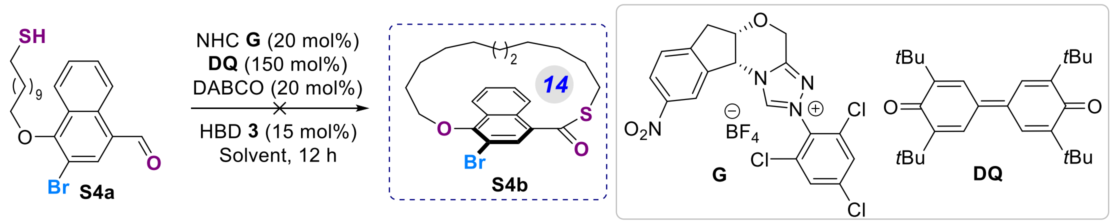


| Entry | Solvent | Temp. (°C) | Results |
|---|---|---|---|
| 1 | Toluene/*n*-Heptane | 100 | A large amount of **S4a** remained; **S4b** not observed |
| 2 | Toluene/*n*-Heptane | 110 | A large amount of **S4a** remained; **S4b** not observed |
| 3 | PhCl | 130 | A large amount of **S4a** remained; **S4b** not observed |
| 4 | Mesitylene | 150 | A large amount of **S4a** remained; **S4b** not observed |

[a] General conditions (unless otherwise specified): **S4a** (6.5 mg, 0.015 mmol, 1.0 equiv.), NHC **G** (20 mol%), **DQ** (9.2 mg, 150 mol%), and DABCO (20 mol%) in solvents (1 mM; entries 1-2, toluene/*n*-heptane = 11:9 v/v) under $N_2$ atmosphere at indicated temperature for 12 h.

**Supplementary Table 4.** Additional screening on the macrolactonization with substrate **S4a**

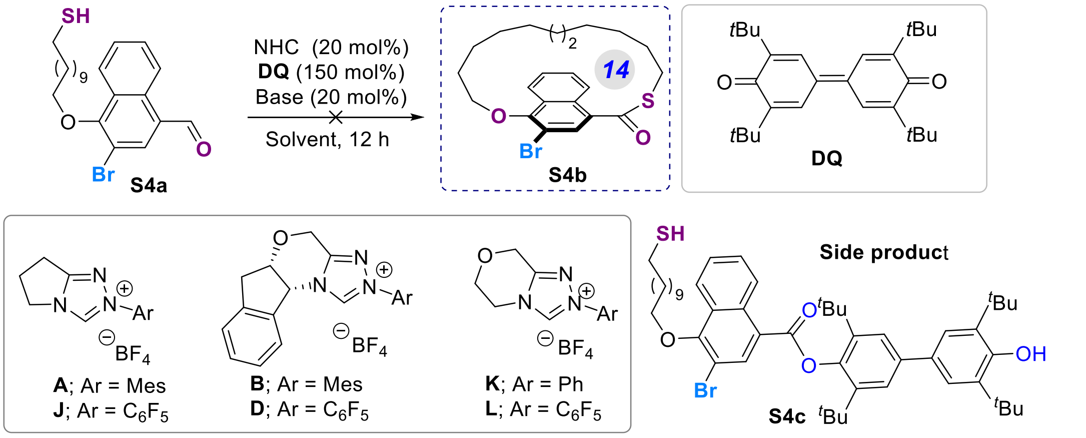


| Entry | NHC | Base | Solvent | Temp. | Result |
|---|---|---|---|---|---|
| 1 | **A**, **B**, **D**, **K**, **J** | $K_2CO_3$ | THF | 70 | A large amount of **S4a** remained |
| 2 | **L** | $K_2CO_3$ | THF | 70 | **S4c** was obtained |
| 3 | **B, D, J, K** | $K_2CO_3$ | Toluene | 100 | A large amount of **S4a** remained |
| 4 | **A, L** | $K_2CO_3$ | Toluene | 100 | **S4c** was obtained |
| 5 | **A, B, D, K** | $Cs_2CO_3$ | THF | 70 | A large amount of **S4a** remained |
| 6 | **J, L** | $Cs_2CO_3$ | THF | 70 | **S4c** was obtained |
| 7 | **A, B, D, J, L** | $Cs_2CO_3$ | Toluene | 100 | A large amount of **S4a** remained |
| 8 | **K** | $Cs_2CO_3$ | Toluene | 100 | **S4c** was obtained |

[a] General conditions (unless otherwise specified): **S4a** (6.5 mg, 0.015 mmol, 1.0 equiv.), NHC Cat. (20 mol%), **DQ** (9.2 mg, 150 mol%), and base (20 mol%) in solvents (1 mM) under $N_2$ atmosphere at indicated temperature for 12 h.

Macrocyclization with *N*-based precursor **S5a** was further studied. The starting material **S5a** was successfully prepared. However, similar to the reaction with precursor **S4a**, we could not obtain the corresponding product **S5b** with our catalytic conditions or at elevated temperatures (Supplementary Table 5, entries 1-4). Additional screenings with NHCs, solvents and temperatures also failed to give the desired macrocyclization product **S5b** (Supplementary Table 6). Coupling product **S5c** with the reduced hydroquinone was detected in several reactions.

***N*-(3-((2-bromo-4-formylnaphthalen-1-yl)oxy)propyl)-4-methylbenzenesulfonamidemethane (S5a)**

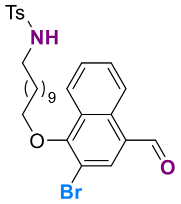


$^{1}$H NMR (400 MHz, $CDCl_3$) δ 10.25 (s, 1H), 9.22 (d, *J* = 10.0 Hz, 1H), 8.20 (d, *J* = 8.4 Hz, 1H), 8.10 (s, 1H), 7.75 (d, *J* = 8.4 Hz, 2H), 7.72 – 7.69 (m, 1H), 7.66 – 7.62 (m, 1H), 7.30 (d, *J* = 8.0 Hz, 2H), 4.43 (t, *J* = 5.6 Hz, 1H), 4.17 (t, *J* = 6.6 Hz, 2H), 2.93 (q, *J* = 6.8 Hz, 2H), 2.42 (s, 3H), 1.99 (q, *J* = 6.6 Hz, 2H), 1.60 (q, *J* = 6.1, 5.6 Hz, 3H), 1.48 – 1.40 (m, 4H), 1.25 – 1.18 (m, 7H).

$^{13}$C NMR (101 MHz, $CDCl_3$) δ 190.3, 157.4, 142.3, 140.2, 136.0, 130.2, 128.8, 128.7, 128.5, 127.8, 126.7, 126.1, 124.2, 121.6, 110.6, 74.1, 42.2, 29.2, 28.7, 28.6, 28.5, 28.4, 28.4, 28.0, 25.5, 24.9, 20.5.

**HRMS** (ESI, m/z) Calcd. for $C_{29}H_{36}BrNO_4SNa^+$, [M+Na] $^+$: 596.1441, found: 596.1434.

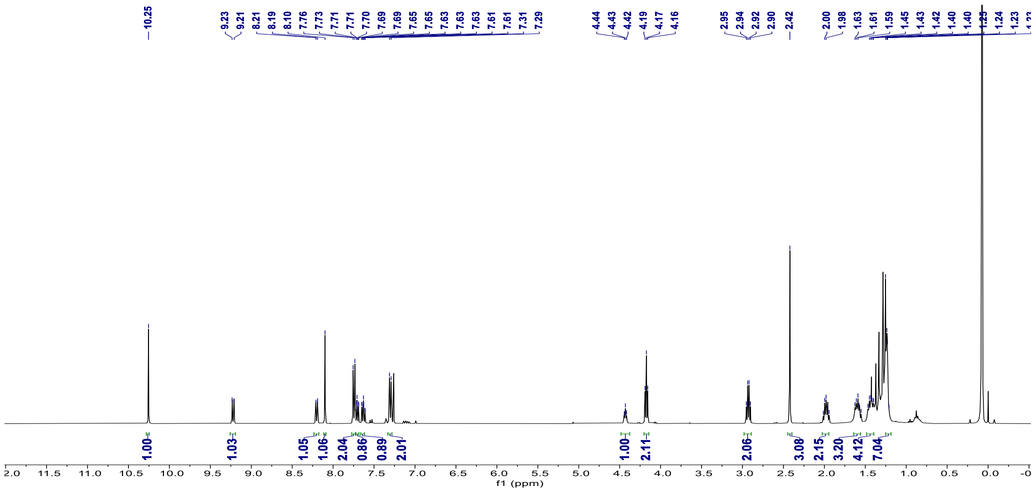


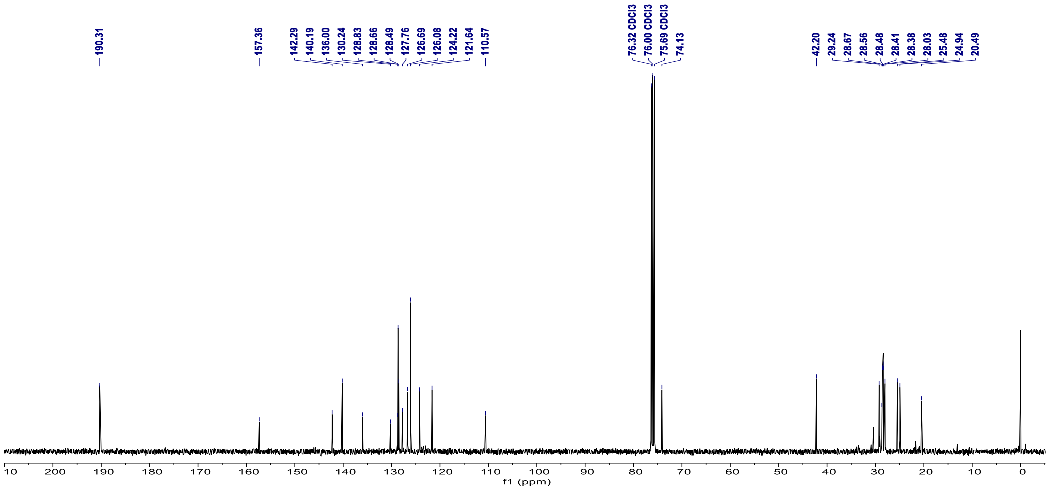


**Supplementary Figure 7.** $^{1}H$ and $^{13}C$ NMR spectra of **S5a**

**Supplementary Table 5.** Macrolactonization of substrate **S5a** with NHC **G**

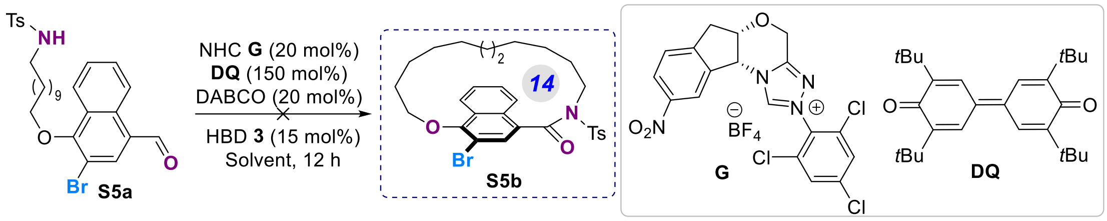


| Entry | Solvent | Temp. (°C) | Results |
|---|---|---|---|
| 1 | Toluene/*n*-Heptane | 100 | no **S5b** was observed |
| 2 | Toluene/*n*-Heptane | 110 | no **S5b** was observed |
| 3 | PhCl | 130 | no **S5b** was observed |
| 4 | Mesitylene | 150 | no **S5b** was observed |

[a] General conditions (unless otherwise specified): **S5a** (8.6 mg, 0.015 mmol, 1.0 equiv.), NHC **G** (20 mol%), **DQ** (9.2 mg, 150 mol%), and DABCO (20-100 mol%) in solvents (1 mM; entries 1-2, toluene/*n*-heptane = 11:9 v/v) under $N_2$ atmosphere at indicated temperature for 12 h.

**Supplementary Table 6.** Additional screening on the macrolactonization of substrate **S5a**

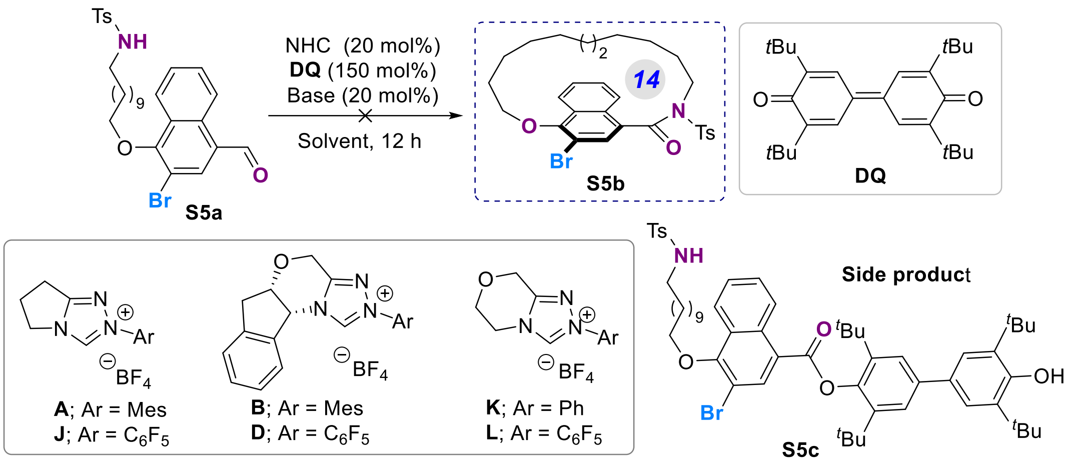


| Entry | NHC | Base | Solvent | Temp. | Result |
|---|---|---|---|---|---|
| 1 | **A, B, K** | $Cs_2CO_3$ or $K_2CO_3$ | THF | 70 | A large amount of **S5a** left |
| 2 | **D, J, L** | $Cs_2CO_3$ or $K_2CO_3$ | THF | 70 | **S5c** was obtained |
| 3 | **A, B, K** | $Cs_2CO_3$ or $K_2CO_3$ | Toluene | 100 | A large amount of **S5a** left |
| 4 | **D, J, L** | $Cs_2CO_3$ or $K_2CO_3$ | Toluene | 100 | **S5c** was obtained |

[a] General conditions (unless otherwise specified): **S5a** (8.6 mg, 0.015 mmol, 1.0 equiv.), NHC (20 mol%), **DQ** (9.2 mg, 150 mol%), and base (20-100 mol%) in solvents (1 mM) under $N_2$ atmosphere at indicated temperature for 12 h.

When 3-Cl substituted precursor **S6a** was subjected to the optimal conditions, no any corresponding product **S6b** was observed with our catalytic conditions or at elevated temperatures (Supplementary Table 7, entries 1-4). Additional screenings with NHCs, solvents and temperatures also failed to give the desired macrocyclization product **S6b** (Supplementary Table 8), wherein particular conditions would give the dimer/trimer side products **S6c** and **S6d**, probably owing to the notable enhanced rigidified macrocyclic structure of **S6b** that poses difficulty on the intramolecular ring closure.

**2-Chloro-4-(3-hydroxypropoxy)-1-naphthaldehyde--methane (S6a)**

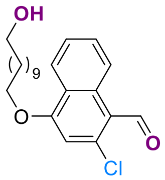


**$^1$H NMR** (400 MHz, $CDCl_3$) δ 10.79 (s, 1H), 9.32 (d, $J$ = 8.6 Hz, 1H), 8.27 (d, $J$ = 8.6 Hz, 1H), 7.71 – 7.65 (m, 1H), 7.56 – 7.51 (m, 1H), 6.81 (s, 1H), 4.21 (t, $J$ = 6.4 Hz, 2H), 3.64 (t, $J$ = 6.6 Hz, 2H), 1.97 (dd, $J$ = 14.9, 6.4 Hz, 2H), 1.60 – 1.53 (m, 5H), 1.37 – 1.28 (m, 11H).

**$^{13}$C NMR** (101 MHz, $CDCl_3$) δ 160.2, 144.1, 132.6, 130.4, 126.4, 125.0, 124.7, 122.3, 119.9, 107.0, 69.2, 63.1, 32.8, 29.6, 29.5, 29.4, 29.3, 28.9, 26.1, 25.7.

**HRMS** (ESI, m/z) Calcd. for $C_{22}H_{29}ClO_3Na^+$, [M+Na] $^+$: 399.1697, found: 399.1691.

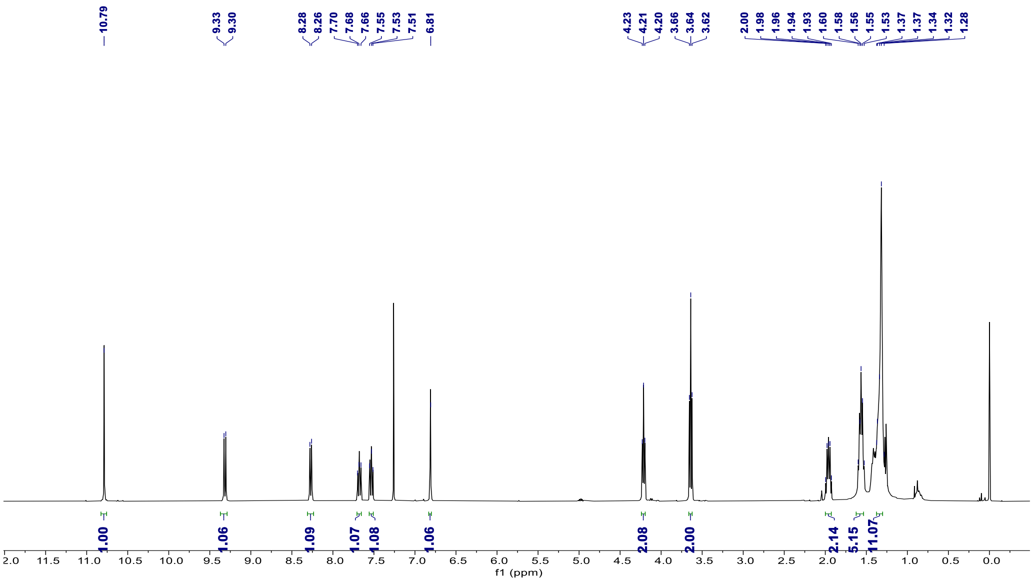

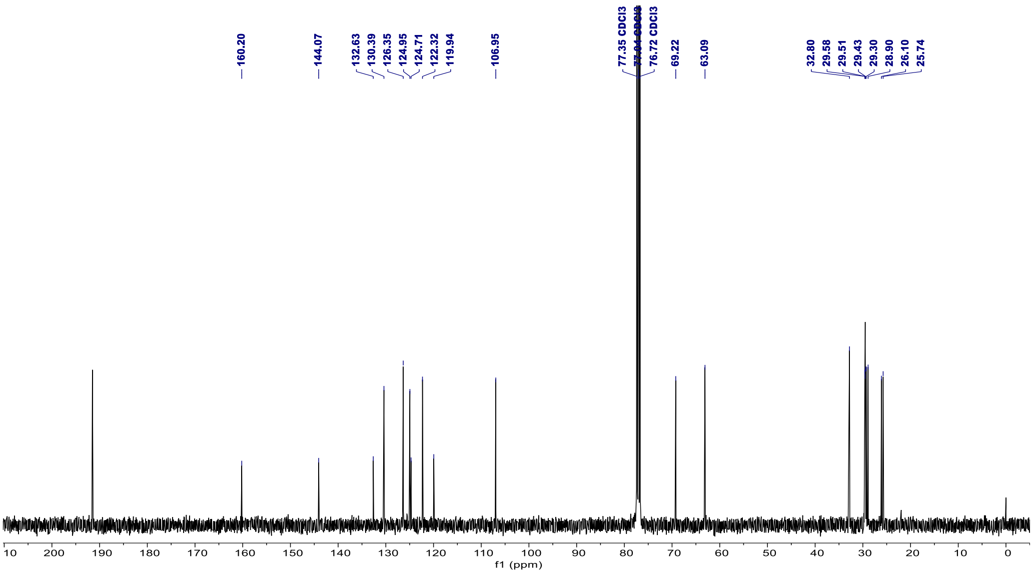


**Supplementary Figure 8.** $^{1}$H and $^{13}$C NMR spectra of **S6a**

**Supplementary Table 7.** Macrolactonization of substrate **S6a** with NHC **G**

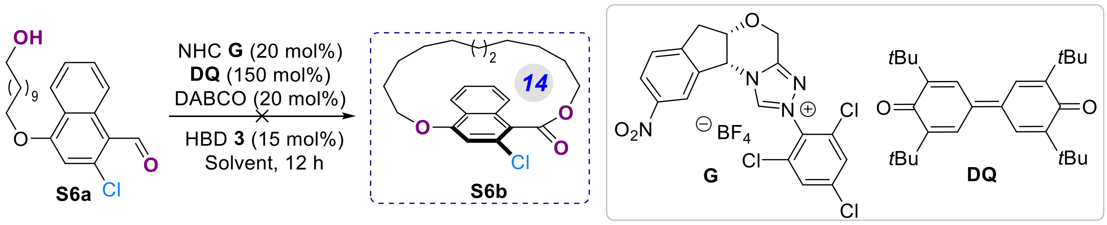


| Entry | Solvent | Temp. (ºC) | Results |
|---|---|---|---|
| 1 | Toluene/*n*-Heptane | 100 | **S6a** remained unreacted |
| 2 | Toluene/*n*-Heptane | 110 | **S6a** remained unreacted |
| 3 | PhCl | 130 | **S6a** remained unreacted |
| 4 | Mesitylene | 150 | **S6a** remained unreacted |

[a] General conditions (unless otherwise specified): **S6a** (5.6 mg, 0.015 mmol, 1.0 equiv.), NHC **G** (20 mol%), **DQ** (9.2 mg, 150 mol%), and DABCO (20 mol%) in solvents (1 mM; entries 1-2, toluene/*n*-heptane = 11:9 v/v) under $N_2$ atmosphere at indicated temperature for 12 h.

**Supplementary Table 8.** Additional screening on the macrolactonization with substrate **S6a**

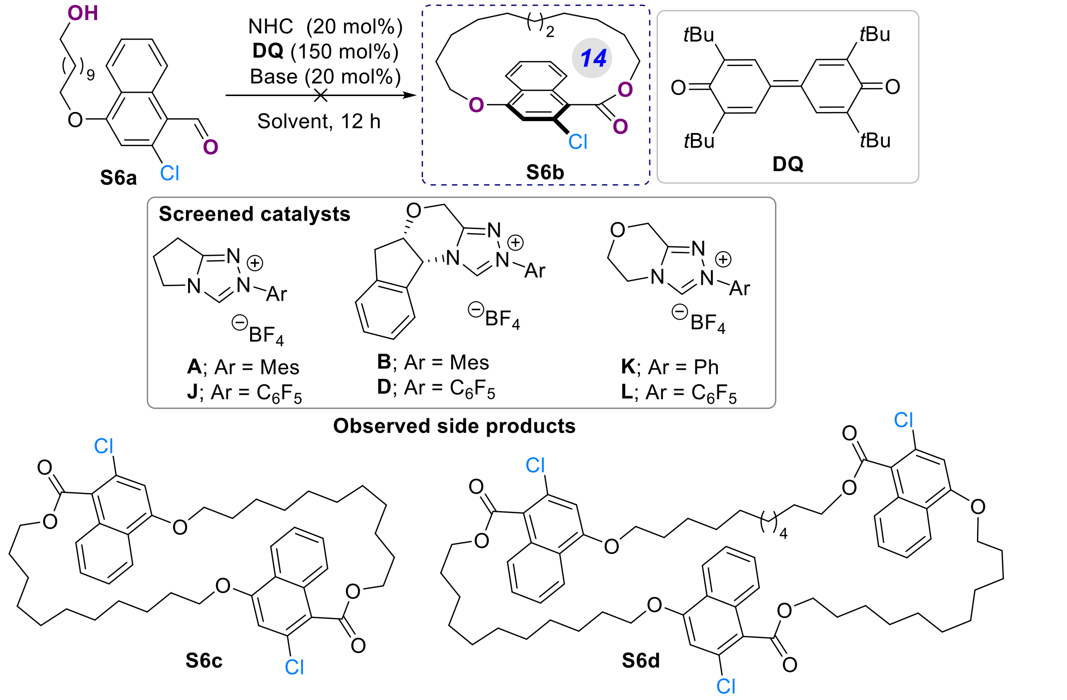


| Entry | NHC | Base | Solvent | Temp. | Results |
|---|---|---|---|---|---|
| 1 | **A, B, D, K, L** | $K_2CO_3$ | THF | 70 | A large amount of **S6a** remained |
| 2 | **J** | $K_2CO_3$ | THF | 70 | **S6c** and **S6d** was isolated; product **S6b** was not observed |
| 3 | **B, D, J-L** | $K_2CO_3$ | Toluene | 100 | A large amount of **S6a** remained |
| 4 | **A** | $K_2CO_3$ | Toluene | 100 | **S6c** and **S6d** was isolated; product **S6b** was not observed |
| 5 | **A, B, D, J** | $Cs_2CO_3$ | THF | 70 | A large amount of **S6a** remained |
| 6 | **K, L** | $Cs_2CO_3$ | THF | 70 | **S6c** and **S6d** was isolated; product **S6b** was not observed |
| 7 | **A, B, D, J** | $Cs_2CO_3$ | Toluene | 100 | A large amount of **S6a** remained |
| 8 | **K, L** | $Cs_2CO_3$ | Toluene | 100 | **S6c** and **S6d** was isolated; product **S6b** was not observed |

[a] General conditions (unless otherwise specified): **S6a** (5.6 mg, 0.015 mmol, 1.0 equiv.), NHC (20 mol%), **DQ** (9.2 mg, 150 mol%), and base (20 mol%) in solvents (1 mM; entries 1-2, toluene/*n*-heptane = 11:9 v/v) under $N_2$ atmosphere at indicated temperature for 12 h.

**1,16-Dichloro-2,14,17,29-tetraoxa-1,16(1,4)-dinaphthalenacyclotriacontaphane-15,30-dione**

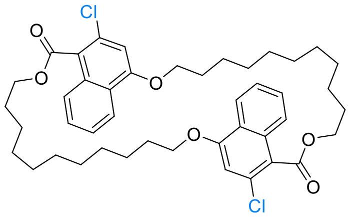


**$^1$H NMR** (400 MHz, $CDCl_3$) δ 8.22 (d, $J$ = 7.8 Hz, 1H), 7.73 (d, $J$ = 8.4 Hz, 1H), 7.54 – 7.48 (m, 1H), 7.43 (ddd, $J$ = 8.2, 6.8, 1.2 Hz, 1H), 6.74 (s, 1H), 4.47 (t, $J$ = 5.8 Hz, 2H), 4.10 (t, $J$ = 6.4 Hz, 2H), 1.88 (p, $J$ = 6.8 Hz, 2H), 1.82 – 1.74 (m, 2H), 1.51 – 1.44 (m, 4H), 1.35 – 1.27 (m, 9H), 0.95 – 0.84 (m, 1H).

**HRMS** (ESI, m/z) Calcd. for $C_{44}H_{54}Cl_2O_6Na^+$, $[M+Na]^+$: 771.3195, found: 771.3189.

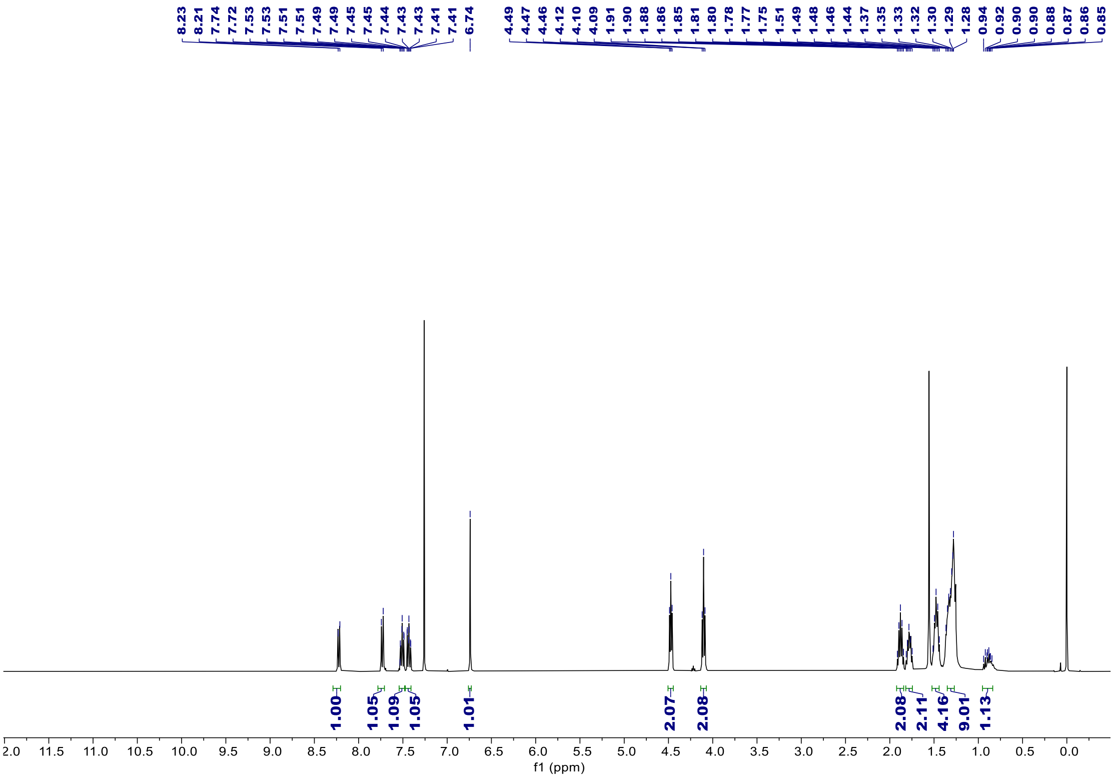


**Supplementary Figure 9.** $^1$H spectra of 1,16-Dichloro-2,14,17,29-tetraoxa-1,16(1,4)-dinaphthalenacyclotriacontaphane-15,30-dione

**1,16,31-trichloro-2,14,17,29,32,44-hexaoxa-1,16,31(1,4)-trinaphthalenacyclopent-atetracontaphane-15,30,45-trione**

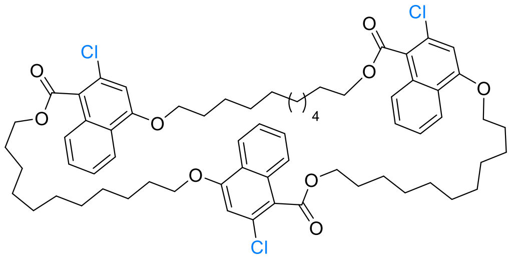


**$^1$H NMR** (400 MHz, $CDCl_3$) δ 8.24 (d, $J$ = 8.4 Hz, 1H), 7.74 (d, $J$ = 8.4 Hz, 1H), 7.57 – 7.50 (m, 1H), 7.50 – 7.43 (m, 1H), 6.75 (s, 1H), 4.46 (t, $J$ = 6.4 Hz, 2H), 4.11 (t, $J$ = 6.4 Hz, 2H), 1.90 (p, $J$ = 6.4 Hz, 2H), 1.78 (dd, $J$ = 13.6, 7.6 Hz, 2H), 1.52 – 1.43 (m, 4H), 1.36 – 1.29 (m, 9H), 0.88 – 0.84 (m, 1H).

**HRMS** (ESI, m/z) Calcd. for $C_{66}H_{81}Cl_3O_9Na^+$, $[M+Na]^+$: 1145.4844, found: 1145.4847.

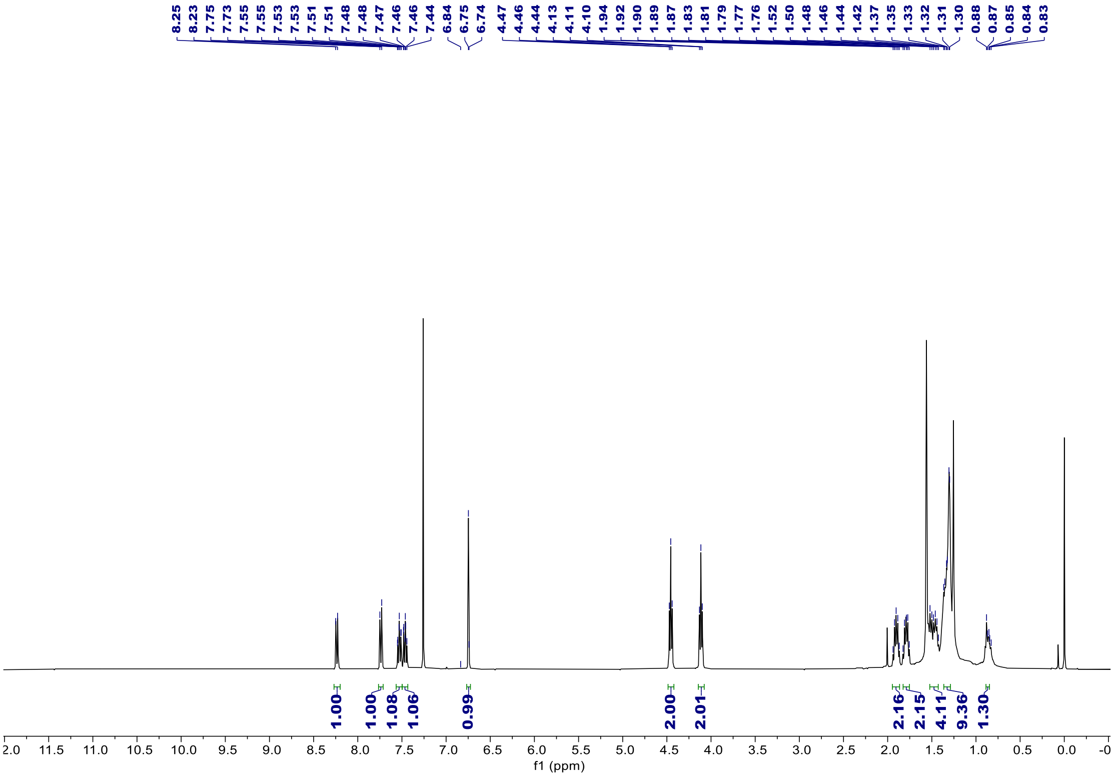


**Supplementary Figure 10.** $^1$H spectra of 1,16,31-trichloro-2,14,17,29,32,44-hexaoxa-1,16,31(1,4)-trinaphthalenacyclopent-atetracontaphane-15,30,45-trione

2,3-Disubstituted precursor, such as **S7a** was also studied for the macrolactonization. Despite extensive conditions have been screened, such as with various NHCs, solvents and temperatures, we could not obtain the corresponding product **S7b** (Supplementary Table 9 and Supplementary Table 10). In these cases, the starting material **S7a** were all remained unreacted, no any dimer/trimer product could be observed, which indicates that the carbene addition to the aldehyde most likely not occurred smoothly to give the key acyl azolium species due to the steric hinderance of the multisubstituted arylcarboxyaldehyde **S7a**.

**3-Bromo-4-(3-hydroxypropoxy)-2-methyl-1-naphthaldehyde-methane (S7a)**

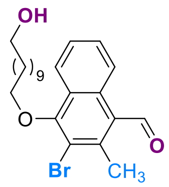


**$^1$H NMR** (400 MHz, $CDCl_3$) δ 10.87 (s, 1H), 8.83 (d, *J* = 8.6 Hz, 1H), 8.14 (d, *J* = 8.4 Hz, 1H), 7.63 (t, *J* = 8.4 Hz, 1H), 7.57 – 7.52 (m, 1H), 4.10 (t, *J* = 6.6 Hz, 2H), 3.64 (t, *J* = 6.6 Hz, 2H), 2.88 (s, 3H), 2.03 – 1.95 (m, 2H), 1.58 (dd, *J* = 13.6, 5.6 Hz, 5H), 1.33 (s, 11H).

**$^{13}$C NMR** (101 MHz, $CDCl_3$) δ 192.9, 157.2, 142.4, 131.7, 129.1, 127.9, 127.5, 126.7, 124.7, 122.6, 118.7, 74.7, 63.1, 32.8, 30.3, 29.6, 29.6, 29.6, 29.5, 29.4, 26.0, 25.8, 19.6.

**HRMS** (ESI, m/z) Calcd. for $C_{23}H_{31}BrO_3Na^+$, $[M+Na]^+$: 457.1349, found: 457.1347.

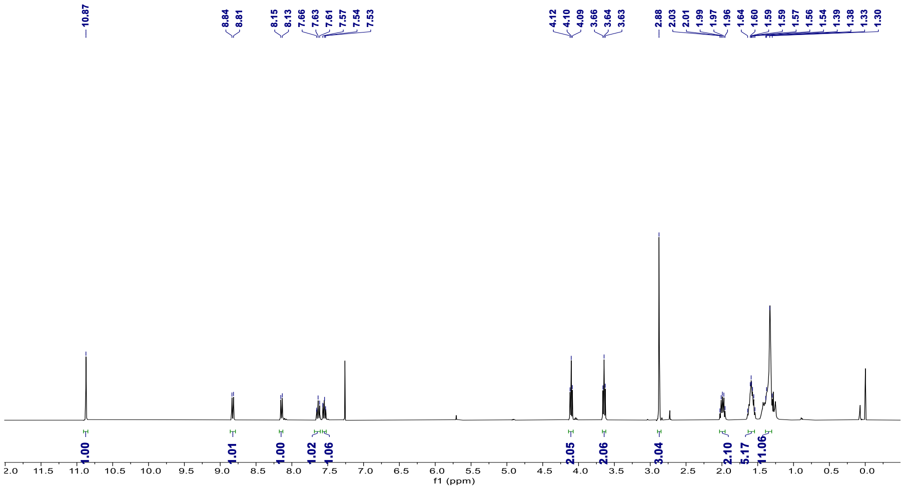

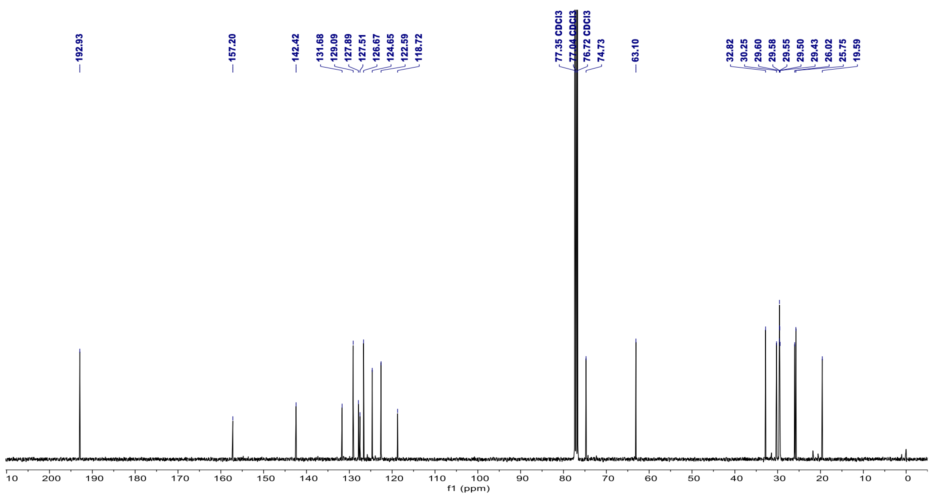


**Supplementary Figure 11.** $^{1}$H and $^{13}$C NMR spectra of **S7a**

**Supplementary Table 9.** Macrolactonization of substrate **S7a** with NHC **G**

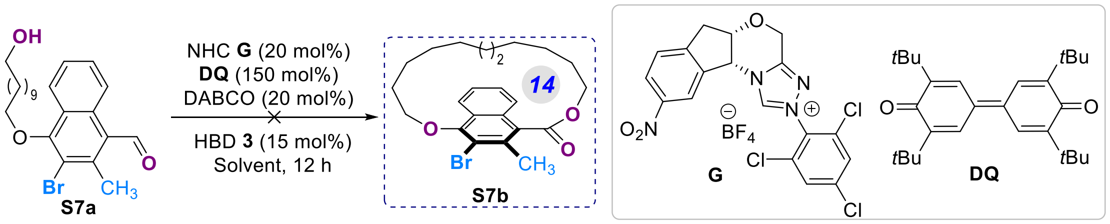


| Entry | Solvent | Temp. (°C) | Results |
|---|---|---|---|
| 1 | Toluene/*n*-Heptane | 100 | **S7a** remained unreacted |
| 2 | Toluene/*n*-Heptane | 110 | **S7a** remained unreacted |
| 3 | PhCl | 130 | **S7a** remained unreacted |
| 4 | Mesitylene | 150 | **S7a** remained unreacted |

[a] General conditions (unless otherwise specified): **S7a** (6.5 mg, 0.015 mmol, 1.0 equiv.), NHC **G** (20 mol%), **DQ** (9.2 mg, 150 mol%), and DABCO (20 mol%) in solvents (1 mM; entries 1-2, toluene/*n*-heptane = 11:9 v/v) under $N_2$ atmosphere at indicated temperature for 12 h.

**Supplementary Table 10.** Additional screening on the macrolactonization with substrate **S7a**

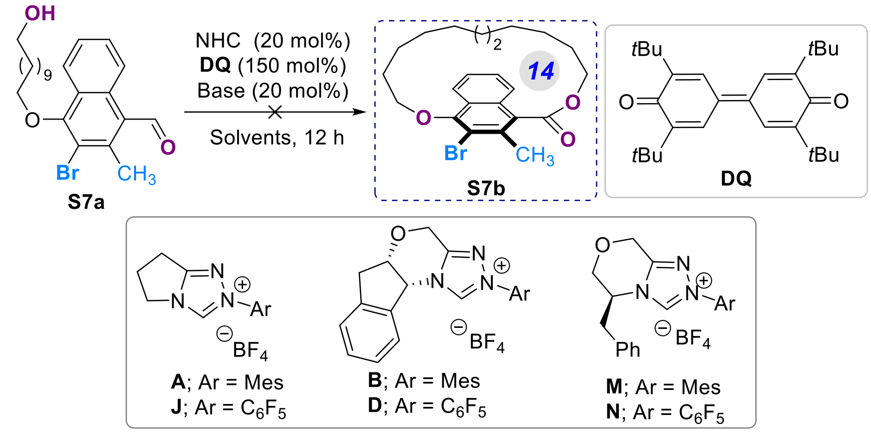


| Entry | NHC | Base | Solvent | Temp. | Result |
|---|---|---|---|---|---|
| 1 | **A, B, D, J, M, N** | $K_2CO_3$ | THF | 70 | **S7a** remained unreacted |
| 2 | **A, B, D, J, M, N** | $Cs_2CO_3$ | THF | 70 | **S7a** remained unreacted |
| 3 | **A, B, D, J, M, N** | $K_2CO_3$ | Toluene | 100 | **S7a** remained unreacted |
| 4 | **A, B, D, J, M, N** | $Cs_2CO_3$ | Toluene | 100 | **S7a** remained unreacted |

[a] General conditions (unless otherwise specified): **S7a** (6.5 mg, 0.015 mmol, 1.0 equiv.), NHC (20 mol%), **DQ** (9.2 mg, 150 mol%), and base (20 mol%) in solvents (1 mM) under $N_2$ atmosphere at indicated temperature for 12 h.

## (7) Intermolecular macrolactonization between S8 and S9

The previous elegant report by Collins *et al.*[18] achieved the enzyme-catalyzed direct intermolecular macrocyclization with dicarboxylic acid and diols as substrates. We applied our carbene catalytic conditions to Collin's reaction system, with acid **S8** and diol **S9** as the two model substrates (Supplementary Table 11). In contrast to the DQ oxidative conditions, carboxylic acid in-situ activation with acyl chloride was used to give the corresponding key acyl azolium intermediate. From our investigations as illustrated in Supplementary Table 11, we were delighted to find that the reaction could deliver to the corresponding macrocyclization product **S10** with NHC **A** and **J** in $CH_2Cl_2$ (entries 1-2, Supplementary Table 11), albeit in relatively low yields. Preliminary studies on the enantioselective catalysis were also performed (entries 3-14). Reaction with NHC **G** in toluene readily afforded the chiral product **S10** in 31% yield and 59:41 er, as a proof-of-concept for the carbene-catalyzed intermolecular macrolactonization strategy for further exploration. (**the absolute configuration of product **S10** was not assigned at this stage)

**Supplementary Table 11.** Investigation on the intermolecular macrolactonization between **S8** and **S9**[a]

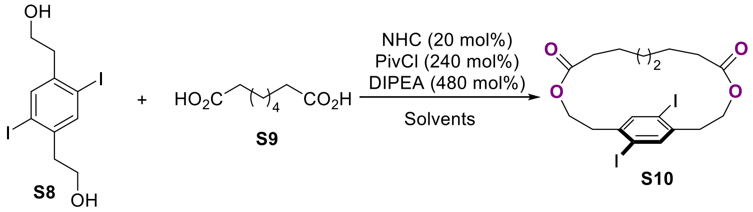


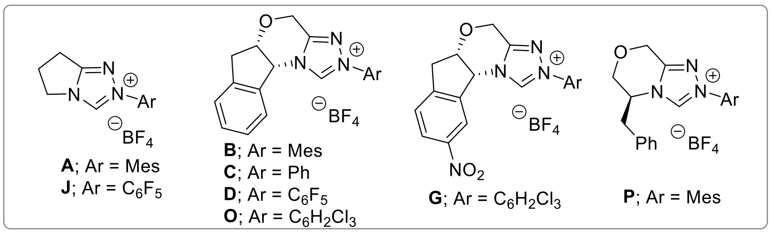


| Entry | NHC | Solvent | Temp. (°C) | Yield (%)[b] | E.r. (%) |
|---|---|---|---|---|---|
| 1 | **A** | $CH_2Cl_2$ | 40 | 28 | 50:50 |
| 2 | **J** | $CH_2Cl_2$ | 40 | 33 | 50:50 |
| 3 | **B** | $CH_2Cl_2$ | 40 | 30 | 50:50 |
| 4 | **C** | $CH_2Cl_2$ | 40 | 0 | n.d. |
| 5 | **D** | $CH_2Cl_2$ | 40 | 32 | 50:50 |
| 6 | **A-D, J** | THF | 70 | 0 | n.d. |

| | | | | | |
|---|---|---|---|---|---|
| 7 | **A** | Toluene | 50 | 0 | n.d. |
| 8 | **A** | Toluene | 100 | 29 | 50:50 |
| 9 | **B** | Toluene | 100 | 20 | 56:44 |
| 10 | **C** | Toluene | 100 | 25 | 52:48 |
| 11 | **D** | Toluene | 100 | 30 | 50:50 |
| 12 | **G** | Toluene | 100 | 31 | **59:41** |
| 13 | **O** | Toluene | 100 | 25 | 55:45 |
| 14 | **P** | Toluene | 100 | 21 | 48:52 |

[a] General conditions (unless otherwise specified): **S8** (4.2 mg, 0.01 mmol, 1.0 equiv.), **S9** (1.7 mg, 0.01 mmol, 1.0 equiv.), NHC (20 mol%), PivCl (2.9 mg, 240 mol%), and DIPEA (6.2 mg, 480 mol%) in solvent (0.1 M) at indicated temperature for 12 h. [b] Yields of **2a** were determined via $^{1}$H NMR analysis with 1,3,5-trimethoxybenzene as an internal standard. n.d. = not determined.

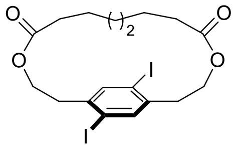

**$^{1}$H NMR** (400 MHz, $CDCl_3$) δ 7.69 (s, 2H), 4.45 (ddd, *J* = 11.1, 7.1, 3.8 Hz, 2H), 4.26 (ddd, *J* = 11.4, 8.0, 3.6 Hz, 2H), 3.25 – 3.11 (m, 2H), 3.01 – 2.86 (m, 2H), 2.34 – 2.16 (m, 5H), 1.42 – 1.35 (m, 4H), 1.08 – 0.95 (m, 4H). (Product matched literature characterization data.[18])

(For entry 12) **HPLC analysis**: 59:41 e.r. (AD-H column, 25 °C, hexane / *i*PrOH = 90 / 10, 0.8 mL / min, λ = 254 nm), Rt (major) = 13.6 min, Rt (minor) = 15.4 min.

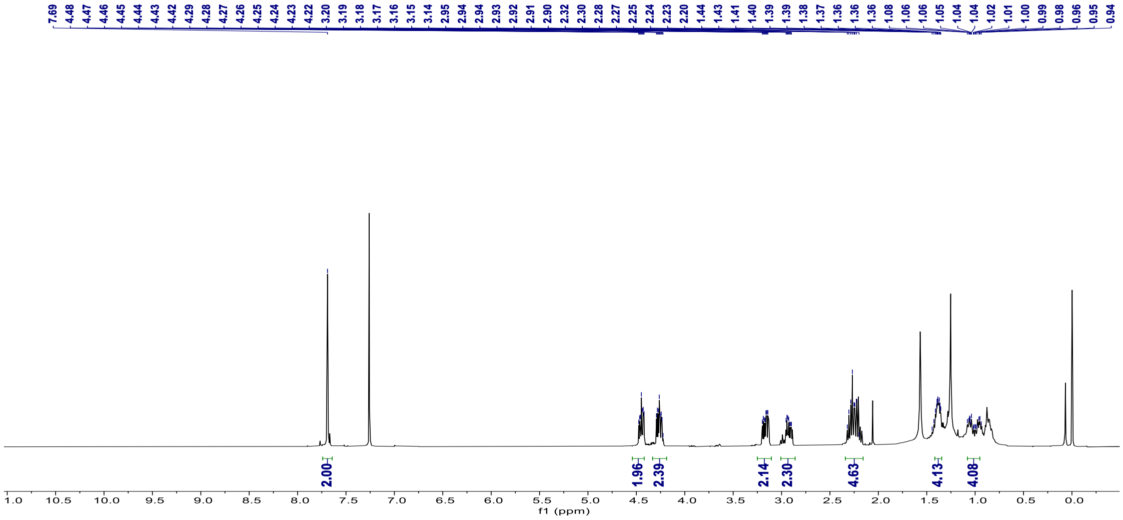

| HPLC of racemic **S10** | HPLC of chiral **S10** |
|---|---|

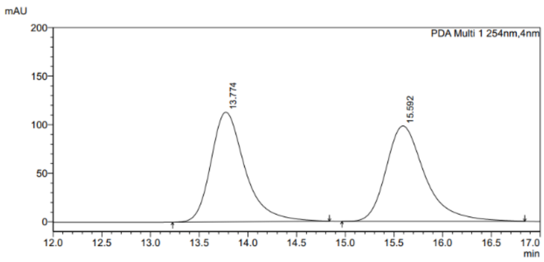


PDA Ch1 254nm

| Peak# | Ret. Time | Area | Area% | Height |
|---|---|---|---|---|
| 1 | 13.774 | 2750323 | 50.110 | 112957 |
| 2 | 15.592 | 2738262 | 49.890 | 98193 |
| Total | | 5488585 | 100.000 | 211150 |

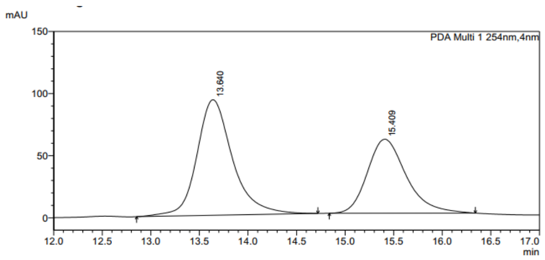


PDA Ch1 254nm

| Peak# | Ret. Time | Area | Area% | Height |
|---|---|---|---|---|
| 1 | 13.640 | 2381659 | 58.924 | 93062 |
| 2 | 15.409 | 1660288 | 41.076 | 59513 |
| Total | | 4041947 | 100.000 | 152575 |

**Supplementary Figure 12.** $^{1}$H and HPLC spectra of **S10**

### (8) X-ray crystallography of chiral product ($R_p$)-2p

Good quality crystal of product **2p** (colorless block crystal) was obtained by evaporation of an acetonitrile/$H_2O$ solution of compound **2p** (~10 mg). CCDC: **1849177** contain the supplementary crystallographic data for this paper. These data can be obtained free of charge from The Cambridge Crystallographic Data Centre via www.ccdc.cam.ac.uk/data_request/cif.

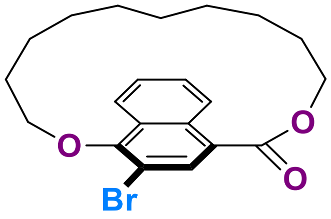


($R_p$)-**2p**

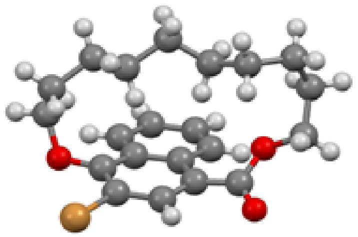

CCDC: **1849177**

# II. Supplementary Notes

## Characterization of substrates and planar chiral products

### (1) Characterization of the prepared substrates

**3-Bromo-4-((11-((tert-butyldimethylsilyl)oxy)undecyl)oxy)-1-naphthaldehyde (S2)**

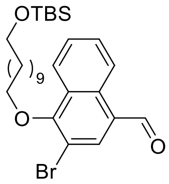

Prepared according to general procedure B (step 1), colorless oil, 51% yield, 272.4 mg.

**$^{1}$H NMR** (400 MHz, $CDCl_3$) δ 10.26 (s, 1H), 9.22 (d, *J* = 9.2 Hz, 1H), 8.21 (d, *J* = 9.2 Hz, 1H), 8.10 (s, 1H), 7.71 (ddd, *J* = 8.4, 6.8, 1.2 Hz, 1H), 7.63 (ddd, *J* = 8.4, 6.8, 1.2 Hz, 1H), 4.18 (t, *J* = 6.6 Hz, 2H), 3.60 (t, *J* = 6.6 Hz, 2H), 2.02 – 1.94 (m, 2H), 1.63 – 1.60 (m, 1H), 1.53 – 1.48 (m, 2H), 1.36 – 1.26 (m, 13H), 0.89 (s, 9H), 0.05 (s, 6H).

**$^{13}$C NMR** (101 MHz, $CDCl_3$) δ 157.0, 138.6, 130.8, 129.9, 129.4, 128.4, 127.7, 125.1, 122.7, 122.3, 75.1, 63.4, 32.9, 30.3, 29.6, 29.6, 29.5, 29.4, 26.0, 26.0, 25.8, 18.4, -5.2.

**HRMS** (ESI, m/z) Calcd. for $C_{29}H_{43}BrO_3Si^+$, [M+H] $^+$: 535.2243, found: 535.2165.

**4-((11-((tert-Butyldimethylsilyl)oxy)undecyl)oxy)-3-phenyl-1-naphthaldehyde (S3a)**

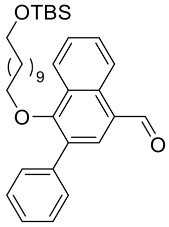

Prepared according to general procedure B (step 1), colorless oil, 82% yield, 436.5 mg.

**$^{1}$H NMR** (400 MHz, $CDCl_3$) δ 10.33 (s, 1H), 9.31 (d, *J* = 8.4 Hz, 1H), 8.35 (d, *J* = 8.4 Hz, 1H), 8.01 (s, 1H), 7.71 – 7.65 (m, 3H), 7.52 – 7.47 (m, 2H), 7.44 – 7.39 (m, 1H), 3.68 (t, *J* = 6.4 Hz, 2H), 3.60 (t, *J* = 6.4 Hz, 2H), 1.67 – 1.59 (m, 4H), 1.51 (t, *J* = 6.8 Hz, 2H), 1.30 – 1.21 (m, 12H), 0.89 (s, 9H), 0.05 (s, 6H).

**$^{13}$C NMR** (101 MHz, $CDCl_3$) δ 192.7, 158.4, 141.1, 137.7, 131.5, 129.3, 128.9, 128.6, 127.8, 127.5, 127.1, 125.0, 123.2, 74.5, 63.4, 32.9, 30.2, 29.6, 29.5, 29.5, 29.4, 29.3, 26.0, 25.9, 25.8, 18.4, -5.2.

**HRMS** (ESI, m/z) Calcd. for $C_{34}H_{48}O_3SiNa^+$, [M+Na] $^+$: 555.3270, found: 555.3265.

**4-((11-((tert-Butyldimethylsilyl)oxy)undecyl)oxy)-3-(4-chlorophenyl)-1-naphthaldehyde (S3b)**

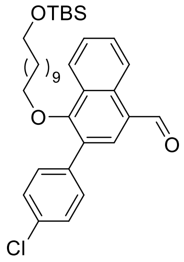

Prepared according to general procedure B (step 1), colorless oil, 80% yield, 453.0 mg.

**$^1$H NMR** (400 MHz, $CDCl_3$) δ 10.34 (s, 1H), 9.29 (d, *J* = 9.2 Hz, 1H), 8.33 (d, *J* = 7.6 Hz, 1H), 7.96 (s, 1H), 7.74 – 7.69 (m, 1H), 7.67 – 7.62 (m, 3H), 7.51 – 7.46 (m, 2H), 3.68 (t, *J* = 6.4 Hz, 2H), 3.60 (t, *J* = 6.6 Hz, 2H), 1.69 – 1.63 (m, 2H), 1.51 (t, *J* = 6.8 Hz, 2H), 1.32 – 1.21 (m, 14H), 0.89 (s, 9H), 0.05 (s, 6H).

**$^{13}$C NMR** (101 MHz, $CDCl_3$) δ 192.5, 158.4, 140.3, 136.1, 133.8, 131.7, 130.7, 129.5, 129.3, 128.9, 127.9, 127.7, 127.3, 125.0, 123.2, 74.7, 63.4, 32.9, 30.2, 29.6, 29.5, 29.5, 29.5, 29.3, 26.0, 25.9, 25.8, 18.4. -4.2.

**HRMS** (ESI, m/z) Calcd. for $C_{34}H_{47}ClO_3SiNa^+$, $[M+Na]^+$: 589.2881, found: 589.2875.

**4-((11-((Tert-butyldimethylsilyl)oxy)undecyl)oxy)-3-(p-tolyl)-1-naphthaldehyde (S3c)**

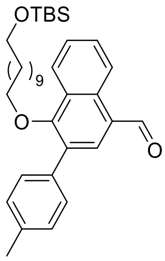

Prepared according to general procedure B (step 1), colorless oil, 71% yield, 387.8 mg.

**$^1$H NMR** (400 MHz, $CDCl_3$) δ 10.32 (s, 1H), 9.30 (d, *J* = 8.4 Hz, 1H), 8.34 (d, *J* = 9.2 Hz, 1H), 7.99 (s, 1H), 7.70 (ddd, *J* = 8.4, 6.8, 1.6 Hz, 1H), 7.63 (ddd, *J* = 8.4, 6.8, 1.2 Hz, 1H), 7.59 (d, *J* = 1.6 Hz, 2H), 7.30 (d, *J* = 7.6 Hz, 2H), 3.69 (t, *J* = 6.6 Hz, 2H), 3.60 (t, *J* = 6.6 Hz, 2H), 2.44 (s, 3H), 1.69 – 1.62 (m, 2H), 1.54 – 1.48 (m, 2H), 1.33 – 1.24 (m, 14H), 0.90 (s, 9H), 0.05 (s, 6H).

**$^{13}$C NMR** (101 MHz, $CDCl_3$) δ 158.3, 141.2, 137.5, 134.7, 131.4, 129.3, 129.3, 129.2, 129.1, 128.9, 127.4, 127.1, 124.9, 123.2, 74.3, 63.3, 32.9, 30.2, 29.6, 29.5, 29.5, 29.5, 29.3, 26.0, 25.9, 25.8, 21.3, 18.4, -6.7.

**HRMS** (ESI, m/z) Calcd. for $C_{34}H_{47}ClO_3SiNa^+$, [M+Na] $^+$: 589.2881, found: 589.2875.

**4-((11-((Tert-butyldimethylsilyl)oxy)undecyl)oxy)-3-(4-methoxyphenyl)-1-naphthaldehyde (S3d)**

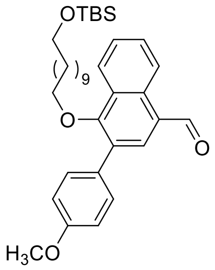


Prepared according to general procedure B (step 1), colorless oil, 75% yield, 421.7 mg.

**$^1$H NMR** (400 MHz, $CDCl_3$) δ 10.32 (s, 1H), 9.29 (d, *J* = 8.8 Hz, 1H), 8.33 (d, *J* = 10.0 Hz, 1H), 7.99 (s, 1H), 7.71 – 7.67 (m, 1H), 7.65 – 7.60 (m, 3H), 7.06 – 7.00 (m, 2H), 3.89 (s, 3H), 3.69 (t, *J* = 6.6 Hz, 2H), 3.60 (t, *J* = 6.6 Hz, 2H), 1.69 – 1.63 (m, 2H), 1.55 – 1.48 (m, 2H), 1.36 – 1.23 (m, 14H), 0.89 (s, 9H), 0.05 (s, 6H).

**$^{13}$C NMR** (101 MHz, $CDCl_3$) δ 192.7, 159.4, 158.2, 141.1, 131.4, 130.5, 130.0, 129.5, 129.1, 128.7, 127.6, 127.1, 125.0, 123.2, 114.1, 74.3, 63.4, 55.4, 32.9, 30.2, 29.7, 29.6, 29.5, 29.3, 26.0, 25.9, 18.4, -5.2.

**HRMS** (ESI, m/z) Calcd. for $C_{35}H_{51}O_4Si^+$, [M+H] $^+$: 563,3557, found: 563.3612.

**1-((11-((Tert-butyldimethylsilyl)oxy)undecyl)oxy)-[2,2'-binaphthalene]-4-carbaldehyde (S3e)**

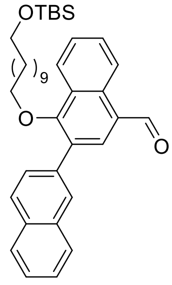


Prepared according to general procedure B (step 1), colorless oil, 65% yield, 378.5 mg.

**$^1$H NMR** (400 MHz, $CDCl_3$) δ 10.37 (s, 1H), 9.33 (d, *J* = 10.4 Hz, 1H), 8.39 (d, *J* = 10.0 Hz, 1H), 8.15 (d, *J* = 1.6 Hz, 1H), 8.13 (s, 1H), 7.98 – 7.91 (m, 3H), 7.85 (dd, *J* = 8.4, 1.8 Hz, 1H), 7.73 (ddd, *J* = 8.4, 6.8, 1.6 Hz, 1H), 7.68 – 7.64 (m, 1H), 7.57 – 7.53 (m, 2H), 3.69 (d, *J* = 6.4 Hz, 2H), 3.59 (t, *J* = 6.6 Hz, 2H), 1.52 – 1.47 (m, 2H), 1.30 – 1.20 (m, 10H), 1.11 (s, 6H), 0.89 (s, 9H), 0.05 (s, 6H).

**$^{13}$C NMR** (101 MHz, $CDCl_3$) δ 192.7, 158.6, 141.2, 135.2, 133.5, 132.7, 131.6, 129.4, 128.8, 128.2, 128.2, 127.8, 127.6, 127.3, 127.2, 126.5, 126.4, 125.0, 123.2, 74.6, 63.4, 32.9, 30.2, 29.6, 29.5, 29.4, 29.2, 26.0, 25.9, -5.2.

**HRMS** (ESI, m/z) Calcd. for $C_{38}H_{50}O_3SiNa^+$, $[M+Na]^+$: 605.3427, found: 605.3421.

**4-((11-((Tert-butyldimethylsilyl)oxy)undecyl)oxy)-3-(thiophen-3-yl)-1-naphthaldehyde (S3f)**

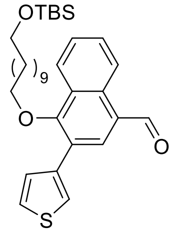


Prepared according to general procedure B (step 1), colorless oil, 51% yield, 274.5 mg.

**$^1$H NMR** (400 MHz, $CDCl_3$) δ 10.34 (s, 1H), 9.27 (d, $J$ = 8.4 Hz, 1H), 8.31 (d, $J$ = 8.4 Hz, 1H), 8.10 (s, 1H), 7.73 – 7.63 (m, 3H), 7.57 (dd, $J$ = 5.0, 1.2 Hz, 1H), 7.46 (dd, $J$ = 5.0, 3.0 Hz, 1H), 3.77 (t, $J$ = 6.6 Hz, 2H), 3.60 (t, $J$ = 6.6 Hz, 2H), 1.79 – 1.72 (m, 2H), 1.53 – 1.48 (m, 2H), 1.44 – 1.39 (m, 2H), 1.30 – 1.26 (m, 12H), 0.90 (s, 9H), 0.05 (s, 6H).

**$^{13}$C NMR** (101 MHz, $CDCl_3$) δ 191.6, 157.0, 139.0, 136.5, 130.4, 128.4, 128.2, 127.2, 126.6, 126.2, 124.7, 123.9, 123.2, 122.8, 122.1, 73.4, 62.3, 57.5, 31.9, 29.3, 28., 28.4, 28.3, 25.0, 24.8, 17.4, 17.4, -6.3.

**HRMS** (ESI, m/z) Calcd. for $C_{32}H_{46}O_3SSiNa^+$, $[M+Na]^+$: 561.2835, found: 561.2829.

**4-((11-((Tert-butyldimethylsilyl)oxy)undecyl)oxy)-3-(furan-3-yl)-1-naphthaldehyde (S3g)**

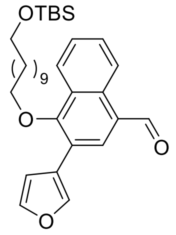


Prepared according to general procedure B (step 1), colorless oil, 65% yield, 339.5 mg.

**$^1$H NMR** (400 MHz, $CDCl_3$) δ 10.36 (s, 1H), 9.25 (d, $J$ = 8.8 Hz, 1H), 8.25 (d, $J$ = 10.0 Hz, 1H), 8.08 (d, $J$ = 5.2 Hz, 2H), 7.70 – 7.61 (m, 2H), 7.58 (t, $J$ = 1.6 Hz, 1H), 6.95 (dd, $J$ = 2.0, 0.8 Hz, 1H), 3.90 (t, $J$ = 6.8 Hz, 2H), 3.60 (t, $J$ = 6.6 Hz, 2H), 1.91– 1.86 (m, 2H), 1.54 – 1.46 (m, 5H), 1.33 – 1.28 (m, 11H), 0.89 (s, 9H), 0.05 (s, 6H).

$^{13}$**C NMR** (101 MHz, $CDCl_3$) δ 191.5, 142.3, 140.5, 137.4, 128.3, 128.0, 126.2, 124.0, 121.9, 120.5, 109.0, 73.1, 62.3, 31.9, 29.4, 28.6, 28.5, 28.4, 24.8, 17.4, -5.8.

**HRMS** (ESI, m/z) Calcd. for $C_{33}H_{46}O4Si^+$, $[M+H]^+$: 523.3244, found: 523.3421.

**3-(Benzo[b]thiophen-3-yl)-4-((11-((tert-butyldimethylsilyl)oxy)undecyl)oxy)-1-naphthaldehyde (S3h)**

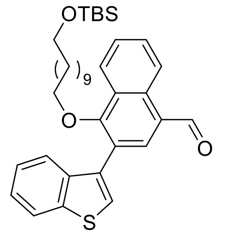


Prepared according to general procedure B (step 1), colorless oil, 55% yield, 323.6 mg.

$^{1}$**H NMR** (400 MHz, $CDCl_3$) δ 10.30 (s, 1H), 9.35 (d, $J$ = 9.6 Hz, 1H), 8.39 (d, $J$ = 7.6 Hz, 1H), 8.04 (s, 1H), 7.99 – 7.94 (m, 1H), 7.75 (ddd, $J$ = 8.4, 6.8, 1.4 Hz, 1H), 7.71 – 7.64 (m, 2H), 7.61 (s, 1H), 7.45 – 7.39 (m, 2H), 3.65 (t, $J$ = 6.6 Hz, 2H), 3.61 (t, $J$ = 6.6 Hz, 2H), 1.53 – 1.47 (m, 4H), 1.33 – 1.23 (m, 7H), 1.15 – 1.04 (m, 7H), 0.90 (s, 9H), 0.06 (s, 6H).

$^{13}$**C NMR** (101 MHz, $CDCl_3$) δ 192.4, 159.3, 141.4, 140.0, 138.3, 133.4, 129.6, 129.3, 127.3, 127.2, 125.5, 125.1, 124.7, 124.5, 123.3, 123.3, 122.8, 122.6, 74.5, 63.4, 32.9, 30.1, 29.6, 29.5, 29.4, 29.1, 26.9, 26.0, 25.8, 25.8, 18.4, -5.2.

**HRMS** (ESI, m/z) Calcd. for $C_{36}H_{48}O_3SSiNa^+$, $[M+Na]^+$: 611.2991, found: 611.2986.

**3-(Benzofuran-3-yl)-4-((11-((tert-butyldimethylsilyl)oxy)undecyl)oxy)-1-naphthaldehyde (S3i)**

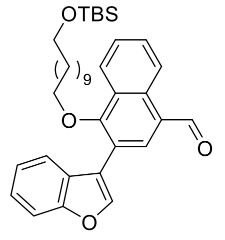


Prepared according to general procedure B (step 1), colorless oil, 58% yield, 331.9 mg.

$^{1}$**H NMR** (400 MHz, $CDCl_3$) δ 10.36 (s, 1H), 9.32 (d, $J$ = 8.4 Hz, 1H), 8.35 (d, $J$ = 9.2 Hz, 1H), 8.18 (s, 1H), 8.04 (s, 1H), 7.79 – 7.72 (m, 2H), 7.68 – 7.60 (m, 2H), 7.40 (ddd, $J$ = 8.4, 7.2, 1.4 Hz, 1H), 7.34 (td, $J$ = 7.2, 1.2 Hz, 1H), 3.80 (t, $J$ = 6.6 Hz, 2H), 3.60 (t, $J$ = 6.6 Hz, 2H), 1.68 – 1.63 (m, 2H), 1.55 – 1.48 (m, 2H), 1.32 – 1.26 (m, 7H), 1.18 (s, 7H), 0.90 (s, 9H), 0.05 (s, 6H).

**$^{13}$C NMR** (101 MHz, $CDCl_3$) δ 192.4, 158.9, 155.4, 143.6, 140.0, 131.7, 129.4, 127.8, 127.3, 126.7, 125.1, 124.9, 123.2, 121.2, 119.7, 117.8, 111.8, 74.5, 63.4, 32.9, 30.2, 29.6, 29.5, 29.5, 29.2, 26.01, 25.9, 25.8, 18.4, -5.2.

**HRMS** (ESI, m/z) Calcd. for $C_{36}H_{48}O_4SiNa^+$, $[M+Na]^+$: 595.3220, found: 595.3214.

**3-Bromo-4-((11-hydroxyundecyl)oxy)-1-naphthaldehyde (1a)**

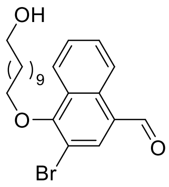


Prepared according to general procedure A, colorless oil, 60% yield, 252.0 mg.

**$^{1}$H NMR** (400 MHz, $CDCl_3$) δ 10.26 (s, 1H), 9.23 (d, $J$ = 9.2 Hz, 1H), 8.21 (d, $J$ = 8.4 Hz, 1H), 8.11 (s, 1H), 7.72 (ddd, $J$ = 8.4, 6.8, 1.2 Hz, 1H), 7.64 (ddd, $J$ = 8.4, 6.8, 1.2 Hz, 1H), 4.18 (t, $J$ = 6.6 Hz, 2H), 3.67 – 3.62 (m, 2H), 2.03 – 1.96 (m, 2H), 1.62 – 1.57 (m, 3H), 1.45 – 1.31 (m, 13H).

**$^{13}$C NMR** (101 MHz, $CDCl_3$) δ 191.5, 159.5, 146.4, 133.2, 129.3, 125.3, 124.6, 124.5, 122.2, 121.5, 108.3, 68.6, 63.0, 32.8, 29.6, 29.5, 29.4, 29.3, 29.1, 26.2, 25.8, 20.8.

**HRMS** (ESI, m/z) Calcd. for $C_{22}H_{29}BrO_3^+$, $[M+H]^+$: 421.1378, found: 421.1503.

**3-Chloro-4-((11-hydroxyundecyl)oxy)-1-naphthaldehyde (1b)**

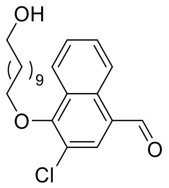


Prepared according to general procedure A, colorless oil, 65% yield, 244.5 mg.

**$^{1}$H NMR** (400 MHz, $CDCl_3$) δ 10.26 (s, 1H), 9.21 (d, $J$ = 8.0 Hz, 1H), 8.22 (d, $J$ = 8.0 Hz, 1H), 7.95 (s, 1H), 7.66 (dddd, $J$ = 24.4, 8.4, 6.8, 1.2 Hz, 2H), 4.21 (t, $J$ = 6.8 Hz, 2H), 3.64 (t, $J$ = 6.8 Hz, 2H), 1.95 (dd, $J$ = 14.0, 7.6 Hz, 2H), 1.60– 1.54 (m, 4H), 1.35– 1.29 (m, 12H).

**$^{13}$C NMR** (101 MHz, $CDCl_3$) δ 191.3, 157.0, 138.6, 130.8, 129.9, 129.4, 128.4, 127.7, 125.1, 122.6, 122.3, 75.1, 63.1, 32.8, 30.3, 29.6, 29.6, 29.5, 29.4, 26.0, 25.8, 21.8.

**HRMS** (ESI, m/z) Calcd. for $C_{22}H_{29}ClO_3Na^+$, $[M+Na]^+$: 399.1703, found: 399.1697.

**4-((11-Hydroxyundecyl)oxy)-3-iodo-1-naphthaldehyde (1c)**

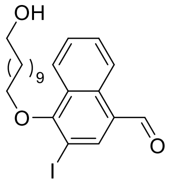


Prepared according to general procedure A, colorless oil, 43% yield, 201.3 mg.

**$^{1}$H NMR** (400 MHz, $CDCl_3$) δ 10.23 (s, 1H), 9.23 (dd, *J* = 8.8, 1.2 Hz, 1H), 8.27 (s, 1H), 8.16 (dd, *J* = 8.8, 1.2 Hz, 1H), 7.71 (ddd, *J* = 8.4, 6.8, 1.2 Hz, 1H), 7.62 (ddd, *J* = 8.4, 6.8, 1.2 Hz, 1H), 4.11 (t, *J* = 6.6 Hz, 2H), 3.64 (t, *J* = 6.6 Hz, 2H), 2.05 – 1.99 (m, 2H), 1.62 – 1.55 (m, 4H), 1.41 – 1.30 (m, 12H).

**$^{13}$C NMR** (101 MHz, $CDCl_3$) δ 191.3, 161.6, 146.6, 132.1, 129.7, 129.3, 129.0, 127.6, 125.4, 122.8, 85.5, 77.4, 76.7, 63.1, 32.8, 30.3, 29.6, 29.6, 29.6, 29.5, 29.4, 26.1, 25.8.

**HRMS** (ESI, m/z) Calcd. for $C_{22}H_{29}IO_3Na^+$, $[M+Na]^+$: 491.1059, found: 491.1054.

**4-((11-Hydroxyundecyl)oxy)-3-phenyl-1-naphthaldehyde (1d)**

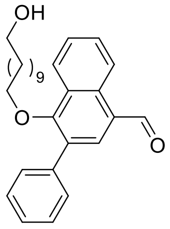


Prepared according to general procedure B (step 2), colorless oil, 75% yield, 313.7 mg.

**$^{1}$H NMR** (400 MHz, $CDCl_3$) δ 10.31 (s, 1H), 9.30 (d, *J* = 8.4 Hz, 1H), 8.35 (d, *J* = 8.4 Hz, 1H), 7.99 (s, 1H), 7.72 – 7.61 (m, 4H), 7.49 (dd, *J* = 8.4, 6.6 Hz, 2H), 7.44 – 7.38 (m, 1H), 3.68 (t, *J* = 6.6 Hz, 2H), 3.64 (t, *J* = 6.6 Hz, 2H), 1.65 – 1.52 (m, 4H), 1.35 – 1.17 (m, 14H).

**$^{13}$C NMR** (101 MHz, $CDCl_3$) δ 192.7, 158.3, 141.1, 137.7, 131.5, 129.3, 128.9, 128.6, 127.7, 127.5, 127.1, 124.9, 123.2, 74.5, 63.0, 32.8, 30.1, 29.6, 29.5, 29.4, 29.2, 25.9, 25.8.

**HRMS** (ESI, m/z) Calcd. for $C_{28}H_{34}O_3Na^+$, $[M+Na]^+$: 441.2406, found: 441.2400.

**3-(4-Chlorophenyl)-4-((11-hydroxyundecyl)oxy)-1-naphthaldehyde (1e)**

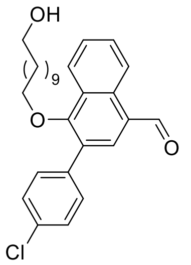


Prepared according to general procedure B (step 2), colorless oil, 60% yield, 271.3 mg.

**$^1$H NMR** (400 MHz, $CDCl_3$) δ 10.34 (s, 1H), 9.29 (d, $J$ = 9.2 Hz, 1H), 8.32 (d, $J$ = 8.4 Hz, 1H), 7.97 (s, 1H), 7.75 – 7.70 (m, 1H), 7.67 – 7.62 (m, 3H), 7.49 – 7.46 (m, 2H), 3.69 (t, $J$ = 6.6 Hz, 2H), 3.64 (t, $J$ = 6.6 Hz, 2H), 1.67 – 1.54 (m, 9H), 1.31 – 1.24 (m, 9H).

**$^{13}$C NMR** (101 MHz, $CDCl_3$) δ 192.4, 158.3, 140.2, 136.1, 133.9, 131.7, 130.7, 129.5, 129.3, 128.9, 127.9, 127.8, 127.3, 125.0, 123.2, 74.8, 63.1, 32.8, 30.2, 29.6, 29.5, 29.4, 29.3, 25.9, 25.8.

**HRMS** (ESI, m/z) Calcd. for $C_{28}H_{33}ClO_3Na^+$, $[M+Na]^+$: 475.2016, found: 475.2010.

**4-((11-Hydroxyundecyl)oxy)-3-(p-tolyl)-1-naphthaldehyde (1f)**

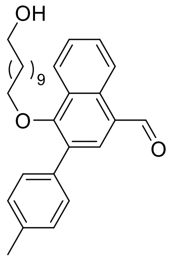


Prepared according to general procedure B (step 2), colorless oil, 66% yield, 285.2 mg.

**$^1$H NMR** (400 MHz, $CDCl_3$) δ 10.32 (s, 1H), 9.29 (d, $J$ = 9.2 Hz, 1H), 8.34 (d, $J$ = 8.0 Hz, 1H), 7.99 (s, 1H), 7.70 (ddd, $J$ = 8.4, 6.8, 1.6 Hz, 1H), 7.63 (ddd, $J$ = 8.4, 6.8, 1.6 Hz, 1H), 7.57 (d, $J$ = 6.8 Hz, 2H), 7.31 (s, 2H), 3.69 (t, $J$ = 6.6 Hz, 2H), 3.64 (t, $J$ = 6.6 Hz, 2H), 2.44 (s, 3H), 1.65 – 1.54 (m, 6H), 1.32 – 1.19 (m, 12H).

**$^{13}$C NMR** (101 MHz, $CDCl_3$) δ 192.7, 158.3, 141.2, 137.6, 134.8, 131.4, 129.4, 129.3, 129.2, 128.9, 127.5, 127.1, 125.0, 123.2, 74.3, 63.1, 32.8, 30.2, 29.6, 29.5, 29.3, 25.9, 25.8, 21.3.

**HRMS** (ESI, m/z) Calcd. for $C_{29}H_{36}O_3Na^+$, $[M+Na]^+$: 455.2562, found: 455.2557.

**4-((11-Hydroxyundecyl)oxy)-3-(4-methoxyphenyl)-1-naphthaldehyde (1g)**

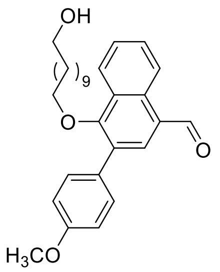


Prepared according to general procedure B (step 2), colorless oil, 72% yield, 322.8 mg.

**$^1$H NMR** (400 MHz, $CDCl_3$) δ 10.32 (s, 1H), 9.29 (d, *J* = 10.0 Hz, 1H), 8.33 (d, *J* = 10.0 Hz, 1H), 7.99 (s, 1H), 7.69 (ddd, *J* = 8.4, 6.8, 1.6 Hz, 1H), 7.65 – 7.60 (m, 3H), 7.15 – 7.11 (m, 2H), 7.05 – 6.99 (m, 2H), 6.83 – 6.80 (m, 2H), 4.19 – 4.15 (m, 1H), 3.94 (d, *J* = 11.0 Hz, 1H), 3.89 (s, 3H), 3.71 – 3.61 (m, 4H), 3.34 (dt, *J* = 8.6, 3.6 Hz, 1H), 2.89 (t, *J* = 4.4 Hz, 1H), 2.74 (dd, *J* = 4.8, 2.4 Hz, 1H), 1.58 – 1.53 (m, 2H), 1.31 – 1.22 (m, 11H).

**$^{13}$C NMR** (101 MHz, $CDCl_3$) δ 192.7, 159.3, 158.1, 156.3, 143.7, 141.1, 131.3, 130.4, 129.9, 129.4, 129.1, 128.6, 127.8, 127.1, 124.9, 123.1, 114.0, 74.2, 68.7, 63.1, 55.4, 50.2, 44.8, 41.7, 32.8, 31.02, 30.2, 26.0, 25.7.

**HRMS** (ESI, m/z) Calcd. for $C_{29}H_{36}O_4Na^+$, $[M+Na]^+$: 471.2511, found: 471.2506.

**1-((11-Hydroxyundecyl)oxy)-[2,2'-binaphthalene]-4-carbaldehyde (1h)**

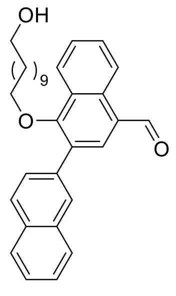


Prepared according to general procedure B (step 2), colorless oil, 75% yield, 351.0 mg.

**$^1$H NMR** (400 MHz, $CDCl_3$) δ 10.37 (s, 1H), 9.33 (d, *J* = 10.0 Hz, 1H), 8.39 (d, *J* = 8.4 Hz, 1H), 8.15 (s, 1H), 8.12 (s, 1H), 7.97 (d, *J* = 8.4 Hz, 1H), 7.95 – 7.90 (m, 2H), 7.85 (dd, *J* = 8.4, 1.6 Hz, 1H), 7.73 (ddd, *J* = 8.4, 6.8, 1.6 Hz, 1H), 7.66 (ddd, *J* = 8.4, 6.8, 1.2 Hz, 1H), 7.58 – 7.52 (m, 2H), 3.70 (t, *J* = 6.4 Hz, 2H), 3.64 (t, *J* = 6.6 Hz, 2H), 1.62 – 1.52 (m, 4H), 1.36 – 1.19 (m, 10H), 1.14 – 1.10 (m, 4H).

**$^{13}$C NMR** (101 MHz, $CDCl_3$) δ 192.7, 158.6, 141.2, 135.2, 133.5, 132.7, 131.6, 129.4, 128.8, 128.2,

128.2, 127.8, 127.6, 127.3, 127.2, 126.4, 126.4, 125.0, 123.2, 74.6, 63.1, 32.8, 30.2, 29.5, 29.4, 29.2, 25.9, 25.7.

**HRMS** (ESI, m/z) Calcd. for $C_{32}H_{36}O_3Na^+$, $[M+Na]^+$: 491.2562, found: 491.2557.

**4-((11-Hydroxyundecyl)oxy)-3-(thiophen-3-yl)-1-naphthaldehyde (1i)**

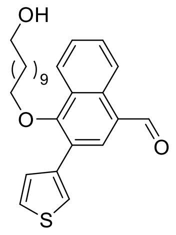


Prepared according to general procedure B (step 2), colorless oil, 61% yield, 258.8 mg.

**$^1$H NMR** (400 MHz, $CDCl_3$) δ 10.34 (s, 1H), 9.27 (d, *J* = 7.2 Hz, 1H), 8.31 (d, *J* = 10.0 Hz, 1H), 8.11 (s, 1H), 7.73 – 7.61 (m, 3H), 7.56 (d, *J* = 1.2 Hz, 1H), 7.46 (dd, *J* = 5.2, 3.2 Hz, 1H), 3.77 (t, *J* = 6.6 Hz, 2H), 3.64 (t, *J* = 6.6 Hz, 2H), 1.80 – 1.71 (m, 2H), 1.60 – 1.53 (m, 2H), 1.44 – 1.39 (m, 2H), 1.36 – 1.26 (m, 12H).

**$^{13}$C NMR** (101 MHz, $CDCl_3$) δ 192.6, 158.0, 140.0, 131.4, 129.5, 129.2, 128.3, 127.7, 127.2, 125.7, 125.0, 124.2, 123.8, 123.1, 74.4, 63.1, 32.8, 30.3, 29.6, 29.5, 29.4, 29.4, 26.0, 25.8.

**HRMS** (ESI, m/z) Calcd. for $C_{26}H_{32}O_3SNa^+$, $[M+Na]^+$: 447.1970, found: 447.1964.

**3-(Furan-3-yl)-4-((11-hydroxyundecyl)oxy)-1-naphthaldehyde (1j)**

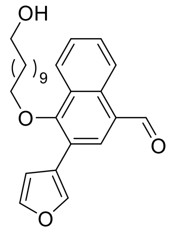


Prepared according to general procedure B (step 2), colorless oil, 63% yield, 257.2 mg.

**$^1$H NMR** (400 MHz, $CDCl_3$) δ 10.36 (s, 1H), 9.25 (d, *J* = 8.2 Hz, 1H), 8.28 – 8.22 (m, 1H), 8.10 – 8.04 (m, 2H), 7.65 (dddd, *J* = 21.6, 8.2, 6.8, 1.4 Hz, 2H), 7.58 (t, *J* = 1.6 Hz, 1H), 6.95 (dd, *J* = 1.6, 08 Hz, 1H), 3.90 (t, *J* = 6.6 Hz, 2H), 3.65 (t, *J* = 6.6 Hz, 2H), 1.93 – 1.86 (m, 2H), 1.59 – 1.48 (m, 6H), 1.35 – 1.30 (m, 10H).

**$^{13}$C NMR** (101 MHz, $CDCl_3$) δ 192.5, 157.7, 143.3, 141.5, 138.4, 131.4, 129.3, 129.0, 128.0, 127.2, 125.0, 122.9, 121.5, 121.0, 110.0, 74.1, 63.1, 32.8, 30.4, 29.7, 29.6, 29.5, 29.4, 29.3, 26.0, 25.8.

**HRMS** (ESI, m/z) Calcd. for $C_{26}H_{32}O_4Na^+$, [M+Na] $^+$: 431.2198, found: 431.2193.

**3-(Benzo[b]thiophen-3-yl)-4-((11-hydroxyundecyl)oxy)-1-naphthaldehyde (1k)**

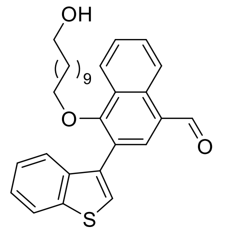

Prepared according to general procedure B (step 2), colorless oil, 54% yield, 256.0 mg.

**$^1$H NMR** (400 MHz, $CDCl_3$) δ 10.28 (s, 1H), 9.35 (d, *J* = 8.4 Hz, 1H), 8.40 (d, *J* = 8.4 Hz, 1H), 8.02 (s, 1H), 7.96 (s, 1H), 7.77 – 7.63 (m, 3H), 7.61 (s, 1H), 7.41 (tt, *J* = 7.2, 5.6 Hz, 2H), 3.64 (td, *J* = 6.4, 2.4 Hz, 4H), 1.61 – 1.53 (m, 2H), 1.52 – 1.44 (m, 2H), 1.37 – 1.22 (m, 6H), 1.18 – 0.99 (m, 8H).

**$^{13}$C NMR** (101 MHz, $CDCl_3$) δ 192.5, 159.2, 141.5, 140.0, 138.2, 133.3, 131.7, 129.5, 129.2, 127.2, 125.5, 125.1, 124.7, 124.5, 123.3, 123.2, 122.8, 122.5, 74.4, 63.0, 32.8, 30.0, 29.5, 29.4, 29.4, 29.1, 25.8, 25.7.

**HRMS** (ESI, m/z) Calcd. for $C_{30}H_{34}O_3SNa^+$, [M+Na] $^+$: 497.2126, found: 497.2121.

**3-(Benzofuran-3-yl)-4-((11-hydroxyundecyl)oxy)-1-naphthaldehyde (1l)**

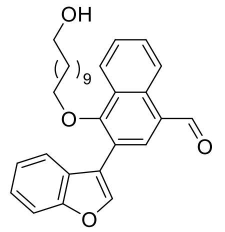

Prepared according to general procedure B (step 2), colorless oil, 52% yield, 238.2 mg.

**$^1$H NMR** (400 MHz, Acetonitrile-*d*$_3$) δ 10.13 (s, 1H), 9.10 (d, *J* = 8.4 Hz, 1H), 8.17 (d, *J* = 8.6 Hz, 1H), 8.03 (s, 1H), 7.96 (s, 1H), 7.63 – 7.55 (m, 2H), 7.49 (d, *J* = 9.6 Hz, 2H), 7.27 (t, *J* = 7.2 Hz, 1H), 7.17 (t, *J* = 7.2 Hz, 1H), 3.55 (t, *J* = 6.4 Hz, 2H), 3.39 (t, *J* = 6.4 Hz, 2H), 1.43 – 1.32 (m, 4H), 1.19 – 0.88 (m, 14H).

**$^{13}$C NMR** (101 MHz, Acetonitrile-*d*$_3$) δ 193.6, 159.3, 155.9, 144.7, 140.8, 132.0, 130.0, 129.7, 128.3, 127.9, 127.1, 125.6, 125.4, 123.7, 122.0, 120.4, 118.1, 74.9, 62.4, 33.3, 30.4, 30.1, 29.9, 29.9, 29.8, 29.5, 26.4, 26.2.

**HRMS** (ESI, m/z) Calcd. for $C_{30}H_{34}O_4Na^+$, $[M+Na]^+$: 481.2355, found: 481.2349.

**4-((11-Hydroxyundecyl)oxy)-2-methyl-1-naphthaldehyde (1m)**

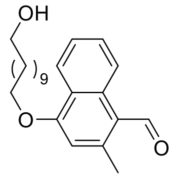


Prepared according to general procedure A, colorless oil, 55% yield, 195.9 mg.

**$^1$H NMR** (400 MHz, $CDCl_3$) δ 10.26 (s, 1H), 9.22 (d, *J* = 8.4 Hz, 1H), 8.16 (d, *J* = 8.4 Hz, 1H), 7.83 (s, 1H), 7.60 (dddd, *J* = 21.2, 8.4, 6.8, 1.6 Hz, 2H), 4.00 (t, *J* = 6.6 Hz, 2H), 3.64 (t, *J* = 6.6 Hz, 2H), 2.50 (s, 3H), 1.93 (dd, *J* = 14.2, 7.6 Hz, 2H), 1.63 – 1.52 (m, 6H), 1.37 – 1.30 (m, 10H).

**$^{13}$C NMR** (101 MHz, $CDCl_3$) δ 192.6, 158.7, 131.0, 128.7, 128.3, 127.3, 126.7, 125.5, 124.9, 122.3, 74.4, 62.9, 32.7, 30.3, 29.6, 29.5, 29.4, 29.3, 26.0, 25.6, 16.0.

**HRMS** (ESI, m/z) Calcd. for $C_{23}H_{32}O_3Na^+$, $[M+Na]^+$: 379.2249, found: 379.2244.

**2-Bromo-4-((11-hydroxyundecyl)oxy)-6-methoxybenzaldehyde (1n)**

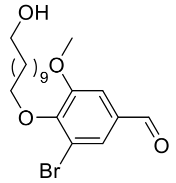


Prepared according to general procedure A with 5-Nitrovanillin and **5a**, colorless oil, 48% yield, 192.0 mg.

**$^1$H NMR** (400 MHz, $CDCl_3$) δ 9.83 (s, 1H), 7.65 (s, 1H), 7.37 (d, *J* = 1.6 Hz, 1H), 4.10 (t, *J* = 6.6 Hz, 2H), 3.91 (s, 3H), 3.63 (t, *J* = 6.6 Hz, 2H), 1.84 – 1.77 (m, 2H), 1.59 – 1.54 (m, 2H), 1.53– 1.47 (m, 2H), 1.34 – 1.27 (m, 12H).

**$^{13}$C NMR** (101 MHz, $CDCl_3$) δ 154.3, 151.4, 128.9, 73.9, 63.4, 56.2, 32.9, 30.2, 29.6, 29.5, 29.4, 26.0, 25.9, 25.8, 18.4.

**HRMS** (ESI, m/z) Calcd. for $C_{19}H_{29}BrO_4Na^+$, $[M+Na]^+$: 423.1147, found: 423.1141.

**4-((11-Hydroxyundecyl)oxy)-2-methoxy-6-nitrobenzaldehyde (1o)**

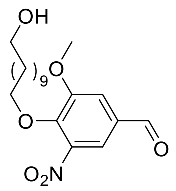

Prepared according to general procedure A with 5-Bromovanillin and **5a**, colorless oil, 61% yield, 224.0 mg.

**$^1$H NMR** (400 MHz, $CDCl_3$) δ 9.89 (s, 1H), 7.81 (s, 1H), 7.59 (s, 1H), 4.23 (t, $J$ = 6.6 Hz, 2H), 3.97 (s, 3H), 3.62 (t, $J$ = 6.6 Hz, 4H), 1.81 – 1.75 (m, 2H), 1.59 – 1.52 (m, 4H), 1.45 – 1.38 (m, 4H), 1.30 – 1.27 (m, 6H).

**$^{13}$C NMR** (101 MHz, $CDCl_3$) δ 154.8, 147.5, 145.0, 131.1, 75.4, 63.1, 56.7, 32.8, 29.9, 29.6, 29.5, 29.4, 29.3, 25.7, 25.6, 21.8.

**HRMS** (ESI, m/z) Calcd. for $C_{19}H_{29}NO_6Na^+$, $[M+Na]^+$: 390.1893, found: 390.1889.

**3-Bromo-4-((10-hydroxydecyl)oxy)-1-naphthaldehyde (1p)**

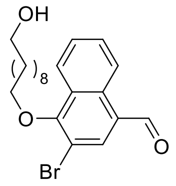

Prepared according to general procedure A, colorless oil, 48% yield, 194.9 mg.

**$^1$H NMR** (400 MHz, $CDCl_3$) δ 10.25 (s, 1H), 9.22 (d, $J$ = 9.6 Hz, 1H), 8.20 (d, $J$ = 8.4 Hz, 1H), 8.10 (s, 1H), 7.71 (ddd, $J$ = 8.4, 6.8, 1.6 Hz, 1H), 7.63 (ddd, $J$ = 8.4, 6.8, 1.6 Hz, 1H), 4.18 (t, $J$ = 6.4 Hz, 2H), 3.65 (t, $J$ = 6.4 Hz, 2H), 2.02 – 1.95 (m, 2H), 1.62 – 1.55 (m, 4H), 1.42 – 1.33 (m, 10H).

**$^{13}$C NMR** (101 MHz, $CDCl_3$) δ 191.3, 158.4, 141.3, 131.3, 129.9, 129.5, 128.8, 127.7, 125.3, 122.7, 111.6, 75.2, 63.1, 32.8, 30.3, 29.6, 29.5, 29.5, 29.4, 26.0, 25.8.

**HRMS** (ESI, m/z) Calcd. for $C_{21}H_{27}BrO_3Na^+$, $[M+Na]^+$: 429.1041, found: 429.1038.

**3-Bromo-4-((9-hydroxynonyl)oxy)-1-naphthaldehyde (1q)**

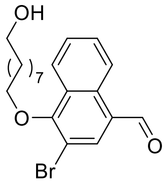

Prepared according to general procedure A, colorless oil, 42% yield, 164.7 mg.

**$^1$H NMR** (400 MHz, $CDCl_3$) δ 10.26 (s, 1H), 9.22 (d, $J$ = 9.6 Hz, 1H), 8.21 (d, $J$ = 7.6 Hz, 1H), 8.10 (s, 1H), 7.71 (ddd, $J$ = 8.4, 6.8, 1.2 Hz, 1H), 7.63 (ddd, $J$ = 8.4, 6.8, 1.2 Hz, 1H), 4.18 (t, $J$ = 6.6 Hz, 2H), 3.64 (d, $J$ = 6.6 Hz, 2H), 1.98 (dt, $J$ = 9.2, 6.8 Hz, 2H), 1.64 – 1.57 (m, 5H), 1.42 – 1.35 (m, 7H).

**$^{13}$C NMR** (101 MHz, $CDCl_3$) δ 191.3, 161.6, 146.6, 132.1, 129.7, 129.3, 129.0, 127.6, 125.4, 122.8, 85.5, 75.4, 63.1, 32.8, 30.3, 29.6, 29.5, 29.5, 26.1, 25.8.

**HRMS** (ESI, m/z) Calcd. for $C_{20}H_{25}BrO_3Na^+$, $[M+Na]^+$: 415.0885, found: 415.0882.

**3-Bromo-4-((8-hydroxyoctyl)oxy)-1-naphthaldehyde (1r)**

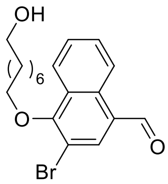

Prepared according to general procedure A, colorless oil, 50% yield, 189.1 mg.

**$^1$H NMR** (400 MHz, $CDCl_3$) δ 10.26 (s, 1H), 9.23 (d, $J$ = 8.4 Hz, 1H), 8.21 (d, $J$ = 8.4 Hz, 1H), 8.12 (s, 1H), 7.71 (ddd, $J$ = 8.4, 6.8, 1.6 Hz, 1H), 7.64 (ddd, $J$ = 8.4, 6.8, 1.6 Hz, 1H), 4.18 (t, $J$ = 6.8 Hz, 2H), 3.64 (d, $J$ = 6.8 Hz, 2H), 2.01 – 1.96 (m, 2H), 1.38 – 1.33 (m, 8H).

**$^{13}$C NMR** (101 MHz, $CDCl_3$) δ 191.2, 158.2, 141.0, 131.1, 129.6, 129.4, 128.6, 127.6, 125.0, 122.5, 111.5, 75.0, 62.6, 32.7, 30.2, 29.4, 29.4, 25.9, 25.7.

**HRMS** (ESI, m/z) Calcd. for $C_{19}H_{23}BrO_3Na^+$, $[M+Na]^+$: 401.0728, found: 401.0725.

**3-Bromo-4-((12-hydroxydodecyl)oxy)-1-naphthaldehyde (1s)**

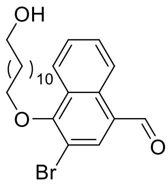

Prepared according to general procedure A, colorless oil, 43% yield, 186.7 mg.

**$^{1}$H NMR** (400 MHz, $CDCl_3$) δ 10.26 (s, 1H), 9.22 (d, *J* = 8.0 Hz, 1H), 8.22 (d, *J* = 8.4 Hz, 1H), 7.96 (s, 1H), 7.66 (dddd, *J* = 24.6, 8.4, 6.8, 1.6 Hz, 2H), 4.22 (t, *J* = 6.6 Hz, 2H), 3.64 (t, *J* = 6.6 Hz, 2H), 1.96 (dt, *J* = 14.8, 6.8 Hz, 2H), 1.59 – 1.55 (m, 3H), 1.40 – 1.28 (m, 17H).

**$^{13}$C NMR** (101 MHz, $CDCl_3$) δ 191.4, 157.0, 138.6, 130.8, 129.9, 129.4, 128.4, 127.7, 125.1, 122.7, 122.3, 75.1, 63.1, 32.8, 30.3, 29.6, 29.6, 29.5, 26.0, 25.8.

**HRMS** (ESI, m/z) Calcd. for $C_{23}H_{31}BrO_3Na^+$, [M+Na]$^+$: 457.1354, found: 457.1348.

**3-Bromo-4-((13-hydroxytridecyl)oxy)-1-naphthaldehyde (1t)**

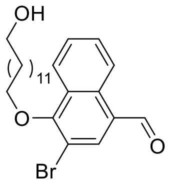


Prepared according to general procedure A, colorless oil, 42% yield, 188.2 mg.

**$^{1}$H NMR** (400 MHz, $CDCl_3$) δ 10.26 (s, 1H), 9.23 (d, *J* = 9.6 Hz, 1H), 8.21 (d, *J* = 10.0 Hz, 1H), 8.10 (s, 1H), 7.71 (ddd, *J* = 8.4, 6.8, 1.4 Hz, 1H), 7.63 (ddd, *J* = 8.4, 6.9, 1.3 Hz, 1H), 4.18 (t, *J* = 6.6 Hz, 2H), 3.64 (t, *J* = 6.6 Hz, 2H), 1.99 (dq, *J* = 8.4, 6.8 Hz, 2H), 1.62 – 1.53 (m, 4H), 1.30 (s, 14H).

**$^{13}$C NMR** (101 MHz, $CDCl_3$) δ 166.1, 159.7, 140.2, 132.7, 129.2, 128.1, 126.5, 126.3, 124.2, 122.8, 85.1, 74.1, 65.2, 30.0, 28.8, 28.6, 28.5, 28.4, 28.1, 27.3, 26.2, 25.7.

**HRMS** (ESI, m/z) Calcd. for $C_{24}H_{33}BrO_3Na^+$, [M+Na]$^+$: 471.1511, found: 471.1506

**3-Bromo-4-(3-((3-((3-hydroxypropyl)thio)propyl)thio)propoxy)-1-naphthaldehyde (1u)**

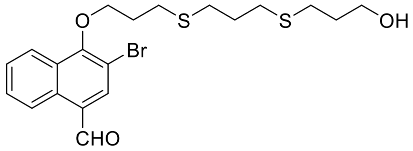


Prepared according to general procedure A, colorless oil, 58% yield, 264.5 mg.

**$^{1}$H NMR** (400 MHz, $CDCl_3$) δ 10.26 (s, 1H), 9.22 (d, *J* = 8.4 Hz, 1H), 8.20 (d, *J* = 8.0 Hz, 1H), 8.10 (s, 1H), 7.72 (ddd, *J* = 8.4, 6.8, 1.4 Hz, 1H), 7.64 (ddd, *J* = 8.2, 6.8, 1.4 Hz, 1H), 4.28 (t, *J* = 6.0 Hz, 2H), 3.76 (t, *J* = 6.0 Hz, 2H), 2.90 (d, *J* = 14.4 Hz, 2H), 2.73 – 2.63 (m, 6H), 2.30 – 2.23 (m, 2H), 1.96 – 1.89 (m, 2H), 1.88 – 1.83 (m, 2H).

**$^{13}$C NMR** (101 MHz, $CDCl_3$) δ 191.3, 157.9, 141.1, 131.3, 129.7, 129.6, 129.0, 127.9, 125.3, 122.6, 111.7, 73.2, 61.8, 32.0, 31.0, 30.9, 30.2, 29.2, 28.8, 28.6.

**HRMS** (ESI, m/z) Calcd. for $C_{20}H_{25}BrO_3S_2Na^+$, $[M+Na]^+$: 479.0326, found: 479.0320.

**3-Bromo-4-((12-hydroxydodeca-5,7-diyn-1-yl)oxy)-1-naphthaldehyde (1v)**

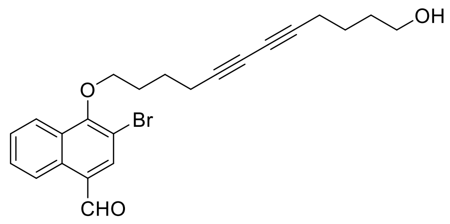


Prepared according to general procedure A, colorless oil, 62% yield, 264.1 mg.

**$^1$H NMR** (400 MHz, $CDCl_3$) δ 10.25 (s, 1H), 9.22 (d, *J* = 8.2 Hz, 1H), 8.18 (d, *J* = 8.2 Hz, 1H), 8.09 (s, 1H), 7.71 (ddd, *J* = 8.4, 6.8, 1.2 Hz, 1H), 7.64 (ddd, *J* = 8.4, 6.8, 1.2 Hz, 1H), 4.19 (t, *J* = 6.2 Hz, 2H), 3.67 (t, *J* = 6.2 Hz, 2H), 2.44 (t, *J* = 6.8 Hz, 2H), 2.32 (t, *J* = 6.8 Hz, 2H), 2.13 – 2.06 (m, 2H), 1.94 – 1.87 (m, 2H), 1.70 – 1.62 (m, 4H).

**$^{13}$C NMR** (101 MHz, $CDCl_3$) δ 191.3, 157.9, 141.1, 131.3, 129.7, 129.6, 129.0, 127.9, 125.3, 122.6, 111.7, 73.2, 61.8, 32.0, 31.0, 30.9, 30.2, 29.2, 28.8, 28.6.

**HRMS** (ESI, m/z) Calcd. for $C_{23}H_{23}BrO_3Na^+$, $[M+Na]^+$: 449.0723, found: 449.0717.

**3-Bromo-4-((5-((5-hydroxypentyl)oxy)pentyl)oxy)-1-naphthaldehyde (1w)**

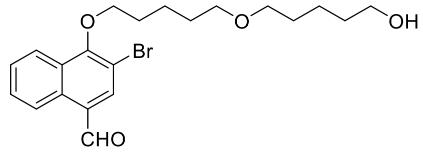


Prepared according to general procedure A, colorless oil, 53% yield, 223.7 mg.

**$^1$H NMR** (400 MHz, $CDCl_3$) δ 10.23 (s, 1H), 9.20 (d, *J* = 8.4 Hz, 1H), 8.18 (d, *J* = 8.4 Hz, 1H), 8.07 (s, 1H), 7.70 (ddd, *J* = 8.4, 6.8, 1.4 Hz, 1H), 7.62 (ddd, *J* = 8.2, 6.8, 1.4 Hz, 1H), 4.17 (t, *J* = 6.5 Hz, 2H), 3.64 (t, *J* = 6.5 Hz, 2H), 3.46 (dt, *J* = 14.9, 6.2 Hz, 4H), 2.01 (p, *J* = 6.8 Hz, 2H), 1.76 – 1.66 (m, 4H), 1.64 – 1.58 (m, 4H), 1.49 – 1.41 (m, 2H).

**$^{13}$C NMR** (101 MHz, $CDCl_3$) δ 158.3, 141.2, 131.2, 129.8, 129.5, 128.7, 127.7, 125.2, 122.6, 111.6, 74.9, 70.9, 70.7, 62.7, 32.5, 30.1, 29.5, 29.4, 22.7, 22.5.

**HRMS** (ESI, m/z) Calcd. for $C_{21}H_{27}BrO_4Na^+$, $[M+Na]^+$: 445.0990, found: 445.1842.

**1-((*R*)-1-Methoxy-1,1-diphenyl-3-(piperidin-1-yl)propan-2-yl)-3-((1S)-(6-methoxyquinolin-4-yl)((2*S*,4*S*,5*R*)-5-vinylquinuclidin-2-yl)methyl)thiourea (**HBD **3)**

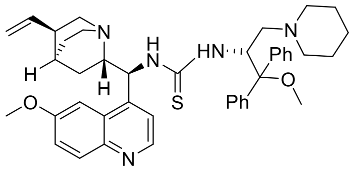


White solid, 51% yield, 562 mg; m.p. 265-266 °C.

$[\alpha]^{25}_{D}$ = -181.2 (*c* = 0.1 in $CHCl_3$);

**$^1$H NMR** (400 MHz, $CDCl_3$) δ 9.51 (s, 1H), 8.72 (d, *J* = 4.4 Hz, 1H), 8.22 (s, 1H), 7.98 (d, *J* = 9.2 Hz, 1H), 7.52 – 7.27 (m, 15H), 6.04 (s, 1H), 5.84 (s, 2H), 5.06 – 4.97 (m, 2H), 4.68 (s, 1H), 4.03 (s, 3H), 3.63 (s, 1H), 3.34 – 3.21 (m, 2H), 2.99 (s, 3H), 2.88 – 2.74 (m, 2H), 2.63 (d, *J* = 12.4 Hz, 1H), 2.36 (s, 3H), 1.65 – 1.52 (m, 3H), 1.09 (d, *J* = 48.2 Hz, 3H).

**$^{13}$C NMR** (101 MHz, $CDCl_3$) δ 153.4, 134.6, 131.2, 131.0, 129.9, 128.9, 128.9, 127.8, 126.5, 126.4, 124.4, 123.6, 123.5, 117.8, 114.1, 109.5, 65.6, 52.7, 35.0, 33.8, 31.9, 31.5, 31.4, 30.6, 30.3, 30.1, 29.7, 29.5, 29.4, 29.2, 29.0, 22.7, 19.2.

**HRMS** (ESI, m/z) Calcd. for $C_{42}H_{52}N_5O_2S^+$, $[M+H]^+$: 690.3763, found: 690.3812.

## (2) Characterization of the planar chiral products

**($R_p$)-12-Bromo-2,14-dioxa-1(1,4)-naphthalenacyclopentadecaphan-15-one (2a)**

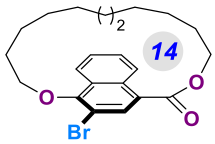

Prepared according to general procedure C, light yellow oil, 80% yield, 16.7 mg.

$[\alpha]^{25}_D$ = -209.2 ($c$ = 0.1 in $CHCl_3$).

**$^1$H NMR** (400 MHz, $CDCl_3$) δ 8.90 (d, $J$ = 8.6 Hz, 1H), 8.36 (s, 1H), 8.26 (d, $J$ = 10.0 Hz, 1H), 7.65 – 7.59 (m, 1H), 7.59 – 7.53 (m, 1H), 4.90 – 4.82 (m, 1H), 4.73 – 4.66 (m, 1H), 4.44 – 4.33 (m, 2H), 3.72 (q, $J$ = 7.0 Hz, 2H), 1.80 – 1.75 (m, 2H), 1.72 – 1.66 (m, 1H), 1.21 – 1.09 (m, 3H), 1.06 – 1.01 (m, 1H), 0.95 – 0.87 (m, 2H), 0.78 – 0.70 (m, 2H), 0.70 – 0.58 (m, 4H), 0.54 – 0.47 (m, 1H).

**$^{13}$C NMR** ((101 MHz, $CDCl_3$) δ 166.3, 135.0, 130.4, 128.1, 126.6, 126.3, 122.9, 110.7, 65.4, 29.7, 28.9, 28.6, 28.4, 28.2, 27.3, 25.9.

**HRMS** (ESI, m/z) Calcd. for $C_{22}H_{27}BrO_3Na^+$, $[M+Na]^+$: 441.1041, found: 441.1036.

**HPLC analysis**: 96:4 e.r. (IC column, 25 °C, hexane / *i*PrOH = 95 / 5, 0.8 mL / min, λ = 254 nm), Rt (minor) = 9.4 min, Rt (major) = 10.1 min.

**($R_p$)-12-Chloro-2,14-dioxa-1(1,4)-naphthalenacyclopentadecaphan-15-one (2b)**

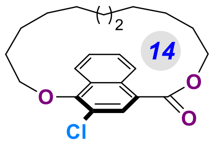

Prepared according to general procedure C, light yellow oil, 61% yield, 11.4 mg.

$[\alpha]^{25}_D$ = -192.8 ($c$ = 0.1 in $CHCl_3$).

**$^1$H NMR** (400 MHz, $CDCl_3$) δ 8.93 – 8.88 (m, 1H), 8.30 – 8.26 (m, 1H), 8.20 (s, 1H), 7.63 – 7.54 (m, 2H), 4.92 – 4.86 (m, 1H), 4.72 – 4.66 (m, 1H), 4.44 – 4.33 (m, 2H), 3.71 (t, $J$ = 7.0 Hz, 1H), 1.85 – 1.78 (m, 3H), 1.20 – 1.09 (m, 3H), 0.97 – 0.87 (m, 3H), 0.85 – 0.72 (m, 3H), 0.68 – 0.60 (m, 3H), 0.55 – 0.44 (m, 2H).

**$^{13}$C NMR** (101 MHz, $CDCl_3$) δ 166.5, 154.4, 132.5, 131.4, 130.4, 128.0, 126.6, 126.2, 123.8, 122.9, 121.2, 72.7, 65.5, 58.5, 29.5, 28.9, 28.4, 28.3, 27.2, 26.1, 25.8, 18.5.

**HRMS** (ESI, m/z) Calcd. for $C_{22}H_{27}ClO_3Na^+$, $[M+Na]^+$: 397.1546, found: 397.1541.

**HPLC analysis**: 95:5 e.r. (IC column, 25 °C, hexane / *i*PrOH = 95 / 5, 0.8 mL / min, λ = 254 nm), Rt (minor) = 8.1 min, Rt (major) = 9.2 min.

**(*R*p)-12-Iodo-2,14-dioxa-1(1,4)-naphthalenacyclopentadecaphan-15-one (2c)**

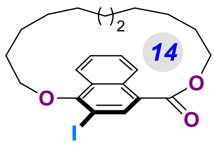

Prepared according to general procedure C, light yellow oil, 75% yield, 17.5 mg.

$[\alpha]^{25}_{D}$ = -158.2 (*c* = 0.1 in $CHCl_3$).

**$^1$H NMR** (400 MHz, $CDCl_3$) δ 8.89 (d, *J* = 7.8 Hz, 1H), 8.55 (s, 1H), 8.20 (d, *J* = 6.6 Hz, 1H), 7.64 – 7.59 (m, 1H), 7.56 – 7.52 (m, 1H), 4.79 (ddd, *J* = 11.9, 7.0, 3.3 Hz, 1H), 4.72 – 4.66 (m, 1H), 4.44 – 4.31 (m, 2H), 3.72 (q, *J* = 7.0 Hz, 2H), 1.82 – 1.76 (m, 3H), 1.39 – 1.34 (m, 2H), 1.08 – 1.02 (m, 1H), 0.99 – 0.86 (m, 3H), 0.72 – 0.61 (m, 6H), 0.58 – 0.50 (m, 1H).

**$^{13}$C NMR** (101 MHz, $CDCl_3$) δ 166.1, 159.7, 140.2, 132.7, 129.2, 128.1, 126.5, 126.3, 124.2, 122.8, 85.1, 74.1, 65.2, 30.0, 28.8, 28.6, 28.5, 28.4, 28.1, 27.3, 26.2, 25.7.

**HRMS** (ESI, m/z) Calcd. for $C_{22}H_{27}IO_3Na^+$, $[M+Na]^+$: 489.0903, found: 489.0897.

**HPLC analysis**: 97:3 e.r. (IG column, 25 °C, hexane / *i*PrOH = 95 / 5, 0.8 mL / min, λ = 254 nm), Rt (major) = 12.4 min, Rt (minor) = 13.5 min.

**(*R*p)-12-Phenyl-2,14-dioxa-1(1,4)-naphthalenacyclopentadecaphan-15-one (2d)**

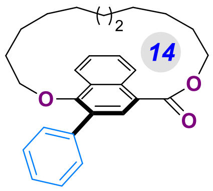

Prepared according to general procedure C at 70 °C, light yellow oil, 81% yield, 16.8 mg.

$[\alpha]^{25}_{D}$ = -74.6 (*c* = 0.1 in $CHCl_3$).

**$^1$H NMR** (400 MHz, $CDCl_3$) δ 8.98 (dd, *J* = 8.4, 1.2 Hz, 1H), 8.44 (dd, *J* = 8.4, 1.2 Hz, 1H), 8.36 (s, 1H), 7.74 – 7.70 (m, 2H), 7.64 – 7.55 (m, 2H), 7.50 (dd, *J* = 8.4, 6.8z Hz, 2H), 7.42 – 7.36 (m, 1H), 4.70 (ddd, *J* = 11.2, 6.8, 4.4 Hz, 1H), 4.37 (ddd, *J* = 10.8, 6.4, 4.3 Hz, 1H), 4.00 (ddd, *J* = 11.2, 6.8, 4.4 Hz, 1H), 3.89 (ddd, *J* = 11.6, 7.8, 3.6 Hz, 1H), 1.83 (td, *J* = 6.0, 3.6 Hz, 2H), 1.44 – 1.37 (m, 2H), 1.34 – 1.19 (m, 4H), 1.16 – 1.09 (m, 1H), 1.00 – 0.94 (m, 1H), 0.78 – 0.65 (m, 5H), 0.56

– 0.39 (m, 3H).

**$^{13}$C NMR** (101 MHz, $CDCl_3$) δ 167.4, 156.2, 138.6, 133.8, 132.0, 130.3, 129.1, 128.7, 127.9, 127.5, 127.1, 126.0, 125.8, 123.5, 122.4, 71.6, 65.3, 29.3, 29.0, 29.0, 28.5, 28.5, 28.0, 27.4, 26.4, 25.4.

**HRMS** (ESI, m/z) Calcd. for $C_{28}H_{32}O_3Na^+$, $[M+Na]^+$: 439.2249, found: 439.2244.

**HPLC analysis**: 93:7 e.r. (IC column, 25 °C, hexane / *i*PrOH = 95 / 5, 0.8 mL / min, λ = 254 nm), Rt (minor) = 7.5 min, Rt (major) = 9.8 min.

**($R_p$)-12-(4-Chlorophenyl)-2,14-dioxa-1(1,4)-naphthalenacyclopentadecaphan-15-one (2e)**

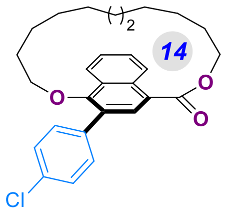


Prepared according to general procedure C, light yellow oil, 85% yield, 19.1 mg.

$[\alpha]^{25}_D$ = -185.3 (*c* = 0.1 in $CHCl_3$).

**$^1$H NMR** (400 MHz, $CDCl_3$) δ 8.96 (d, *J* = 8.0 Hz, 1H), 8.42 (d, *J* = 9.0 Hz, 1H), 8.30 (s, 1H), 7.68 – 7.55 (m, 4H), 7.50 – 7.46 (m, 2H), 4.68 (ddd, *J* = 11.2, 6.4, 4.4 Hz, 1H), 4.38 (ddd, *J* = 10.8, 6.4, 4.4 Hz, 1H), 4.03 (ddd, *J* = 12.0, 6.8, 4.0 Hz, 1H), 3.89 (ddd, *J* = 12.0, 7.8, 3.6 Hz, 1H), 1.86 – 1.78 (m, 2H), 1.44 – 1.37 (m, 2H), 1.28 – 1.19 (m, 4H), 1.13 – 1.05 (m, 1H), 1.00 – 0.91 (m, 1H), 0.76 – 0.62 (m, 5H), 0.55 – 0.36 (m, 3H).

**$^{13}$C NMR** ((101 MHz, $CDCl_3$) δ 167.3, 156.3, 137.0, 133.6, 133.1, 132.1, 130.5, 130.3, 129.0, 128.1, 126.2, 126.1, 125.9, 123.5, 122.8, 71.9, 65.3, 29.7, 29.4, 29.0, 28.5, 28.5, 28.1, 27.4, 26.4, 25.4.

**HRMS** (ESI, m/z) Calcd. for $C_{28}H_{31}ClO_3Na^+$, $[M+Na]^+$: 473.1859, found: 473.1853.

**HPLC analysis**: 91:9 e.r. (IC column, 25 °C, hexane / *i*PrOH = 95 / 5, 0.8 mL / min, λ = 254 nm), Rt (major) = 9.2 min, Rt (minor) = 11.3 min.

**($R_p$)-12-(P-tolyl)-2,14-dioxa-1(1,4)-naphthalenacyclopentadecaphan-15-one (2f)**

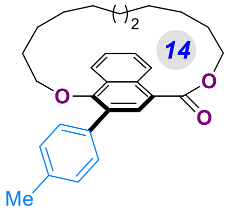


Prepared according to general procedure C at 70 ºC, light yellow oil, 88% yield, 18.9 mg.

$[\alpha]^{25}_{D}$ = -173.5 (*c* = 0.1 in $CHCl_3$).

**$^1$H NMR** (400 MHz, $CDCl_3$) δ 8.96 (d, *J* = 8.8 Hz, 1H), 8.43 (d, *J* = 8.8 Hz, 1H), 8.35 (s, 1H), 7.63 – 7.54 (m, 4H), 7.30 (d, *J* = 7.6 Hz, 2H), 4.69 (ddd, *J* = 11.2, 6.8, 4.4 Hz, 1H), 4.36 (ddd, *J* = 11.2, 6.8, 4.4 Hz, 1H), 4.02 – 3.96 (m, 1H), 3.91 (ddd, *J* = 11.2, 7.8, 4.0 Hz, 1H), 2.43 (s, 3H), 1.82 (dt, *J* = 12.8, 3.6 Hz, 2H), 1.44 – 1.37 (m, 2H), 1.28 – 1.21 (m, 4H), 1.13 – 1.06 (m, 1H), 1.00 – 0.93 (m, 1H), 0.77 – 0.65 (m, 5H), 0.57 – 0.47 (m, 2H), 0.44 – 0.34 (m, 1H).

**$^{13}$C NMR** (101 MHz, $CDCl_3$) δ 167.5, 156.1, 137.3, 135.7, 133.9, 131.9, 130.4, 129.5, 129.0, 127.7, 127.1, 126.0, 125.8, 123.4, 122.4, 71.4, 65.3, 29.4, 29.1, 29.0, 28.5, 28.5, 28.1, 27.5, 26.5, 25.4, 21.3.

**HRMS** (ESI, m/z) Calcd. for $C_{29}H_{34}O_3Na^+$, $[M+Na]^+$: 453.2406, found: 453.2400.

**HPLC analysis**: 93:7 e.r. (IC column, 25 ºC, hexane / *i*PrOH = 95 / 5, 0.8 mL / min, λ = 254 nm), Rt (minor) = 7.6 min, Rt (major) = 12.2 min.

**($R_p$)-12-(4-Methoxyphenyl)-2,14-dioxa-1(1,4)-naphthalenacyclopentadecaphan-15-one (2g)**

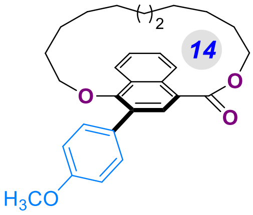


Prepared according to general procedure C at 70 ºC, light yellow oil, 82% yield, 18.3 mg.

$[\alpha]^{25}_{D}$ = -163.3 (*c* = 0.1 in $CHCl_3$).

**$^1$H NMR** (400 MHz, $CDCl_3$) δ 8.96 (d, *J* = 8.4 Hz, 1H), 8.42 (d, *J* = 8.4 Hz, 1H), 8.34 (s, 1H), 7.67 – 7.54 (m, 4H), 7.03 (d, *J* = 8.8 Hz, 2H), 4.69 (ddd, *J* = 11.2, 6.8, 4.4 Hz, 1H), 4.36 (ddd, *J* = 10.8, 6.4, 4.4 Hz, 1H), 4.03 – 3.91 (m, 2H), 3.89 (s, 3H), 1.86 – 1.79 (m, 2H), 1.43 – 1.38 (m, 2H), 1.29 – 1.23 (m, 4H), 1.14 – 1.07 (m, 1H), 1.00 – 0.94 (m, 1H), 0.77 – 0.65 (m, 5H), 0.56 – 0.47 (m, 2H),

0.44 – 0.36 (m, 1H).

**$^{13}$C NMR** (101 MHz, $CDCl_3$) δ 167.5, 159.2, 155.9, 133.8, 131.8, 131.0, 130.4, 130.3, 127.6, 126.9, 126.0, 125.8, 123.4, 122.4, 71.3, 65.2, 55.3, 29.7, 29.3, 29.1, 29.0, 28.5, 28.1, 27.5, 26.5, 25.4.

**HRMS** (ESI, m/z) Calcd. for $C_{29}H_{34}O_4Na^+$, $[M+Na]^+$: 469.2355, found: 469.2349.

**HPLC analysis**: 94:6 e.r. (IC column, 25 °C, hexane / *i*PrOH = 95 / 5, 0.8 mL / min, λ = 254 nm), Rt (minor) = 11.1 min, Rt (major) = 14.8 min.

**($R_p$)-12-(Naphthalen-2-yl)-2,14-dioxa-1(1,4)-naphthalenacyclopentadecaphan-15-one (2h)**

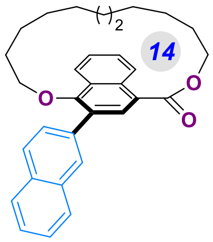


Prepared according to general procedure C, light yellow oil at 70 °C, 78% yield, 18.2 mg.

$[\alpha]^{25}_D$ = -135.5 ($c$ = 0.1 in $CHCl_3$).

**$^1$H NMR** (400 MHz, $CDCl_3$) δ 8.99 (d, $J$ = 10.0 Hz, 1H), 8.49 (s, 2H), 8.18 (s, 1H), 7.99 – 7.86 (m, 4H), 7.67 – 7.52 (m, 4H), 4.70 (ddd, $J$ = 11.1, 6.9, 4.2 Hz, 1H), 4.45 – 4.38 (m, 1H), 4.02 – 3.95 (m, 1H), 3.89 (ddd, $J$ = 11.6, 7.6, 3.6 Hz, 1H), 1.85 (qd, $J$ = 6.8, 3.2 Hz, 2H), 1.49 – 1.39 (m, 3H), 1.25 – 1.10 (m, 3H), 1.06 – 0.98 (m, 1H), 0.92 – 0.74 (m, 3H), 0.73 – 0.66 (m, 3H), 0.58 – 0.42 (m, 3H).

**$^{13}$C NMR** (101 MHz, $CDCl_3$) δ 167.5, 156.5, 136.2, 134.0, 133.7, 132.7, 132.0, 130.4, 128.3, 128.3, 127.9, 127.8, 127.8, 127.3, 126.9, 126.3, 126.3, 126.1, 125.9 123.5, 122.5, 29.7, 29.3, 29.1, 28.6, 28.5, 28.0, 27.5, 26.6, 25.4.

**HRMS** (ESI, m/z) Calcd. for $C_{32}H_{34}O_3Na^+$, $[M+Na]^+$: 489.2406, found: 489.2400.

**HPLC analysis**: 93:7 e.r. (IC column, 25 °C, hexane / *i*PrOH = 95 / 5, 0.8 mL / min, λ = 254 nm), Rt (minor) = 8.4 min, Rt (major) = 9.3 min.

**($R_p$)-12-(Thiophen-3-yl)-2,14-dioxa-1(1,4)-naphthalenacyclopentadecaphan-15-one (2i)**

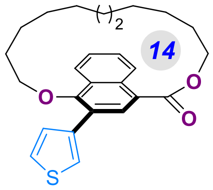


Prepared according to general procedure C, light yellow oil, 80% yield, 16.9 mg.

$[\alpha]^{25}_{D}$ = -108.5 ($c$ = 0.1 in $CHCl_3$).

**$^1$H NMR** (400 MHz, $CDCl_3$) δ 8.97 (dd, $J$ = 8.0, 1.2 Hz, 1H), 8.44 (s, 1H), 8.39 (dd, $J$ = 7.6, 1.2 Hz, 1H), 7.68 (dd, $J$ = 3.2, 1.2 Hz, 1H), 7.63 – 7.53 (m, 3H), 7.44 (dd, $J$ = 5.0, 3.2 Hz, 1H), 4.74 – 4.67 (m, 1H), 4.36 (ddd, $J$ = 10.8, 6.0, 4.4 Hz, 1H), 4.15 – 4.04 (m, 2H), 1.86 – 1.78 (m, 2H), 1.53 – 1.47 (m, 1H), 1.46 – 1.38 (m, 2H), 1.35 – 1.28 (m, 2H), 1.23 – 1.16 (m, 1H), 1.13 – 1.05 (m, 1H), 1.01 – 0.94 (m, 1H), 0.78 – 0.63 (m, 5H), 0.58 – 0.37 (m, 3H).

**$^{13}$C NMR** (101 MHz, $CDCl_3$) δ 167.3, 156.2, 138.7, 133.1, 132.0, 130.3, 128.3, 127.8, 126.1, 125.9, 125.7, 123.4, 123.2, 122.6, 122.4, 71.9, 29.4, 28.9, 28.5, 28.4, 28.1, 27.4, 26.2, 25.6.

**HRMS** (ESI, m/z) Calcd. for $C_{26}H_{30}O_3SNa^+$, $[M+Na]^+$: 445.1813, found: 445.1808.

**HPLC analysis**: 95:5 e.r. (IC column, 25 °C, hexane / $i$PrOH = 95 / 5, 0.8 mL / min, λ = 254 nm), Rt (minor) = 8.5 min, Rt (major) = 17.1 min.

**($R_p$)-12-(Furan-3-yl)-2,14-dioxa-1(1,4)-naphthalenacyclopentadecaphan-15-one (2j)**

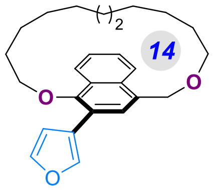


Prepared according to general procedure C at 70 °C, light yellow oil, 76% yield, 15.4 mg.

$[\alpha]^{25}_{D}$ = -131.3 ($c$ = 0.1 in $CHCl_3$).

**$^1$H NMR** (400 MHz, $CDCl_3$) δ 8.93 (d, $J$ = 10.2 Hz, 1H), 8.39 (s, 1H), 8.30 (d, $J$ = 6.0 Hz, 1H), 8.05 (s, 1H), 7.61 – 7.52 (m, 3H), 6.95 (s, 1H), 4.73 – 4.66 (m, 1H), 4.37 (dq, $J$ = 9.6, 5.2 Hz, 2H), 4.27 (dt, $J$ = 11.6, 5.2 Hz, 1H), 1.81 (dd, $J$ = 12.0, 5.6 Hz, 2H), 1.54 – 1.38 (m, 5H), 1.06 (q, $J$ = 6.4 Hz, 1H), 1.01 – 0.94 (m, 1H), 0.77 – 0.60 (m, 6H), 0.58 – 0.43 (m, 3H).

**$^{13}$C NMR** (101 MHz, $CDCl_3$) δ 167.4, 156.2, 138.6, 133.8, 132.0, 130.3, 129.1, 128.7, 127.8, 127.1, 126.0, 125.4, 123.4, 122.3, 71.5, 65.2, 29.3, 29.0, 28.9, 28.5, 28.4, 28.0, 27.4, 26.4, 25.3.

**HRMS** (ESI, m/z) Calcd. for $C_{26}H_{30}O_4Na^+$, $[M+Na]^+$: 429.2042, found: 429.2036.

**HPLC analysis**: 96:4 e.r. (IC column, 25 °C, hexane / *i*PrOH = 95 / 5, 0.8 mL / min, λ = 254 nm), Rt (minor) = 9.0 min, Rt (major) = 19.8 min.

**($R_p$)-12-(Benzo[b]thiophen-3-yl)-2,14-dioxa-1(1,4)-naphthalenacyclopentadecaphan-15-one (2k)**

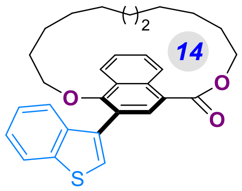


Prepared according to general procedure C, light yellow oil, 85% yield, 20.1 mg.

$[\alpha]^{25}_D$ = -172.3 (*c* = 0.1 in $CHCl_3$).

**$^1$H NMR** (400 MHz, $CDCl_3$) δ 8.99 (d, *J* = 9.2 Hz, 1H), 8.50 (d, *J* = 10.0 Hz, 1H), 8.40 (s, 1H), 7.98 – 7.93 (m, 1H), 7.87 – 7.81 (m, 1H), 7.69 – 7.58 (m, 3H), 7.45 – 7.39 (m, 2H), 4.75 – 4.66 (m, 1H), 4.43 – 4.35 (m, 1H), 3.91 – 3.84 (m, 1H), 3.79 (ddd, *J* = 12.0, 7.8, 3.6 Hz, 1H), 1.83 (p, *J* = 5.6 Hz, 2H), 1.49 – 1.21 (m, 6H), 1.16 – 1.08 (m, 1H), 1.05 – 0.95 (m, 1H), 0.82 – 0.73 (m, 5H), 0.62 – 0.40 (m, 3H).

**$^{13}$C NMR** (101 MHz, $CDCl_3$) δ 167.4, 156.6, 140.2, 137.5, 134.9, 134.2, 132.2, 130.3, 128.0, 126.2, 125.9, 124.8, 124.7, 124.4, 123.8, 123.5, 122.8, 122.5, 121.0, 71.2, 65.5, 29.4, 29.1, 29.0, 28.6, 28.5, 28.1, 27.4, 26.6, 25.4.

**HRMS** (ESI, m/z) Calcd. for $C_{30}H_{32}O_3S\ Na^+$, $[M+Na]^+$: 495.1970, found: 495.1964.

**HPLC analysis**: 95:5 e.r. (IC column, 25 °C, hexane / *i*PrOH = 95 / 5, 0.8 mL / min, λ = 254 nm), Rt (minor) = 8.9 min, Rt (major) = 10.0 min.

**($R_p$)-12-(Benzofuran-3-yl)-2,14-dioxa-1(1,4)-naphthalenacyclopentadecaphan-15-one (2l)**

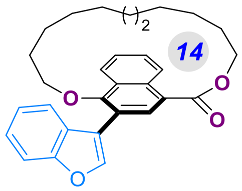


Prepared according to general procedure C, light yellow oil, 84% yield, 19.2 mg.

$[\alpha]^{25}_D$ = -165.3 (*c* = 0.1 in $CHCl_3$).

**$^1$H NMR** (400 MHz, $CDCl_3$) δ 8.98 (d, *J* = 8.8 Hz, 1H), 8.48 (s, 2H), 8.02 (s, 1H), 7.85 (d, *J* = 7.2 Hz, 1H), 7.67 – 7.57 (m, 3H), 7.42– 7.30 (m, 2H), 4.71 (ddd, *J* = 11.2, 6.4, 4.4 Hz, 1H), 4.43 – 4.36 (m, 1H), 4.11 (td, J = 8.0, 4.0 Hz, 1H), 4.04 (ddd, *J* = 12.1, 6.4, 4.0 Hz, 1H), 1.87 – 1.80 (m, 2H), 1.47 – 1.41 (m, 2H), 1.28 – 1.19 (m, 4H), 1.15 – 1.09 (m, 1H), 1.03– 0.96 (m, 1H), 0.77 – 0.65 (m, 5H), 0.57 – 0.41 (m, 3H).

**$^{13}$C NMR** (101 MHz, $CDCl_3$) δ 167.3, 156.6, 155.6, 143.0, 133.1, 132.1, 130.3, 127.9, 126.5, 126.2, 126.0, 124.8, 123.4, 123.1, 122.8, 121.7, 119.3, 118.0, 111.7, 71.7, 65.3, 31.5, 29.5, 28.9, 28.6, 28.5, 28.2, 27.5, 26.3, 25.6.

**HRMS** (ESI, m/z) Calcd. for $C_{30}H_{32}O_4Na^+$, $[M+Na]^+$: 479.2198, found: 479.2193.

**HPLC analysis**: 94:6 e.r. (IC column, 25 ºC, hexane / *i*PrOH = 95 / 5, 0.8 mL / min, λ = 254 nm), Rt (minor) = 7.2 min, Rt (major) = 8.4 min.

**(*R*$_p$)-13-Methyl-2,14-dioxa-1(1,4)-naphthalenacyclopentadecaphan-15-one (2m)**

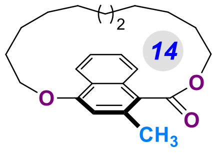


Prepared according to general procedure C, light yellow oil, 78% yield, 13.8 mg.

$[\alpha]^{25}_D$ = -122.4 (*c* = 0.1 in $CHCl_3$).

**$^1$H NMR** (400 MHz, $CDCl_3$) δ 8.86 (d, *J* = 8.6 Hz, 1H), 8.20 (d, *J* = 8.6 Hz, 1H), 8.09 (s, 1H), 7.55 – 7.48 (m, 2H), 4.66 (dt, *J* = 11.2, 5.6 Hz, 1H), 4.48 – 4.42 (m, 1H), 4.40 – 4.34 (m, 2H), 2.51 (s, 3H), 1.80 – 1.74 (m, 2H), 1.58 – 1.49 (m, 2H), 1.43 – 1.35 (m, 2H), 1.19 – 1.12 (m, 2H), 1.07 – 0.86 (m, 3H), 0.85 – 0.79 (m, 1H), 0.73 – 0.64 (m, 3H), 0.63 – 0.55 (m, 3H).

**$^{13}$C NMR** (101 MHz, $CDCl_3$) δ 167.7, 156.6, 134.4, 131.7, 129.0, 126.8, 126.2, 125.6, 124.7, 122.8, 122.5, 72.6, 65.0, 29.8, 28.8, 28.5, 28.4, 28.2, 27.4, 25.9, 25.8, 16.9.

**HRMS** (ESI, m/z) Calcd. for $C_{23}H_{30}O_3Na^+$, $[M+Na]^+$: 377.2093, found: 377.2087.

**HPLC analysis**: 95:5 e.r. (IC column, 25 ºC, hexane / *i*PrOH = 95 / 5, 0.8 mL / min, λ = 254 nm), Rt (major) = 8.7 min, Rt (minor) = 9.8 min.

**($R_p$)-12-Bromo-16-methoxy-2,14-dioxa-1(1,4)-benzenacyclopentadecaphan-15-one (2n)**

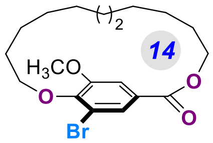


Prepared according to general procedure C, light yellow oil, 71% yield, 14.1 mg.

$[\alpha]^{25}_{D}$ = -120.4 (*c* = 0.1 in $CHCl_3$).

**$^1$H NMR** (400 MHz, $CDCl_3$) δ 7.88 (dd, *J* = 2.0, 1.0 Hz, 1H), 7.53 (t, *J* = 1.6 Hz, 1H), 4.71 – 4.65 (m, 1H), 4.64 – 4.58 (m, 1H), 4.36 – 4.27 (m, 2H), 3.93 (s, 3H), 3.81 – 3.64 (m, 1H), 1.76 – 1.70 (m, 2H), 1.40 – 1.35 (m, 2H), 1.22 – 1.15 (m, 3H), 1.11 – 0.93 (m, 7H), 0.86 – 0.78 (m, 2H), 0.71 – 0.62 (m, 2H).

**$^{13}$C NMR** (101 MHz, $CDCl_3$) δ 165.4, 152.9, 148.5, 127.1, 126.4, 117.9, 112.7, 71.7, 66.0, 58.5, 56.2, 29.4, 29.1, 28.8, 28.6, 28.4, 27.3, 26.5, 25.4, 18.5.

**HRMS** (ESI, m/z) Calcd. for $C_{19}H_{27}BrO_4Na^+$, [M+Na] $^+$: 421.0990, found: 421.0985.

**HPLC analysis**: 82:18 e.r. (IC column, 25 ºC, hexane / *i*PrOH = 95 / 5, 0.8 mL / min, λ = 254 nm), Rt (minor) = 11.0 min, Rt (major) = 16.4 min.

**($R_p$)-12-Methoxy-16-nitro-2,14-dioxa-1(1,4)-benzenacyclopentadecaphan-15-one (2o)**

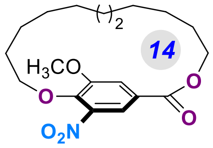


Prepared according to general procedure C, light yellow oil, 61% yield, 11.1 mg.

$[\alpha]^{25}_{D}$ = -135.3 (*c* = 0.1 in $CHCl_3$).

**$^1$H NMR** (400 MHz, $CDCl_3$) δ 8.07 (d, *J* = 2.0 Hz, 1H), 7.75 (d, *J* = 2.0 Hz, 1H), 4.75 (ddd, *J* = 11.6, 6.6, 3.6 Hz, 1H), 4.71 – 4.65 (m, 1H), 4.36 – 4.31 (m, 1H), 4.26 – 4.20 (m, 1H), 3.99 (s, 3H), 1.75 (t, *J* = 5.6 Hz, 2H), 1.43 – 1.37 (m, 2H), 1.23 – 1.13 (m, 3H), 1.10 – 0.93 (m, 7H), 0.83 (dd, *J* = 9.2, 6.0 Hz, 2H), 0.72 – 0.65 (m, 2H).

**$^{13}$C NMR** (101 MHz, $CDCl_3$) δ 152.4, 144.0, 124.2, 117.3, 115.2, 71.7, 65.4, 55.7, 28.3, 28.0, 28.0, 27.9, 27.4, 27.4, 26.2, 25.5, 24.1.

**HRMS** (ESI, m/z) Calcd. for $C_{19}H_{27}NO_6Na^+$, [M+Na] $^+$: 688.1736, found: 388.1731.

**HPLC analysis**: 90:10 e.r. (IC column, 25 °C, hexane / *i*PrOH = 95 / 5, 0.8 mL / min, λ = 254 nm), Rt (minor) = 18.5 min, Rt (major) = 27.6 min.

**(*R*p)-12-Bromo-2,13-dioxa-1(1,4)-naphthalenacyclotetradecaphan-14-one (2p)**

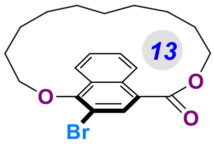


Prepared according to general procedure C, light yellow oil, 58% yield, 11.7 mg.

$[\alpha]^{25}_{D}$ = -171.6 (*c* = 0.1 in $CHCl_3$).

**$^1$H NMR** (400 MHz, $CDCl_3$) δ 8.74 (d, *J* = 7.8 Hz, 1H), 8.31 (s, 1H), 8.24 (d, *J* = 8.9 Hz, 1H), 7.63 – 7.53 (m, 2H), 4.75 – 4.68 (m, 1H), 4.56 – 4.47 (m, 2H), 4.46 – 4.39 (m, 1H), 1.71 – 1.68 (m, 3H), 1.57 – 1.49 (m, 1H), 1.35 – 1.31 (m, 3H), 1.01 – 0.93 (m, 2H), 0.73 – 0.68 (m, 2H), 0.60 – 0.51 (m, 2H), 0.50 – 0.43 (m, 2H), 0.39 – 0.29 (m, 1H).

**$^{13}$C NMR** (101 MHz, $CDCl_3$) δ 165.8, 154.9, 133.6, 130.5, 129.6, 126.9, 125.5, 125.4, 124.1, 121.8, 110.0, 72.8, 64.9, 28.4, 28.3, 27.9, 27.6, 26.6, 26.5, 25.5, 25.3.

**HRMS** (ESI, m/z) Calcd. for $C_{21}H_{25}BrO_3Na^+$, [M+Na] $^+$: 427.0885, found: 427.0879.

**HPLC analysis**: 96:4 e.r. (IC column, 25 °C, hexane / *i*PrOH = 95 / 5, 0.8 mL / min, λ = 254 nm), Rt (major) = 8.9 min, Rt (minor) = 10.0 min.

**(*R*p)-12-Bromo-2,12-dioxa-1(1,4)-naphthalenacyclotridecaphan-13-one (2q)**

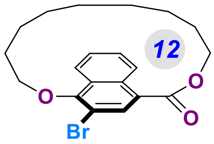


Prepared according to general procedure C, light yellow oil, 46% yield, 9.0 mg.

$[\alpha]^{25}_{D}$ = -125.4 (*c* = 0.1 in $CHCl_3$).

**$^1$H NMR** (400 MHz, $CDCl_3$) δ 8.51 (dd, *J* = 8.0, 1.6 Hz, 1H), 8.24 (dd, *J* = 8.0, 1.6 Hz, 1H), 8.18 (s, 1H), 7.63 – 7.50 (m, 2H), 4.68 (ddd, *J* = 12.0, 8.2, 1.6 Hz, 1H), 4.61 (ddd, *J* = 10.7, 6.9, 3.5 Hz, 1H), 4.42 – 4.34 (m, 2H), 1.76 – 1.64 (m, 4H), 1.51 – 1.46 (m, 1H), 0.83 – 0.62 (m, 5H), 0.60 – 0.51 (m, 1H), 0.46 – 0.34 (m, 1H), 0.15 (ddt, *J* = 18.3, 12.8, 5.8 Hz, 1H), 0.08 – 0.01 (m, 1H).

**$^{13}$C NMR** (101 MHz, $CDCl_3$) δ 166.3, 155.1, 133.2, 130.6, 129.5, 126.6, 125.9, 125.6, 125.6, 121.8,

110.1, 72.8, 66.5, 30.1, 28.9, 28.7, 28.3, 28.0, 27.3, 27.0, 25.6.

**HRMS** (ESI, m/z) Calcd. for $C_{20}H_{23}BrO_3Na^+$, $[M+Na]^+$: 413.0728, found: 413.0723.

**HPLC analysis**: 95:5 e.r. (IG column, 25 °C, hexane / *i*PrOH = 95 / 5, 0.8 mL / min, λ = 254 nm), Rt (major) = 9.0 min, Rt (minor) = 10.1 min.

**($R_p$)-12-Bromo-2,15-dioxa-1(1,4)-naphthalenacyclohexadecaphan-16-one (2s)**

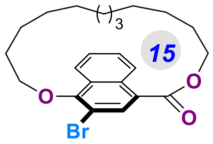


Prepared according to general procedure C, light yellow oil, 76% yield, 16.4 mg.

$[\alpha]^{25}_D$ = -213.8 (*c* = 0.1 in $CHCl_3$).

**$^1$H NMR** (400 MHz, $CDCl_3$) δ 8.97 (d, *J* = 8.6 Hz, 1H), 8.38 (s, 1H), 8.27 (d, *J* = 8.6 Hz, 1H), 7.66 – 7.60 (m, 1H), 7.58 – 7.53 (m, 1H), 4.83 – 4.73 (m, 2H), 4.44 (ddd, *J* = 11.4, 7.0, 3.9 Hz, 1H), 4.30 – 4.23 (m, 1H), 3.72 (q, *J* = 7.0 Hz, 1H), 1.85 – 1.75 (m, 3H), 1.17 – 1.03 (m, 3H), 0.98 – 0.77 (m, 9H), 0.70 – 0.51 (m, 4H).

**$^{13}$C NMR** (101 MHz, $CDCl_3$) δ 166.1, 156.8, 135.2, 132.0, 130.1, 128.1, 126.3, 123.7, 123.0, 110.4, 66.3, 58.5, 30.4, 29.6, 29.1, 28.6, 27.9, 27.4, 27.2, 25.7, 18.5.

**HRMS** (ESI, m/z) Calcd. for $C_{23}H_{29}BrO_3Na^+$, $[M+Na]^+$: 455.1198, found: 455.1192.

**HPLC analysis**: 93:7 e.r. (IC column, 25 °C, hexane / *i*PrOH = 95 / 5, 0.8 mL / min, λ = 254 nm), Rt (minor) = 9.6 min, Rt (major) = 10.9 min.

**($R_p$)-12-Bromo-2,16-dioxa-1(1,4)-naphthalenacycloheptadecaphan-17-one (2t)**

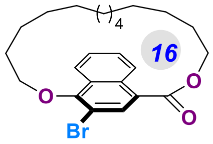


Prepared according to general procedure C, light yellow oil, 74% yield, 16.5 mg.

$[\alpha]^{25}_D$ = -203.6 (*c* = 0.1 in $CHCl_3$).

**$^1$H NMR** (400 MHz, $CDCl_3$) δ 8.98 (d, J = 9.0 Hz, 1H), 8.38 (s, 1H), 8.27 (d, J = 7.0 Hz, 1H), 7.63 (ddd, J = 8.5, 6.8, 1.5 Hz, 1H), 7.56 (ddd, J = 8.2, 6.8, 1.3 Hz, 1H), 4.82 (dt, J = 11.6, 6.5 Hz, 1H), 4.76 – 4.70 (m, 1H), 4.38 (dt, J = 11.6, 6.8 Hz, 1H), 4.28 – 4.22 (m, 1H), 1.84 – 1.78 (m, 2H),

1.70 – 1.61 (m, 2H), 1.46 – 1.41 (m, 1H), 1.11 – 0.94 (m, 8H), 0.93 – 0.70 (m, 7H)..

**$^{13}$C NMR** (101 MHz, $CDCl_3$) δ 165.7, 156.1, 135.2, 132.1, 128.1, 126.6, 126.3, 123.7, 123.1, 73.8, 65.26, 29.1, 29.0, 28.5, 28.4, 27.9, 27.7, 27.6, 27.5, 25.6, 24.6.

**HRMS** (ESI, m/z) Calcd. for $C_{24}H_{31}BrO_3Na^+$, $[M+Na]^+$: 469.1354, found: 469.0983.

**HPLC analysis**: 86:14 e.r. (IC column, 25 ºC, hexane / *i*PrOH = 95 / 5, 0.8 mL / min, λ = 254 nm), Rt (minor) = 7.4 min, Rt (major) = 8.4 min.

**(*R*$_p$)-1$^2$-Bromo-2,13-dioxa-6,10-dithia-1(1,4)-naphthalenacyclotetradecaphan-14-one--methane (2u)**

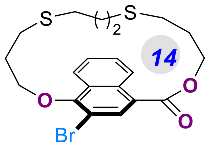


Prepared according to general procedure C, light yellow oil, 60% yield, 13.7 mg.

$[\alpha]^{25}_{D}$ = -71.5 (*c* = 0.1 in $CHCl_3$).

**$^1$H NMR** (400 MHz, $CDCl_3$) δ 8.92 (d, *J* = 8.2 Hz, 1H), 8.40 (s, 1H), 8.27 (s, 1H), 7.64 (ddd, *J* = 8.5, 6.8, 1.5 Hz, 1H), 7.58 (ddd, *J* = 8.2, 6.8, 1.3 Hz, 1H), 4.97 – 4.90 (m, 1H), 4.77 (ddd, *J* = 11.2, 6.9, 4.0 Hz, 1H), 4.55 – 4.49 (m, 1H), 4.35 (ddd, *J* = 11.0, 6.6, 4.0 Hz, 1H), 2.59 (dq, *J* = 13.4, 6.8 Hz, 2H), 2.25 – 2.04 (m, 6H), 1.98 – 1.91 (m, 2H), 1.86 – 1.81 (m, 1H), 1.60 – 1.53 (m, 1H), 1.24 – 1.15 (m, 2H).

**$^{13}$C NMR** (101 MHz, $CDCl_3$) δ 166.0, 156.1, 135.1, 131.8, 130.1, 128.3, 126.9, 126.4, 124.1, 122.69, 110.6, 72.3, 63.7, 31.3, 31.1, 30.1, 29.9, 29.6, 28.4, 28.1.

**HRMS** (ESI, m/z) Calcd. for $C_{20}H_{23}BrO_3S_2Na^+$, $[M+Na]^+$: 477.0164, found: 477.0159.

**HPLC analysis**: 94:6 e.r. (IE column, 25 ºC, hexane / *i*PrOH = 95 / 5, 0.8 mL / min, λ = 254 nm), Rt (major) = 26.4 min, Rt (minor) = 31.7 min.

**(*R*$_p$)-13-Bromo-4-((12-hydroxydodeca-5,7-diyn-1-yl)oxy)-naphthalenacycloheptadecaphan-16-one (2v)**

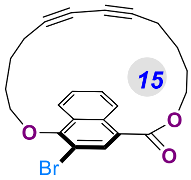

Prepared according to general procedure C, light yellow oil, 45% yield, 9.5 mg.

$[\alpha]^{25}_{D}$ = -81.8 ($c$ = 0.1 in $CHCl_3$).

**$^1$H NMR** (400 MHz, $CDCl_3$) δ 8.97 (d, $J$ = 8.1 Hz, 1H), 8.45 (s, 1H), 8.22 (d, $J$ = 7.1 Hz, 1H), 7.63 (ddd, $J$ = 8.5, 6.8, 1.5 Hz, 1H), 7.55 (ddd, $J$ = 8.3, 6.9, 1.3 Hz, 1H), 4.93 – 4.79 (m, 2H), 4.40 – 4.31 (m, 1H), 4.11 (dt, $J$ = 10.9, 5.0 Hz, 1H), 2.52 – 2.43 (m, 1H), 2.25 (dt, $J$ = 13.6, 4.6 Hz, 1H), 2.10 (ddd, $J$ = 13.1, 9.0, 4.5 Hz, 1H), 2.01 – 1.94 (m, 1H), 1.86 – 1.71 (m, 5H), 1.58 – 1.50 (m, 1H), 1.43 – 1.38 (m, 1H), 1.17 (td, $J$ = 9.3, 5.5 Hz, 1H).

**$^{13}$C NMR** (101 MHz, $CDCl_3$) δ 165.9, 154.5, 135.7, 132.2, 130.5, 128.0, 126.6, 126.4, 123.8, 122.7, 111.4, 77.0, 75.4, 72.7, 66.3, 65.4, 64.1, 27.9, 26.5, 25.7, 23.6, 18.7, 18.3.

**HRMS** (ESI, m/z) Calcd. for $C_{23}H_{21}BrO_3Na^+$, $[M+Na]^+$: 447.0566, found: 447.0564.

**HPLC analysis**: 91:9 e.r. (IG column, 25 °C, hexane / *i*PrOH = 95 / 5, 0.8 mL / min, λ = 254 nm), Rt (major) = 10.1 min, Rt (minor) = 10.6 min.

**(*R*$_p$)-12-Bromo-2,8,14-trioxa-1(1,4)-naphthalenacyclopentadecaphan-15-one (2w)**

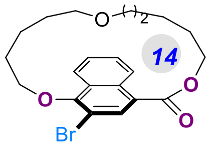


Prepared according to general procedure C, light yellow oil, 66% yield, 13.8 mg.

$[\alpha]^{25}_{D}$ = -101.8 ($c$ = 0.1 in $CHCl_3$).

**$^1$H NMR** (400 MHz, $CDCl_3$) δ 8.89 (d, $J$ = 8.0 Hz, 1H), 8.38 (s, 1H), 8.24 (d, $J$ = 8.0 Hz, 1H), 7.62 (ddd, $J$ = 8.6, 6.8, 1.5 Hz, 1H), 7.55 (ddd, $J$ = 8.2, 6.8, 1.3 Hz, 1H), 4.83 (ddd, $J$ = 12.0, 7.5, 2.7 Hz, 1H), 4.70 (ddd, $J$ = 11.2, 7.1, 4.3 Hz, 1H), 4.39 (tdd, $J$ = 10.8, 7.1, 3.4 Hz, 2H), 3.15 (dt, $J$ = 12.0, 4.4 Hz, 1H), 3.06 – 2.99 (m, 1H), 2.90 (dt, $J$ = 8.8, 5.4 Hz, 1H), 2.69 (ddd, $J$ = 8.8, 7.1, 5.4 Hz, 1H), 1.81 – 1.73 (m, 3H), 1.43 – 1.33 (m, 5H), 1.18 – 1.12 (m, 1H), 1.02 – 0.95 (m, 1H), 0.89 – 0.81 (m, 1H), 0.72 – 0.57 (m, 2H).

**$^{13}$C NMR** (101 MHz, $CDCl_3$) δ 166.4, 156.4, 135.1, 132.0, 130.1, 128.0, 126.6, 126.5, 123.9, 122.8, 111.0, 74.0, 70.5, 70.4, 65.2, 30.8, 29.6, 29.1, 27.8, 24.5, 22.8.

**HRMS** (ESI, m/z) Calcd. for $C_{21}H_{25}BrO_4Na^+$, $[M+Na]^+$: 443.0828, found: 443.0826.

**HPLC analysis**: 96:4 e.r. (IE column, 25 °C, hexane / *i*PrOH = 95 / 5, 0.8 mL / min, λ = 254 nm), Rt (major) = 17.6 min, Rt (minor) = 20.4 min.

**($R_p$)-12-(4-(Tert-butyl)phenyl)-2,14-dioxa-1(1,4)-naphthalenacyclopentadecaphan-15-one (7)**

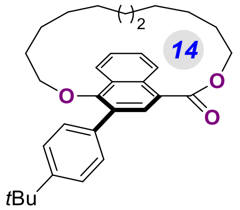


Light yellow oil, 85% yield, 20.1 mg.

$[\alpha]^{25}_{D}$ = -142.7 (*c* = 0.1 in $CHCl_3$).

**$^1$H NMR** (400 MHz, $CDCl_3$) δ 8.99 (d, *J* = 7.2 Hz, 1H), 8.44 (d, *J* = 7.2 Hz, 1H), 8.37 (s, 1H), 7.66 – 7.63 (m, 2H), 7.61 – 7.53 (m, 2H), 7.52 – 7.49 (m, 2H), 4.70 (ddd, *J* = 11.2, 6.8, 4.4 Hz, 1H), 4.33 (ddd, *J* = 10.8, 6.8, 4.4 Hz, 1H), 4.04 – 3.97 (m, 1H), 3.93 (ddd, *J* = 12.0, 8.0, 3.6 Hz, 1H), 1.82 (q, *J* = 4.8, 3.6 Hz, 2H), 1.39 (s, 9H), 1.30 – 1.22 (m, 6H), 1.14 – 1.08 (m, 1H), 1.00– 0.94 (m, 1H), 0.78 – 0.65 (m, 5H), 0.58 – 0.48 (m, 2H), 0.44– 0.36 (m, 1H).

**$^{13}$C NMR** (101 MHz, $CDCl_3$) δ 167.4, 156.2, 150.6, 135.6, 134.1, 132.0, 130.4, 128.7, 127.7, 127.0, 125.9, 125.8, 125.7, 123.5, 122.2, 71.5, 65.2, 34.7, 31.4, 29.5, 29.0, 29.0, 28.6, 28.5, 28.1, 27.5, 26.4, 25.5.

**HRMS** (ESI, m/z) Calcd. for $C_{32}H_{40}O_3Na^+$, $[M+Na]^+$: 495.2875, found: 495.2870.

**HPLC analysis**: 94:6 e.r. (IC column, 25 °C, hexane / *i*PrOH = 95 / 5, 0.8 mL / min, λ = 254 nm), Rt (major) = 8.6 min, Rt (minor) = 9.6 min

**($R_p$)-(E)-12-styryl-2,14-dioxa-1(1,4)-naphthalenacyclopentadecaphan-15-one (9)**

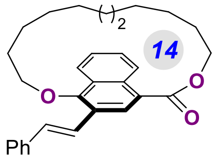


Light yellow oil, 42% yield, 9.3 mg.

$[\alpha]^{25}_{D}$ = -73.2 (*c* = 0.05 in $CHCl_3$).

**$^1$H NMR** (400 MHz, $CDCl_3$) δ 8.89 (d, *J* = 9.7 Hz, 1H), 8.58 (s, 1H), 8.27 (d, *J* = 10.1 Hz, 1H), 7.64 – 7.52 (m, 5H), 7.41 (s, 2H), 7.30 (t, *J* = 3.6 Hz, 2H), 4.69 (ddd, *J* = 11.1, 6.6, 4.6 Hz, 1H), 4.54 (ddd, *J* = 11.2, 6.6, 4.2 Hz, 1H), 4.46 – 4.40 (m, 2H), 1.86 – 1.79 (m, 2H), 1.48 – 1.38 (m, 3H), 1.23 – 1.18 (m, 2H), 1.08 – 1.02 (m, 1H), 0.99 – 0.94 (m, 1H), 0.89 (q, *J* = 5.6, 4.8 Hz, 1H), 0.76 – 0.59 (m, 7H), 0.50 (q, *J* = 6.6 Hz, 1H).

$^{13}$**C NMR** (101 MHz, $CDCl_3$) δ 166.8, 155.9, 137.6, 136.9, 134.7, 131.5, 130.6, 128.5, 127.8, 126.6, 126.4, 126.2, 125.1, 122.8, 113.8, 111.0, 73.8, 65.9, 29.4, 29.3, 28.9, 28.6, 27.6, 27.4, 26.5, 26.3.

**HRMS** (ESI, m/z) Calcd. for $C_{30}H_{34}O_3Na^+$, [M+Na] $^+$: 465.2406, found: 465.2400.

**HPLC analysis**: 95:5 e.r. (IC column, 25 °C, hexane / *i*PrOH = 95 / 5, 0.8 mL / min, λ = 254 nm), Rt (major) = 8.7 min, Rt (minor) = 9.8 min

**(*R*$_p$)-12-(Phenylethynyl)-2,14-dioxa-1(1,4)-naphthalenacyclopentadecaphan-15-one (11)**

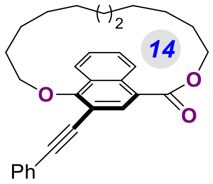


Light yellow oil, 92% yield, 20.2 mg.

$[\alpha]^{25}_D$ = -145.5 (*c* = 0.1 in $CHCl_3$).

$^1$**H NMR** (400 MHz, Chloroform-*d*) δ 8.91 (d, *J* = 8.2 Hz, 1H), 8.38 (d, *J* = 6.8 Hz, 1H), 8.32 (s, 1H), 7.65 – 7.53 (m, 4H), 7.44 – 7.36 (m, 3H), 5.38 (ddd, *J* = 11.2, 7.2, 3.6 Hz, 1H), 4.69 (ddd, *J* = 11.0, 6.8, 4.2 Hz, 1H), 4.50 (ddd, *J* = 11.4, 7.2, 3.6 Hz, 1H), 4.38 (ddd, *J* = 11.0, 6.6, 4.2 Hz, 1H), 1.88 – 1.57 (m, 5H), 1.51 – 1.35 (m, 2H), 1.23 – 1.10 (m, 2H), 1.05 – 0.83 (m, 5H), 0.73 – 0.66 (m, 2H), 0.60 – 0.39 (m, 2H).

$^{13}$**C NMR** (101 MHz, Chloroform-*d*) δ 167.0, 160.3, 135.0, 132.2, 131.4, 129.4, 128.6, 128.5, 126.3, 126.0, 123.3, 123.1, 122.4, 109.2, 95.4, 86.3, 72.1, 65.5, 29.5, 29.1, 28.8, 28.5, 28.5, 28.2, 27.3, 26.5, 25.5.

**HRMS** (ESI, m/z) Calcd. for $C_{30}H_{32}O_3Na^+$, [M+Na] $^+$: 463.2249, found: 463.2244.

**HPLC analysis**: 97:3 e.r. (IC column, 25 °C, hexane / *i*PrOH = 95 / 5, 0.8 mL / min, λ = 254 nm), Rt (major) = 12.4 min, Rt (minor) = 13.5 min

# III. Supplementary Figures

## $^{1}$H, $^{13}$C NMR spectra and HPLC traces

**Supplementary Figure 13.** $^{1}$H and $^{13}$C NMR spectra of **S2**

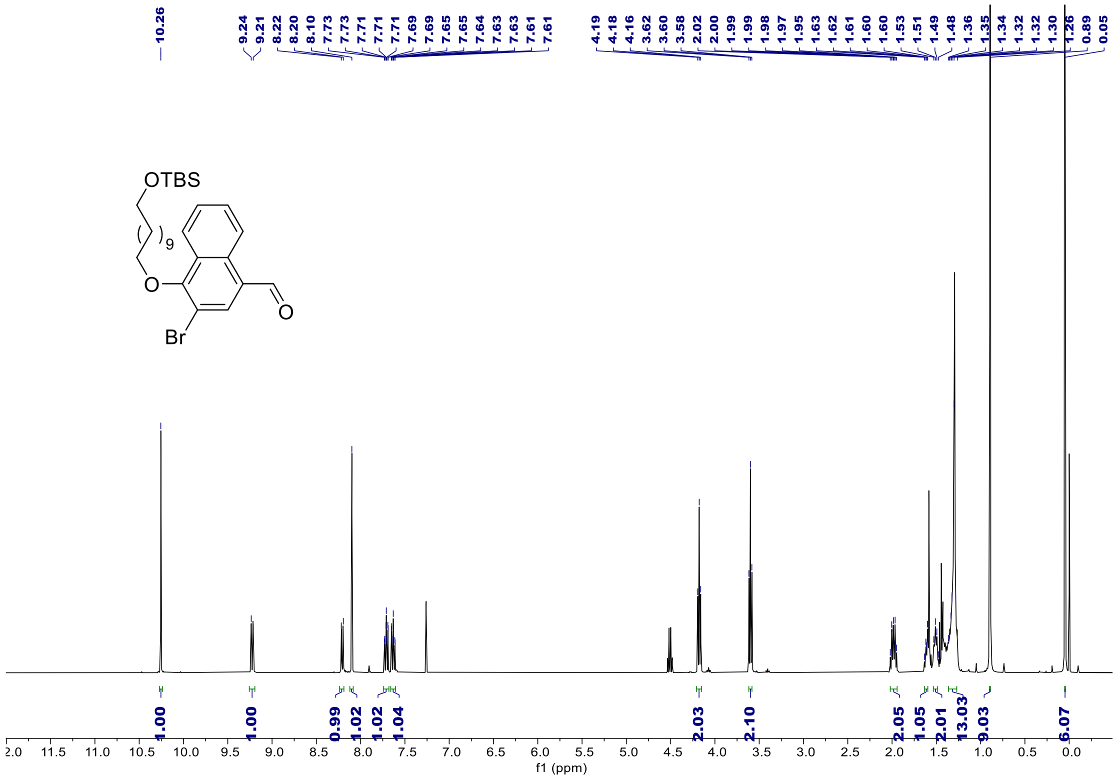


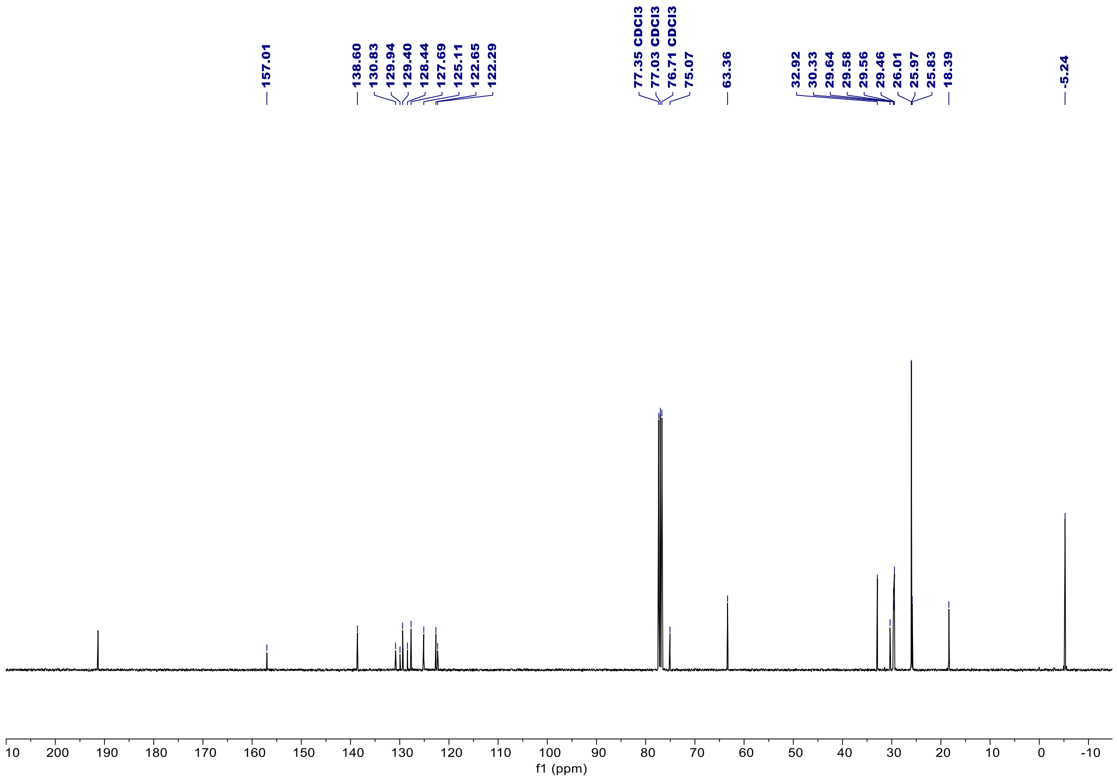

**Supplementary Figure 14.** $^{1}H$ and $^{13}C$ NMR spectra of **S3a**

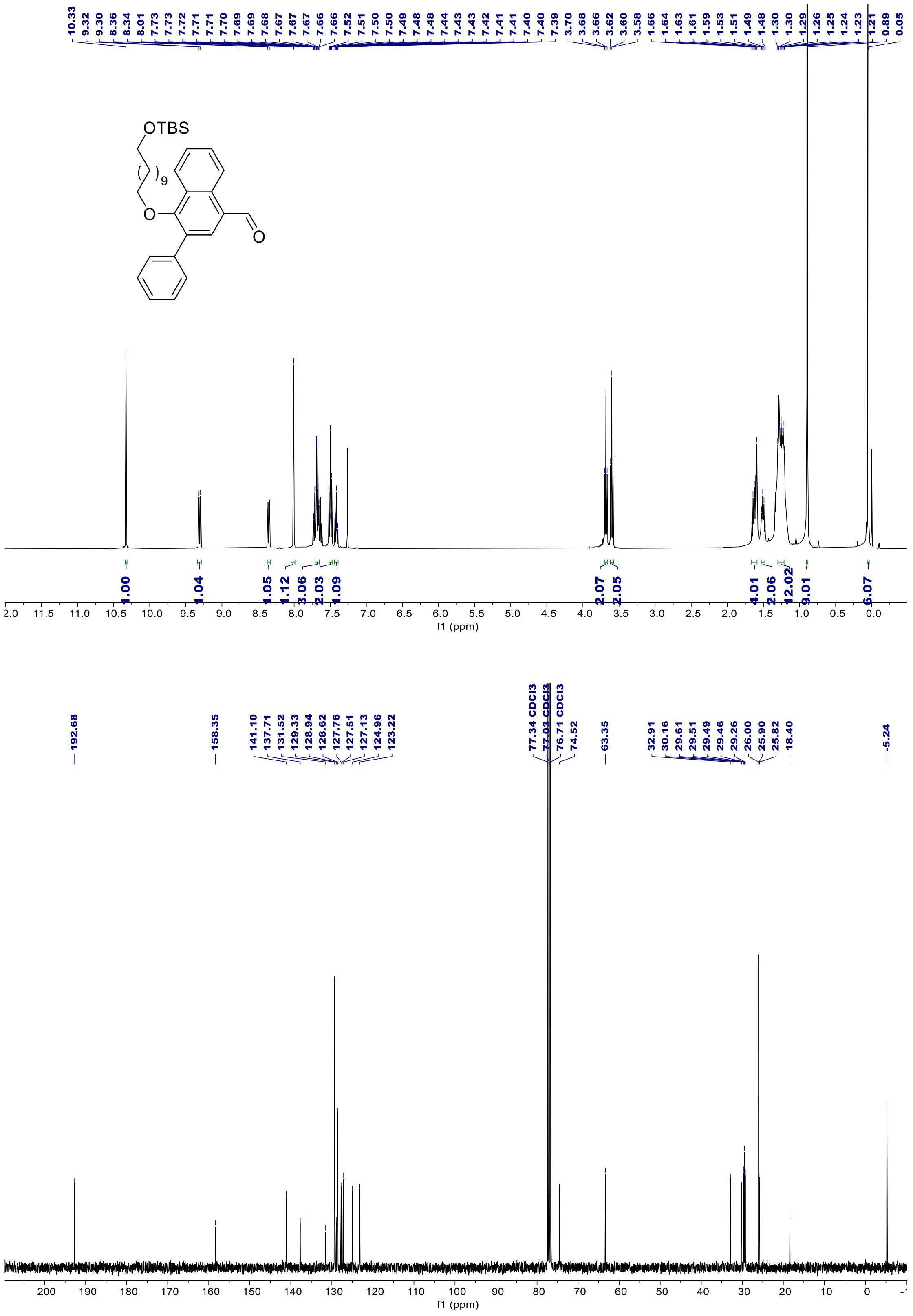

**Supplementary Figure 15.** $^{1}$H and $^{13}$C NMR spectra of **S3b**

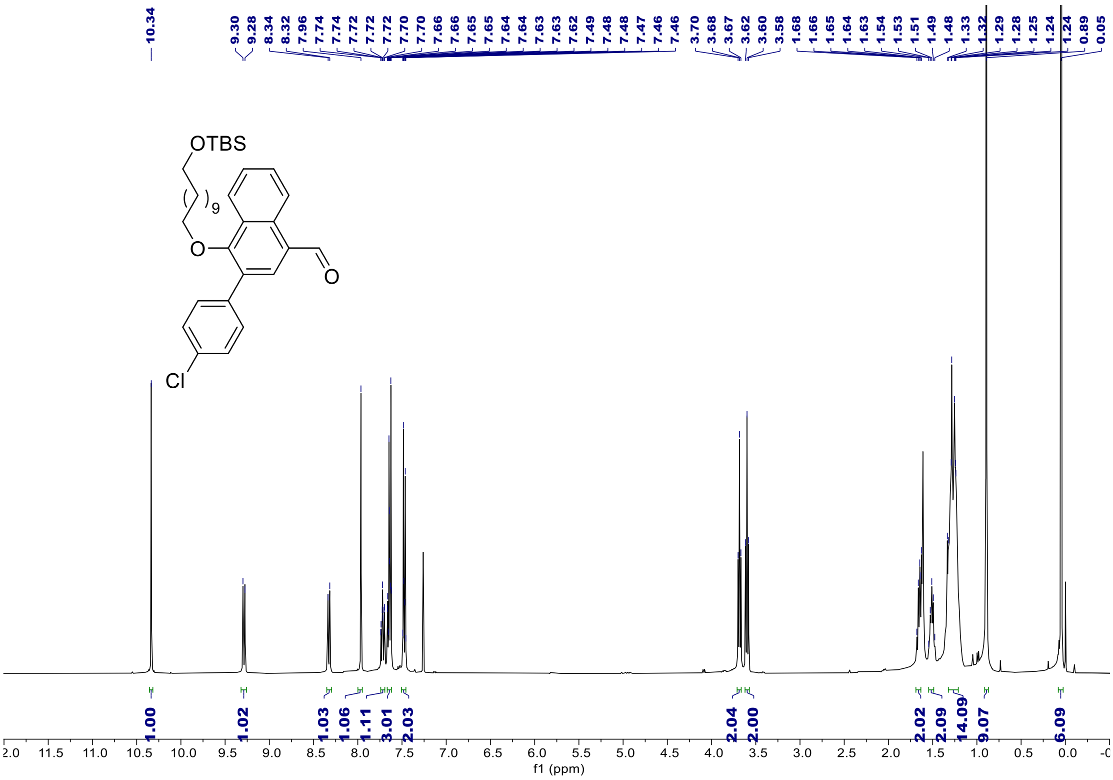


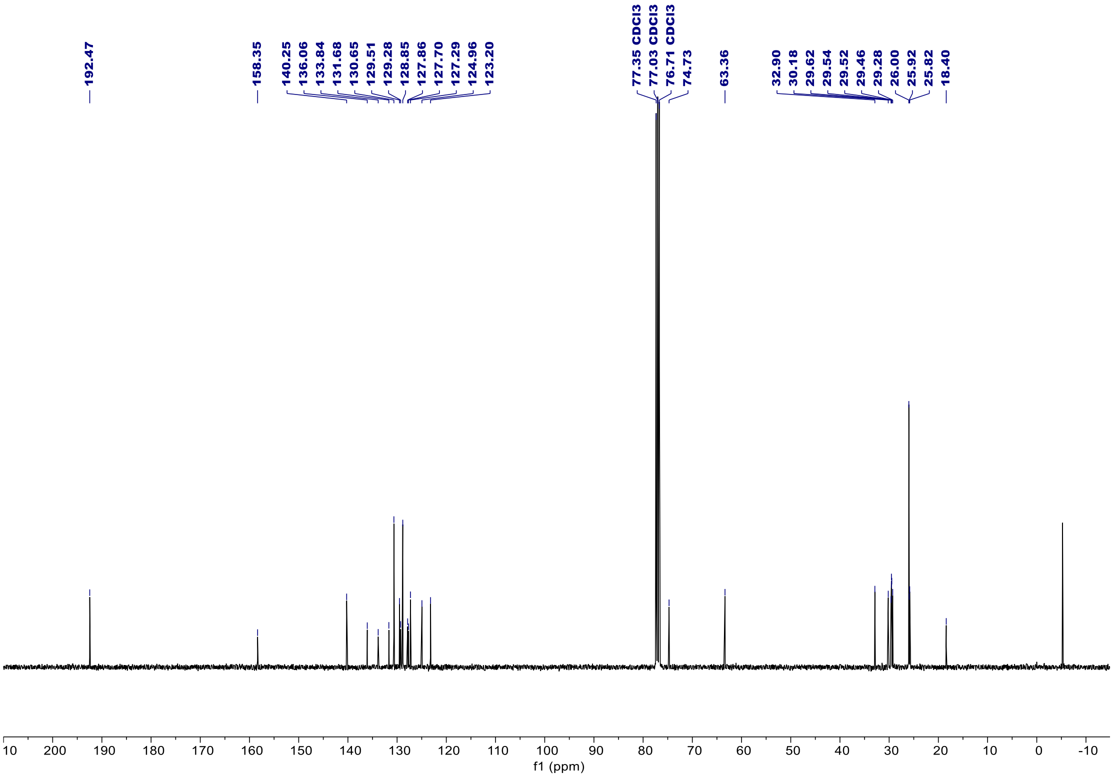

**Supplementary Figure 16.** $^{1}H$ and $^{13}C$ NMR spectra of **S3c**

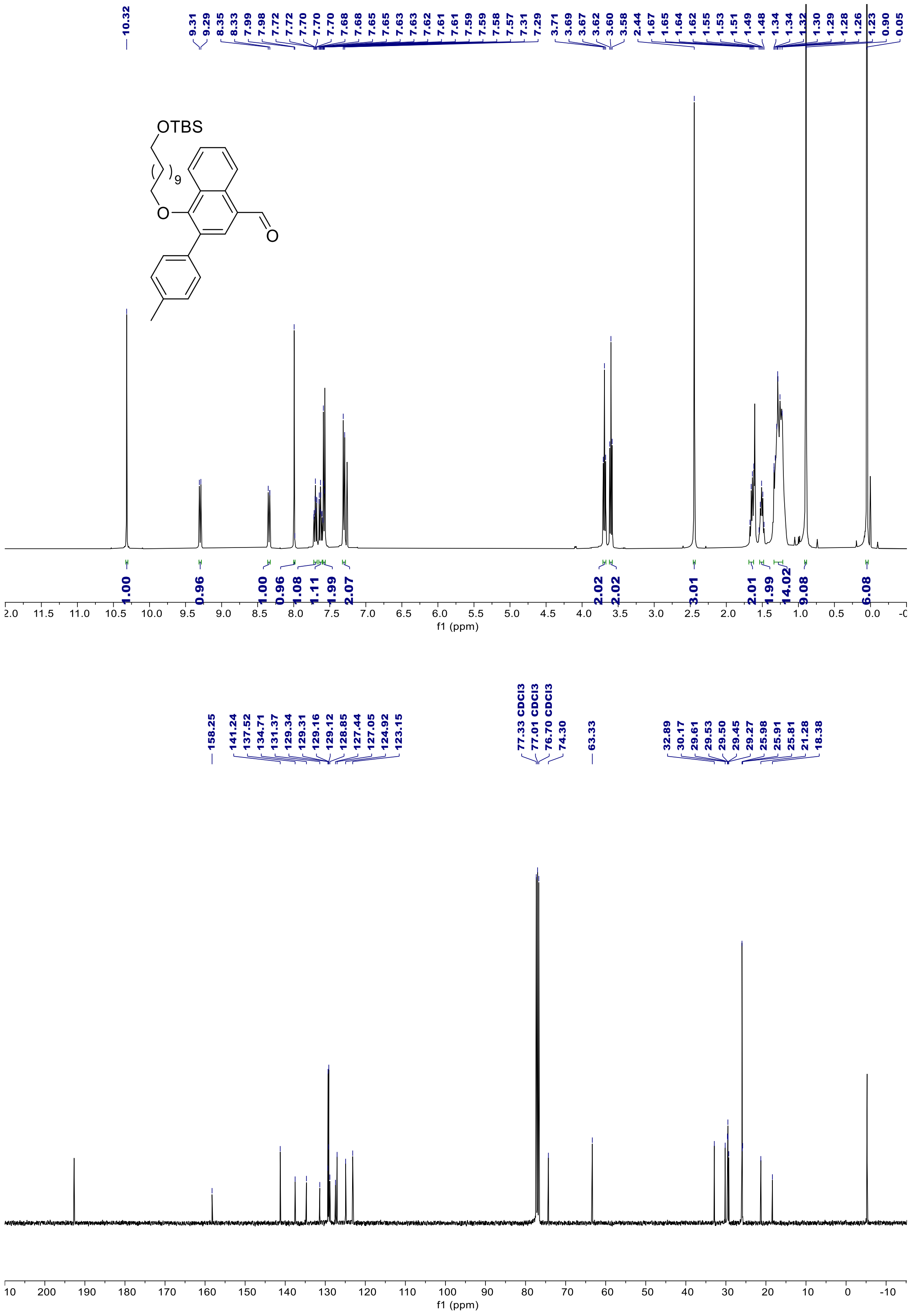

**Supplementary Figure 17.** $^{1}H$ and $^{13}C$ NMR spectra of **S3d**

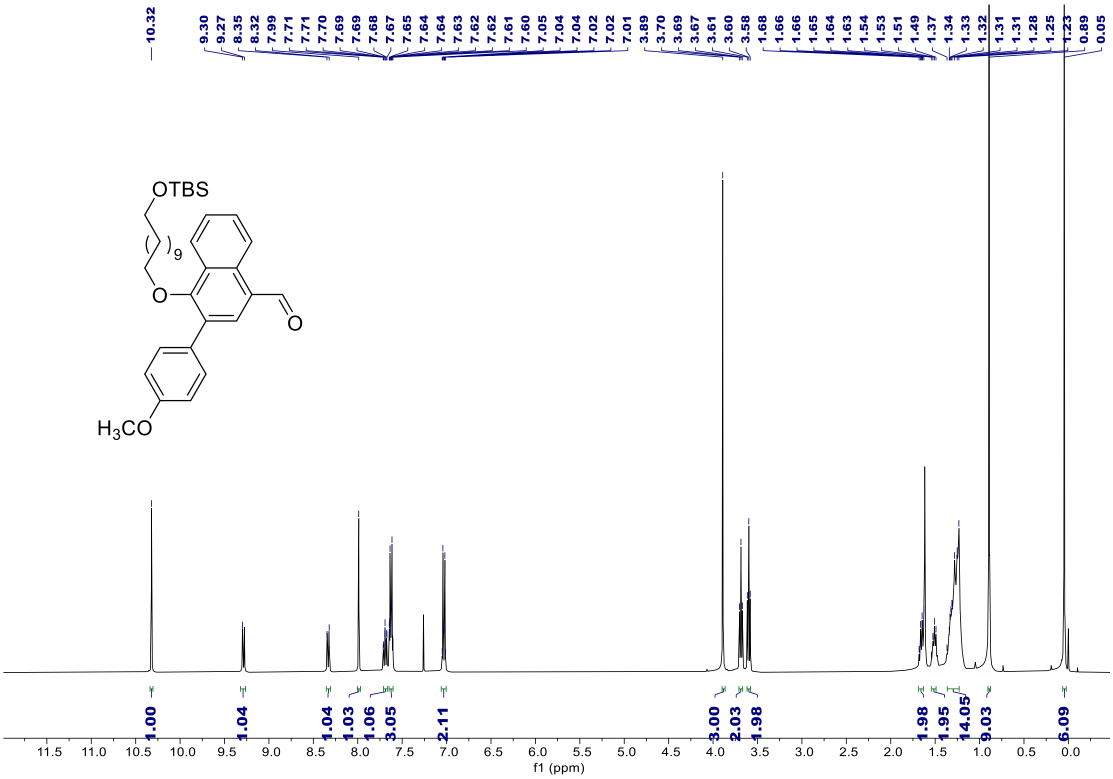


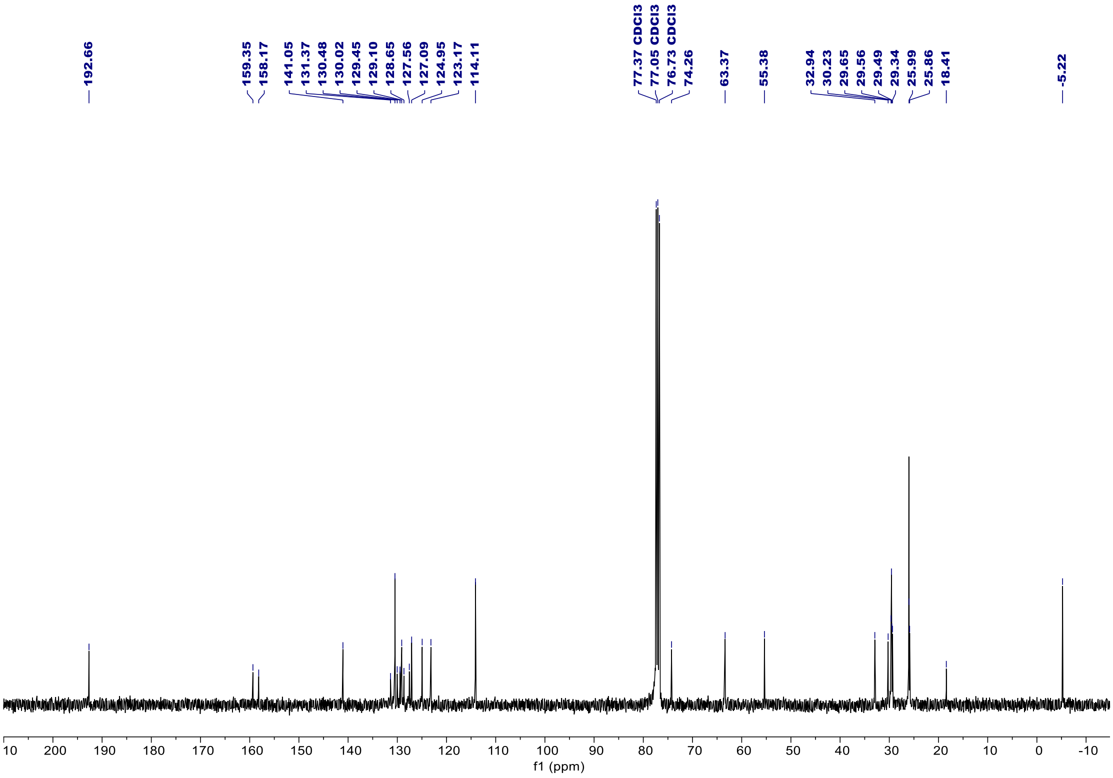

**Supplementary Figure 18.** $^{1}$H and $^{13}$C NMR spectra of **S3e**

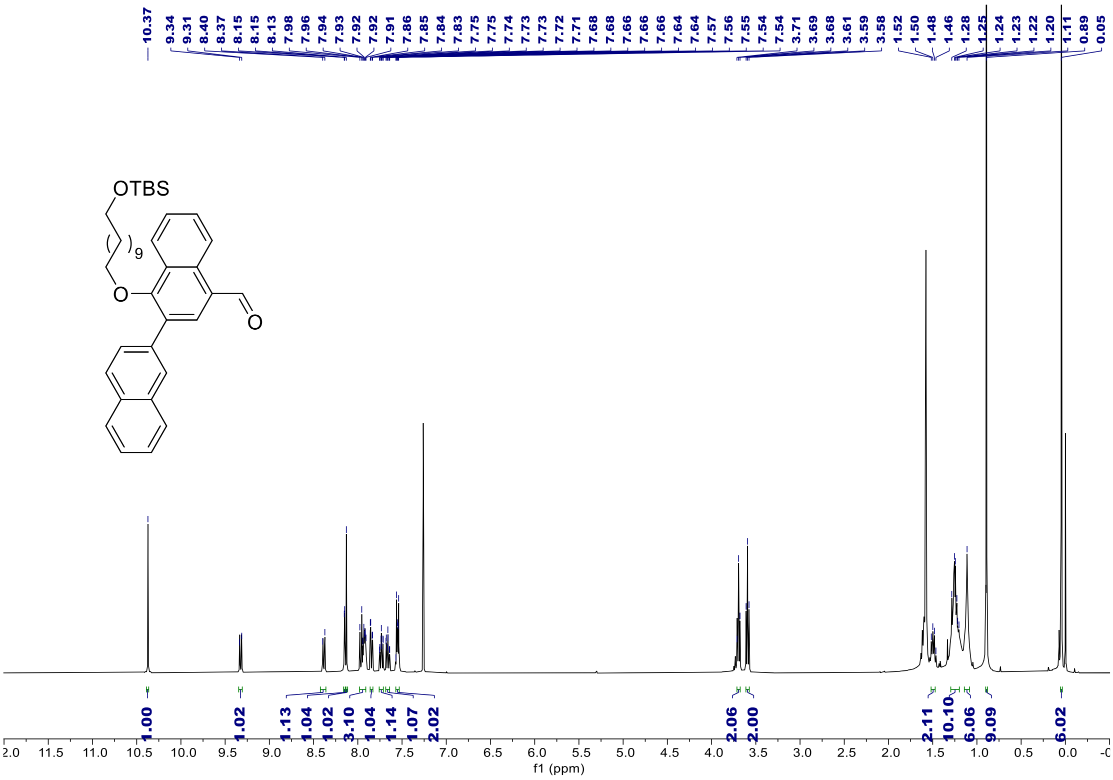


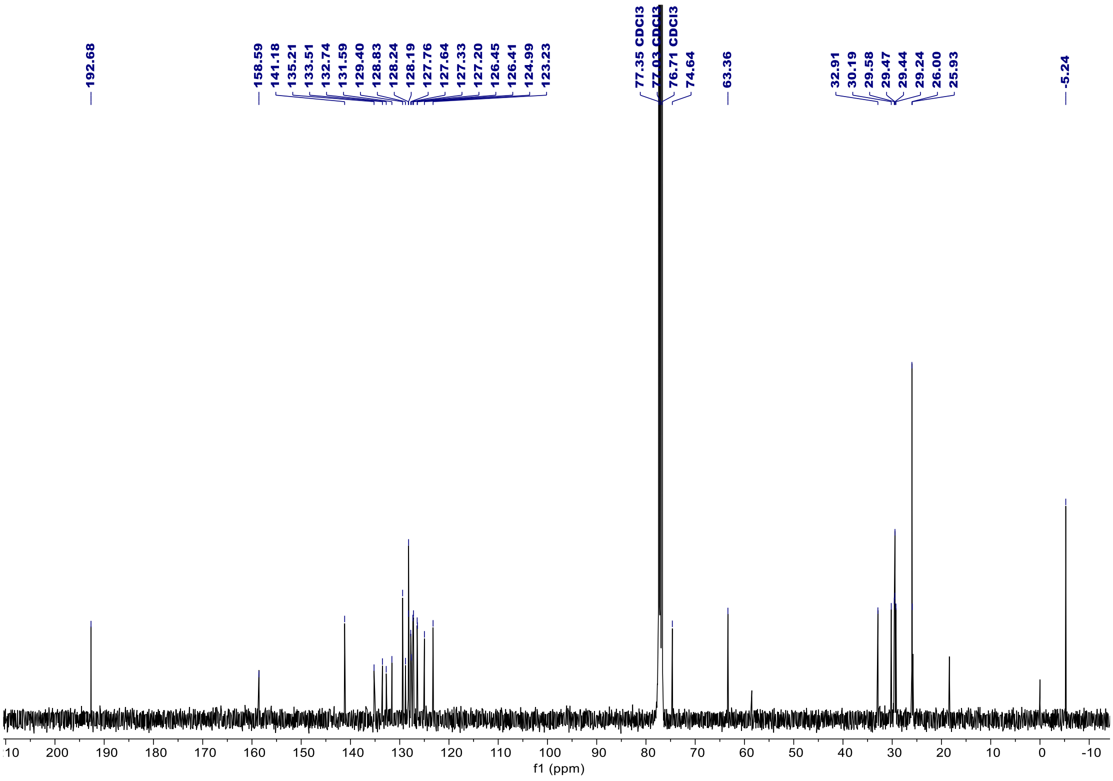

**Supplementary Figure 19.** $^{1}H$ and $^{13}C$ NMR spectra of **S3f**

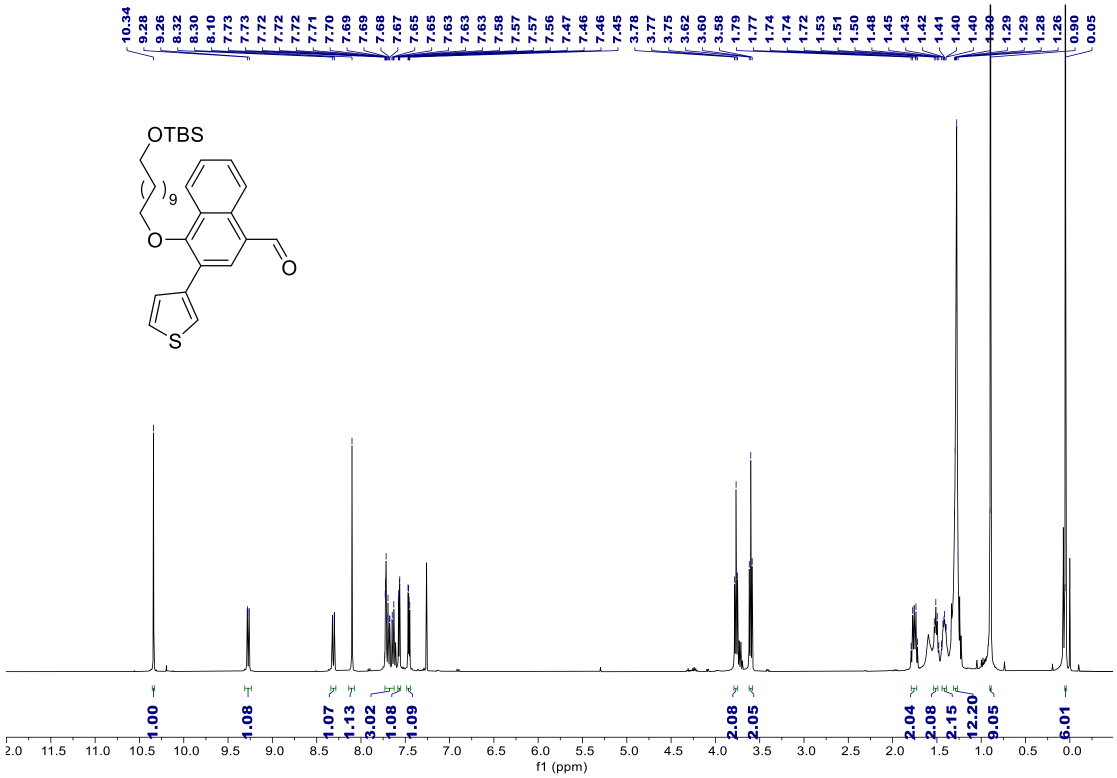


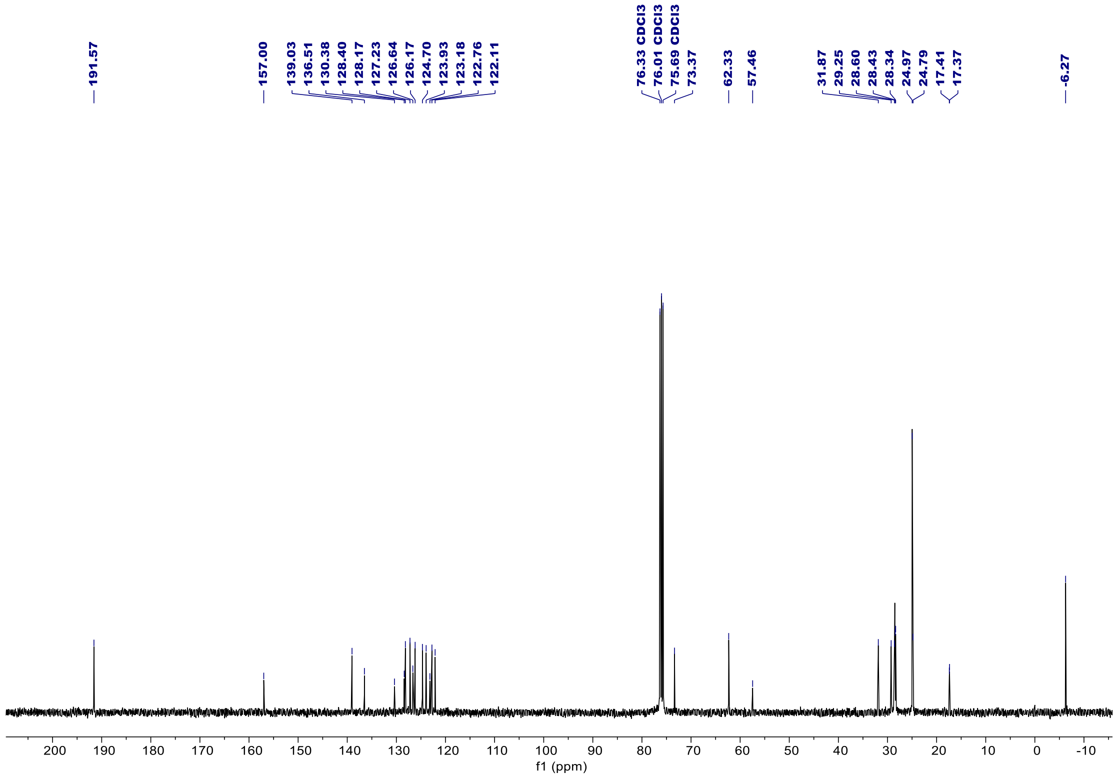

**Supplementary Figure 20.** $^{1}$H and $^{13}$C NMR spectra of **S3g**

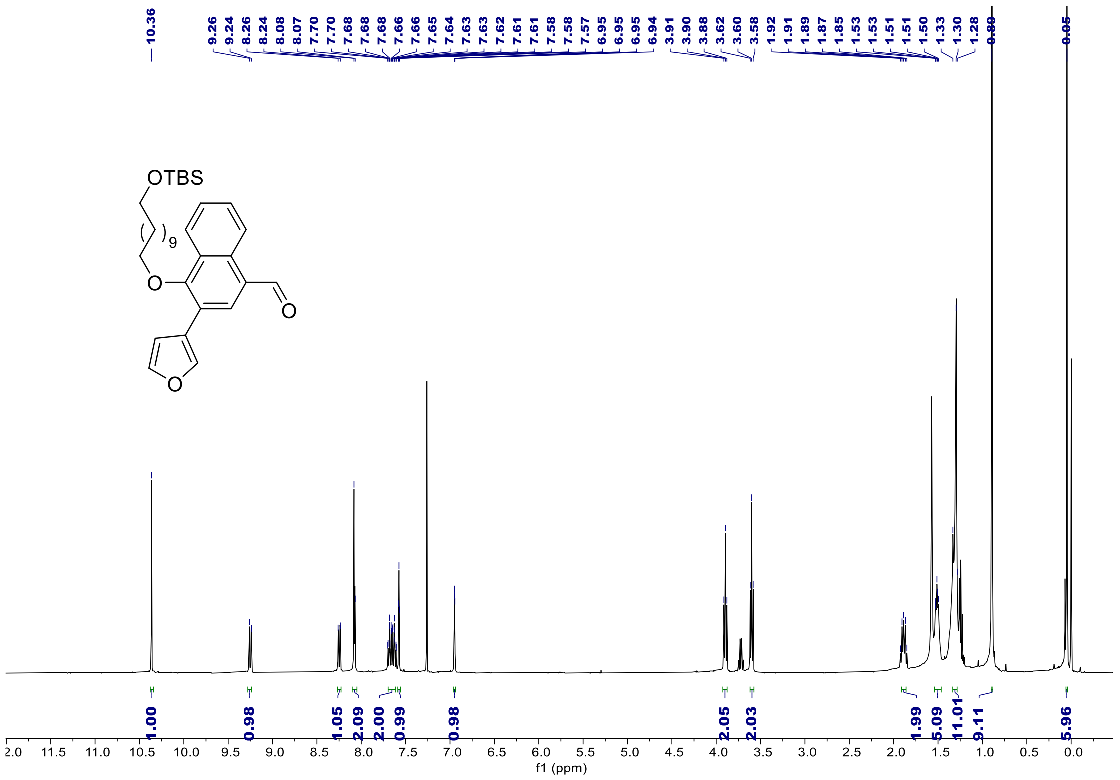


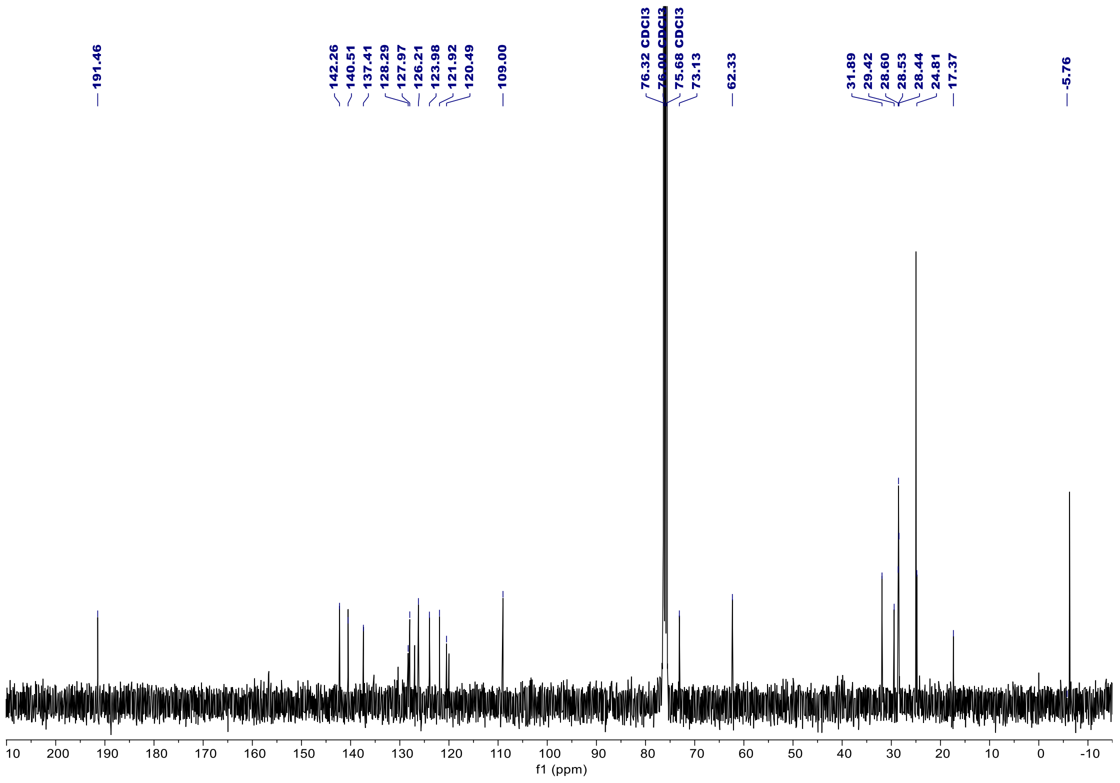

**Supplementary Figure 21.** $^{1}$H and $^{13}$C NMR spectra of **S3h**

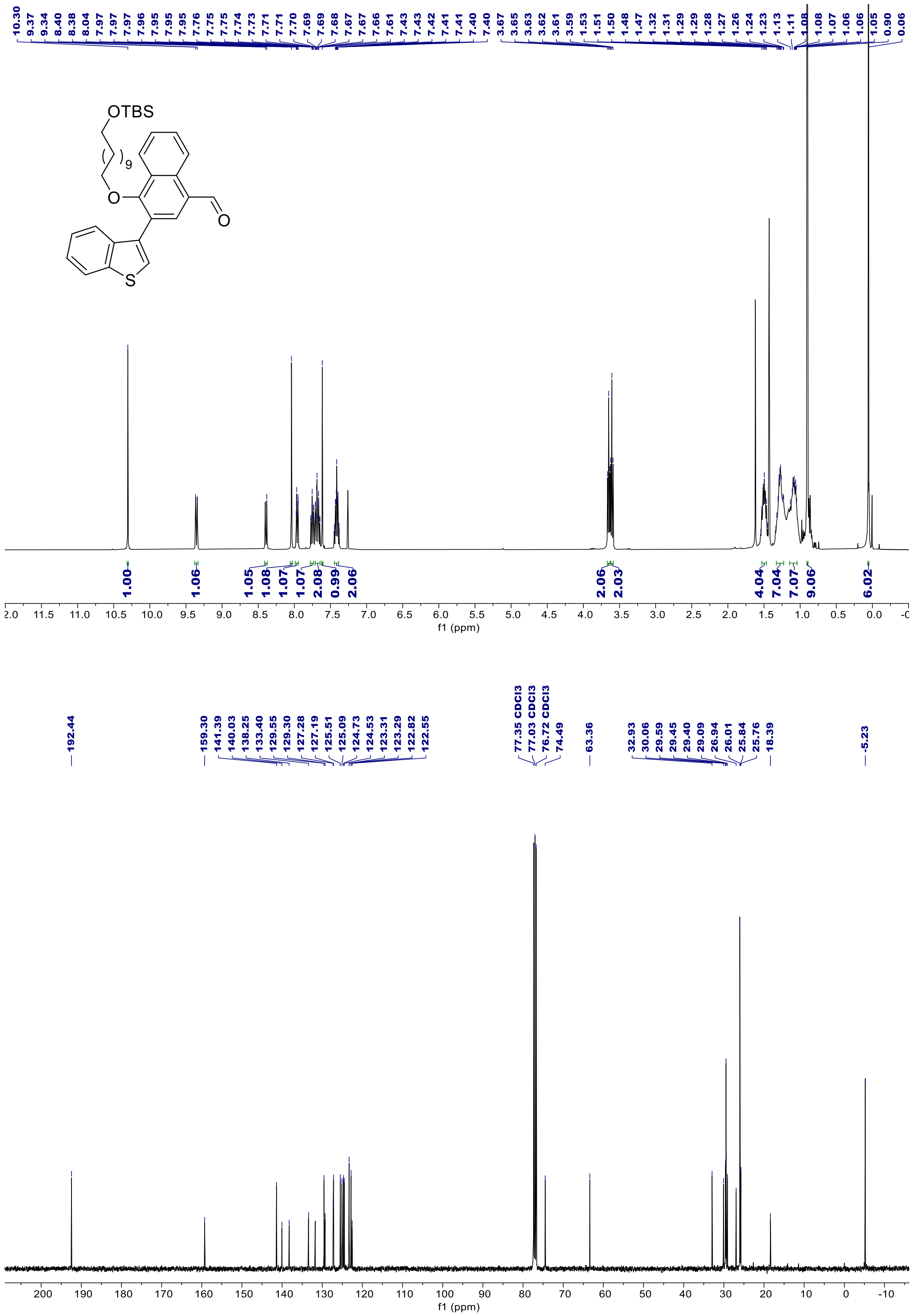

**Supplementary Figure 22.** $^{1}H$ and $^{13}C$ NMR spectra of **S3i**

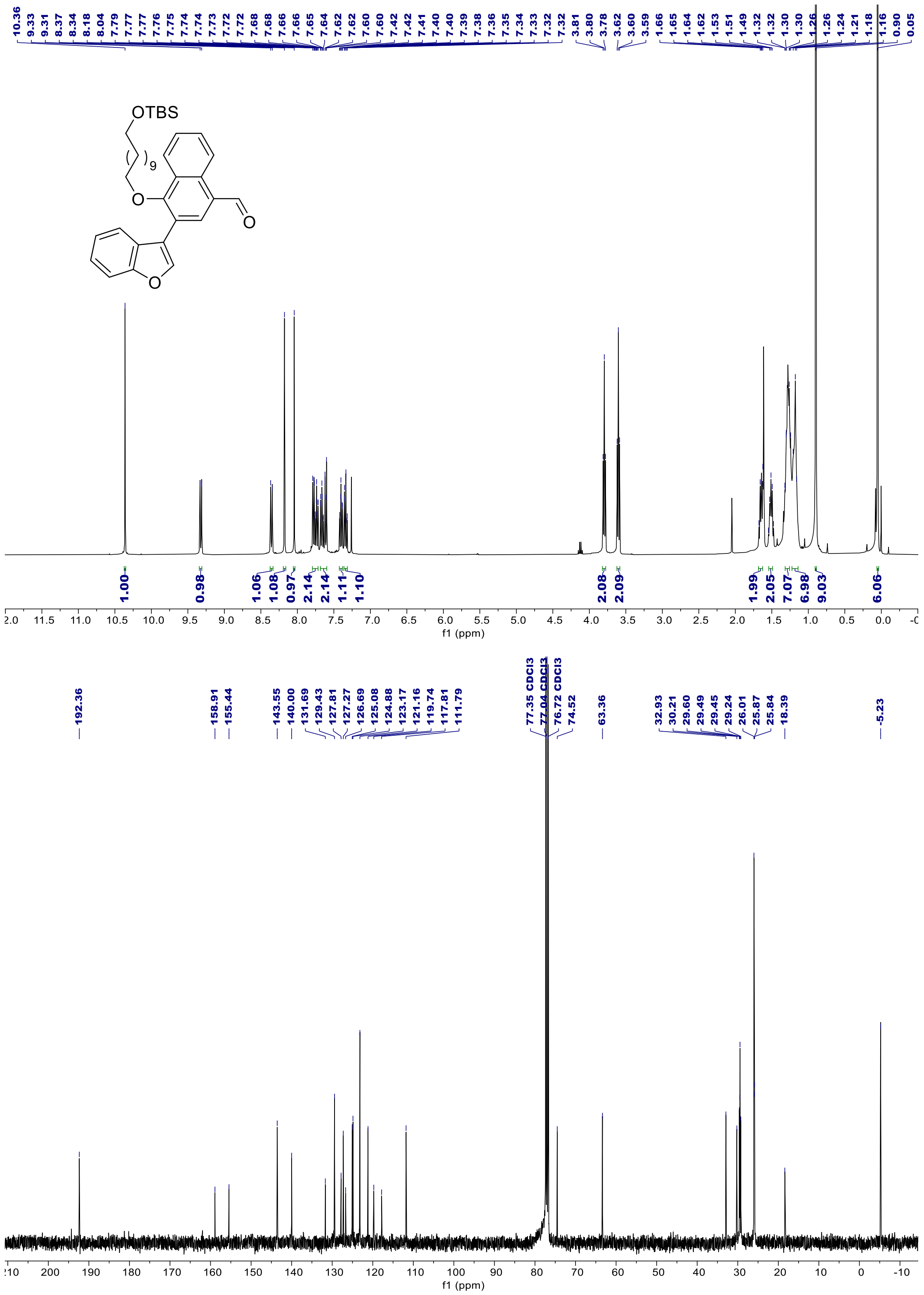

**Supplementary Figure 23.** $^{1}$H and $^{13}$C NMR spectra of **1a**

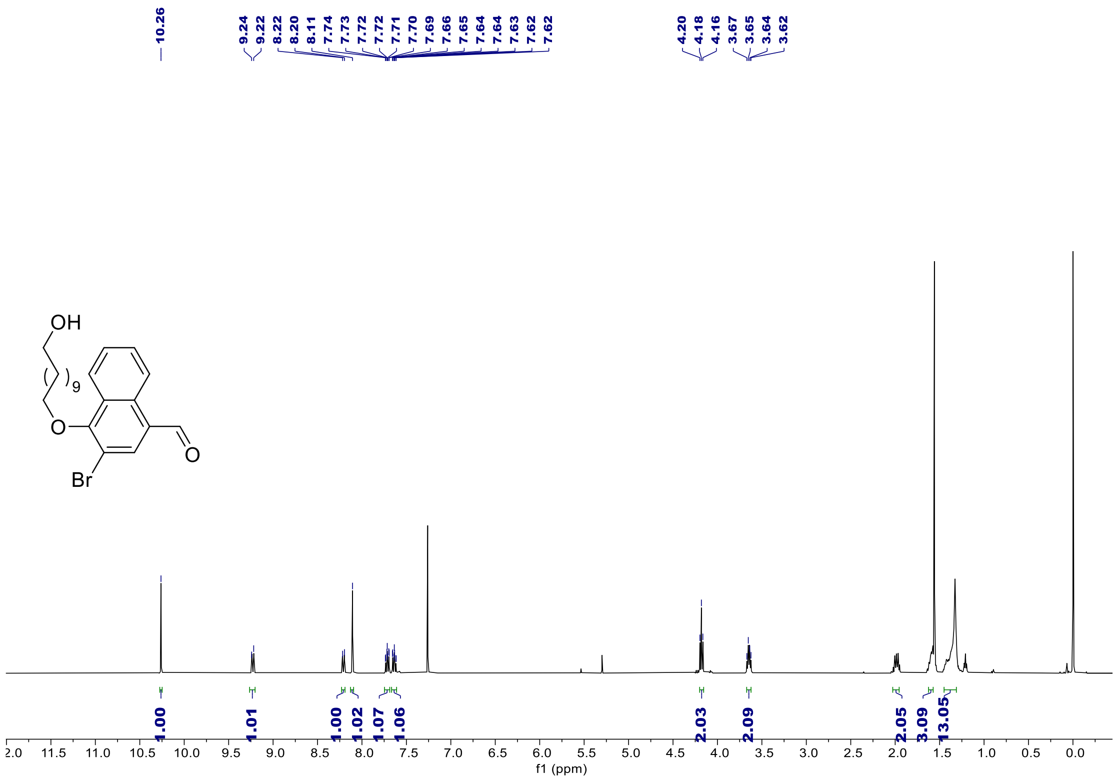


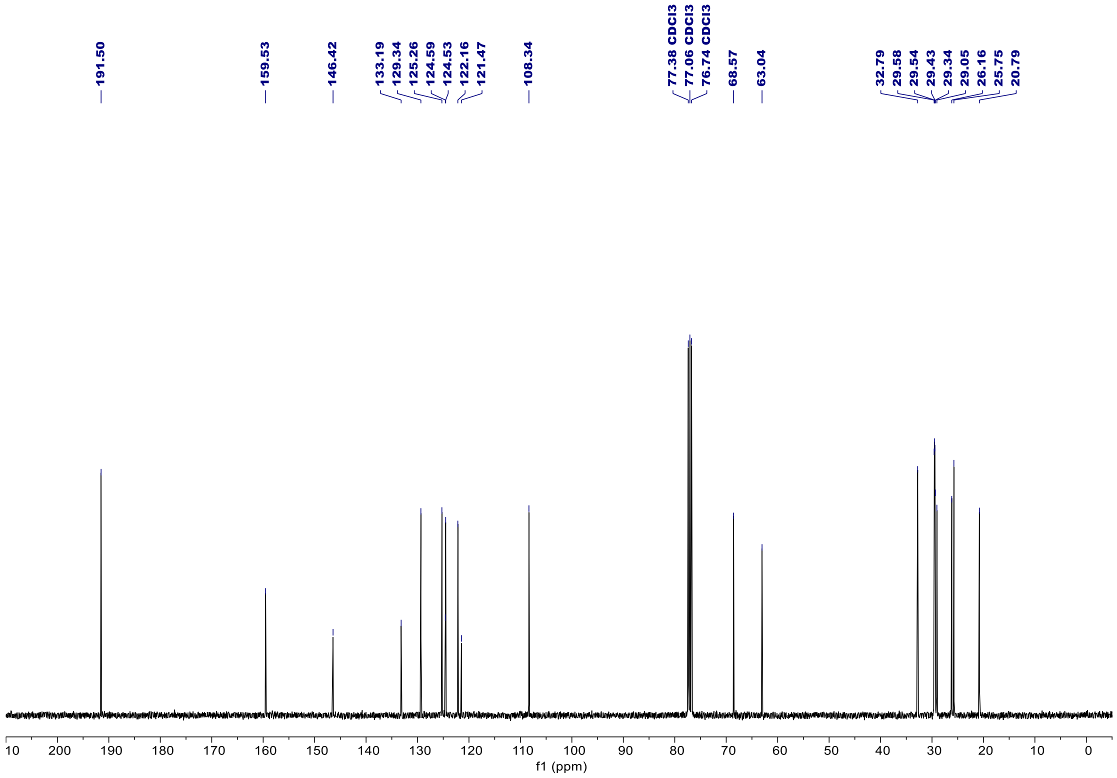

**Supplementary Figure 24.** $^{1}$H and $^{13}$C NMR spectra of **1b**

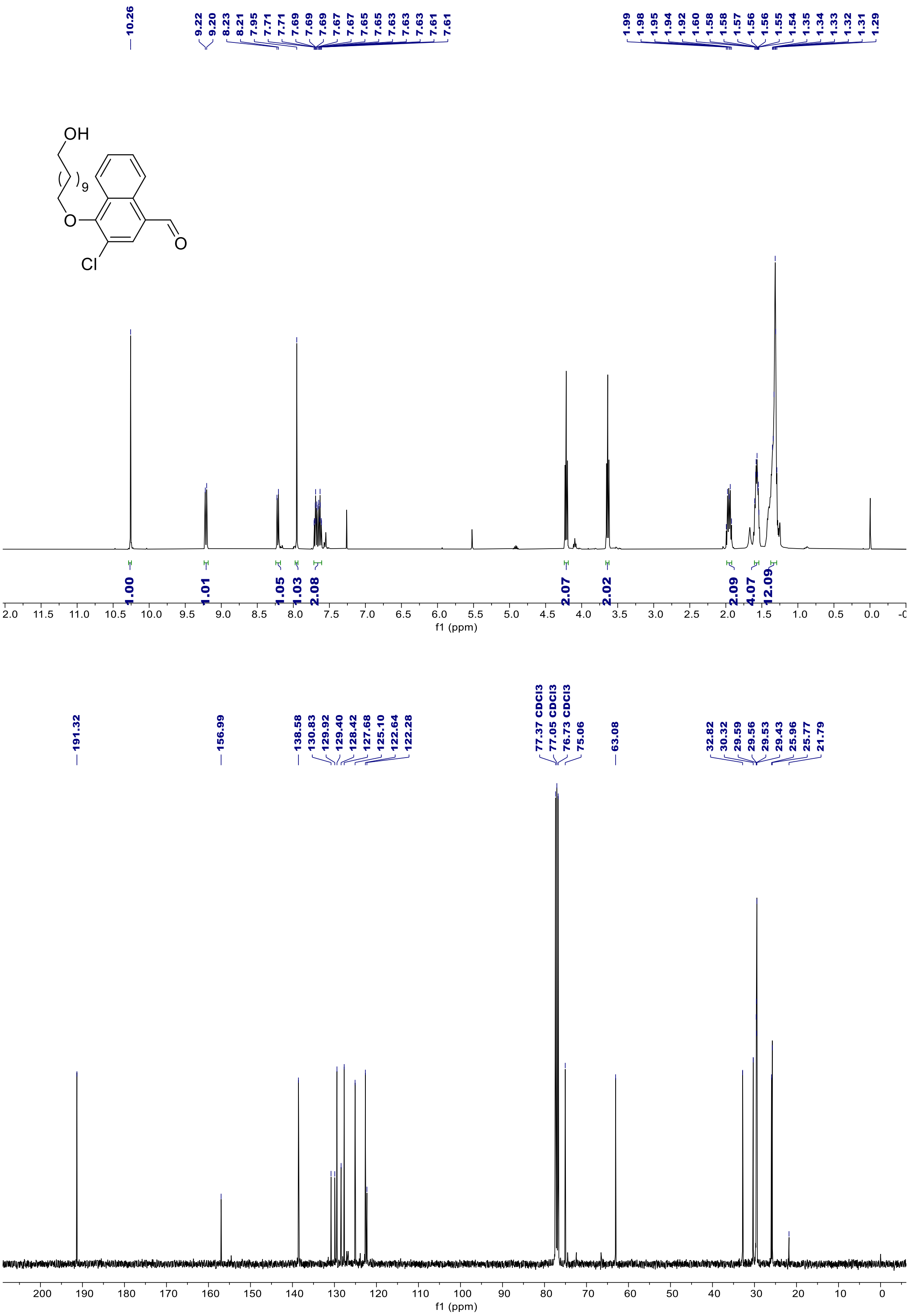

**Supplementary Figure 25.** $^{1}$H and $^{13}$C NMR spectra of **1c**

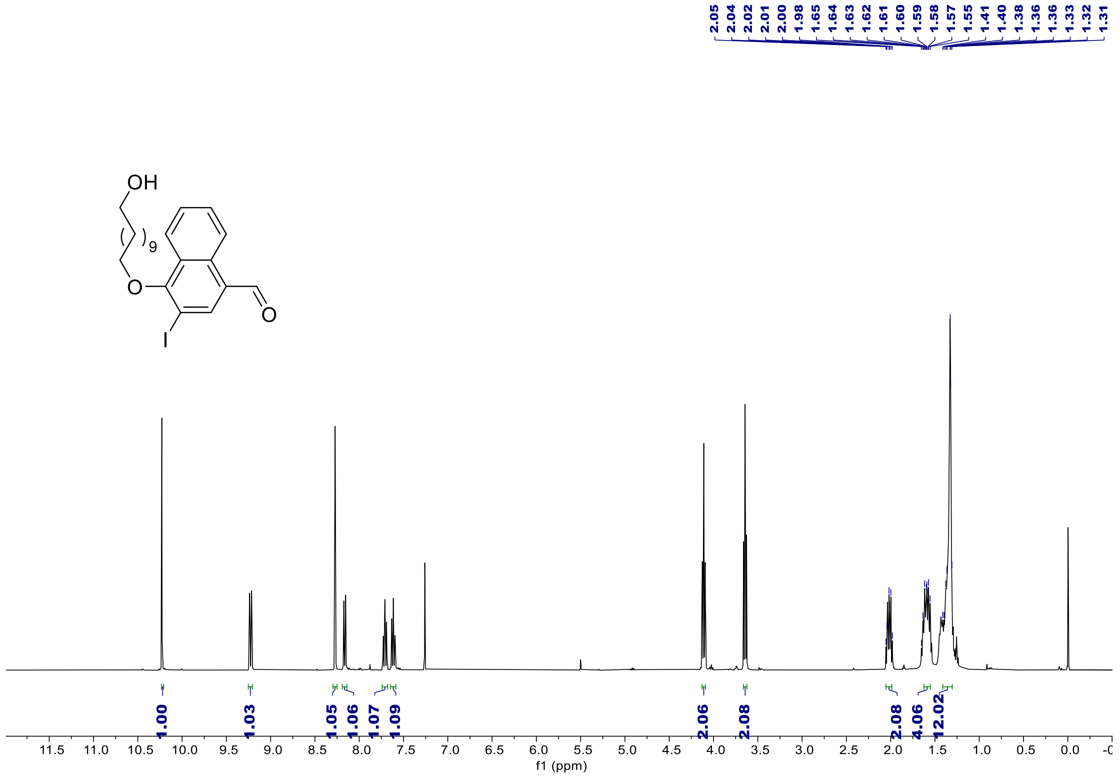


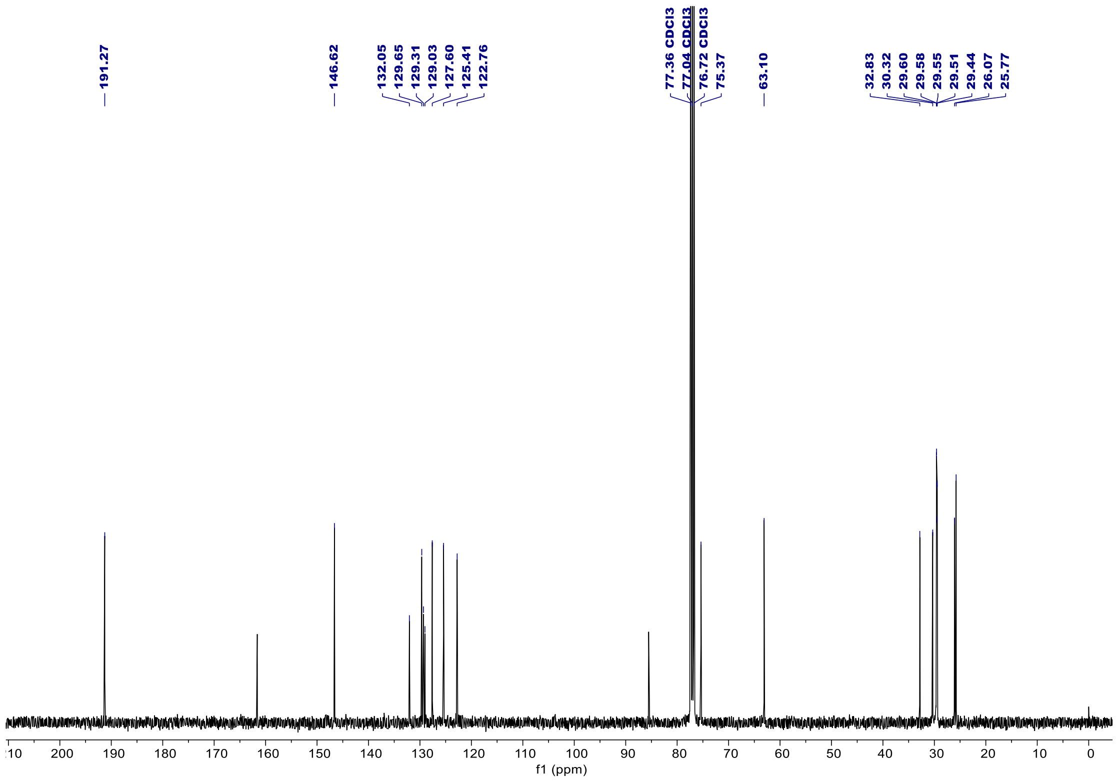

**Supplementary Figure 26.** $^{1}$H and $^{13}$C NMR spectra of **1d**

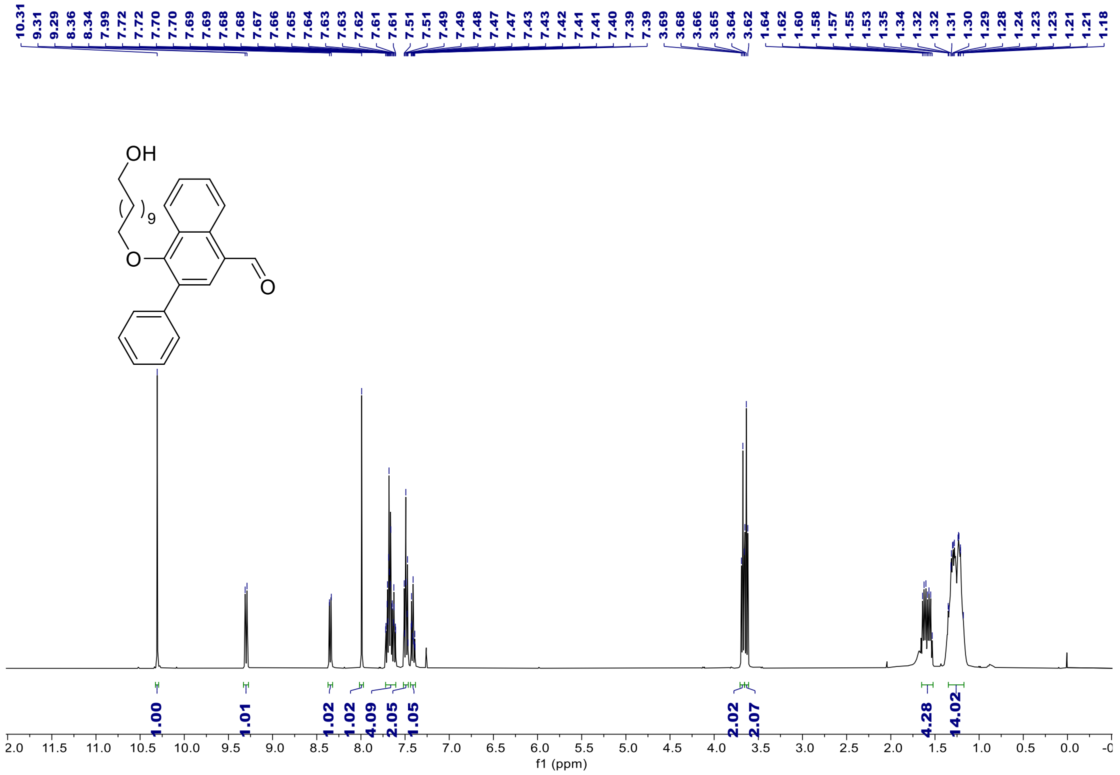

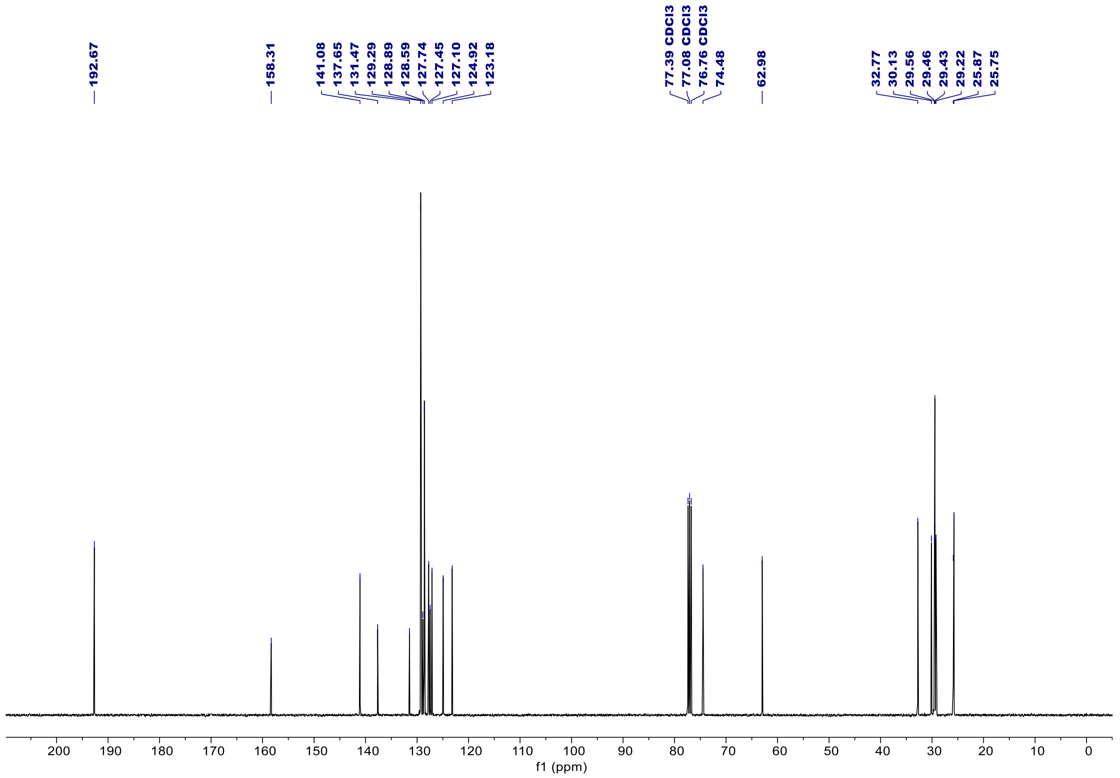

**Supplementary Figure 27.** $^{1}$H and $^{13}$C NMR spectra of **1e**

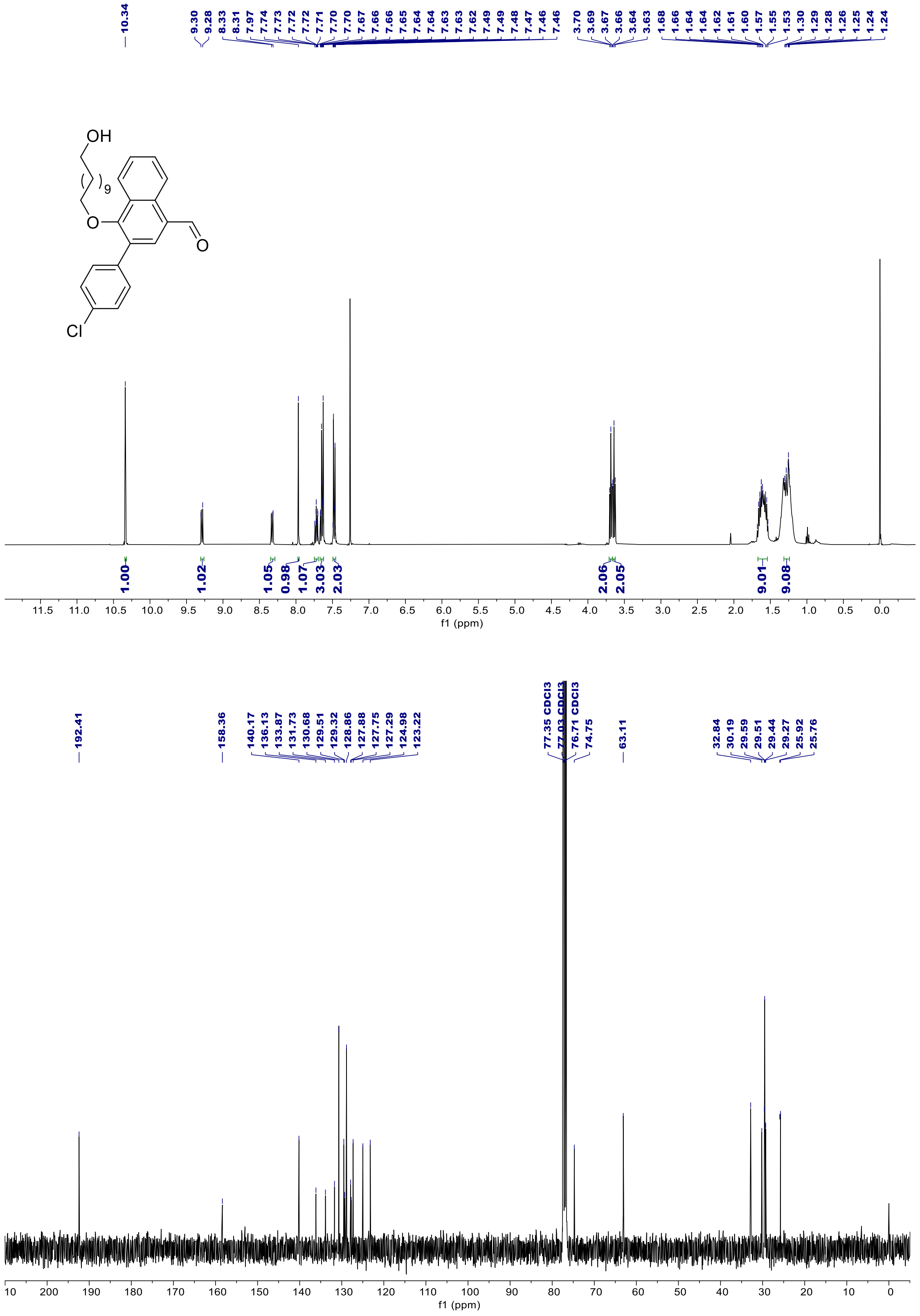

**Supplementary Figure 28.** $^{1}$H and $^{13}$C NMR spectra of **1f**

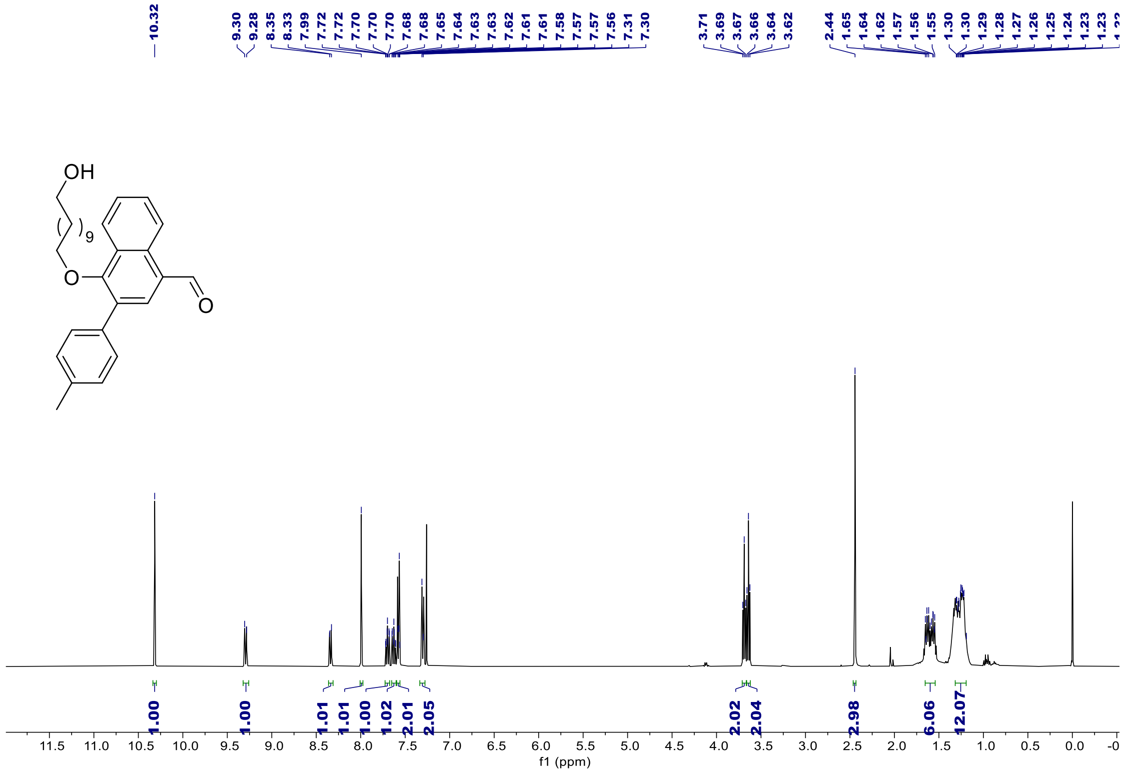


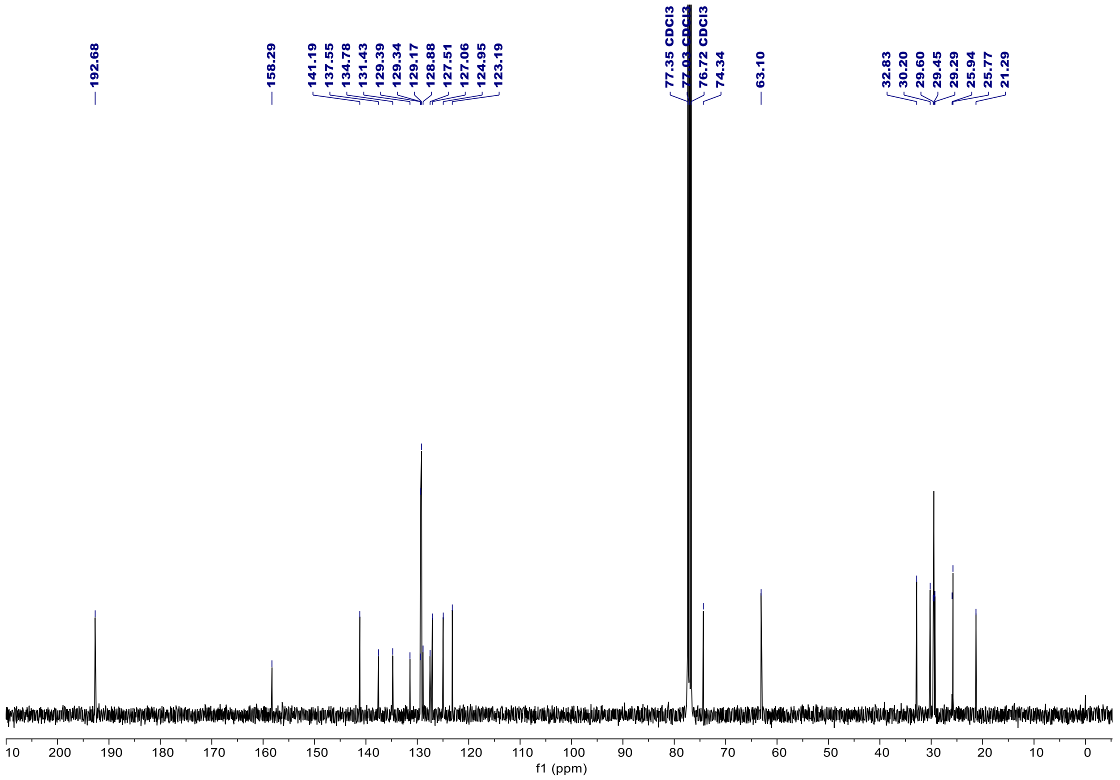

**Supplementary Figure 29.** $^{1}H$ and $^{13}C$ NMR spectra of **1g**

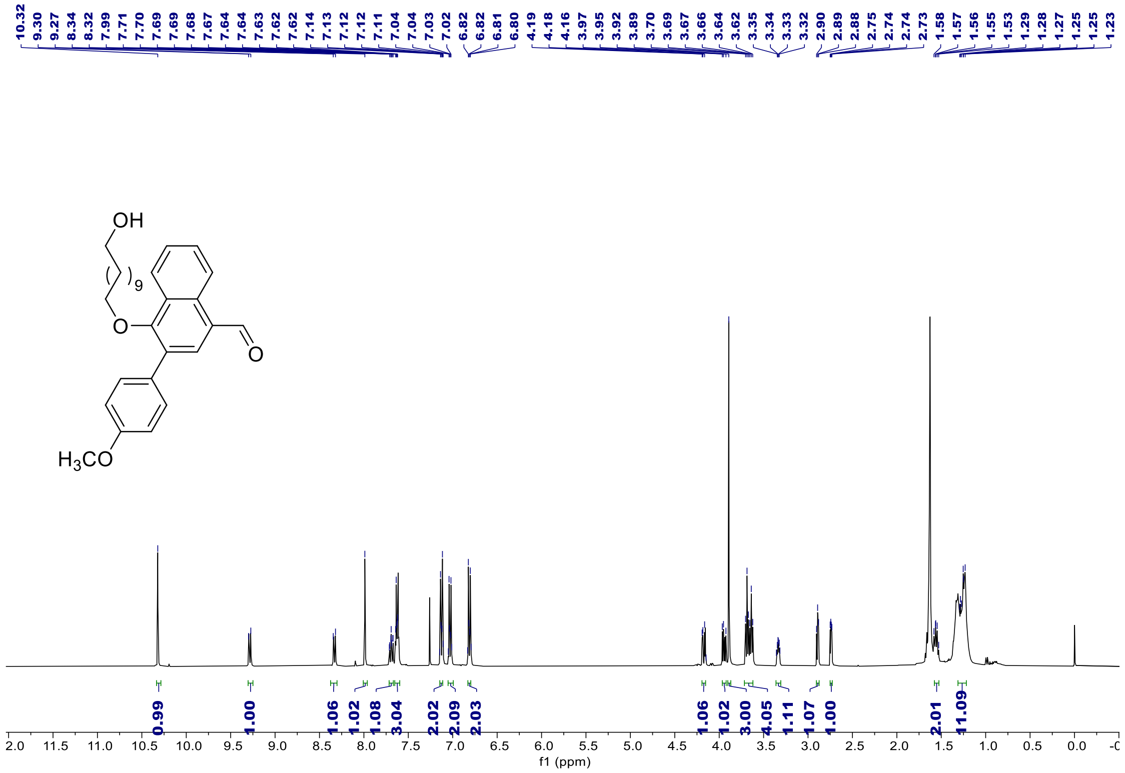


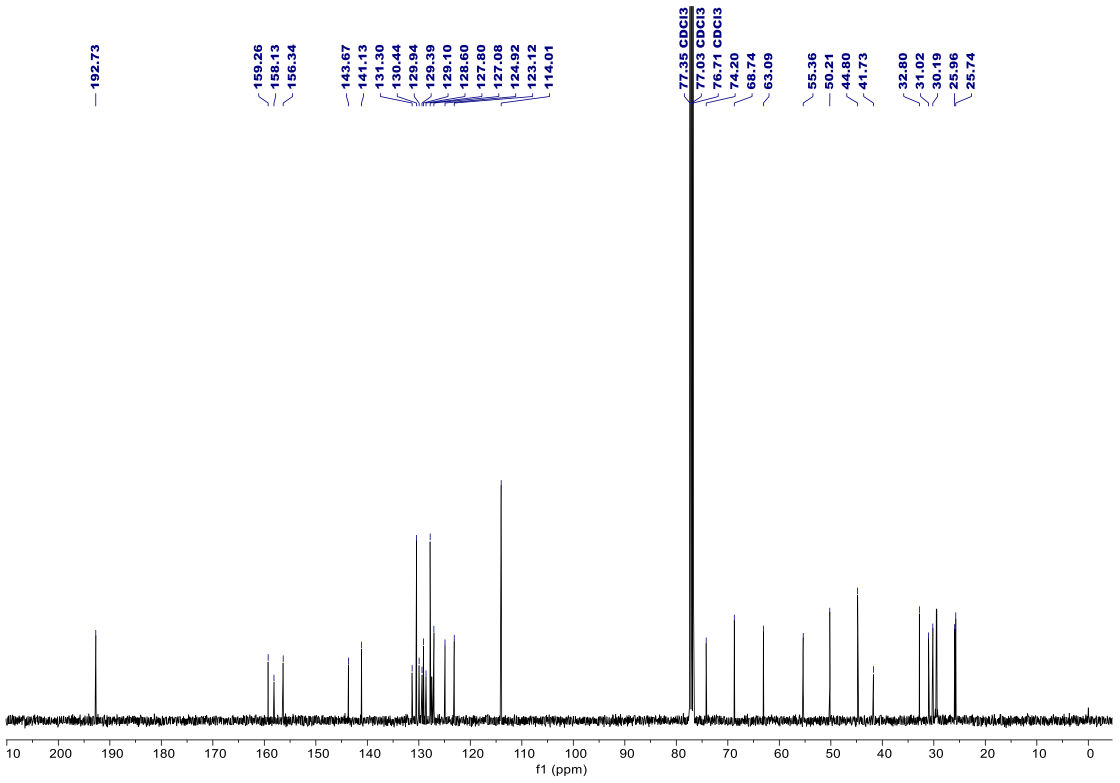

**Supplementary Figure 30.** $^{1}H$ and $^{13}C$ NMR spectra of **1h**

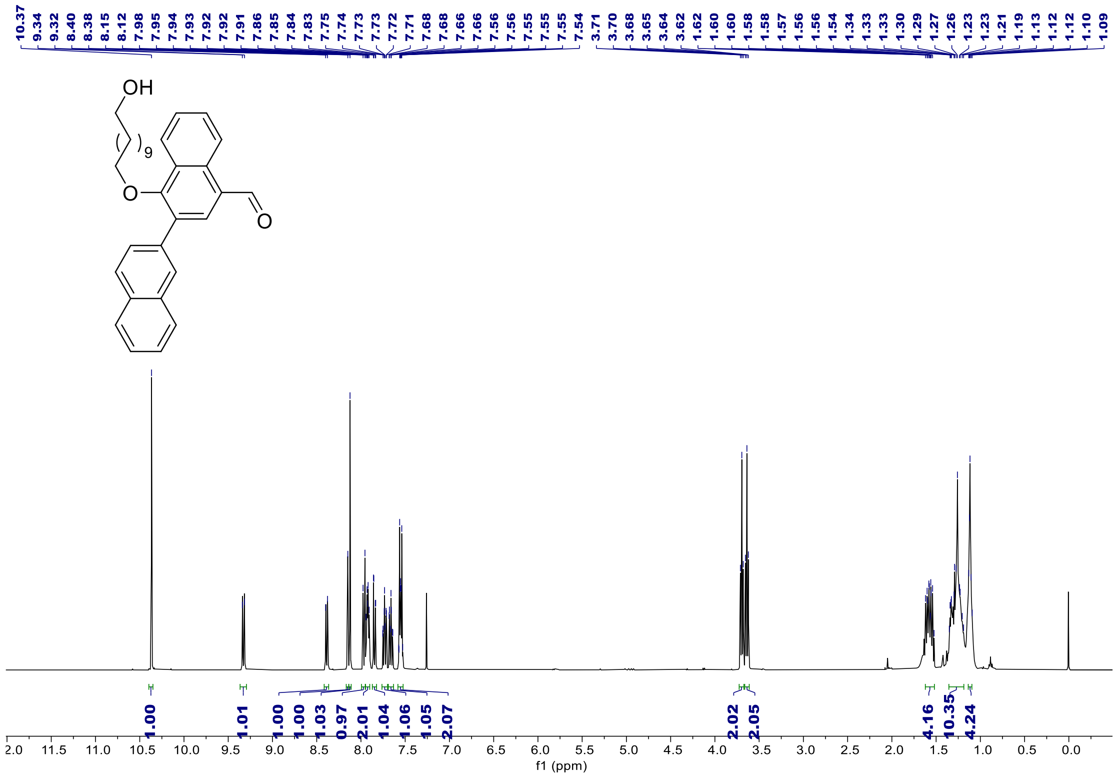

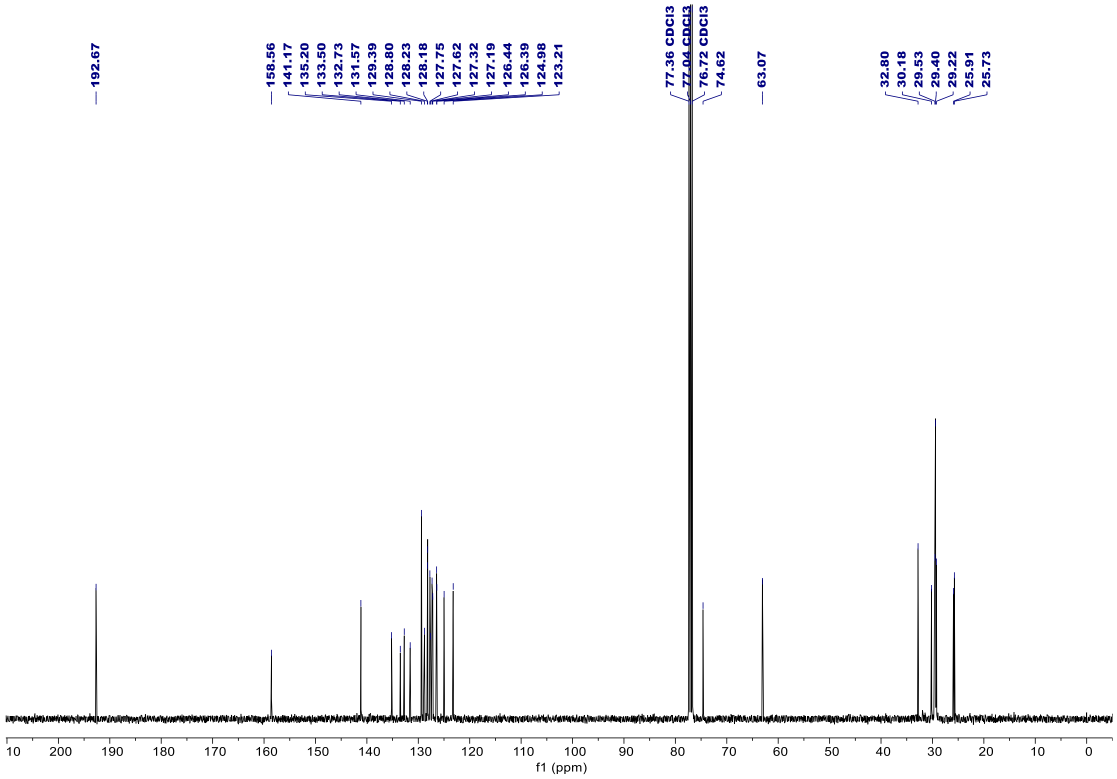

**Supplementary Figure 31.** $^{1}H$ and $^{13}C$ NMR spectra of **1i**

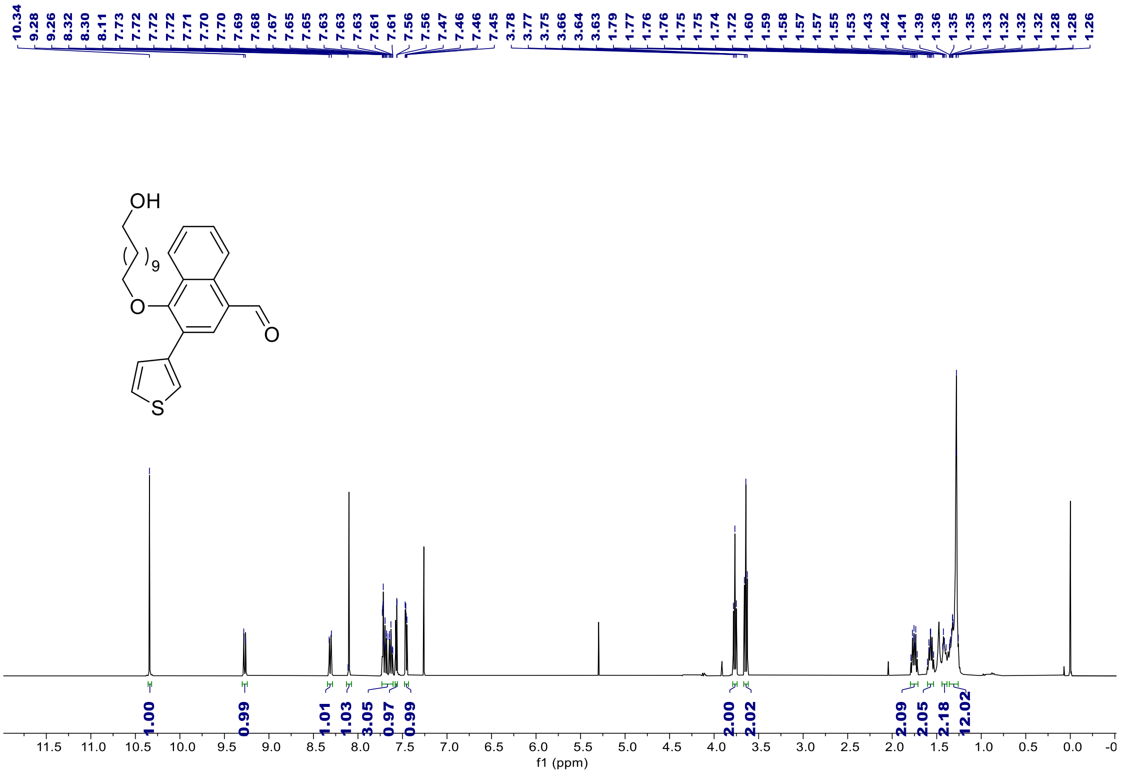

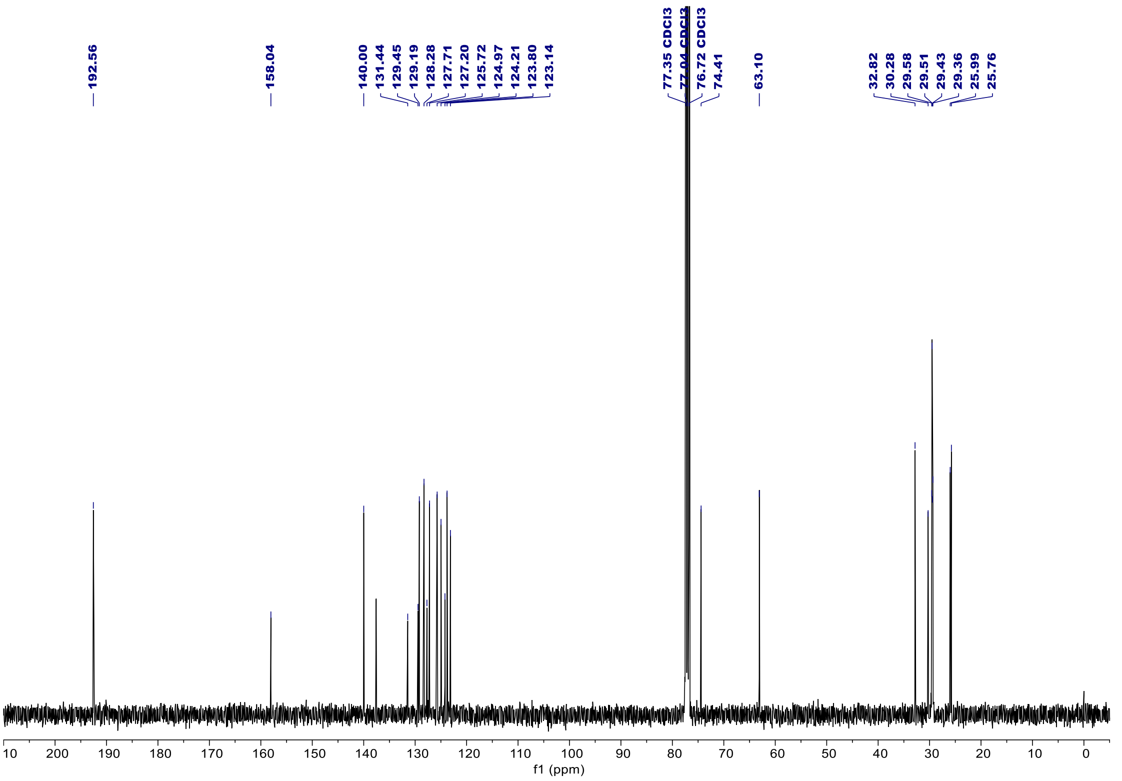

**Supplementary Figure 32.** $^{1}$H and $^{13}$C NMR spectra of **1j**

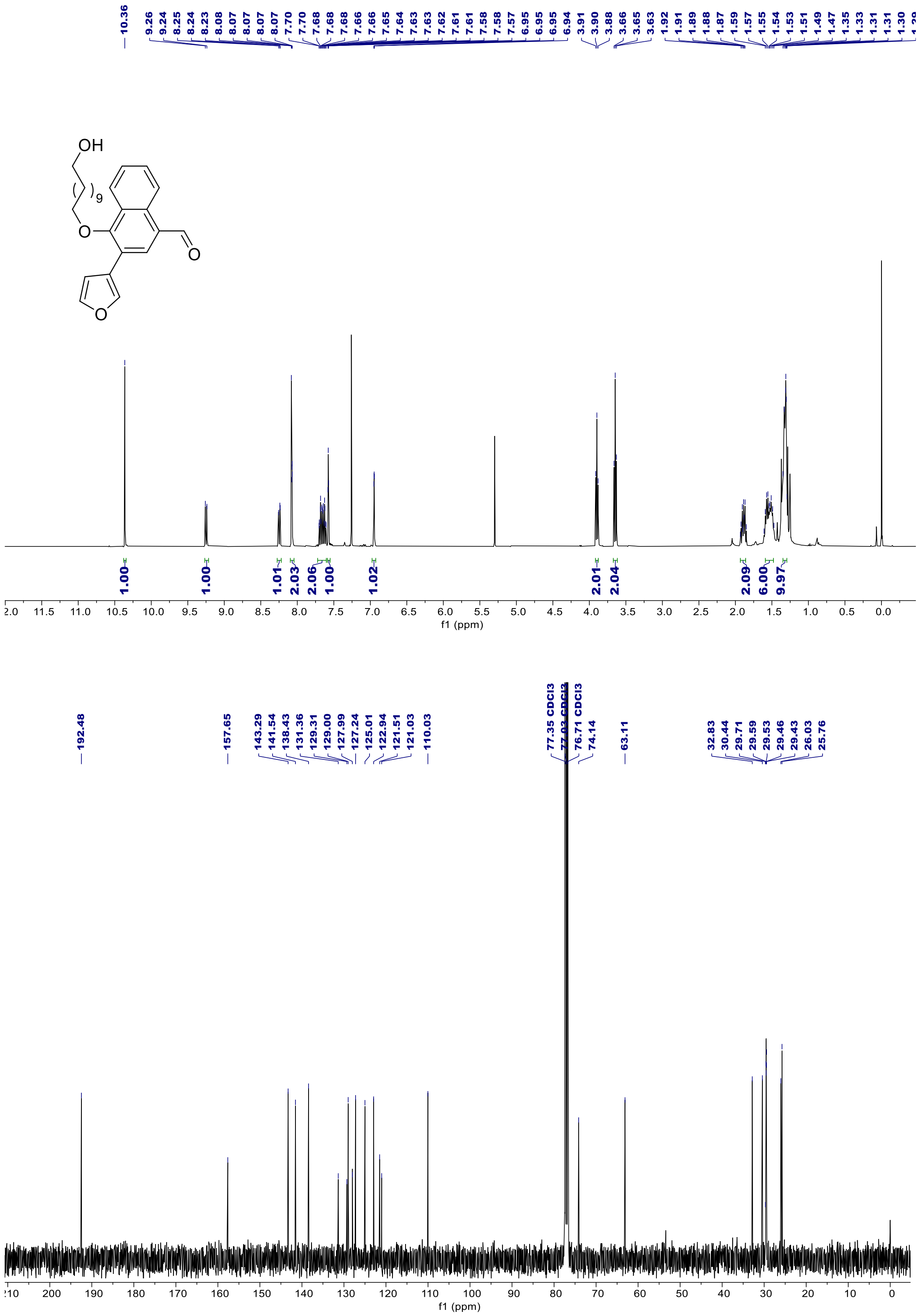

**Supplementary Figure 33.** $^{1}H$ and $^{13}C$ NMR spectra of **1k**

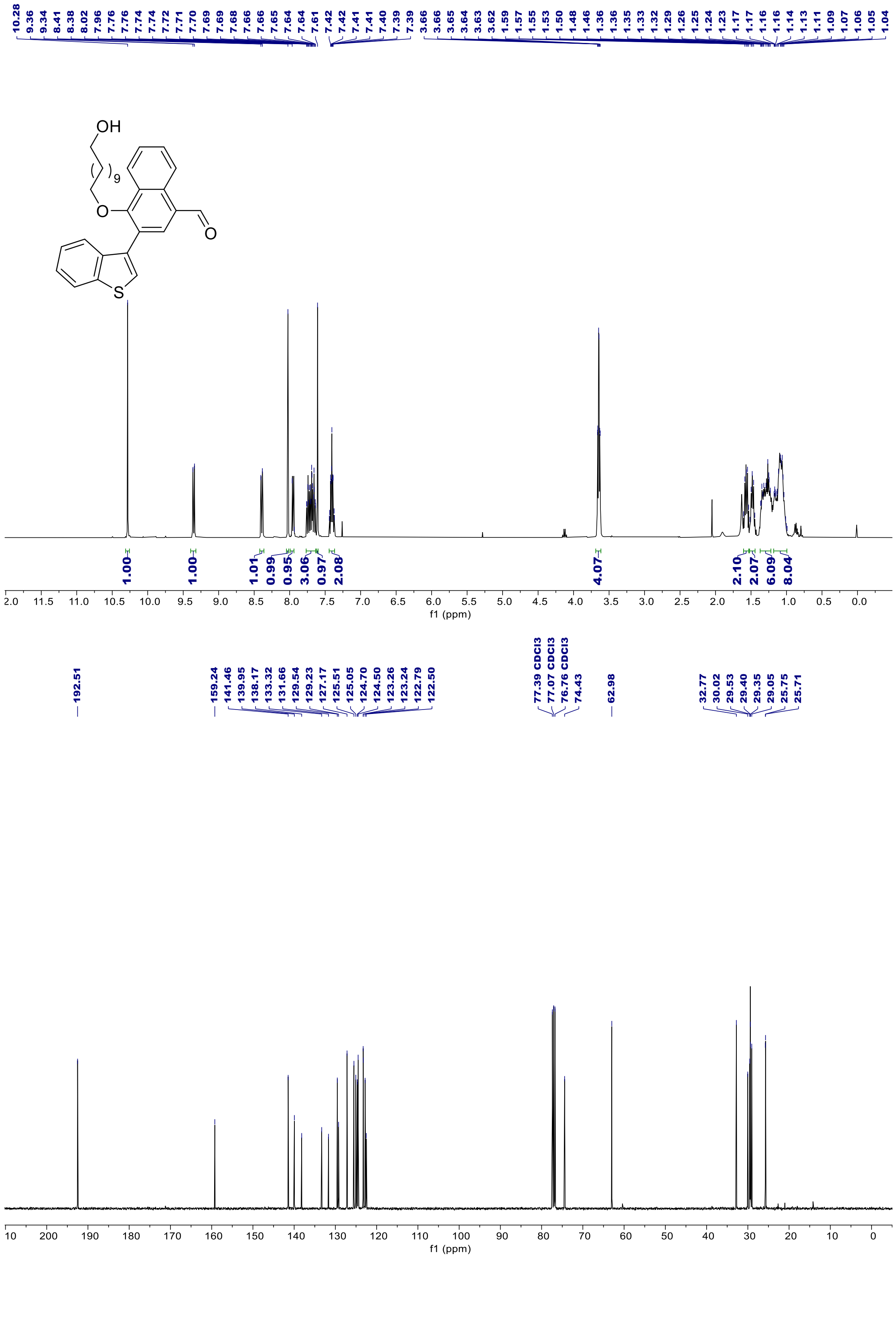

**Supplementary Figure 34.** $^1$H and $^{13}$C NMR spectra of **1l**

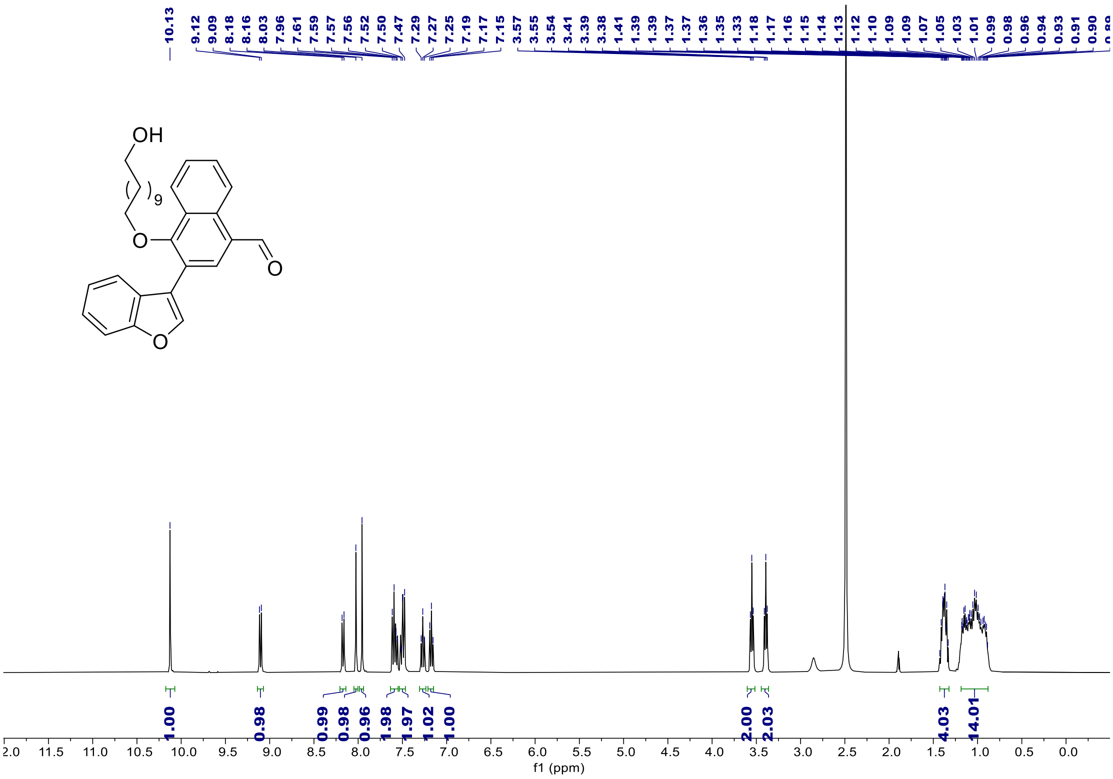


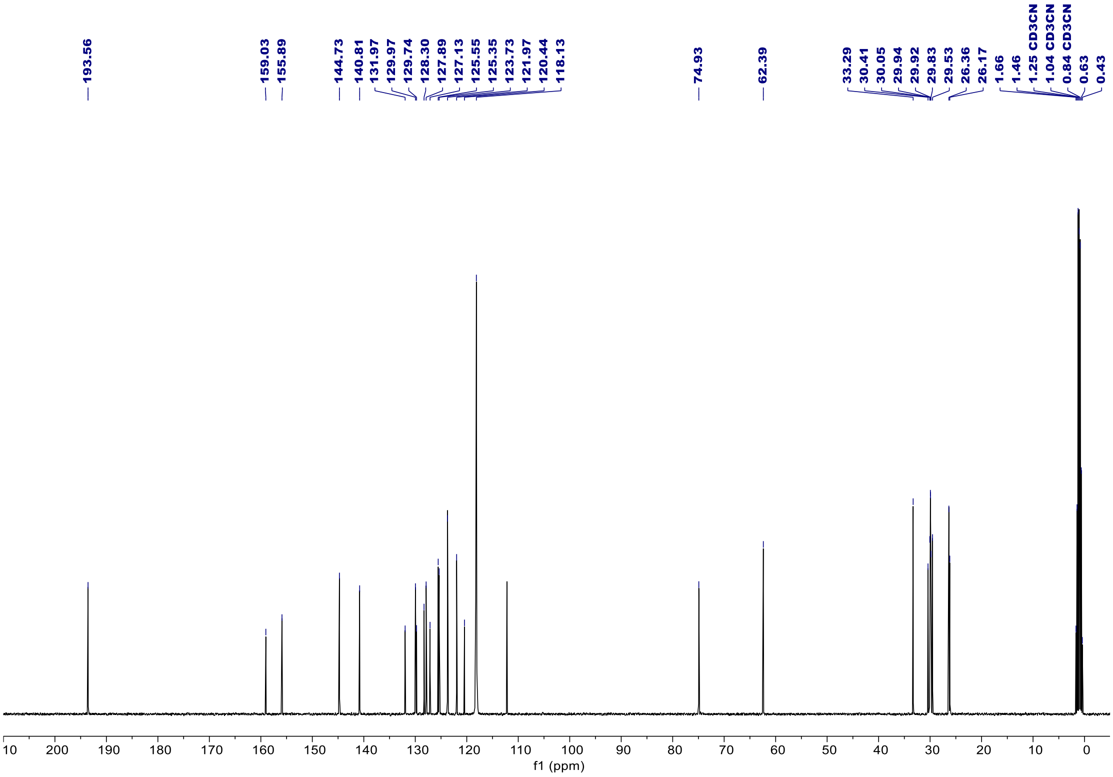

**Supplementary Figure 35.** $^{1}$H and $^{13}$C NMR spectra of **1m**

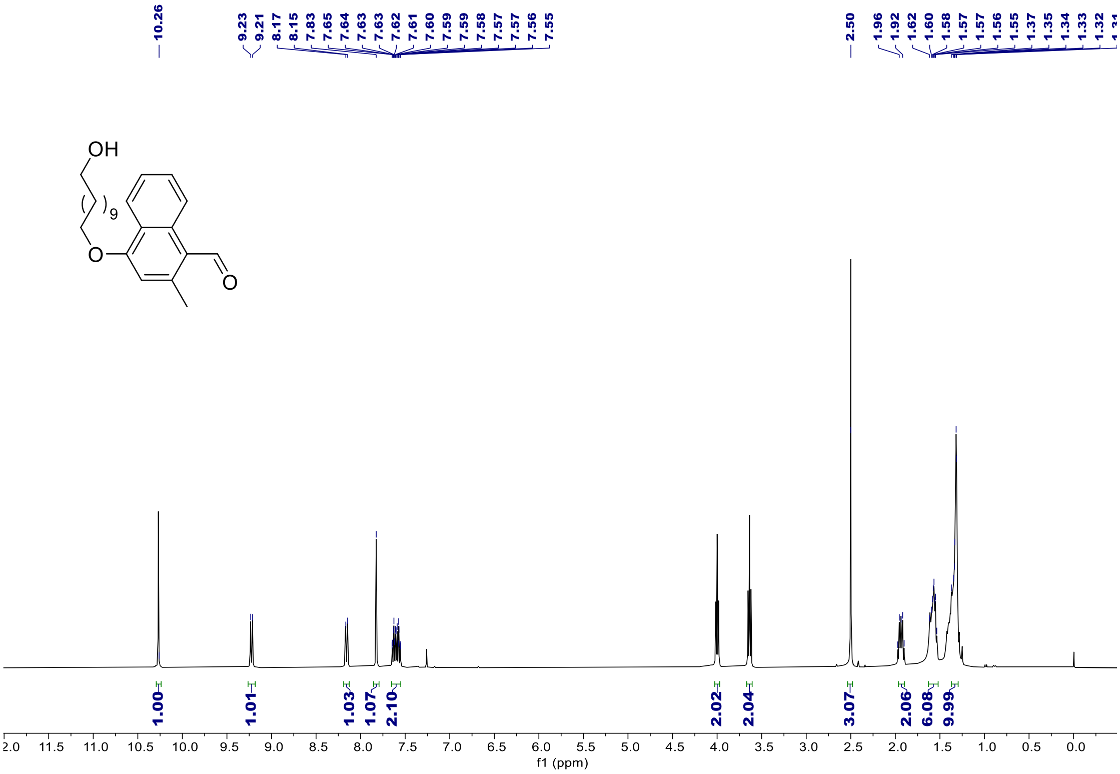


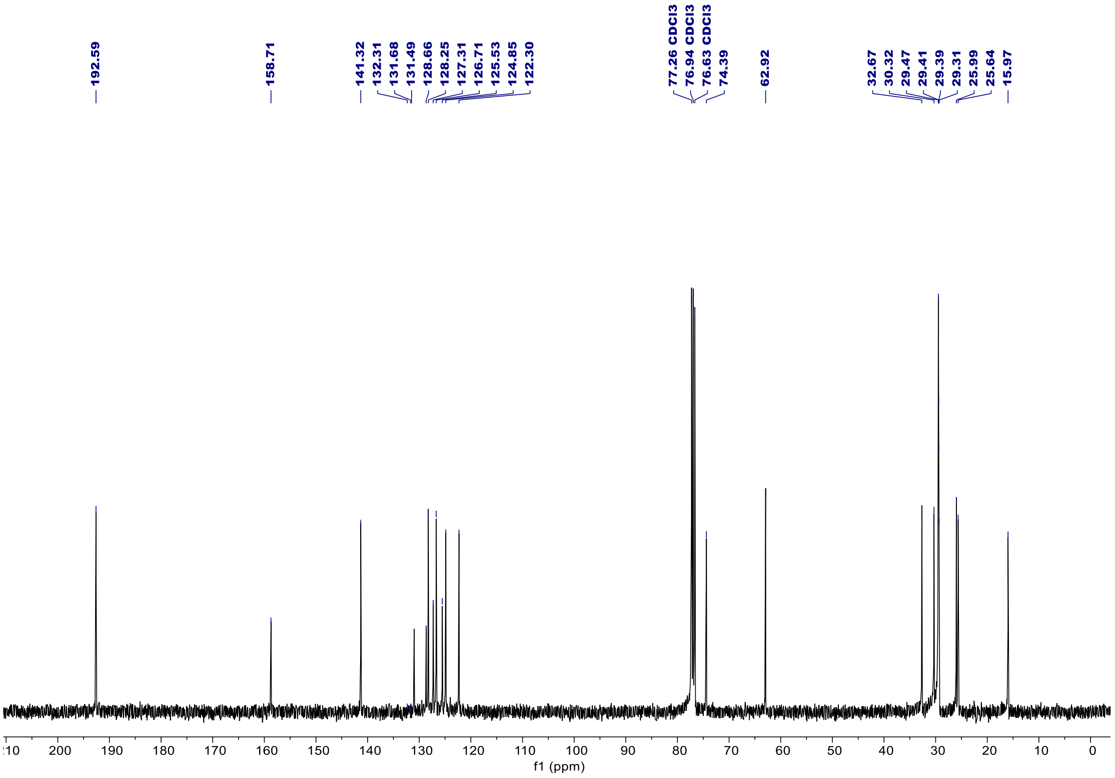

**Supplementary Figure 36.** $^{1}H$ and $^{13}C$ NMR spectra of **1n**

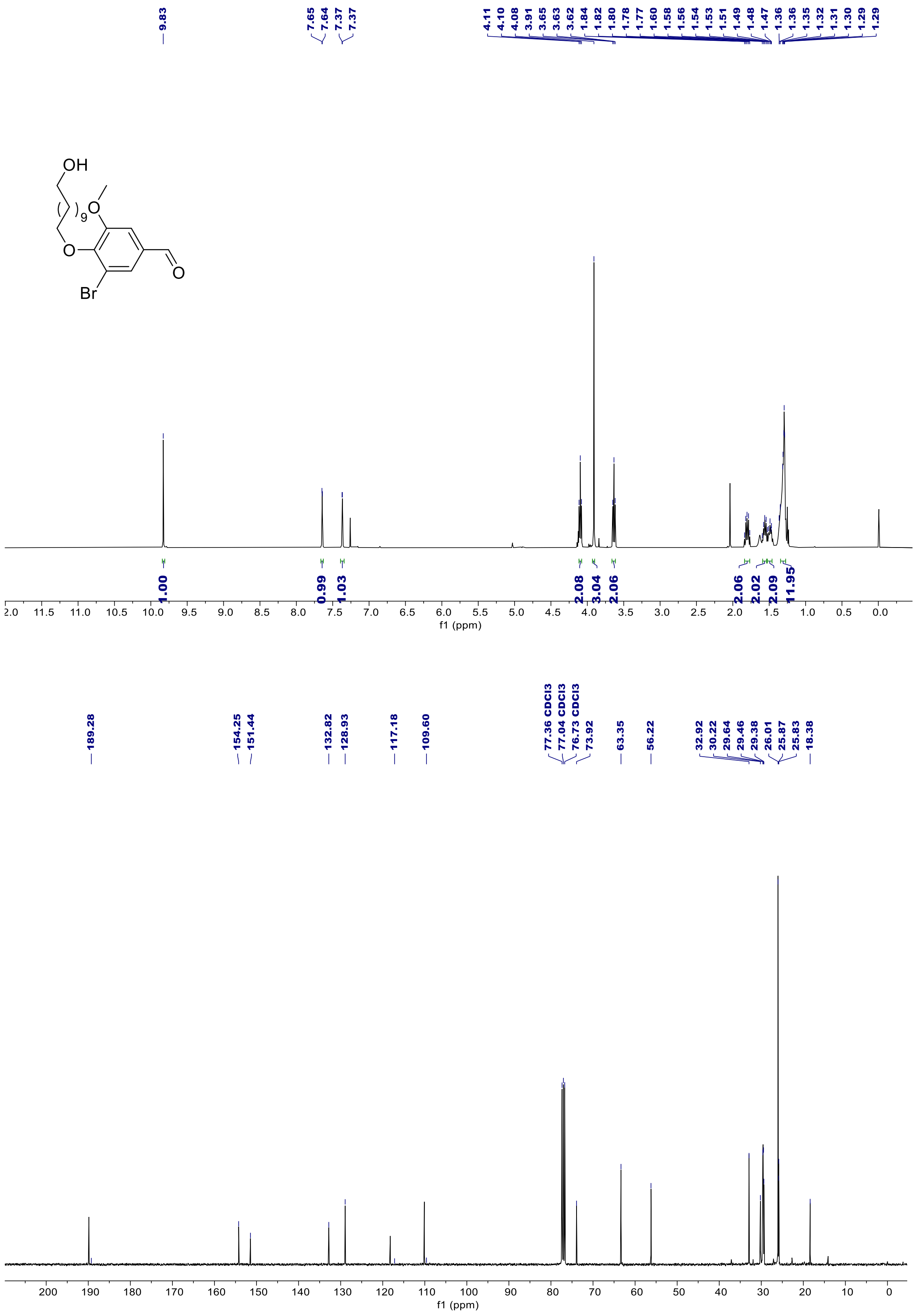

**Supplementary Figure 37.** $^{1}H$ and $^{13}C$ NMR spectra of **1o**

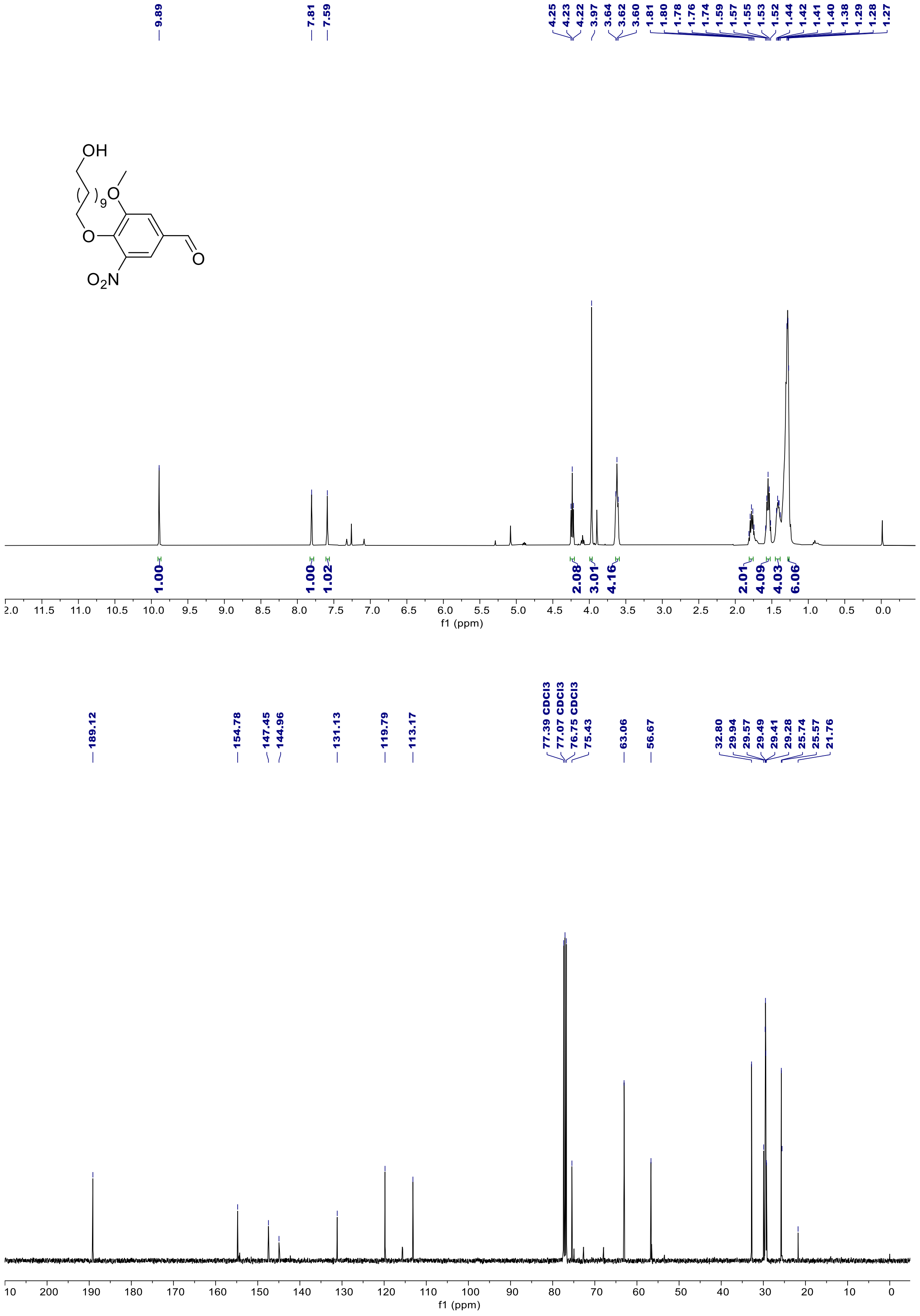

**Supplementary Figure 38.** $^{1}H$ and $^{13}C$ NMR spectra of **1p**

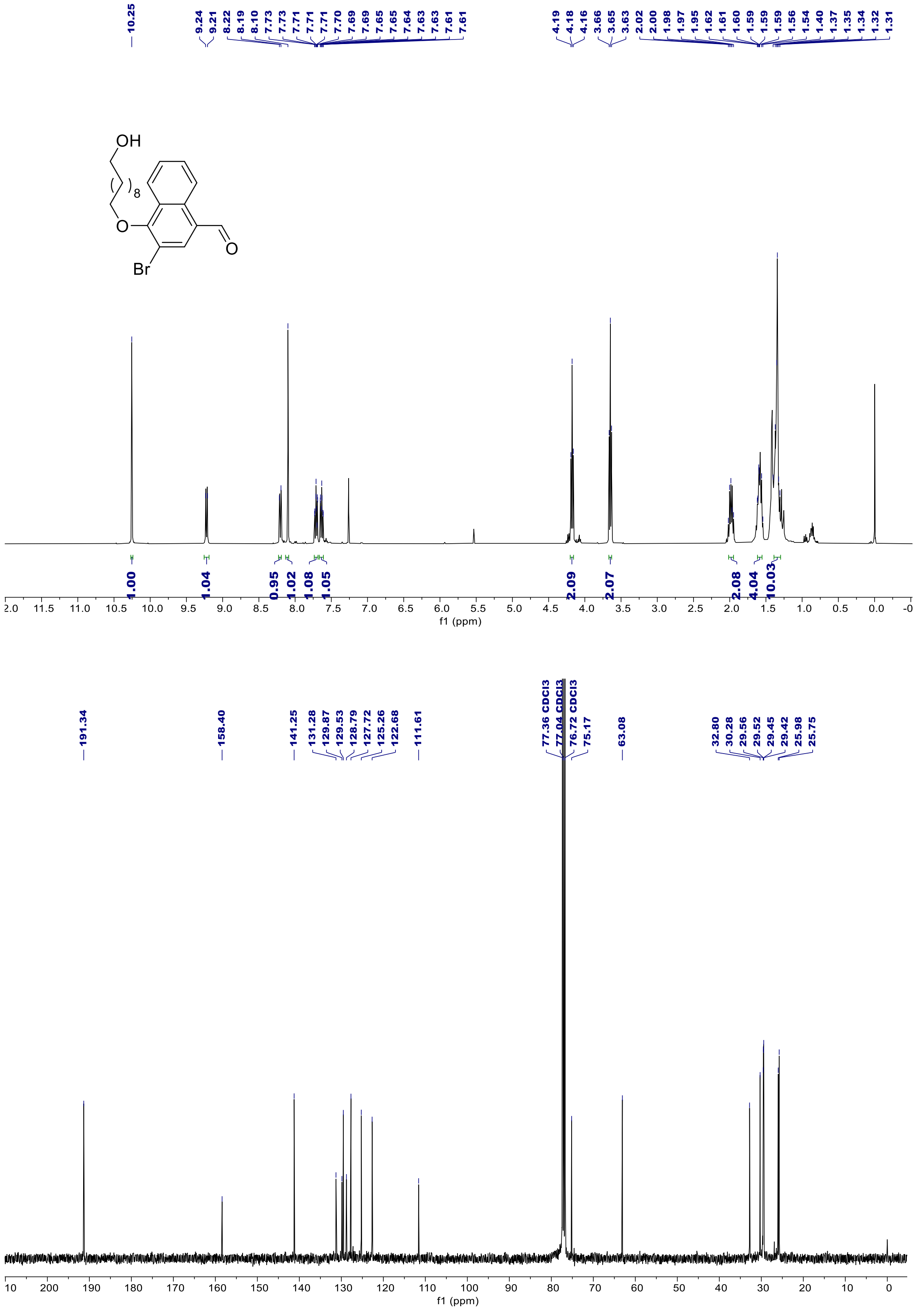

**Supplementary Figure 39.** $^{1}H$ and $^{13}C$ NMR spectra of **1q**

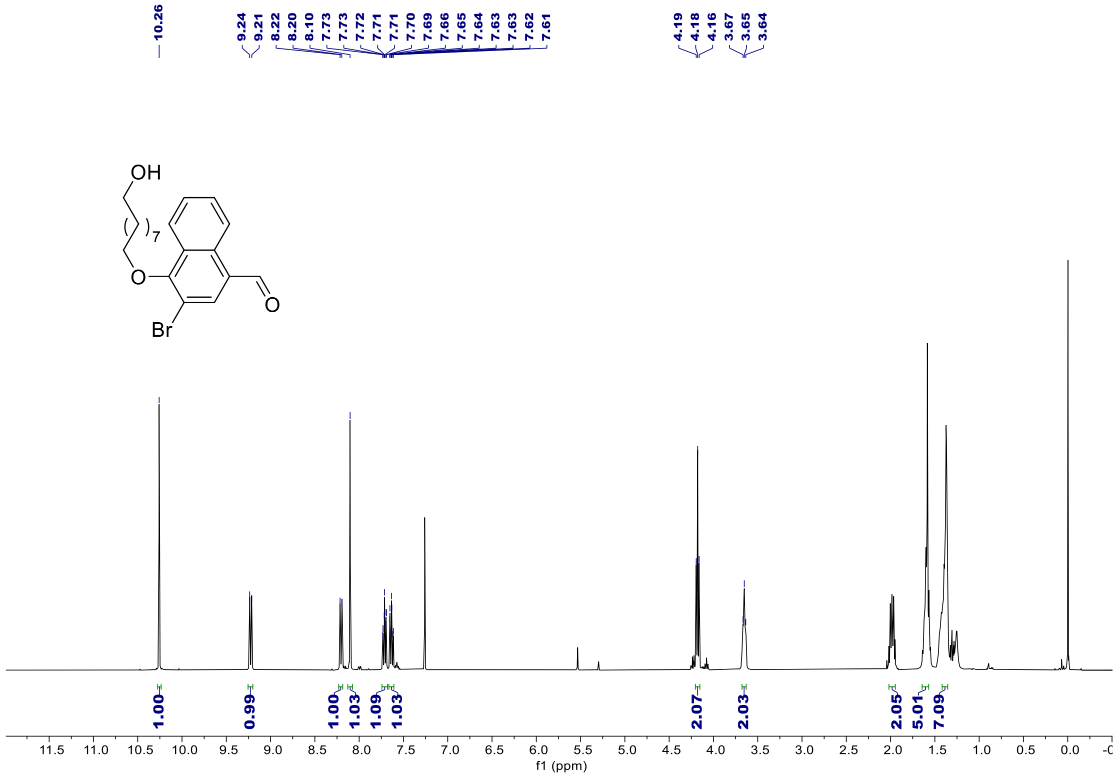

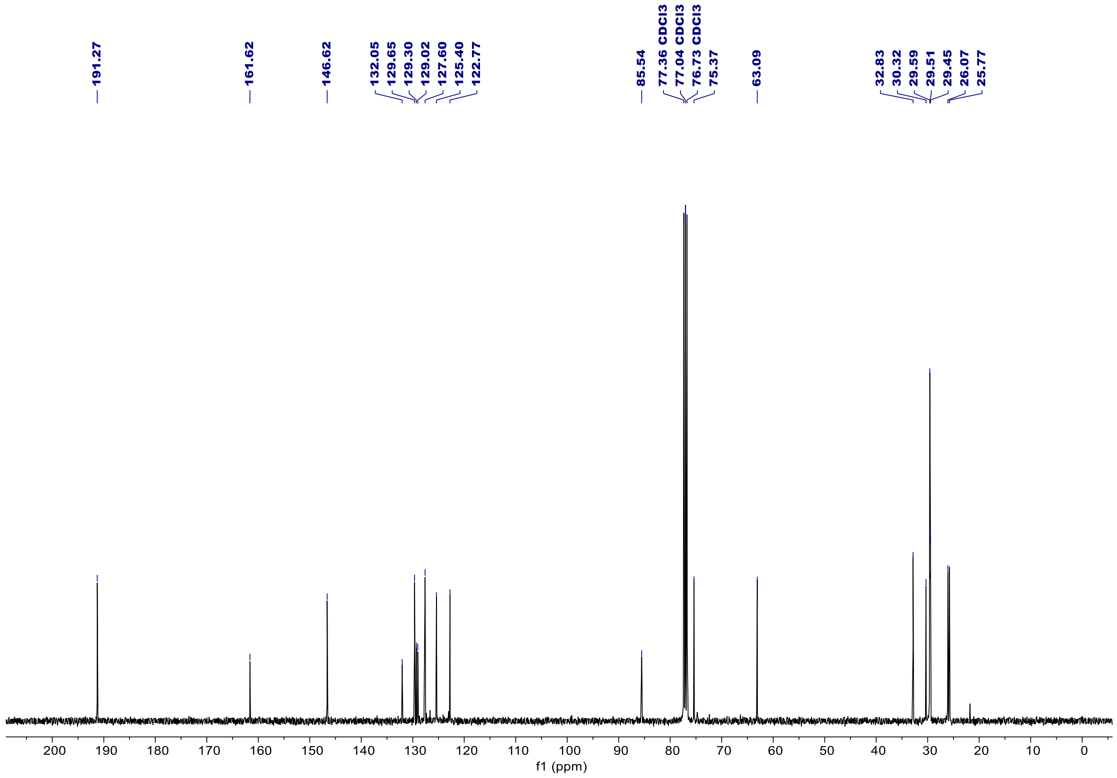

**Supplementary Figure 40.** $^{1}$H and $^{13}$C NMR spectra of **1r**

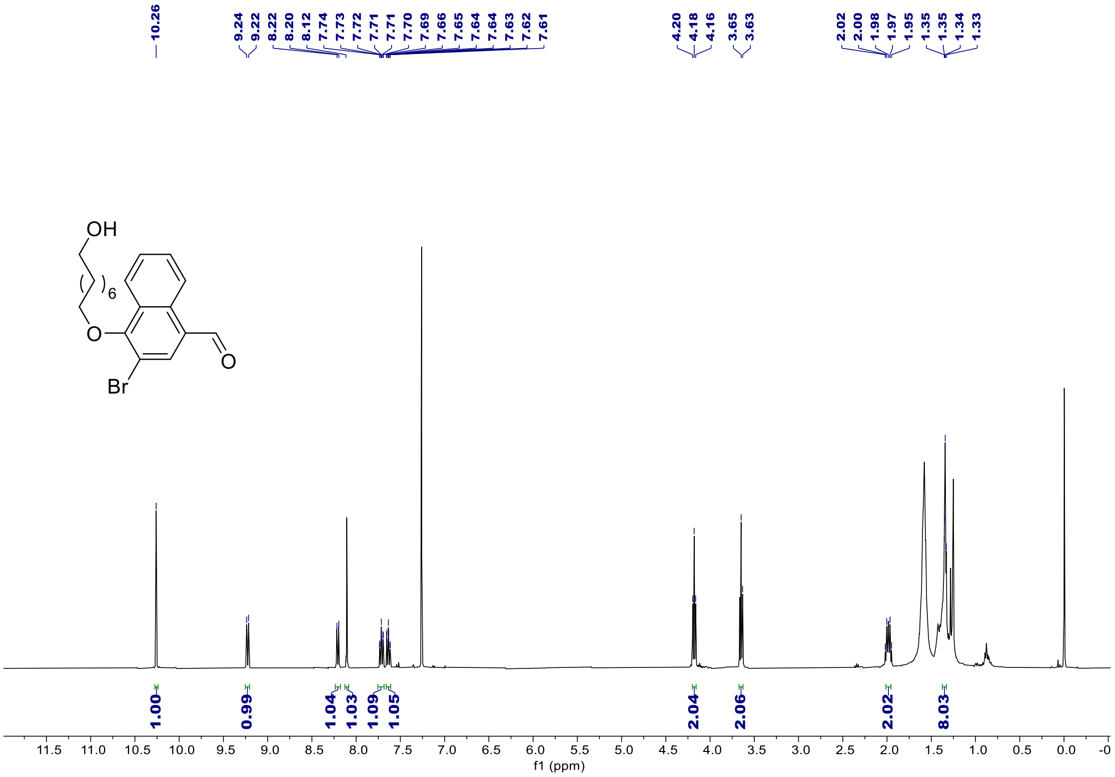

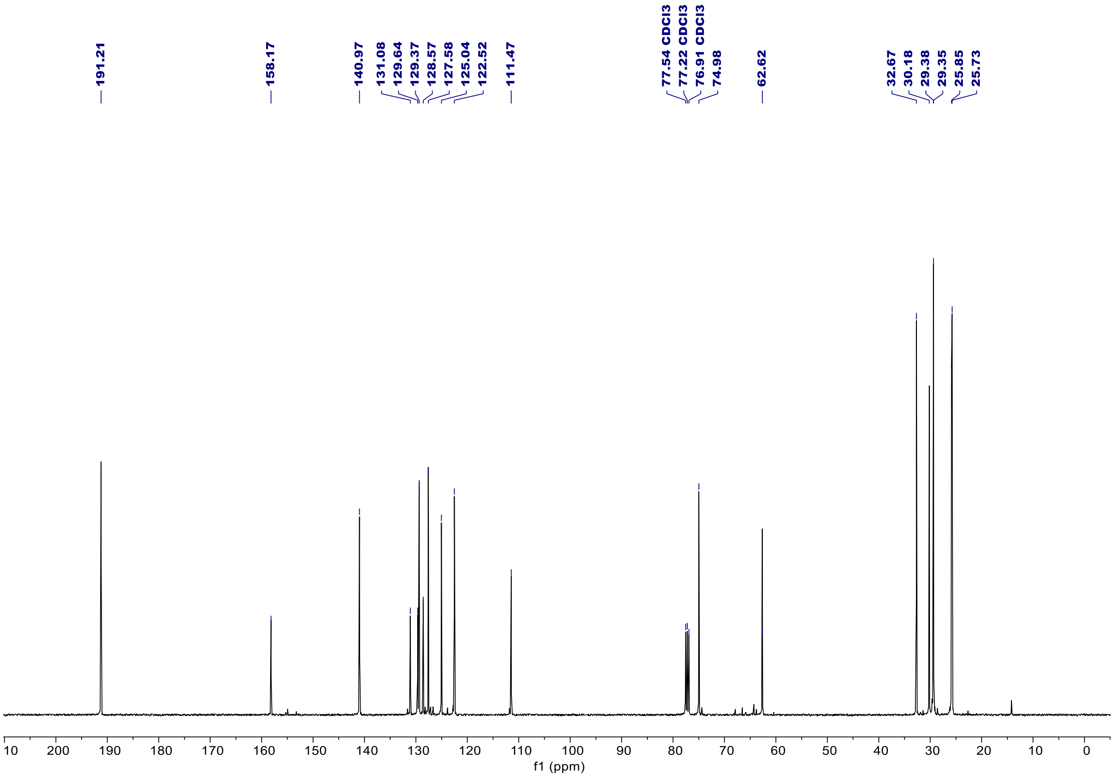

**Supplementary Figure 41.** $^{1}H$ and $^{13}C$ NMR spectra of **1s**

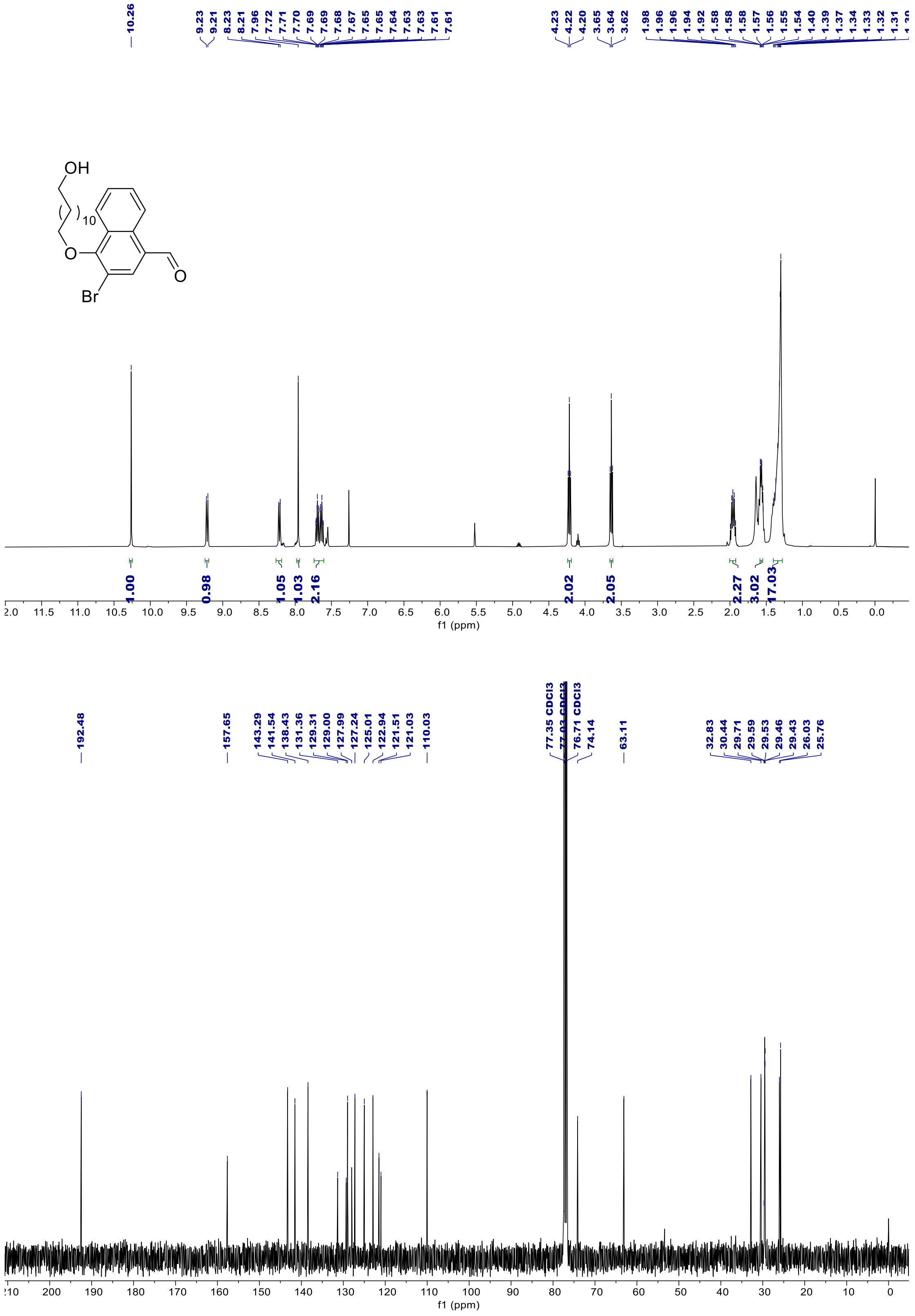

**Supplementary Figure 42.** $^{1}$H and $^{13}$C NMR spectra of **1t**

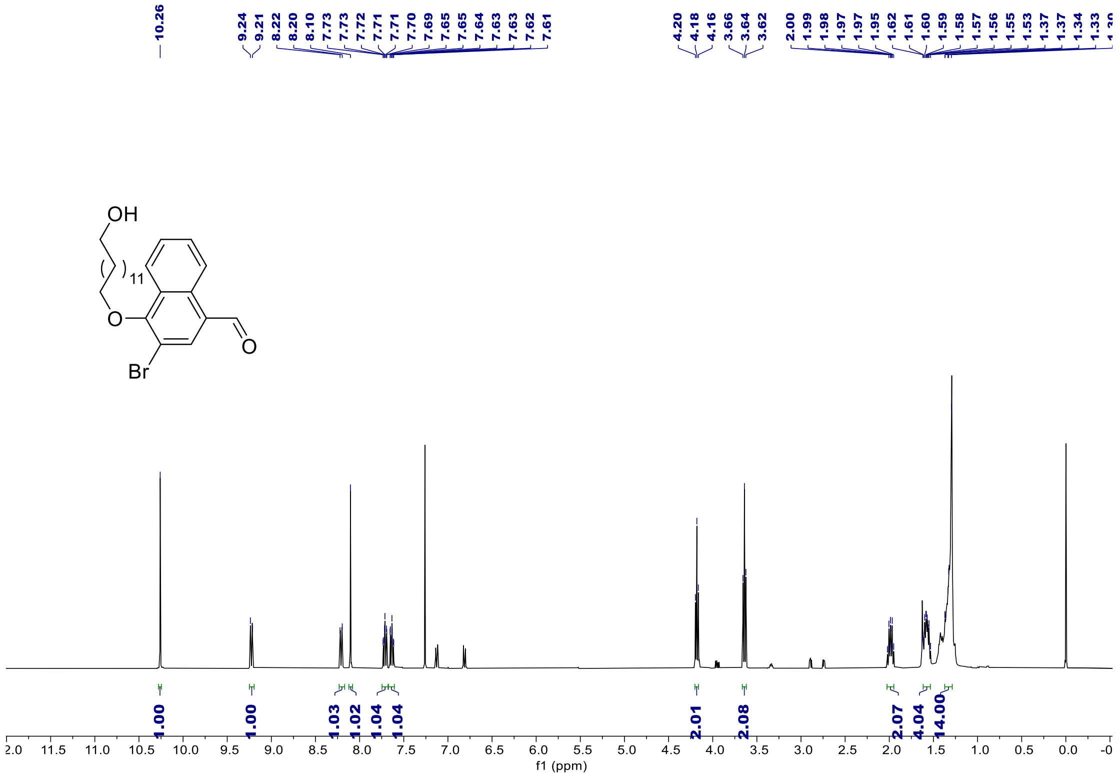


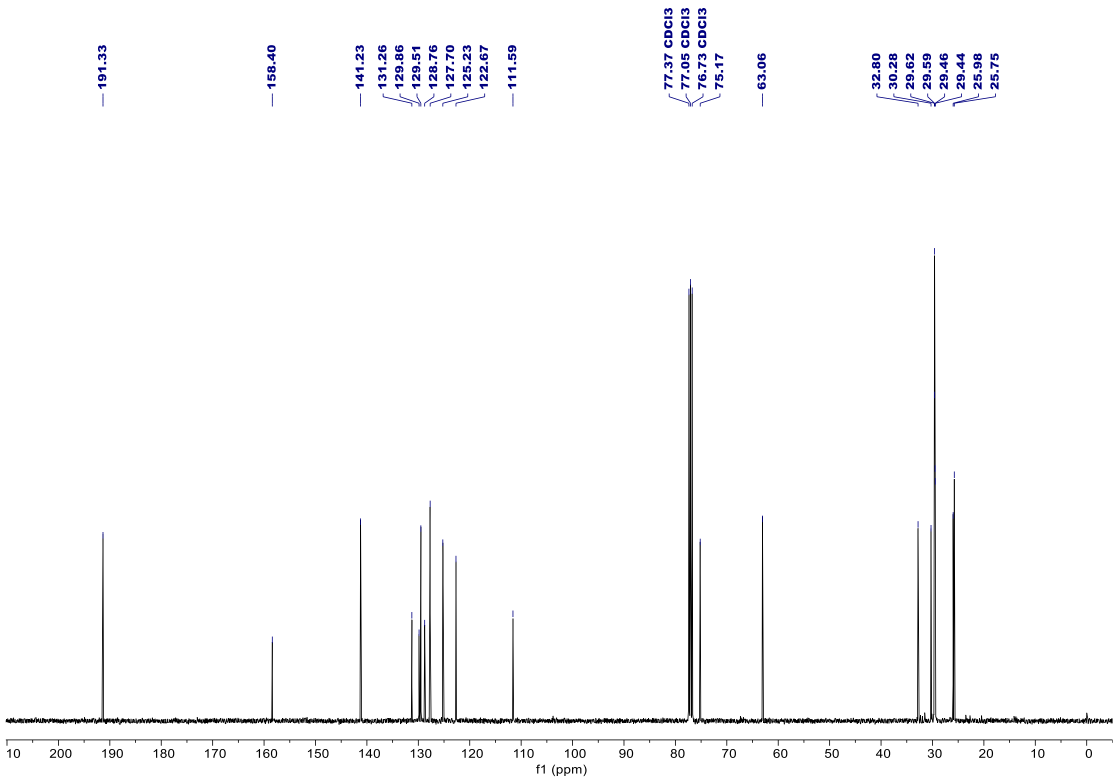

**Supplementary Figure 43.** $^{1}H$ and $^{13}C$ NMR spectra of **1u**

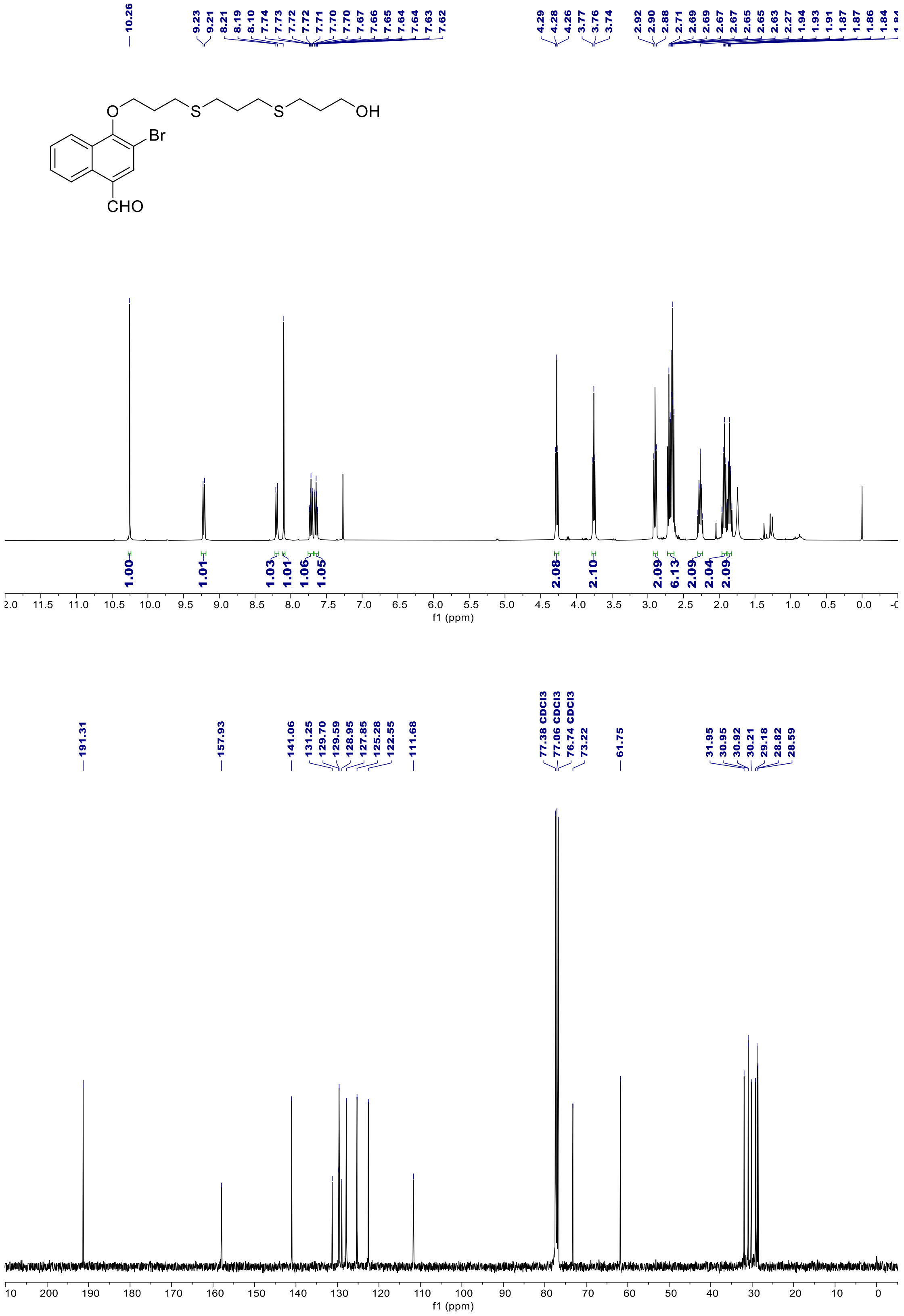

**Supplementary Figure 44.** $^{1}H$ and $^{13}C$ NMR spectra of **1v**

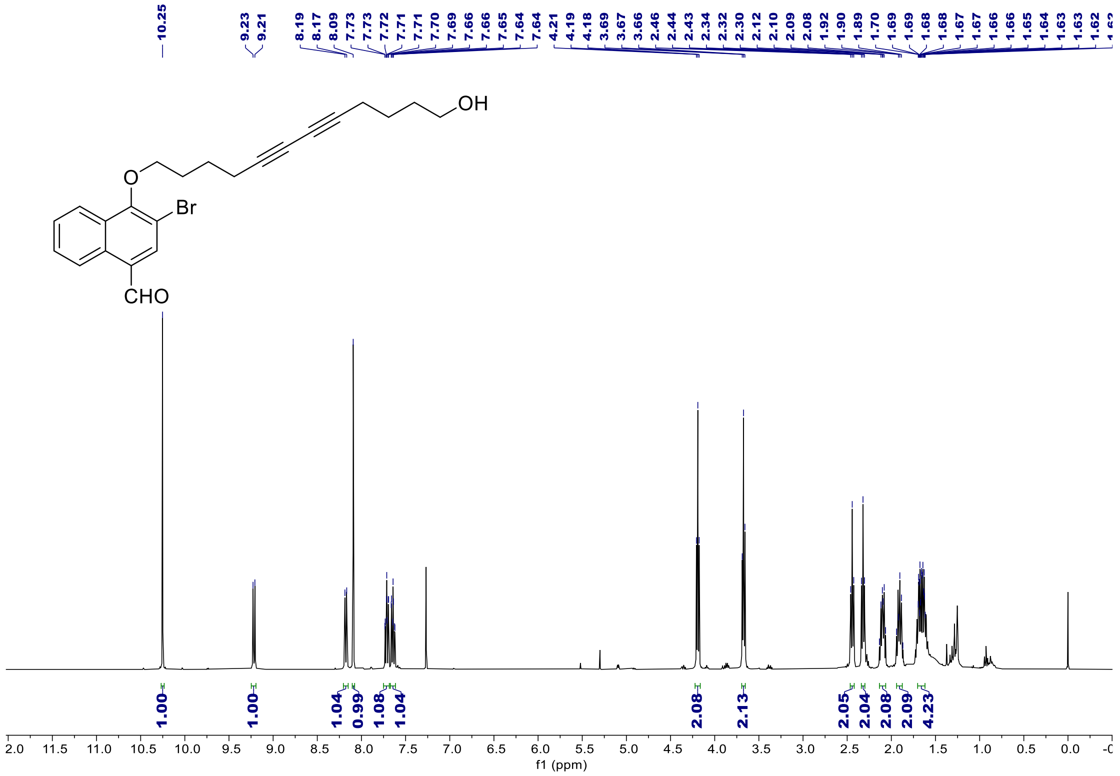


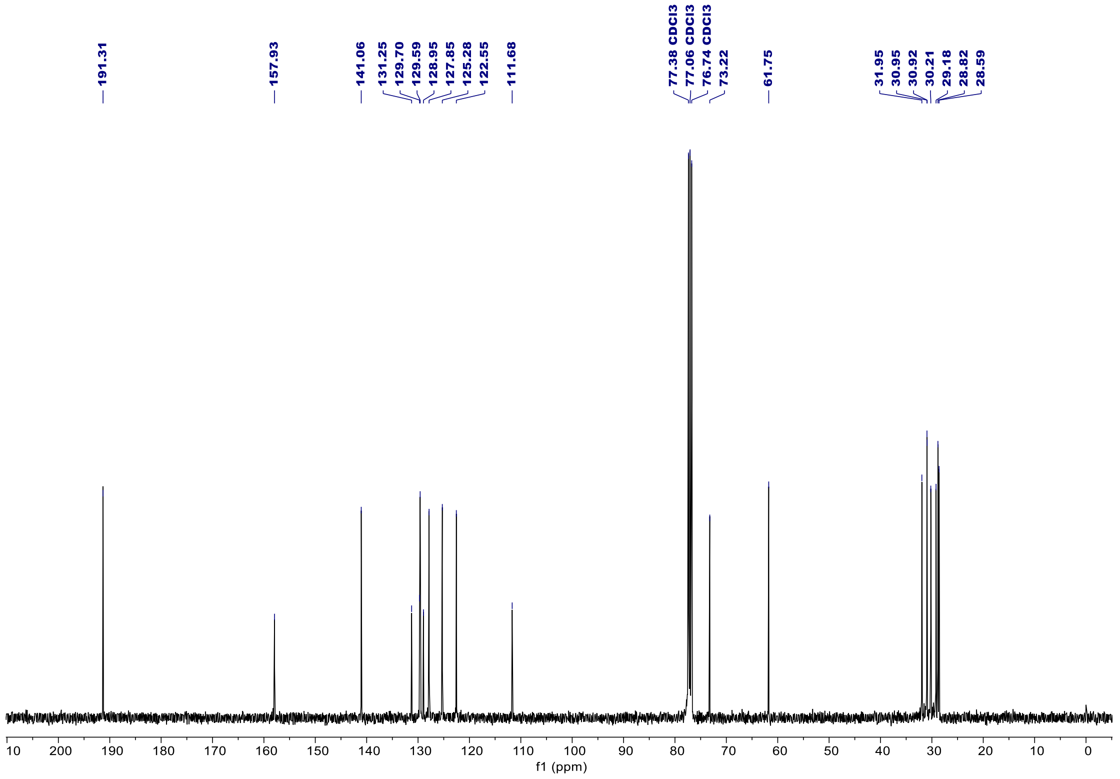

**Supplementary Figure 45.** $^{1}H$ and $^{13}C$ NMR spectra of **1w**

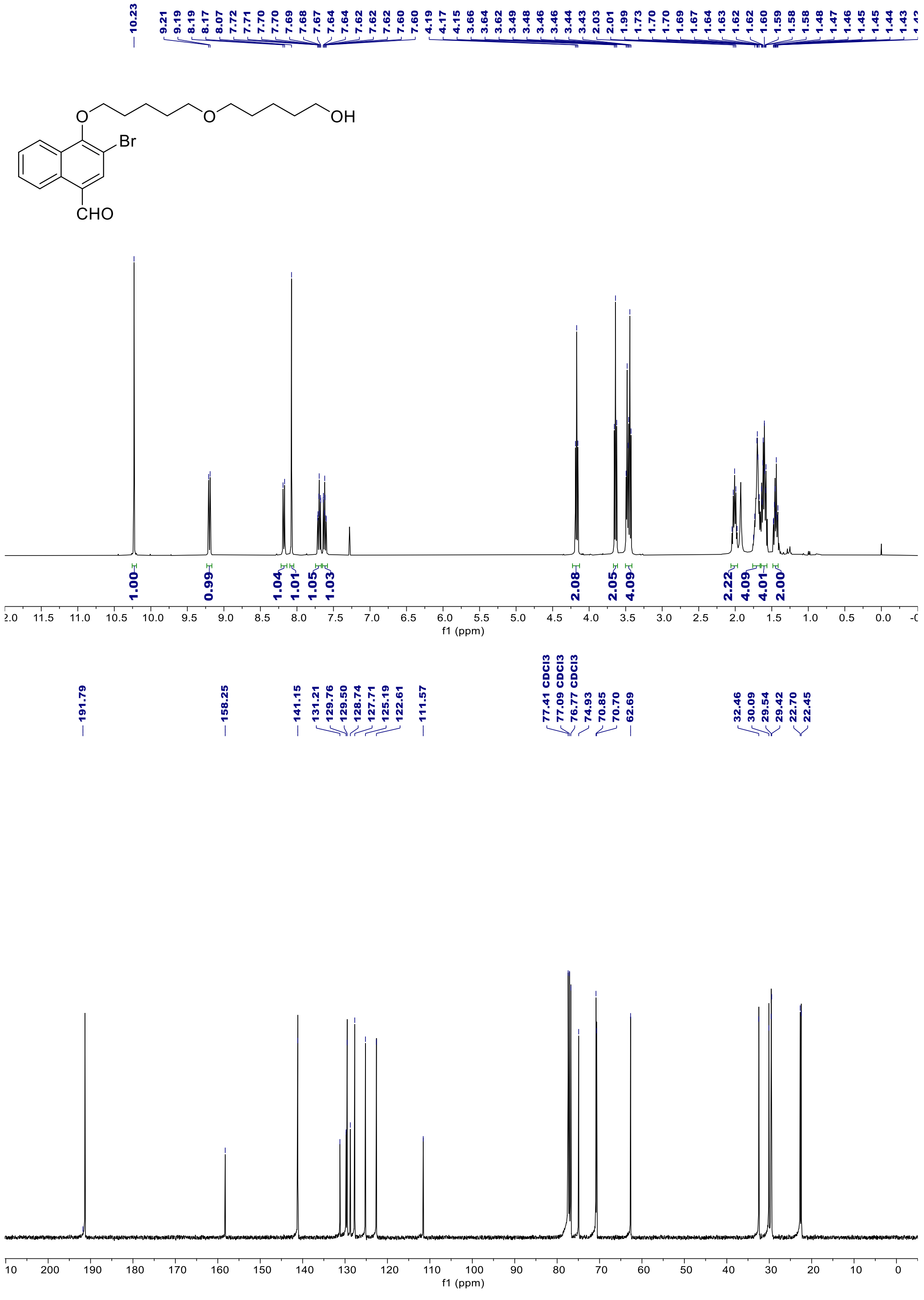

**Supplementary Figure 46.** $^{1}$H and $^{13}$C NMR spectra of HBD **3**

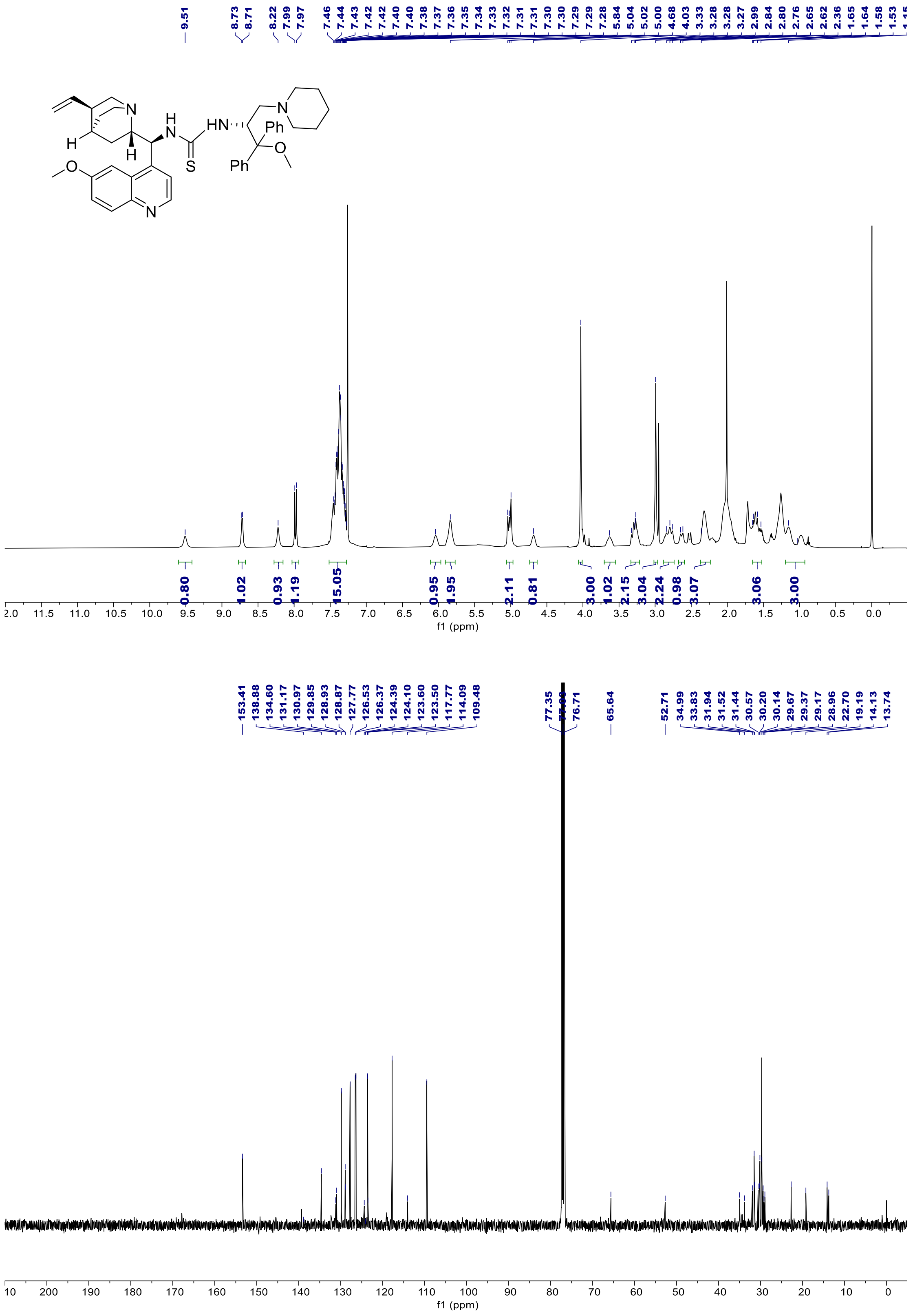

**Supplementary Figure 47.** $^{1}$H, $^{13}$C and HPLC spectra of **2a**

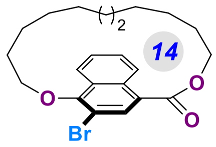


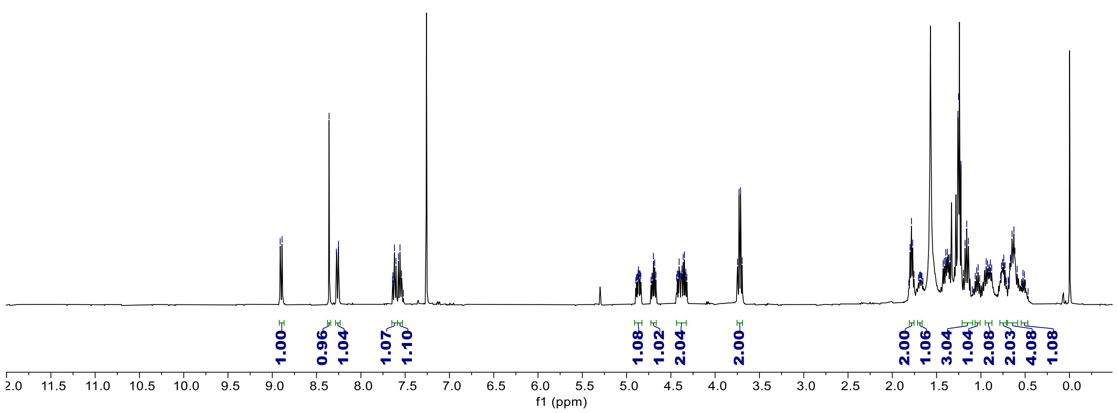


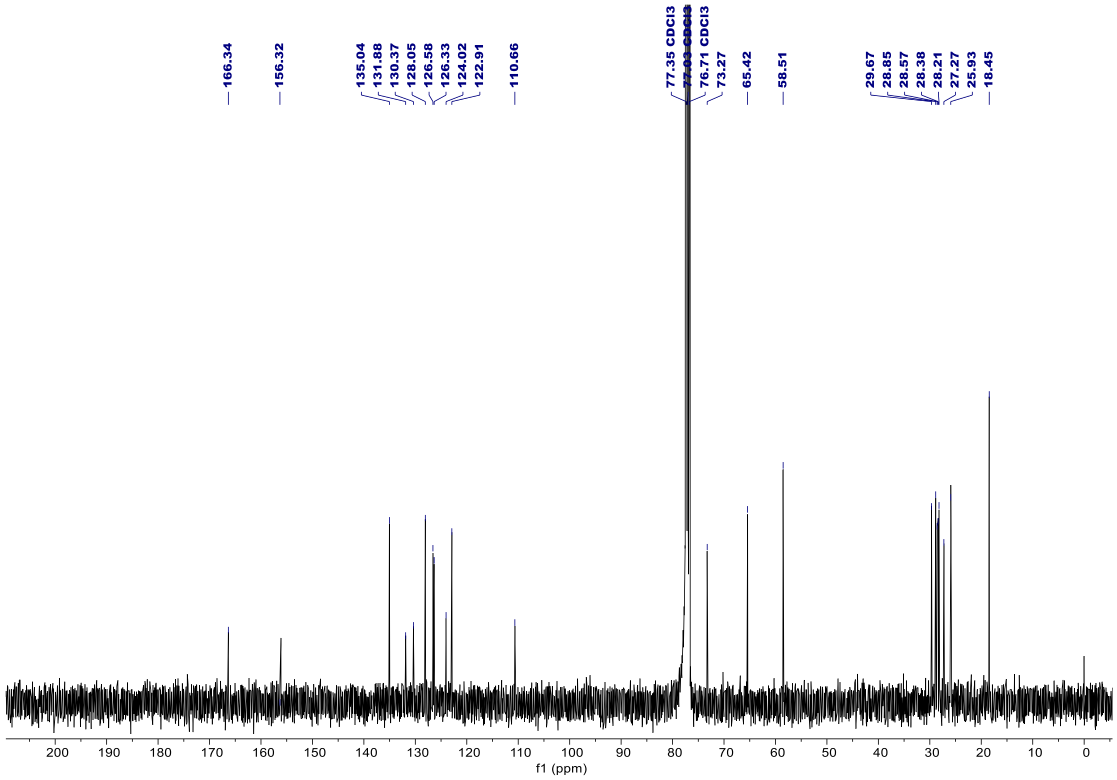

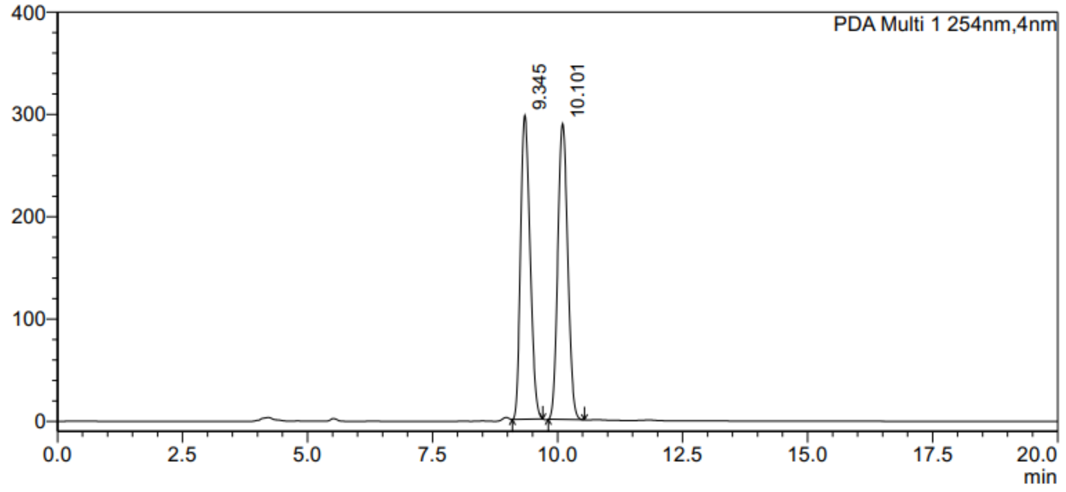


PDA Ch1 254nm

| Peak# | Ret. Time | Area | Area% | Height |
|---|---|---|---|---|
| 1 | 9.345 | 3937955 | 49.565 | 297353 |
| 2 | 10.101 | 4007086 | 50.435 | 289356 |
| Total | | 7945041 | 100.000 | 586709 |

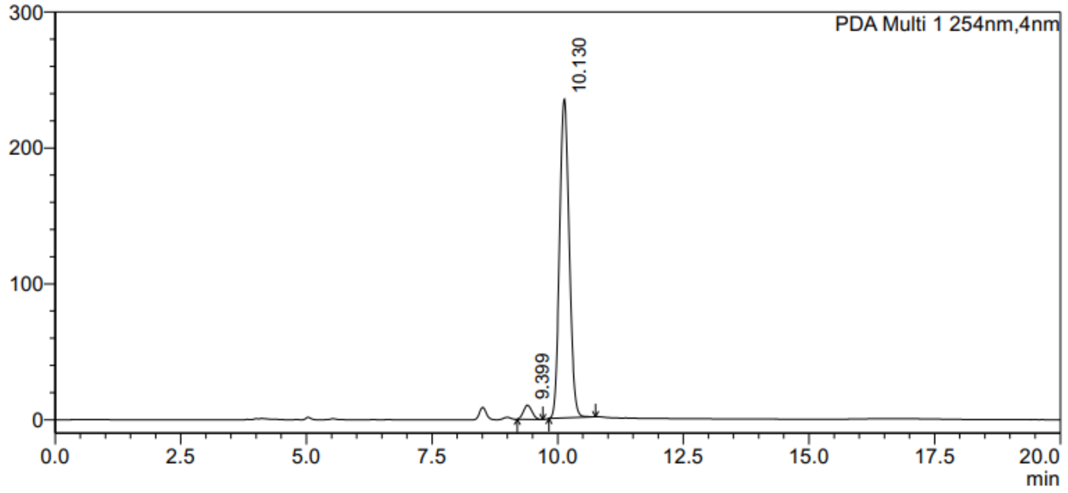


PDA Ch1 254nm

| Peak# | Ret. Time | Area | Area% | Height |
|---|---|---|---|---|
| 1 | 9.399 | 124119 | 3.813 | 10468 |
| 2 | 10.130 | 3130759 | 96.187 | 234454 |
| Total | | 3254878 | 100.000 | 244922 |

**Supplementary Figure 48.** $^{1}$H, $^{13}$C and HPLC spectra of **2b**

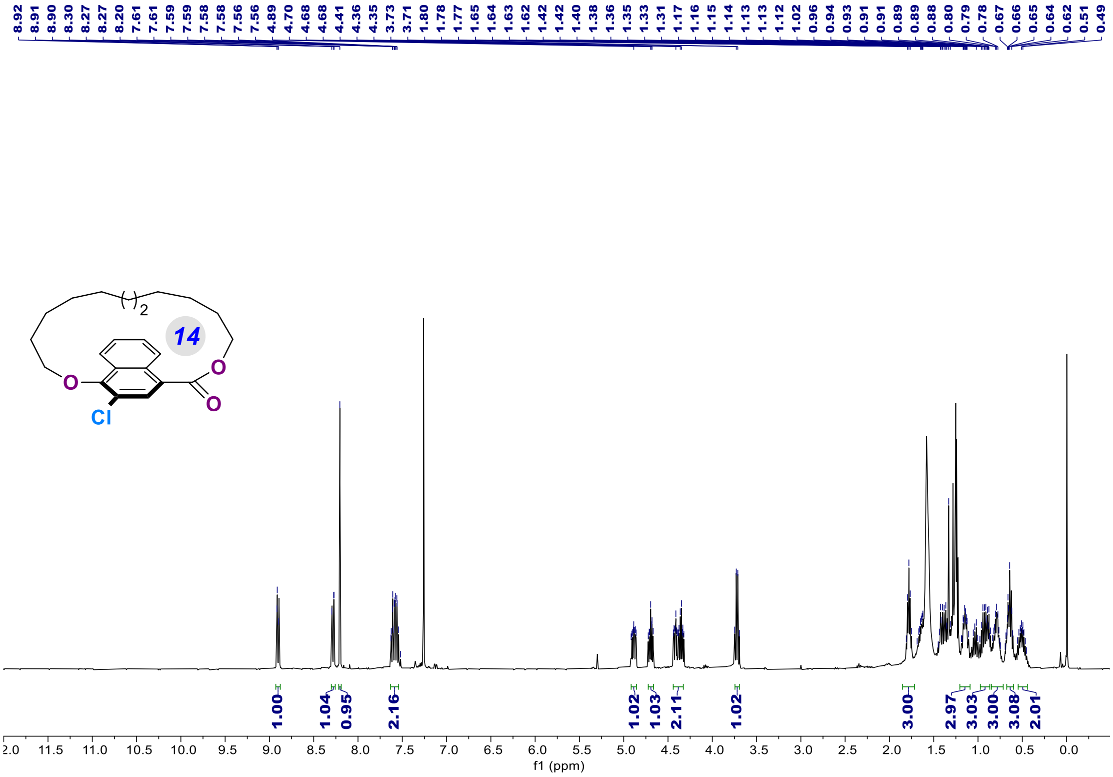

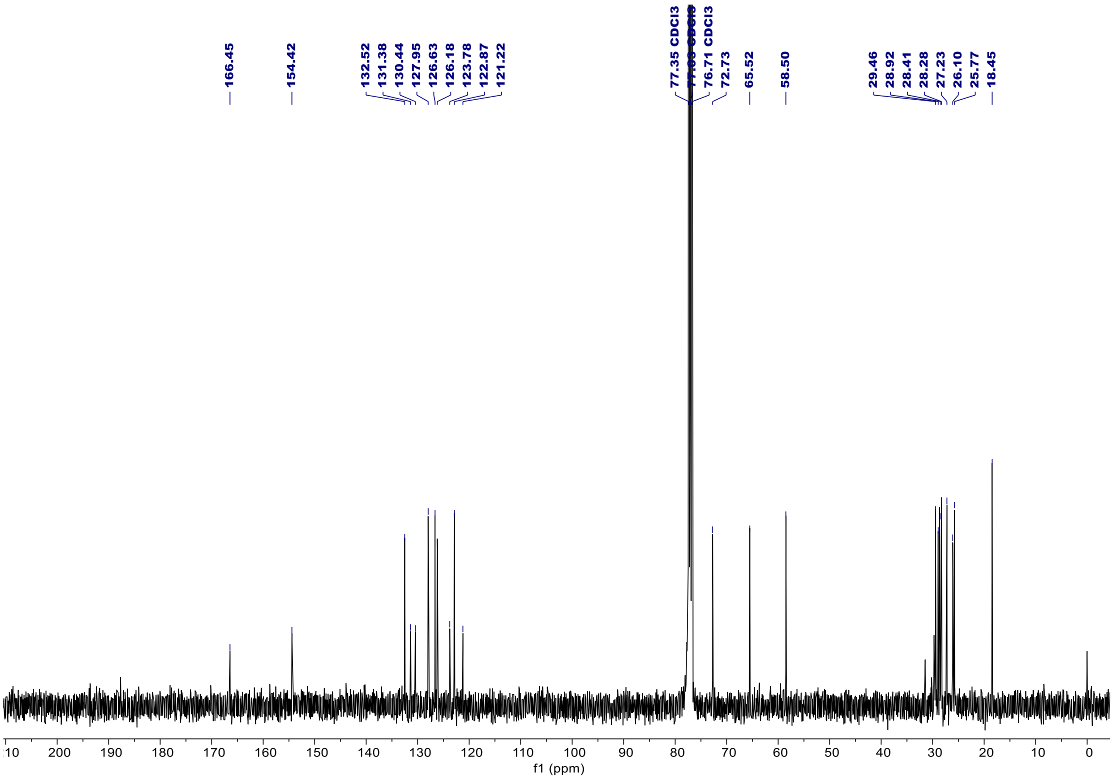

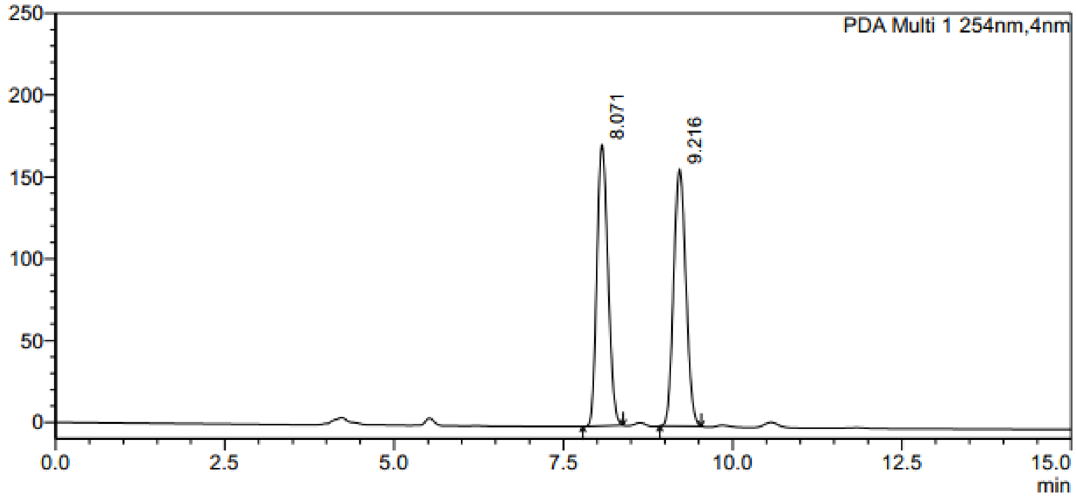


PDA Ch1 254nm

| Peak# | Ret. Time | Area | Area% | Height |
|---|---|---|---|---|
| 1 | 8.071 | 1961716 | 50.021 | 171631 |
| 2 | 9.216 | 1960055 | 49.979 | 156605 |
| Total | | 3921771 | 100.000 | 328236 |

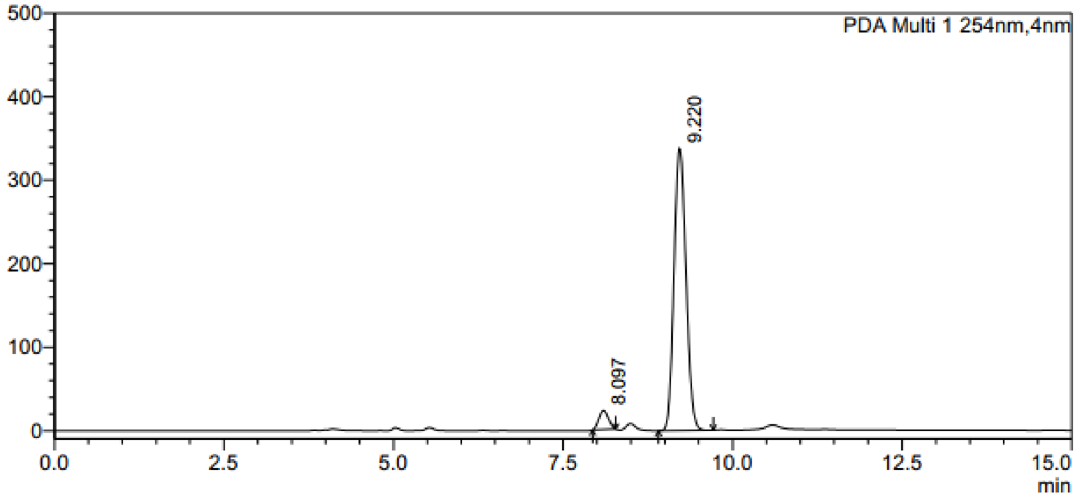


PDA Ch1 254nm

| Peak# | Ret. Time | Area | Area% | Height |
|---|---|---|---|---|
| 1 | 8.097 | 223438 | 5.070 | 22523 |
| 2 | 9.220 | 4183518 | 94.930 | 337760 |
| Total | | 4406955 | 100.000 | 360283 |

**Supplementary Figure 49.** $^{1}H$, $^{13}C$ and HPLC spectra of **2c**

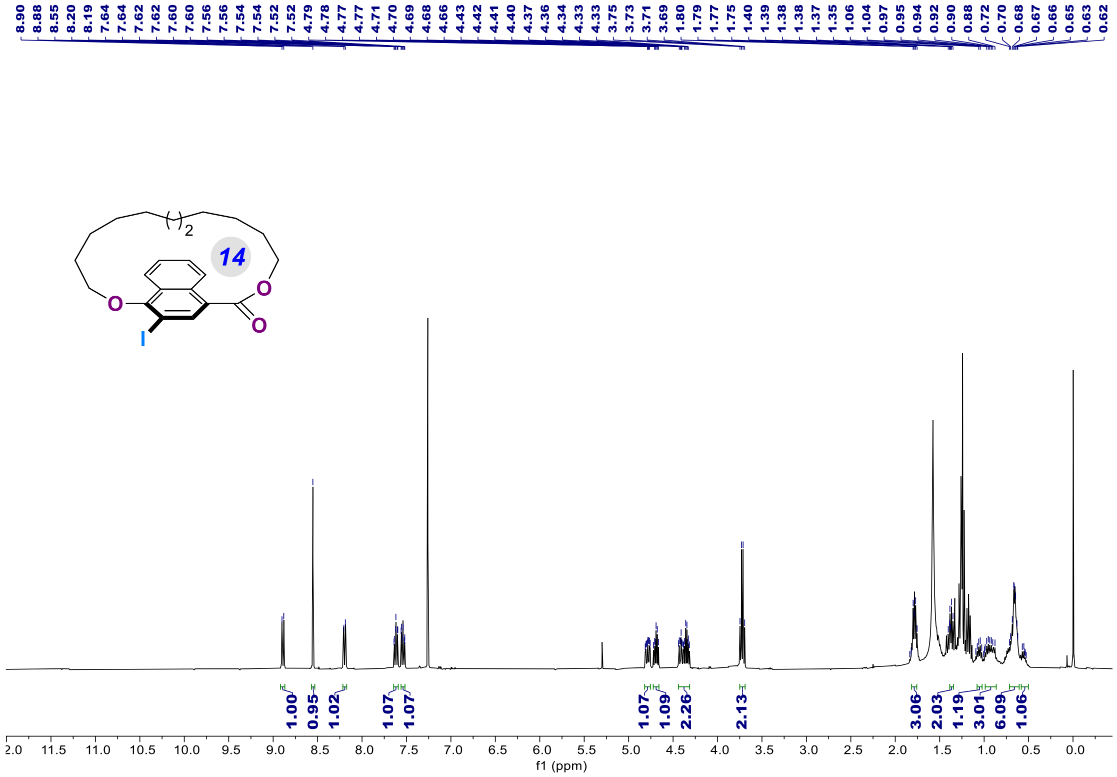


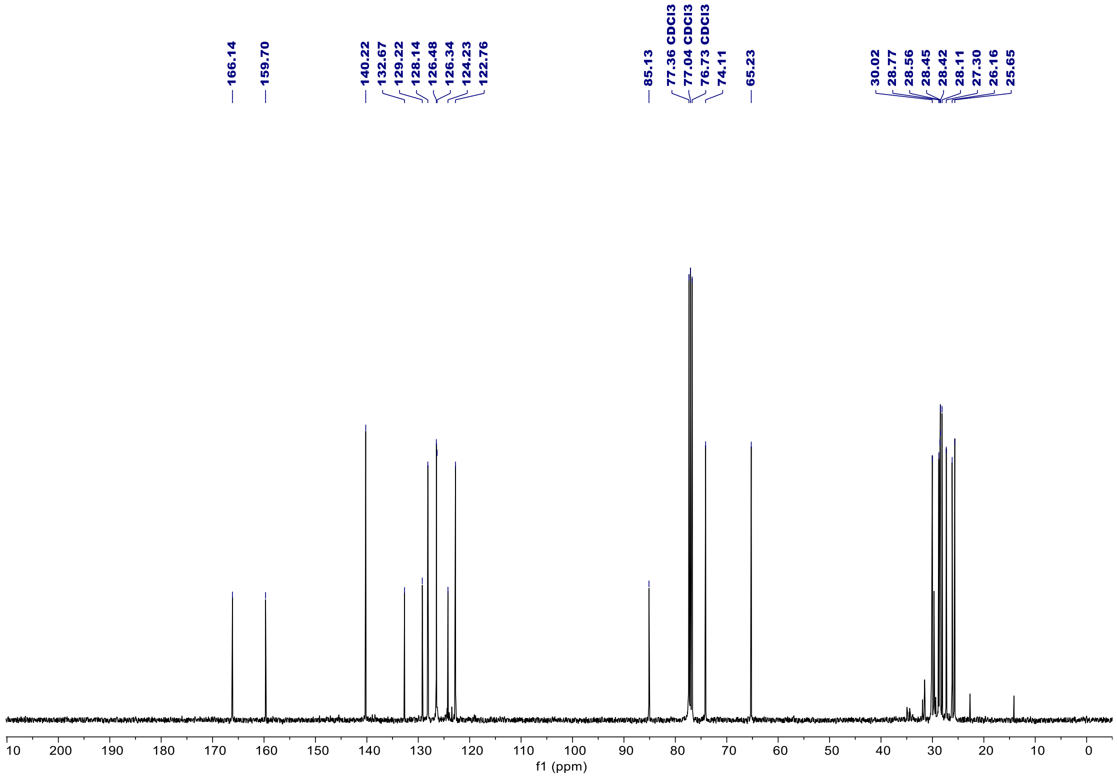

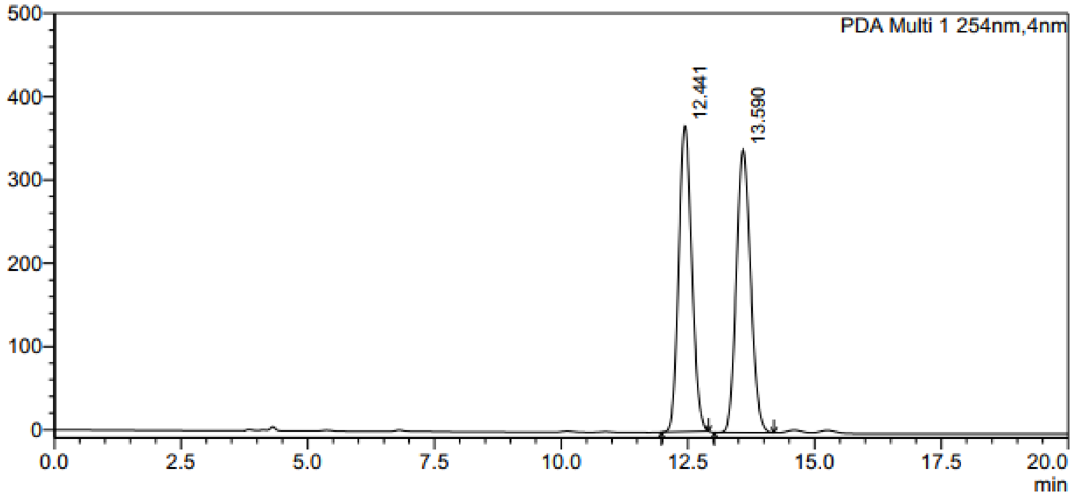


PDA Ch1 254nm

| Peak# | Ret. Time | Area | Area% | Height |
|---|---|---|---|---|
| 1 | 12.441 | 6627299 | 49.766 | 367364 |
| 2 | 13.590 | 6689718 | 50.234 | 339843 |
| Total | | 13317017 | 100.000 | 707208 |

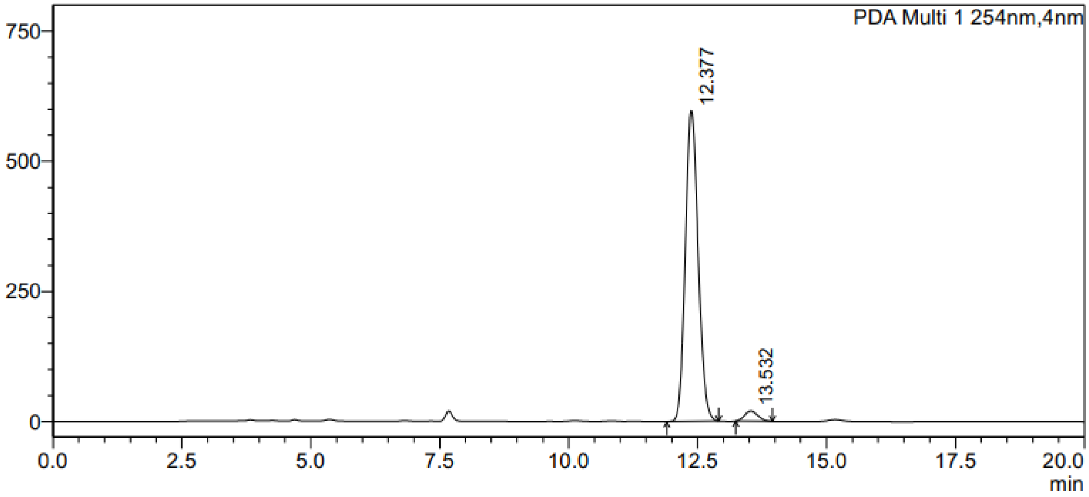


PDA Ch1 254nm

| Peak# | Ret. Time | Area | Area% | Height |
|---|---|---|---|---|
| 1 | 12.377 | 10364985 | 96.652 | 596627 |
| 2 | 13.532 | 359060 | 3.348 | 19825 |
| Total | | 10724045 | 100.000 | 616452 |

**Supplementary Figure 50.** $^{1}$H, $^{13}$C and HPLC spectra of **2d**

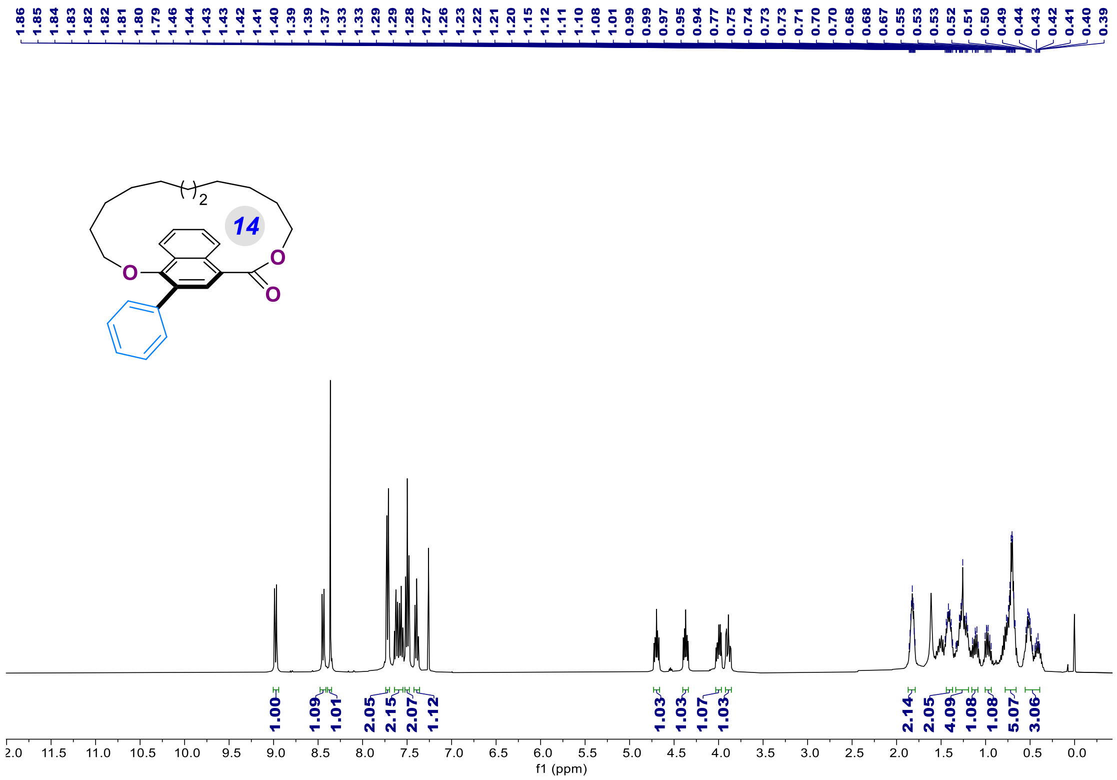


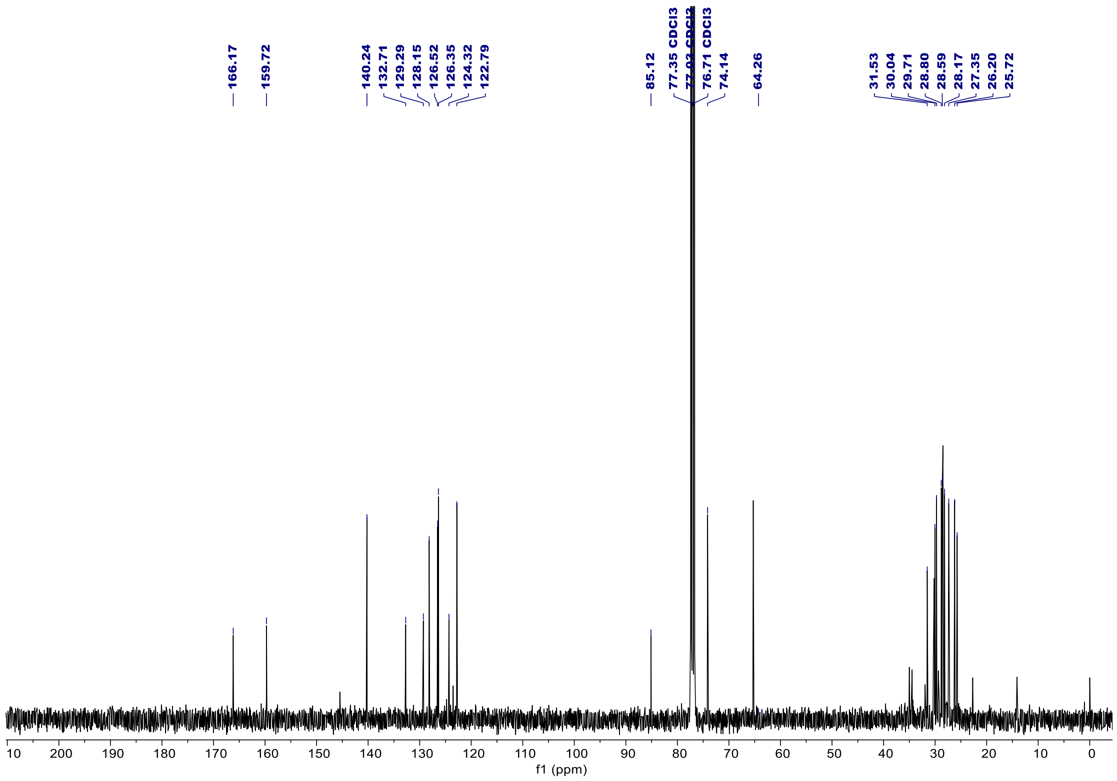

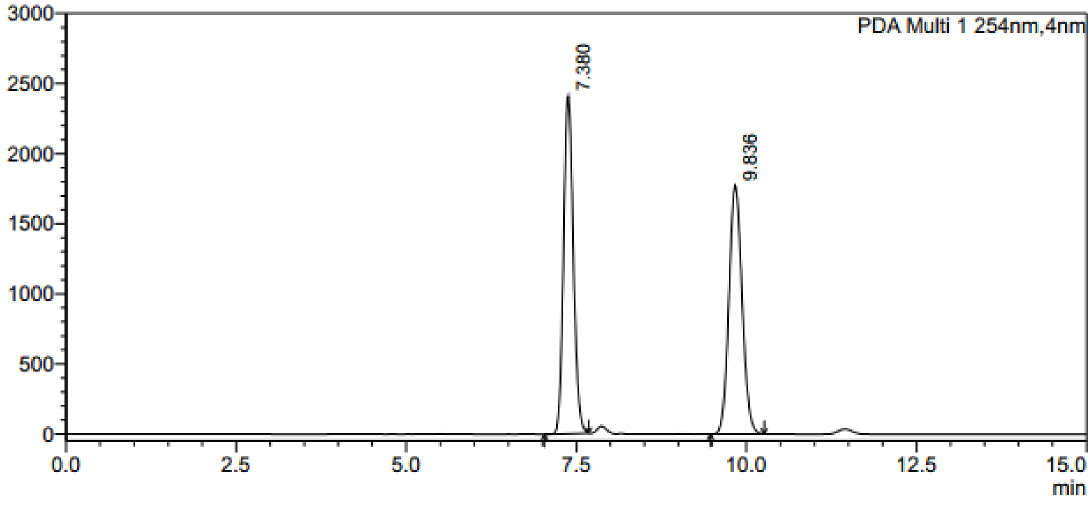


PDA Ch1 254nm

| Peak# | Ret. Time | Area | Area% | Height |
|---|---|---|---|---|
| 1 | 7.380 | 23694683 | 49.704 | 2405179 |
| 2 | 9.836 | 23976427 | 50.296 | 1773262 |
| Total | | 47671110 | 100.000 | 4178441 |

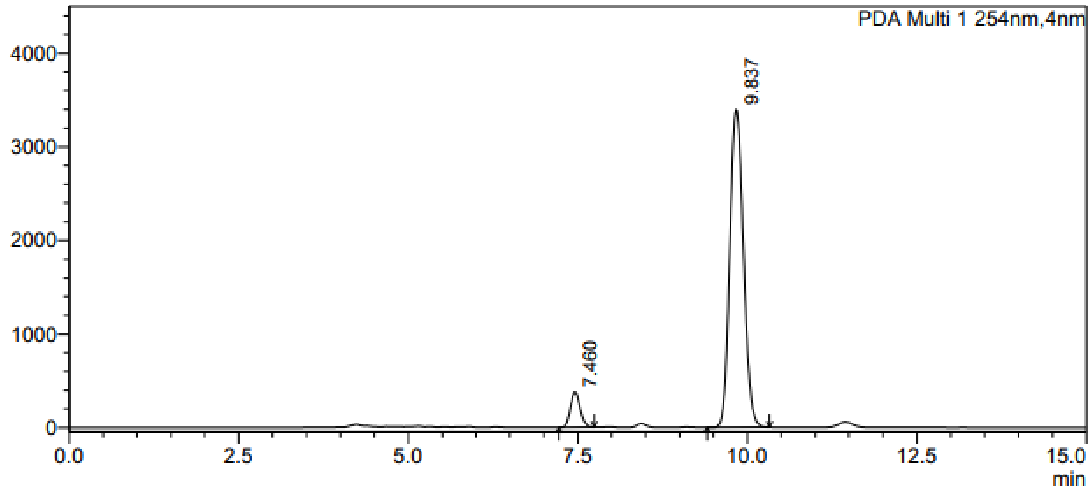


PDA Ch1 254nm

| Peak# | Ret. Time | Area | Area% | Height |
|---|---|---|---|---|
| 1 | 7.460 | 3764990 | 7.383 | 377492 |
| 2 | 9.837 | 47231219 | 92.617 | 3390250 |
| Total | | 50996209 | 100.000 | 3767741 |

**Supplementary Figure 51.** $^1$H, $^{13}$C and HPLC spectra of **2e**

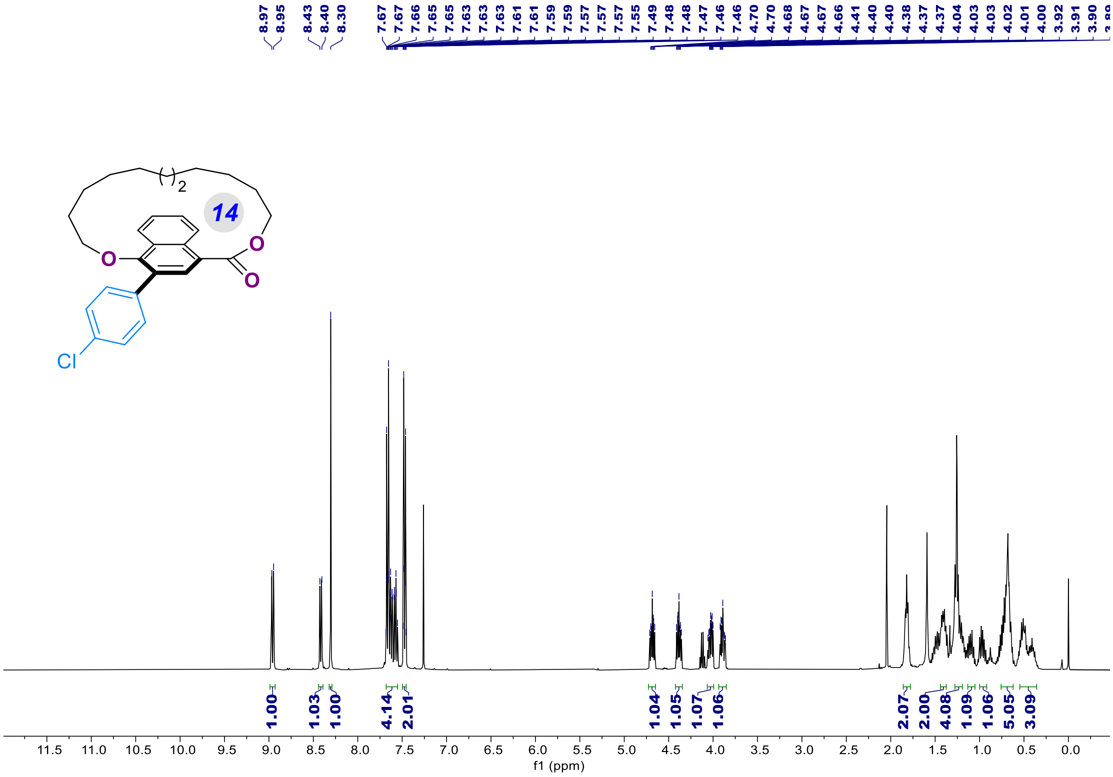

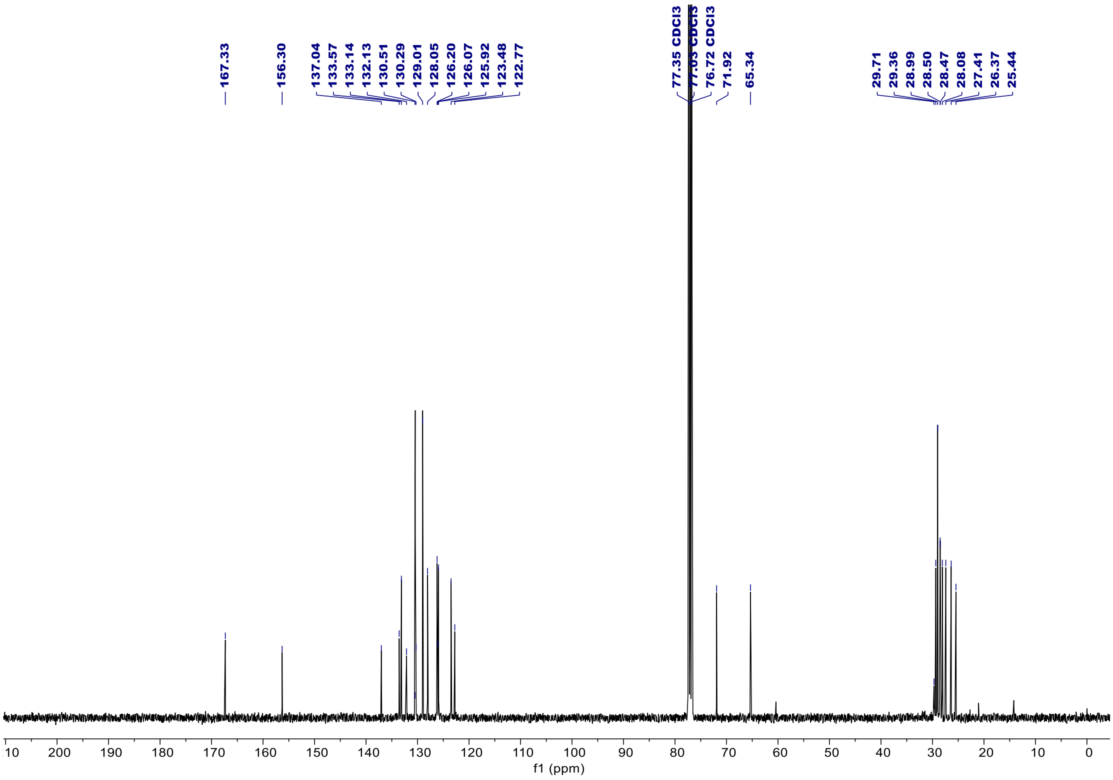

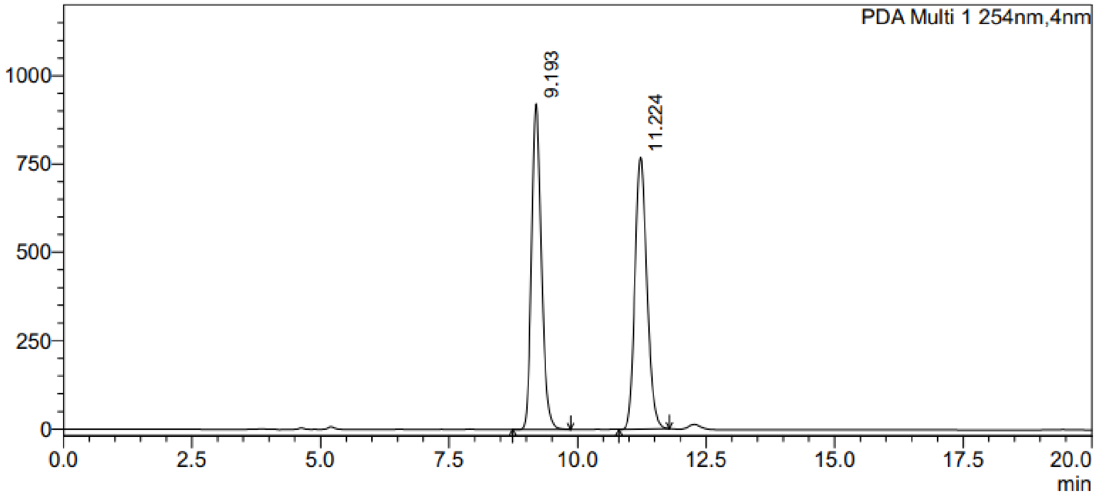


PDA Ch1 254nm

| Peak# | Ret. Time | Area | Area% | Height |
|---|---|---|---|---|
| 1 | 9.193 | 12341300 | 49.698 | 922482 |
| 2 | 11.224 | 12491315 | 50.302 | 768744 |
| Total | | 24832616 | 100.000 | 1691227 |

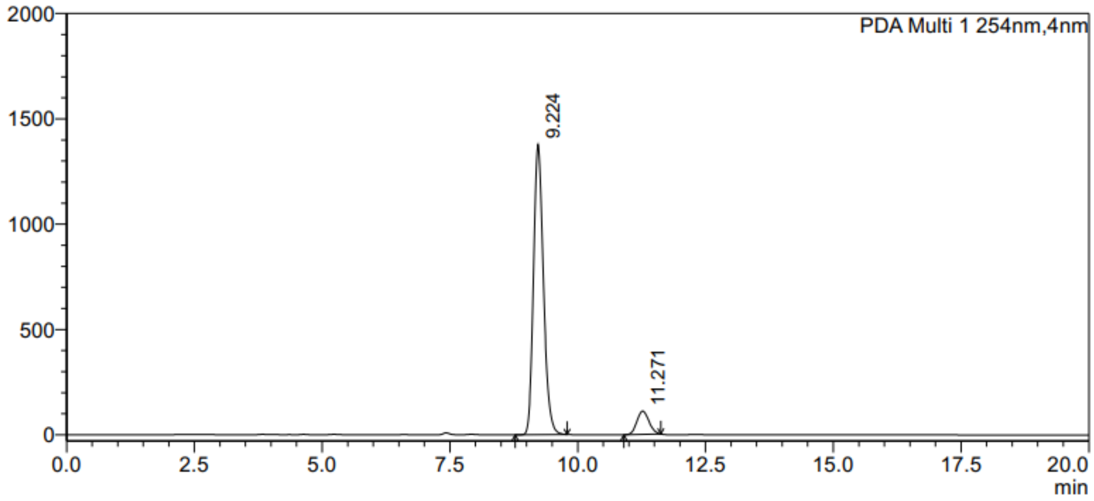


PDA Ch1 254nm

| Peak# | Ret. Time | Area | Area% | Height |
|---|---|---|---|---|
| 1 | 9.224 | 18670585 | 91.071 | 1376902 |
| 2 | 11.271 | 1830581 | 8.929 | 111150 |
| Total | | 20501167 | 100.000 | 1488051 |

**Supplementary Figure 52.** $^{1}$H, $^{13}$C and HPLC spectra of **2f**

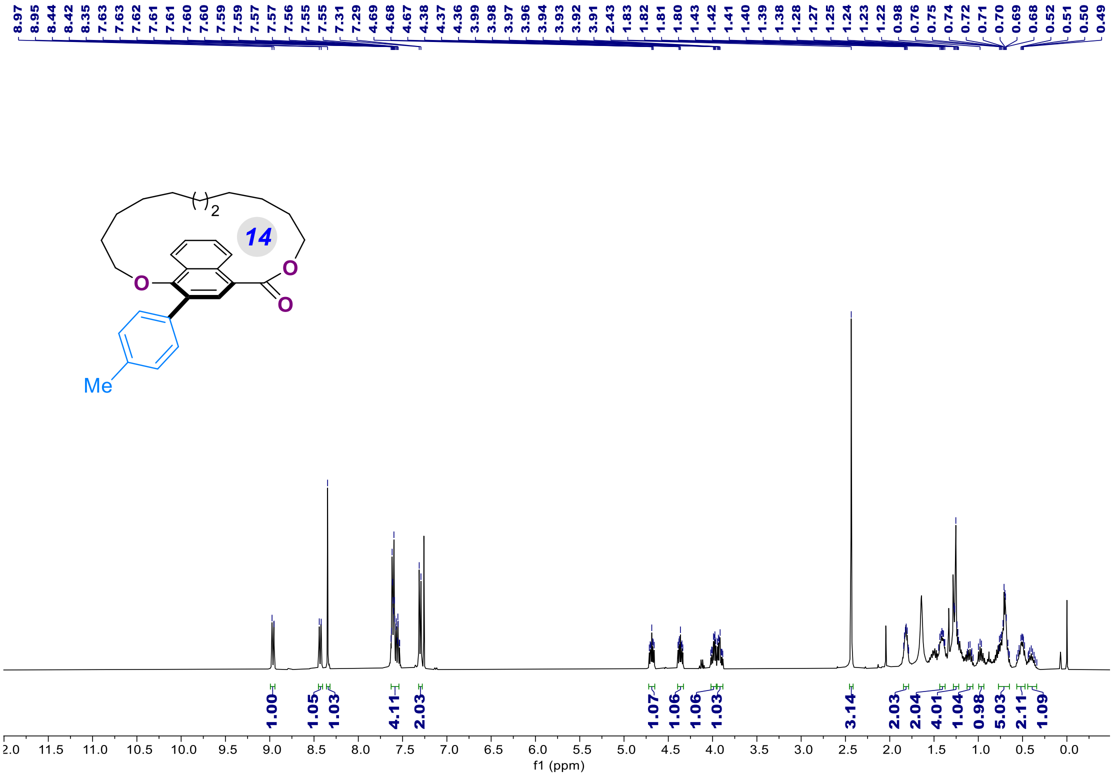


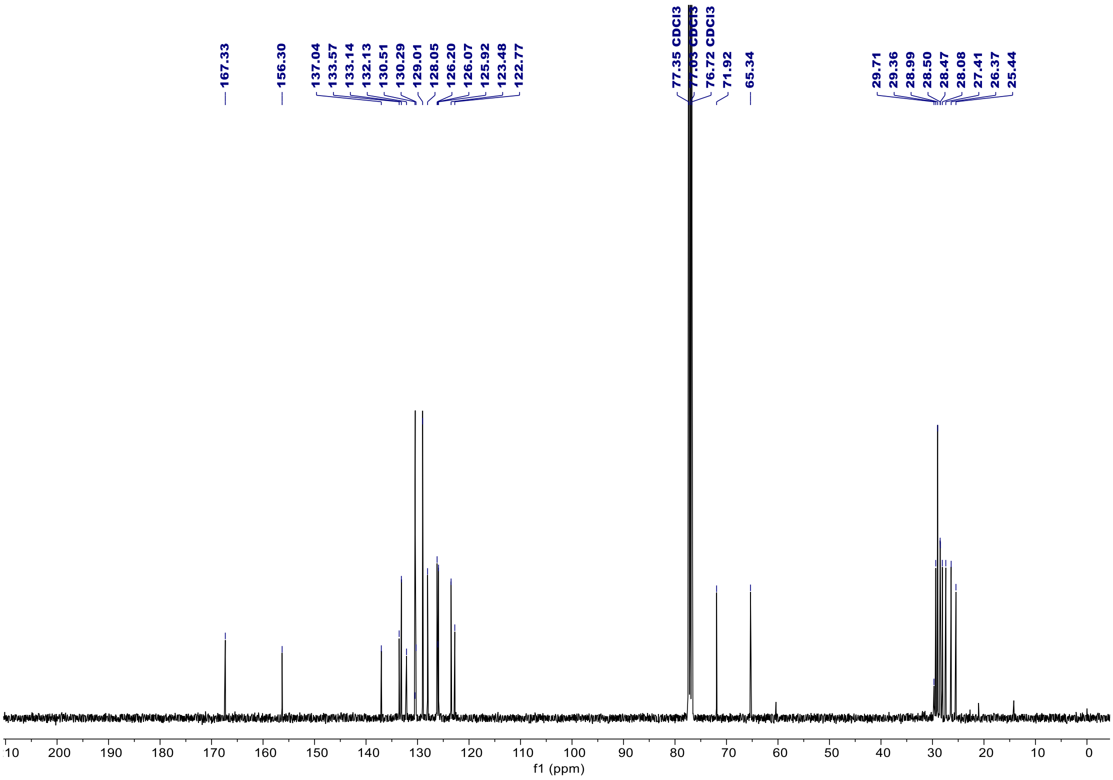

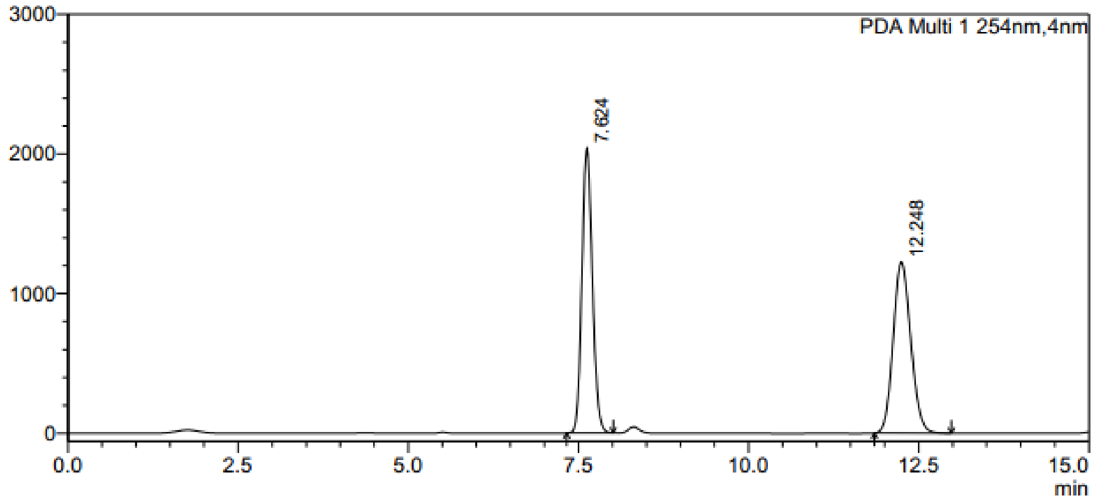


PDA Ch1 254nm

| Peak# | Ret. Time | Area | Area% | Height |
|---|---|---|---|---|
| 1 | 7.624 | 22004803 | 49.834 | 2035251 |
| 2 | 12.248 | 22151744 | 50.166 | 1225595 |
| Total | | 44156547 | 100.000 | 3260846 |

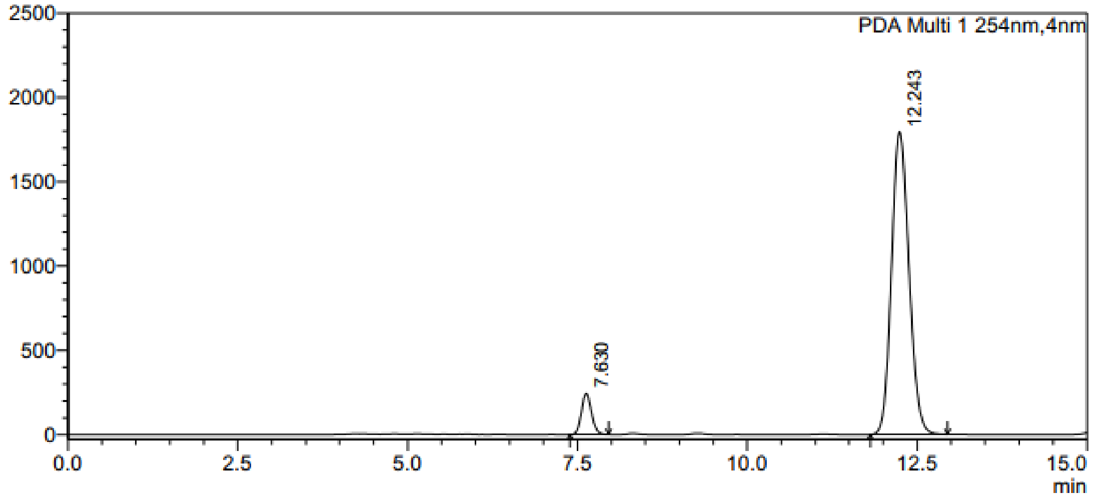


PDA Ch1 254nm

| Peak# | Ret. Time | Area | Area% | Height |
|---|---|---|---|---|
| 1 | 7.630 | 2522136 | 7.310 | 242465 |
| 2 | 12.243 | 31981182 | 92.690 | 1794598 |
| Total | | 34503318 | 100.000 | 2037063 |

**Supplementary Figure 53.** $^{1}$H, $^{13}$C and HPLC spectra of **2g**

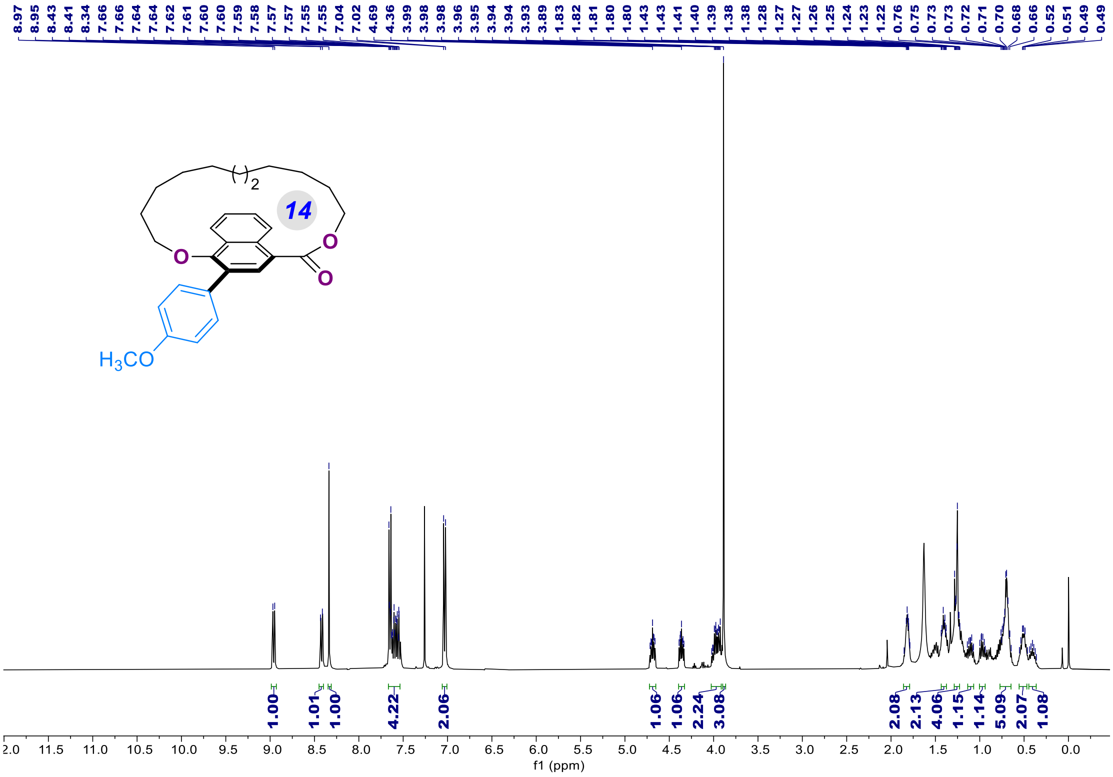


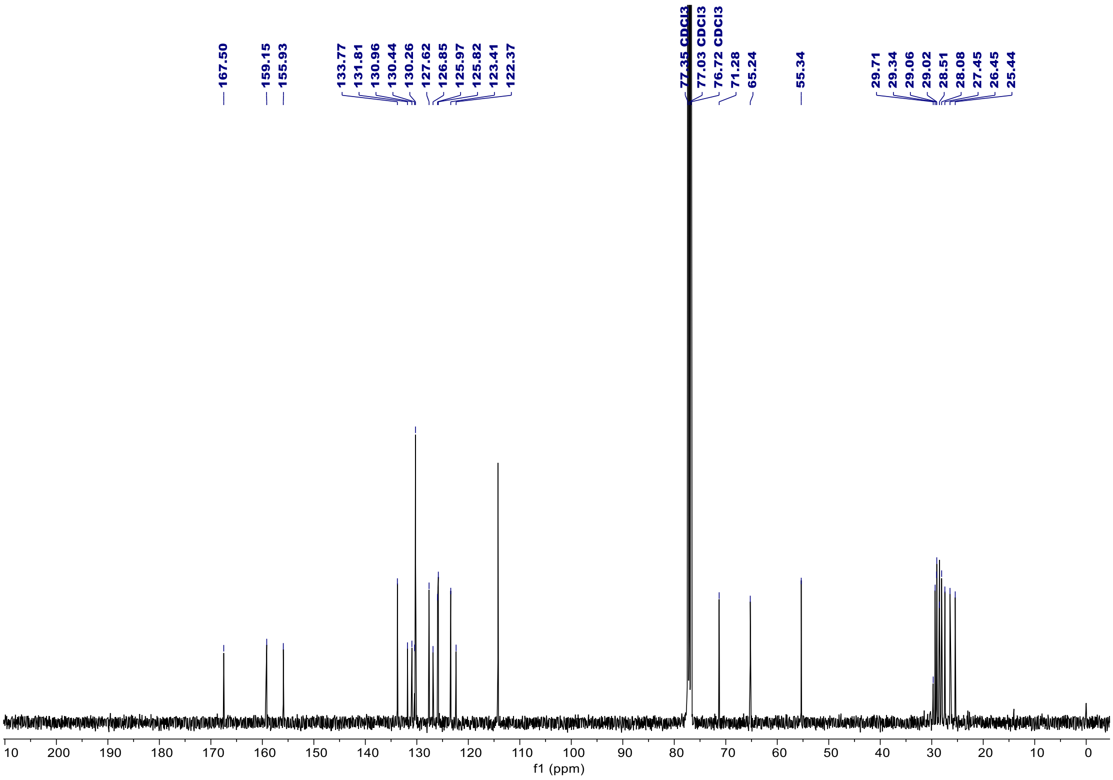

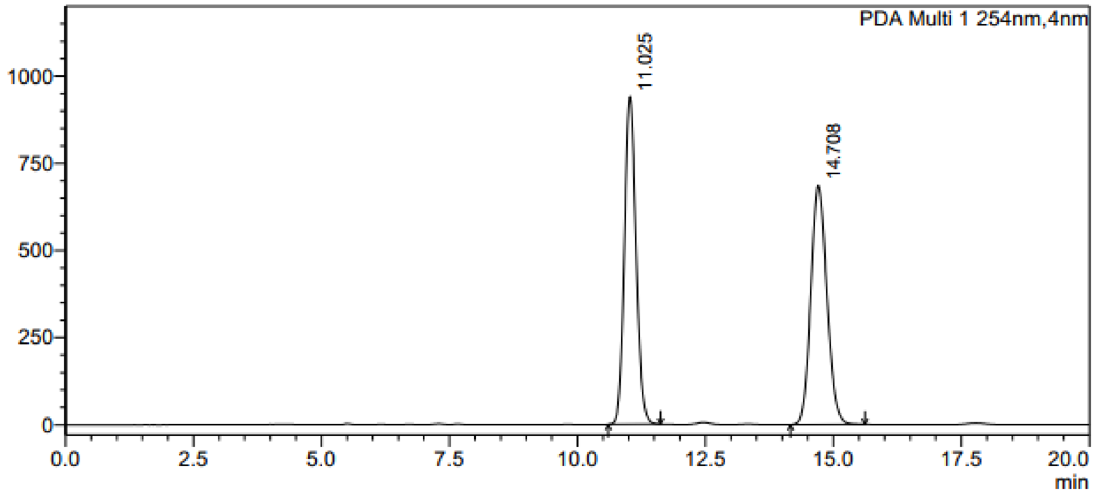


PDA Ch1 254nm

| Peak# | Ret. Time | Area | Area% | Height |
|---|---|---|---|---|
| 1 | 11.025 | 14969939 | 49.943 | 939337 |
| 2 | 14.708 | 15003971 | 50.057 | 685515 |
| Total | | 29973910 | 100.000 | 1624852 |

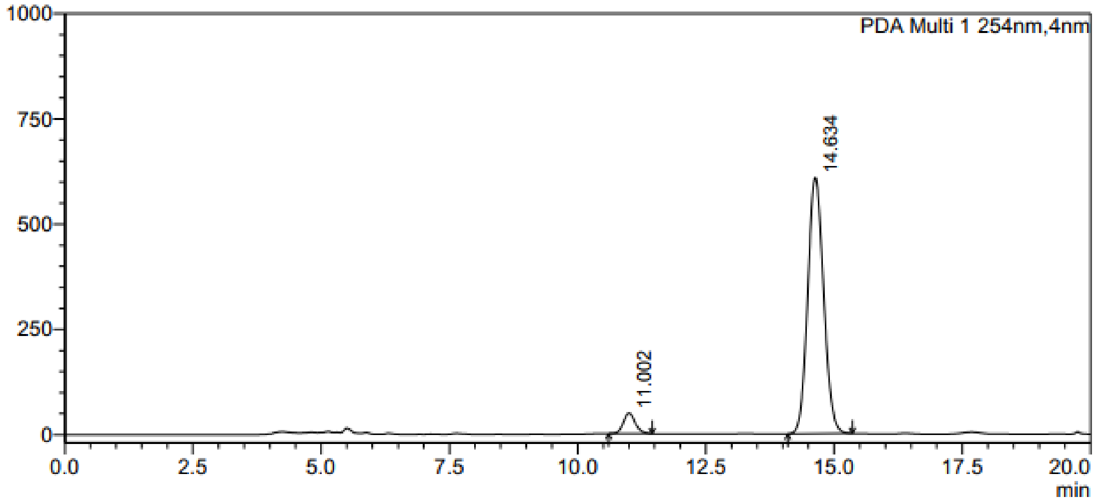


PDA Ch1 254nm

| Peak# | Ret. Time | Area | Area% | Height |
|---|---|---|---|---|
| 1 | 11.002 | 789700 | 5.682 | 48432 |
| 2 | 14.634 | 13107783 | 94.318 | 607898 |
| Total | | 13897483 | 100.000 | 656330 |

**Supplementary Figure 54.** $^{1}$H, $^{13}$C and HPLC spectra of **2h**

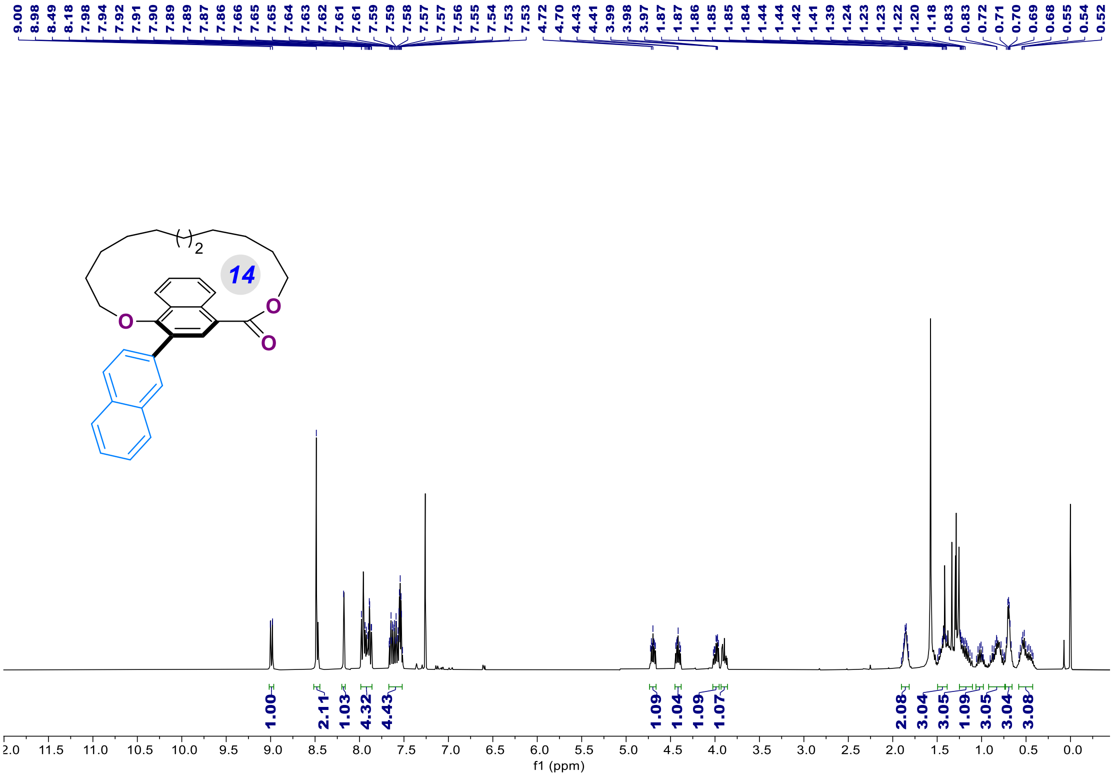


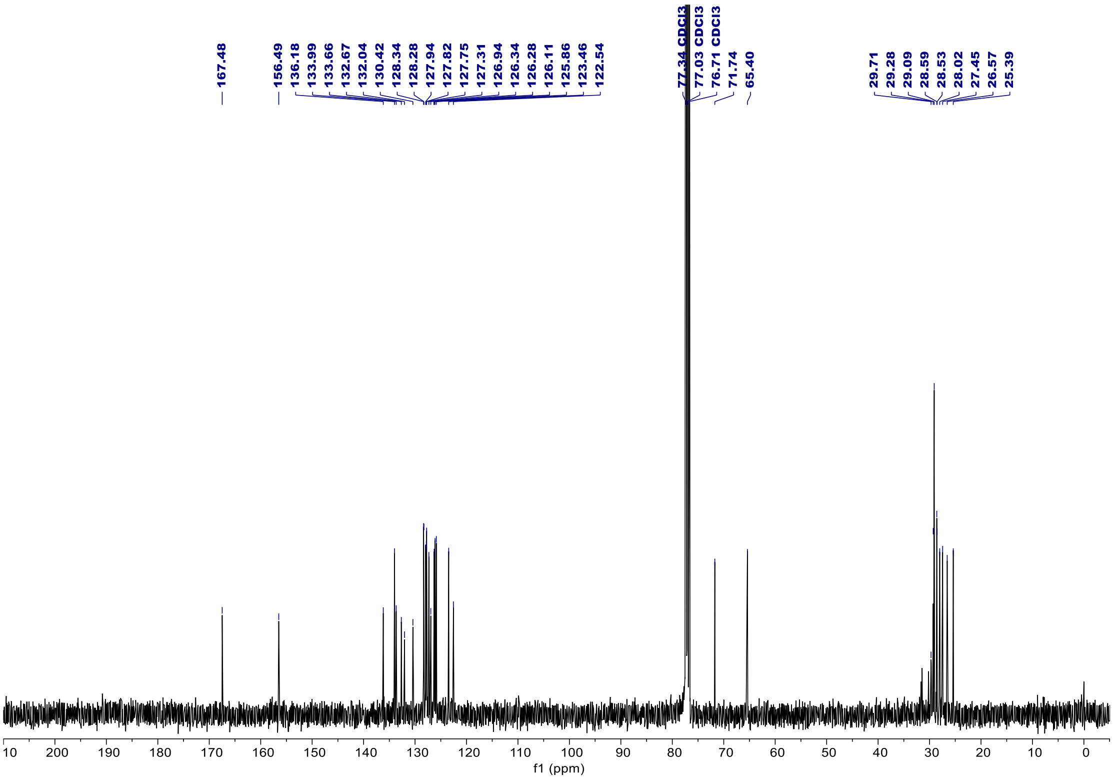

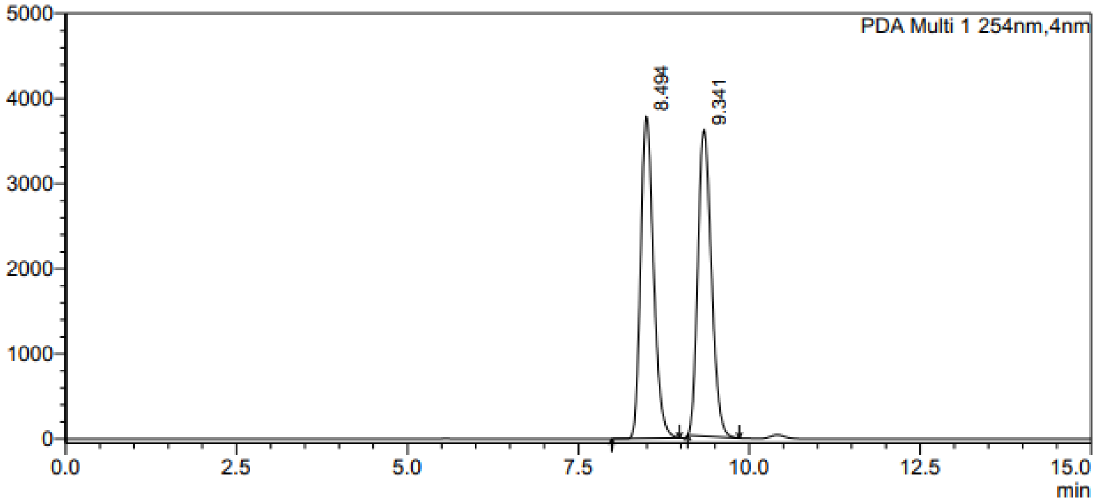


PDA Ch1 254nm

| Peak# | Ret. Time | Area | Area% | Height |
|---|---|---|---|---|
| 1 | 8.494 | 49739653 | 50.012 | 3775691 |
| 2 | 9.341 | 49715863 | 49.988 | 3600109 |
| Total | | 99455516 | 100.000 | 7375799 |

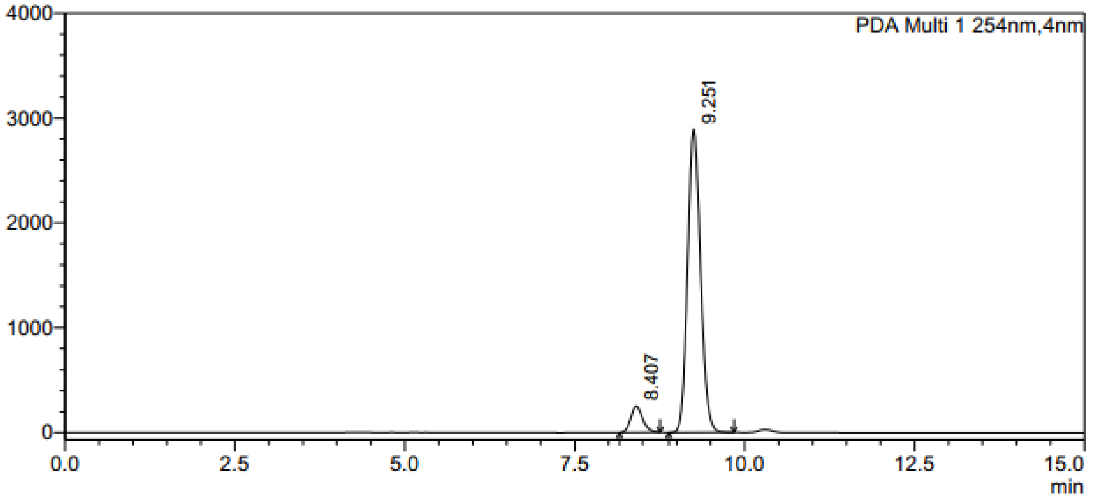


PDA Ch1 254nm

| Peak# | Ret. Time | Area | Area% | Height |
|---|---|---|---|---|
| 1 | 8.407 | 3046469 | 7.418 | 249564 |
| 2 | 9.251 | 38023610 | 92.582 | 2885660 |
| Total | | 41070080 | 100.000 | 3135224 |

**Supplementary Figure 55.** $^1$H, $^{13}$C and HPLC spectra of **2i**

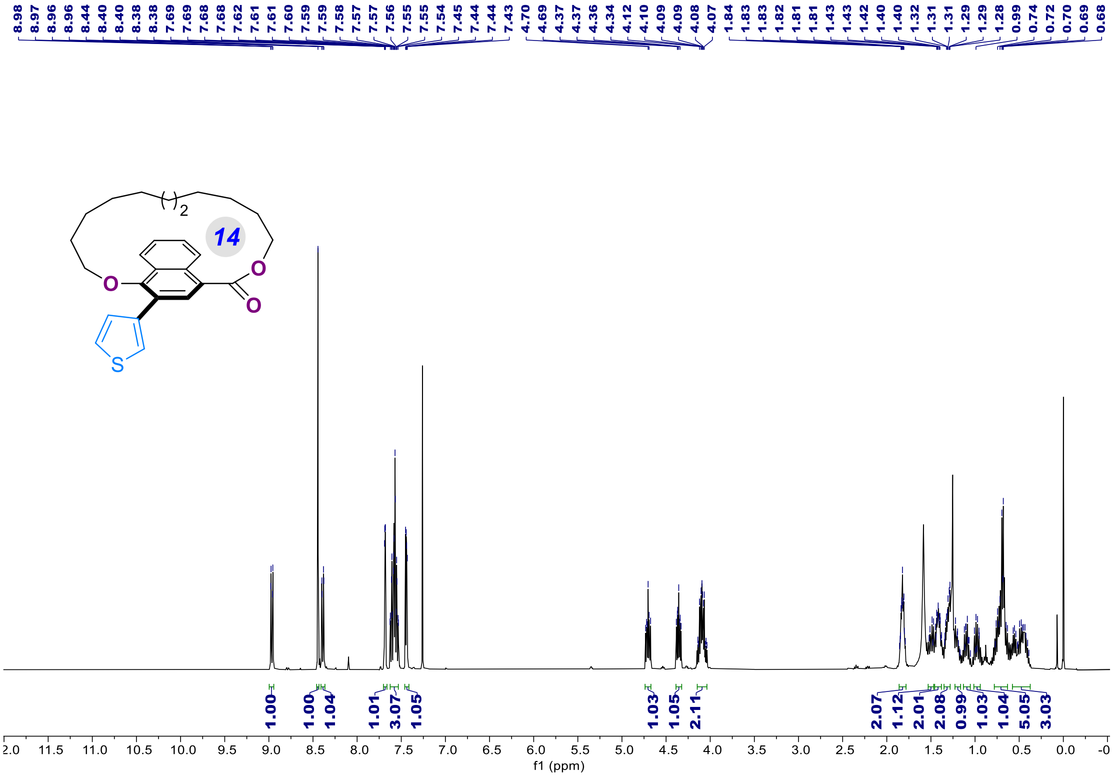


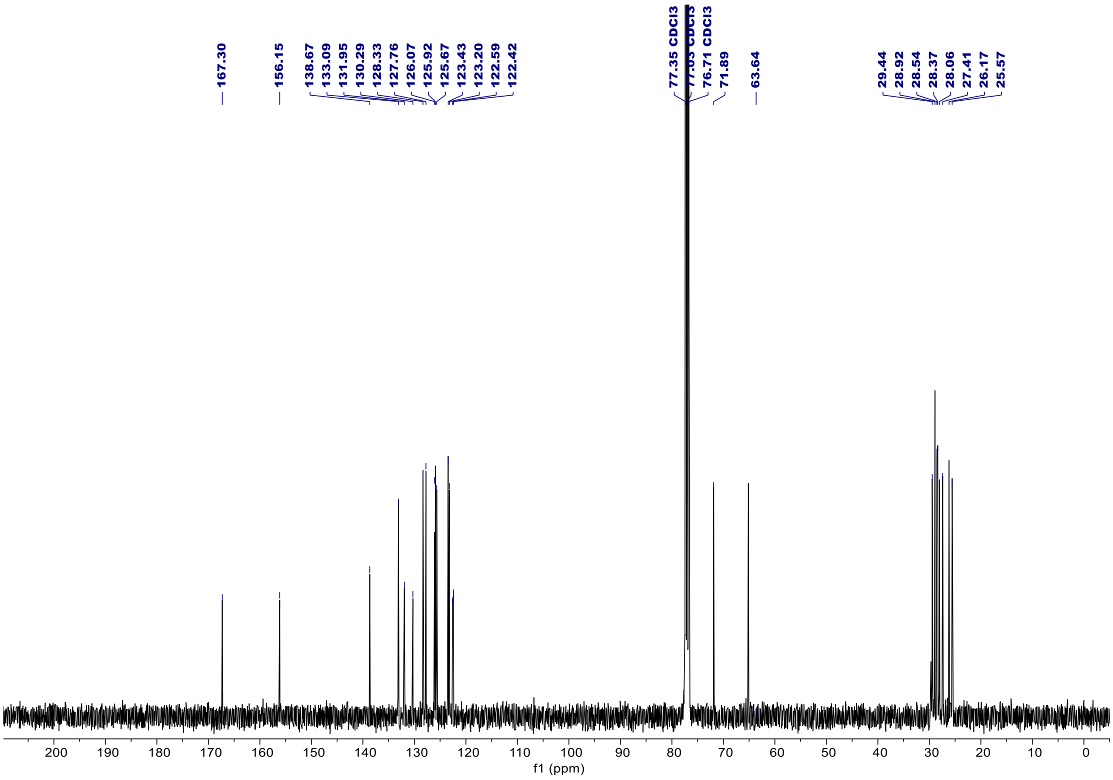

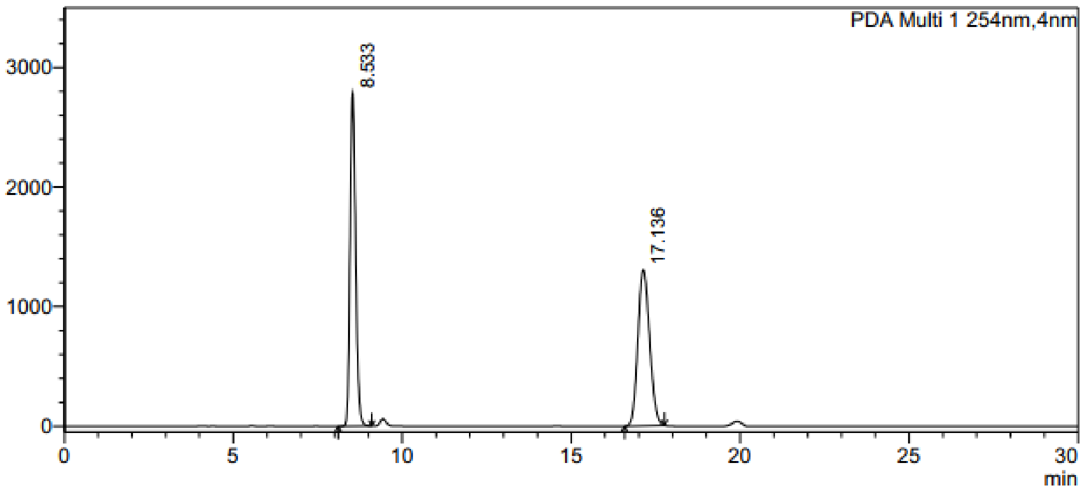


PDA Ch1 254nm

| Peak# | Ret. Time | Area | Area% | Height |
|---|---|---|---|---|
| 1 | 8.533 | 31982458 | 50.371 | 2775627 |
| 2 | 17.136 | 31511551 | 49.629 | 1302939 |
| Total | | 63494009 | 100.000 | 4078566 |

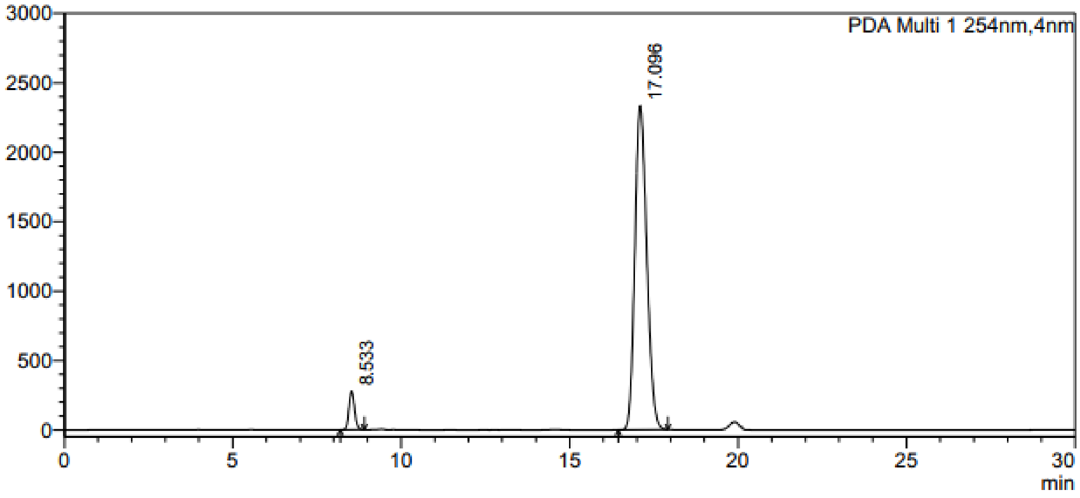


PDA Ch1 254nm

| Peak# | Ret. Time | Area | Area% | Height |
|---|---|---|---|---|
| 1 | 8.533 | 3202671 | 5.301 | 275881 |
| 2 | 17.096 | 57214070 | 94.699 | 2330517 |
| Total | | 60416741 | 100.000 | 2606398 |

**Supplementary Figure 56.** $^{1}H$, $^{13}C$ and HPLC spectra of **2j**

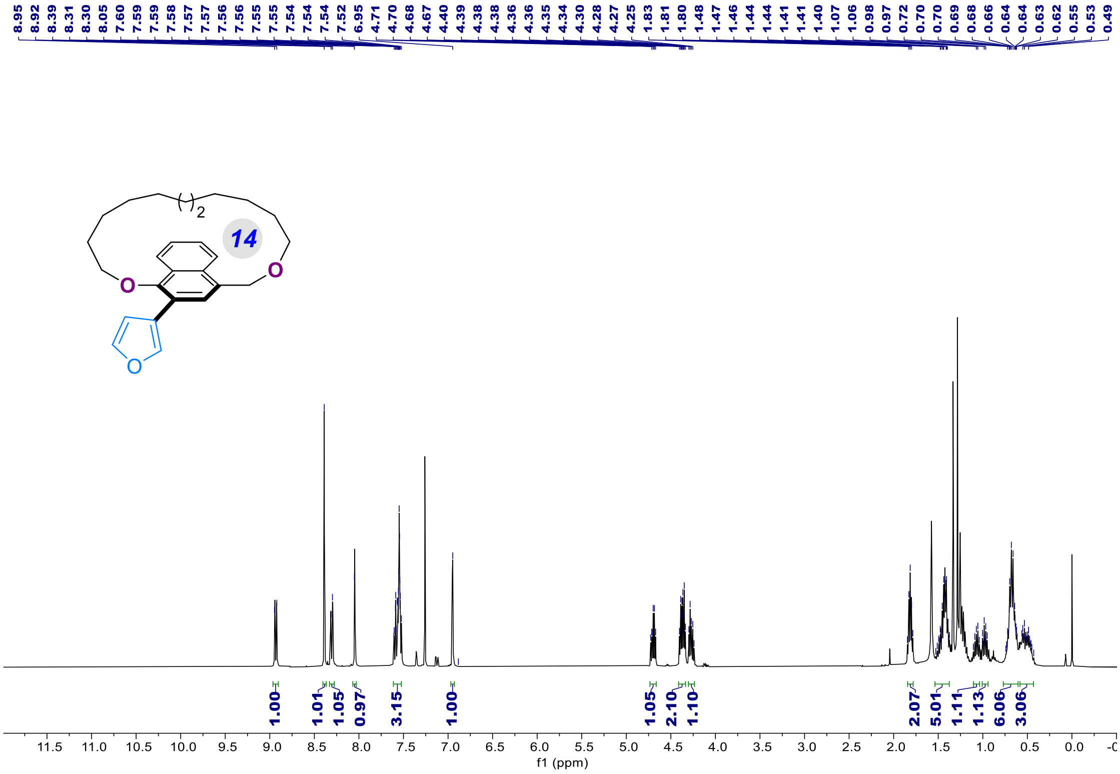

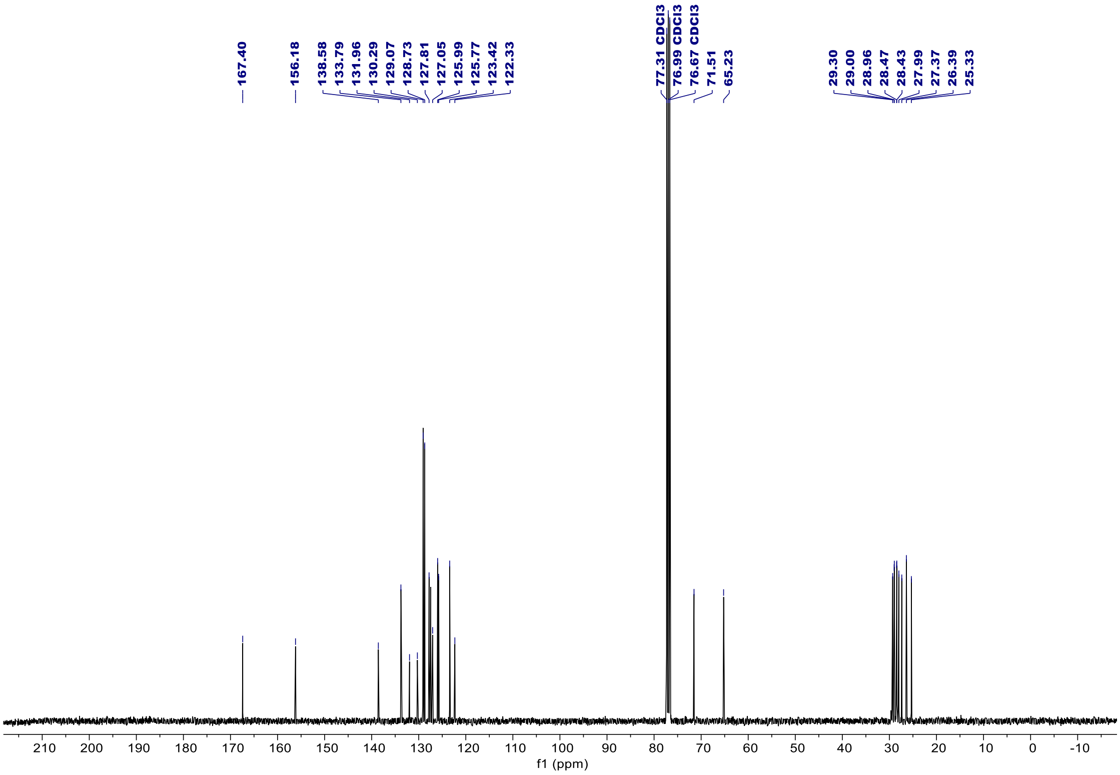

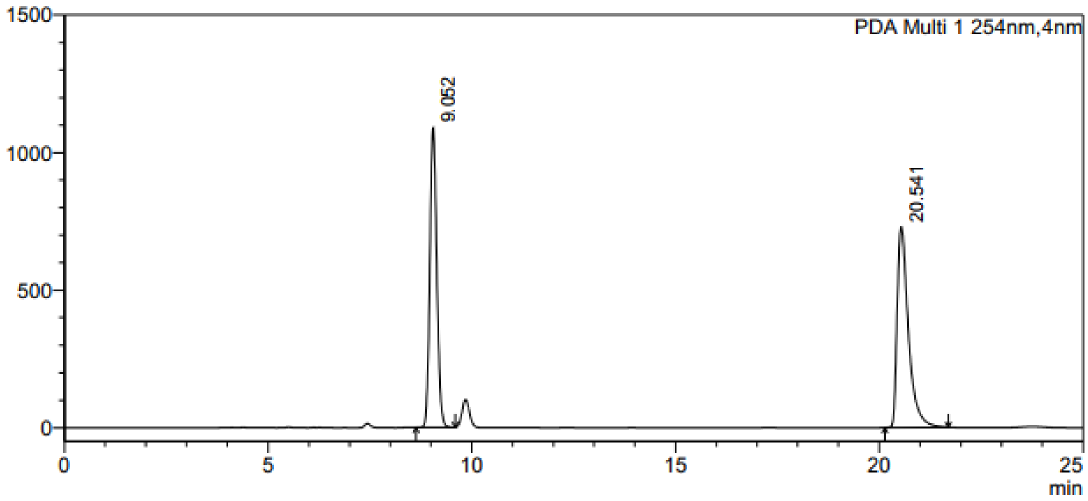


PDA Ch1 254nm

| Peak# | Ret. Time | Area | Area% | Height |
|---|---|---|---|---|
| 1 | 9.052 | 12937298 | 48.293 | 1087325 |
| 2 | 20.541 | 13851953 | 51.707 | 728823 |
| Total | | 26789251 | 100.000 | 1816148 |

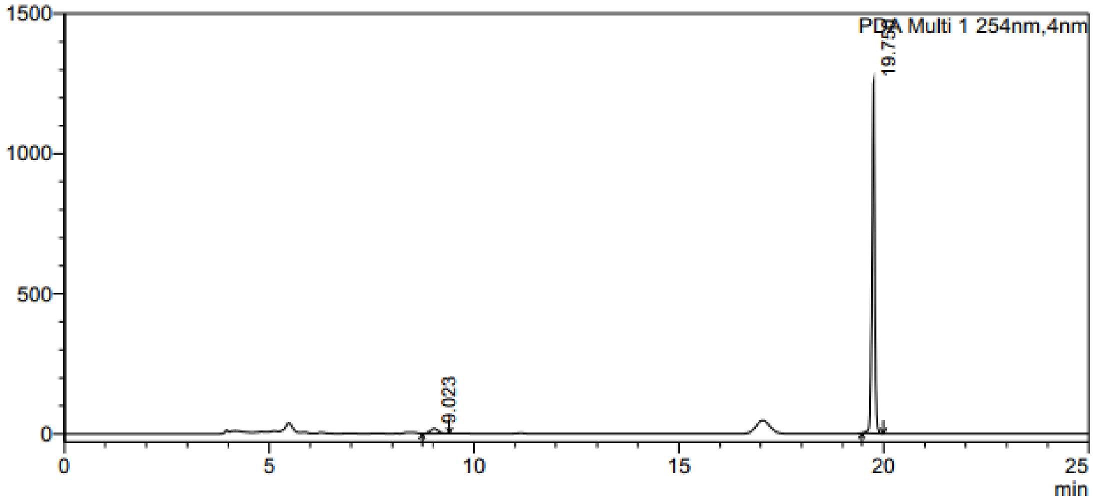


PDA Ch1 254nm

| Peak# | Ret. Time | Area | Area% | Height |
|---|---|---|---|---|
| 1 | 9.023 | 217321 | 3.582 | 16945 |
| 2 | 19.750 | 5849935 | 96.418 | 1259989 |
| Total | | 6067256 | 100.000 | 1276934 |

**Supplementary Figure 57.** $^{1}H$, $^{13}C$ and HPLC spectra of **2k**

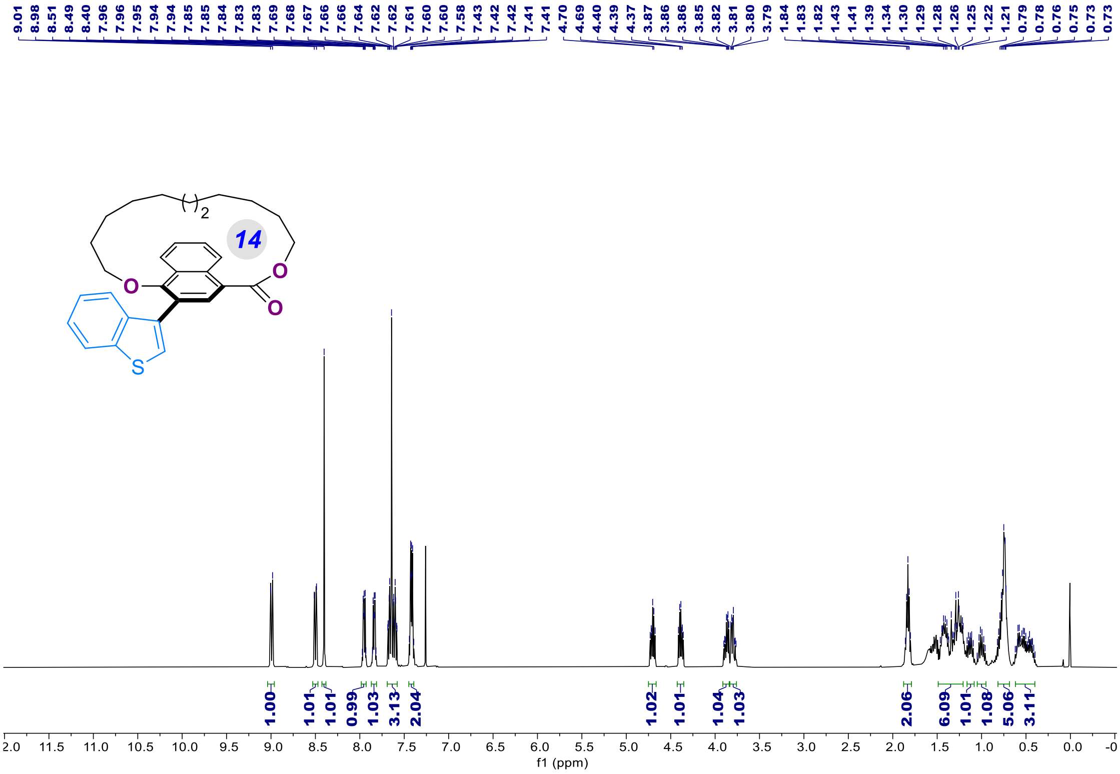


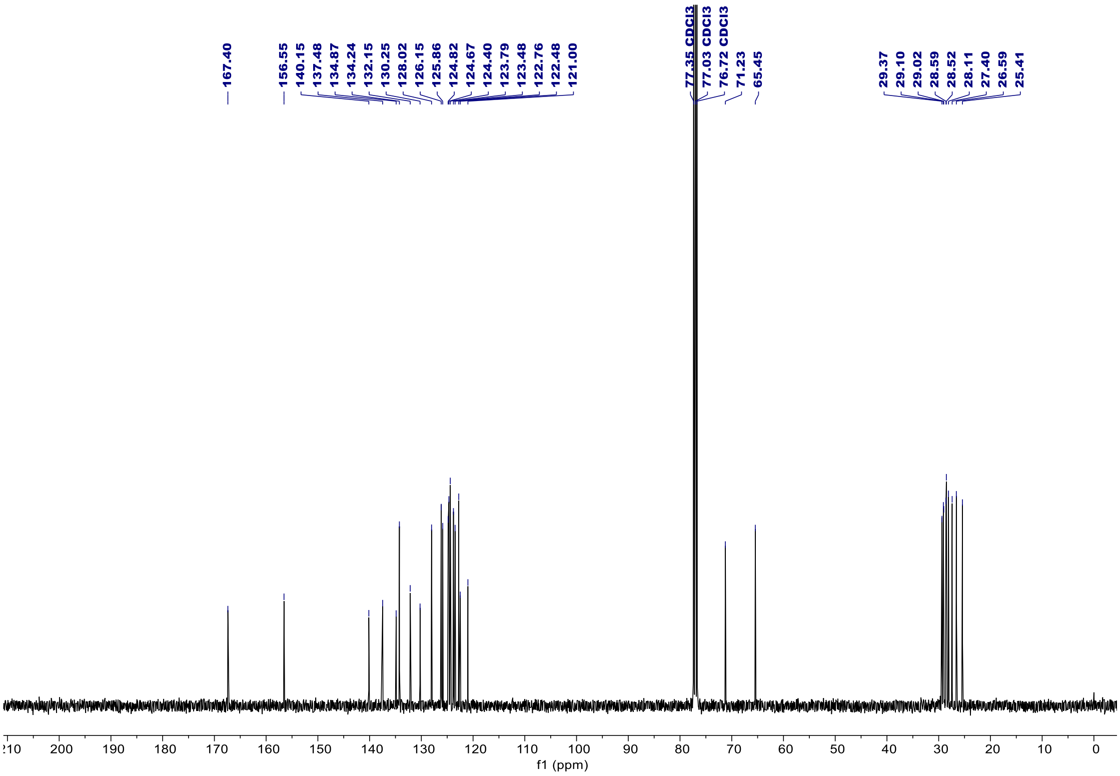

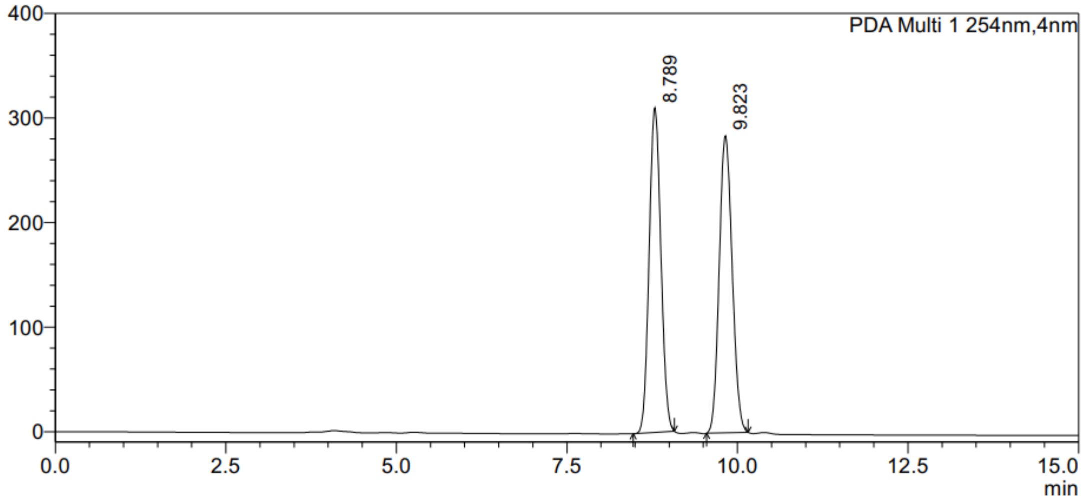


PDA Ch1 254nm

| Peak# | Ret. Time | Area | Area% | Height |
|---|---|---|---|---|
| 1 | 8.789 | 3713086 | 49.724 | 310531 |
| 2 | 9.823 | 3754325 | 50.276 | 284272 |
| Total | | 7467410 | 100.000 | 594802 |

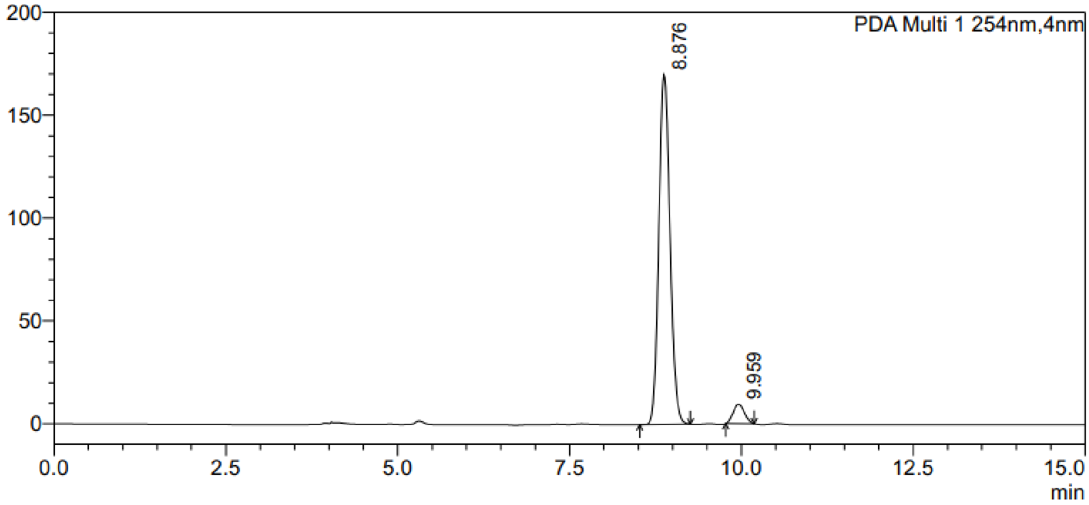


PDA Ch1 254nm

| Peak# | Ret. Time | Area | Area% | Height |
|---|---|---|---|---|
| 1 | 8.876 | 1918549 | 94.646 | 169766 |
| 2 | 9.959 | 108529 | 5.354 | 9397 |
| Total | | 2027078 | 100.000 | 179163 |

**Supplementary Figure 58.** $^{1}H$, $^{13}C$ and HPLC spectra of **2l**

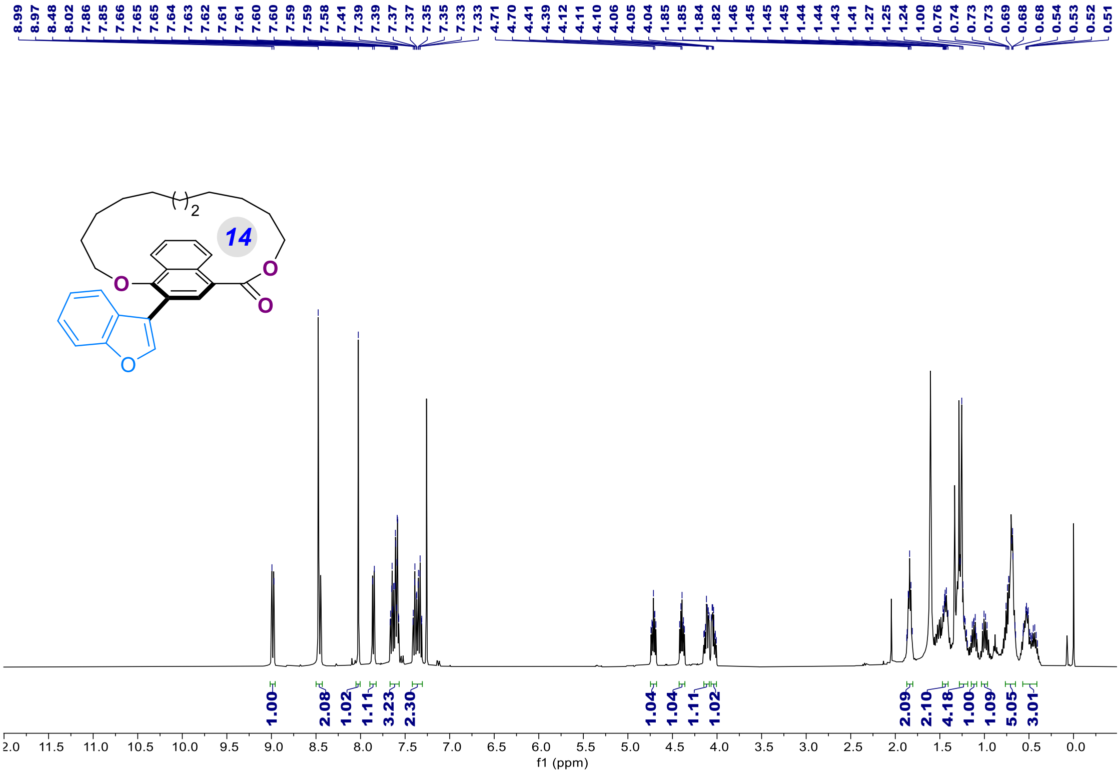


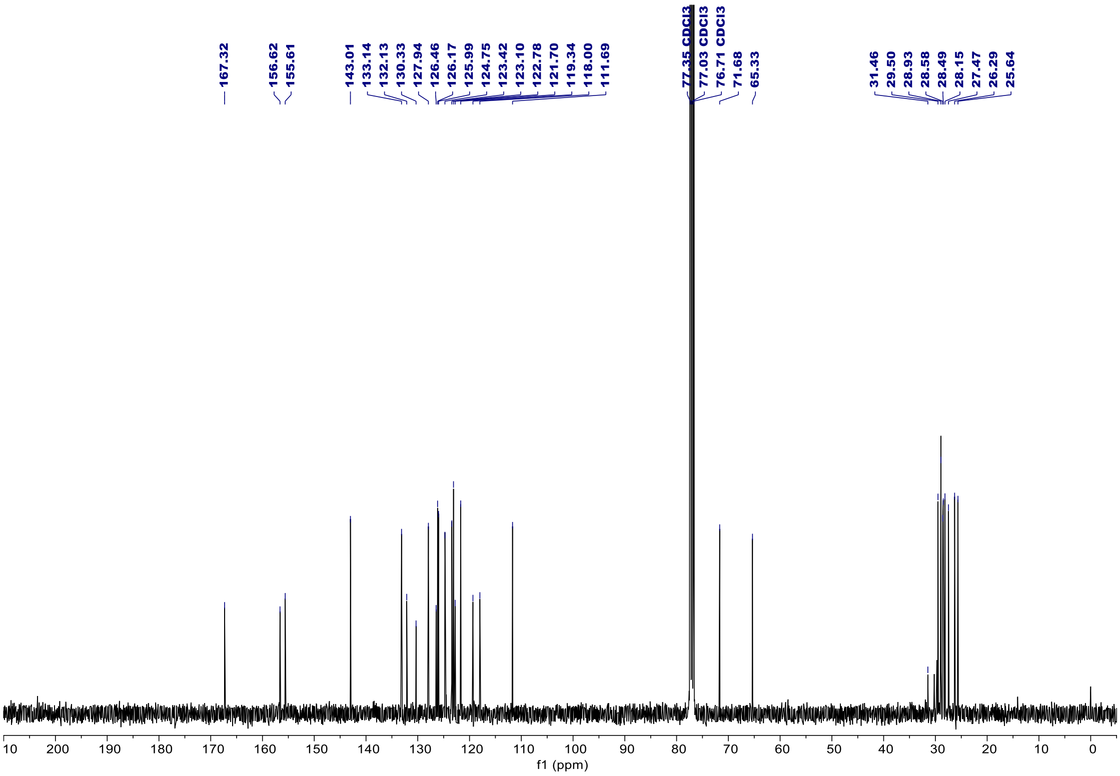

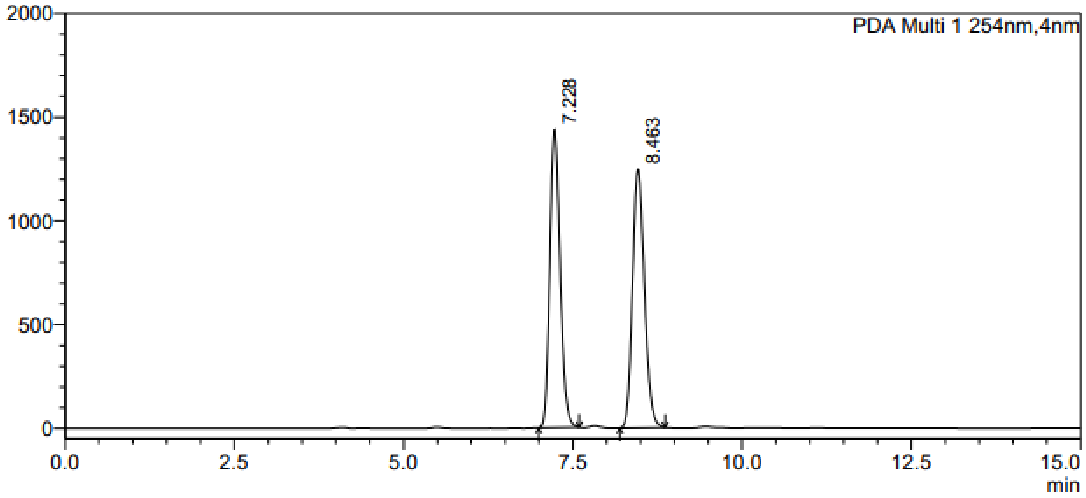


PDA Ch1 254nm

| Peak# | Ret. Time | Area | Area% | Height |
|---|---|---|---|---|
| 1 | 7.228 | 15223533 | 49.727 | 1436824 |
| 2 | 8.463 | 15390406 | 50.273 | 1245473 |
| Total | | 30613939 | 100.000 | 2682297 |

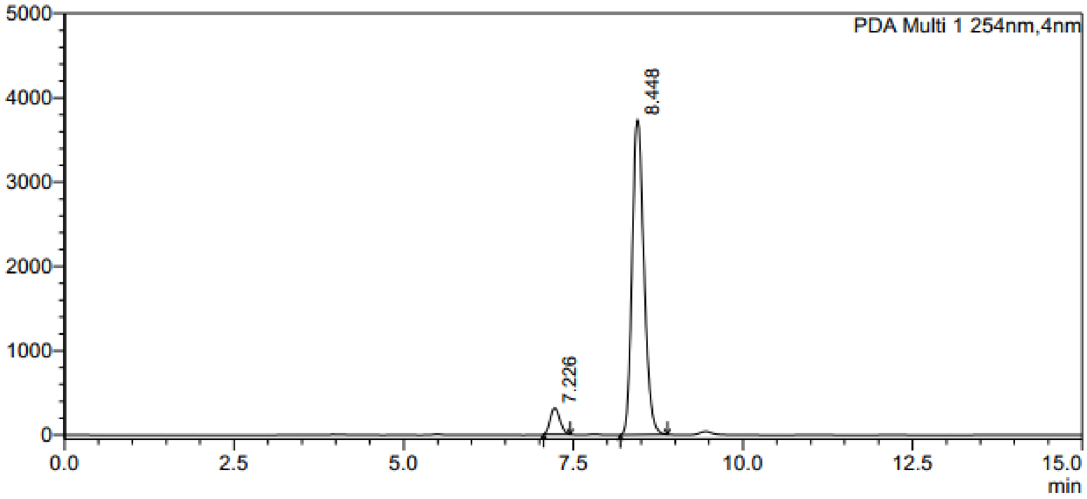


PDA Ch1 254nm

| Peak# | Ret. Time | Area | Area% | Height |
|---|---|---|---|---|
| 1 | 7.226 | 3137453 | 6.435 | 314566 |
| 2 | 8.448 | 45621800 | 93.565 | 3726692 |
| Total | | 48759253 | 100.000 | 4041258 |

**Supplementary Figure 59.** $^{1}H$, $^{13}C$ and HPLC spectra of **2m**

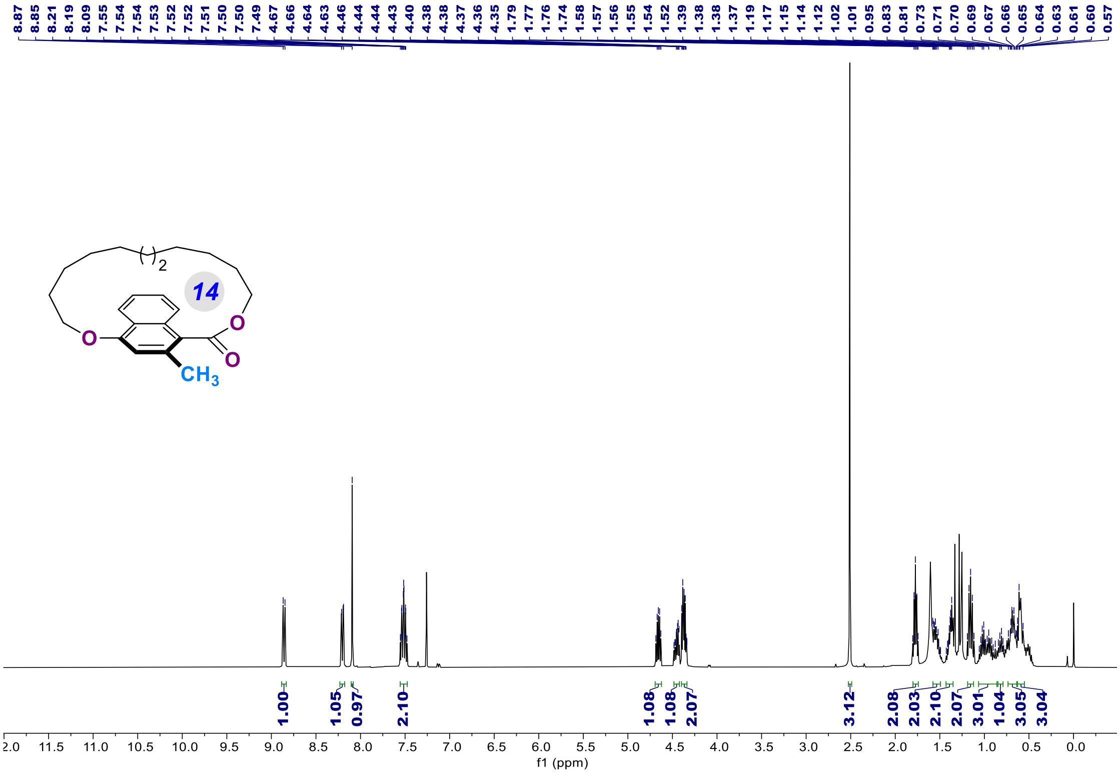


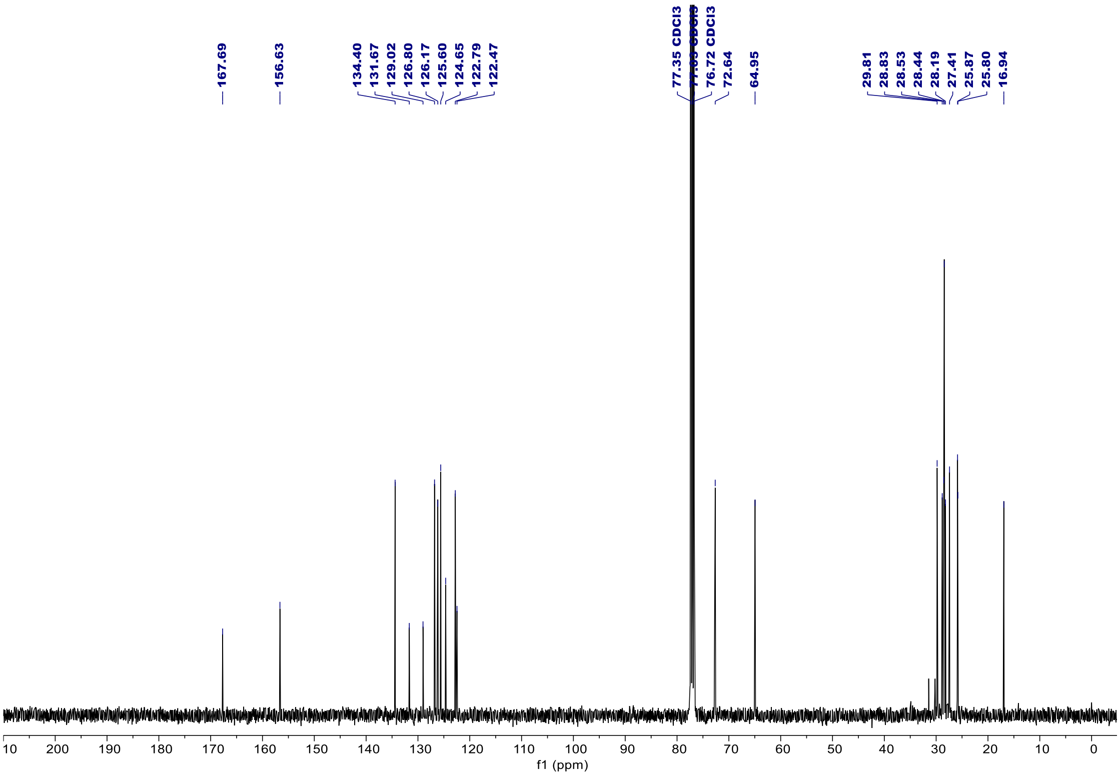

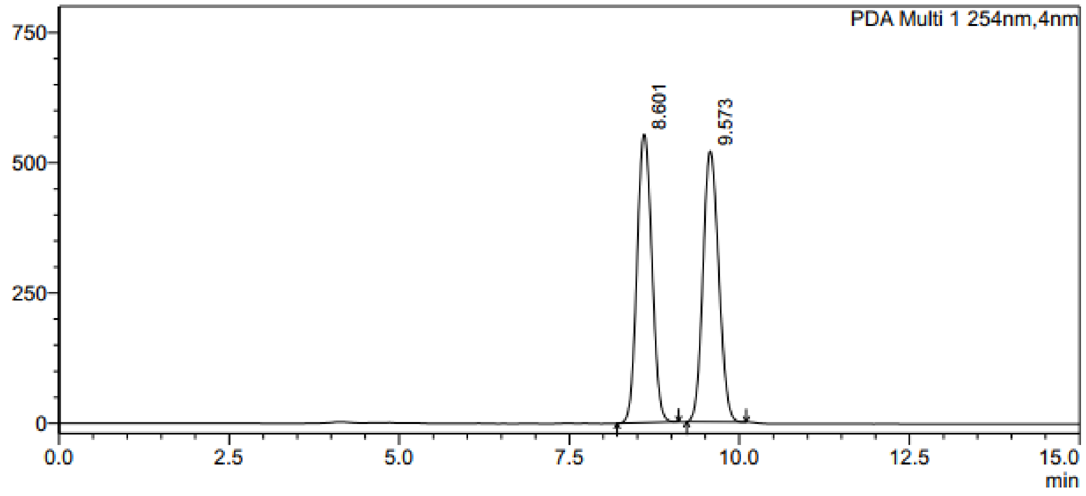


PDA Ch1 254nm

| Peak# | Ret. Time | Area | Area% | Height |
|---|---|---|---|---|
| 1 | 8.601 | 8360196 | 49.858 | 553741 |
| 2 | 9.573 | 8407831 | 50.142 | 519799 |
| Total | | 16768027 | 100.000 | 1073540 |

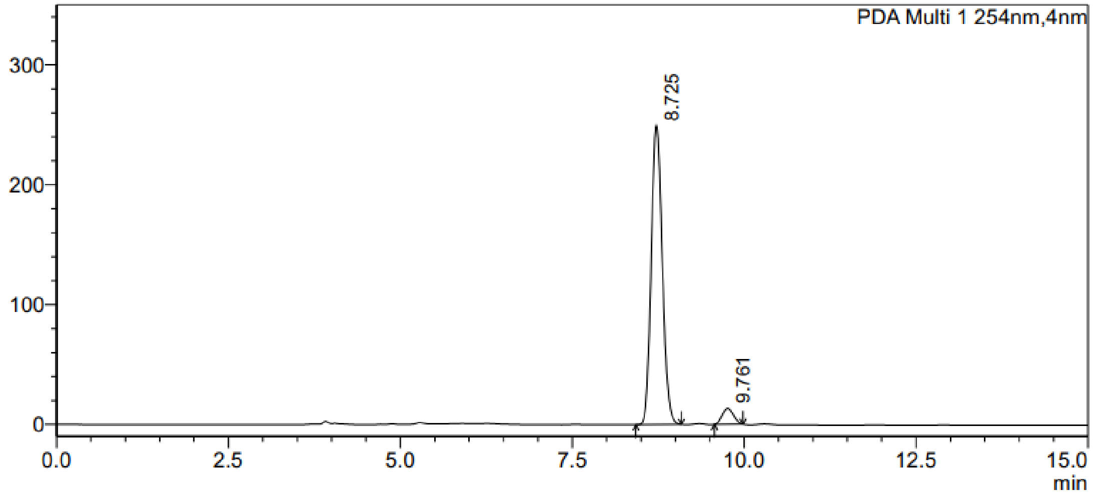


PDA Ch1 254nm

| Peak# | Ret. Time | Area | Area% | Height |
|---|---|---|---|---|
| 1 | 8.725 | 2795359 | 94.866 | 249135 |
| 2 | 9.761 | 151283 | 5.134 | 13311 |
| Total | | 2946642 | 100.000 | 262446 |

**Supplementary Figure 60.** $^{1}$H, $^{13}$C and HPLC spectra of **2n**

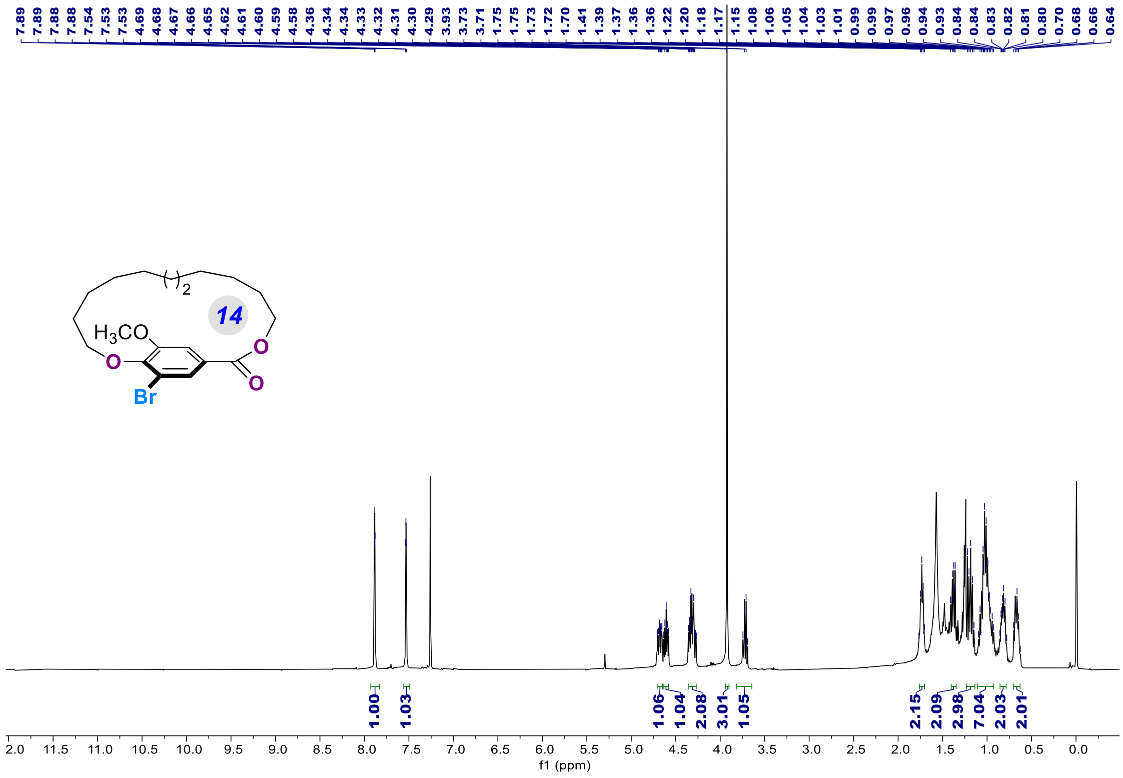


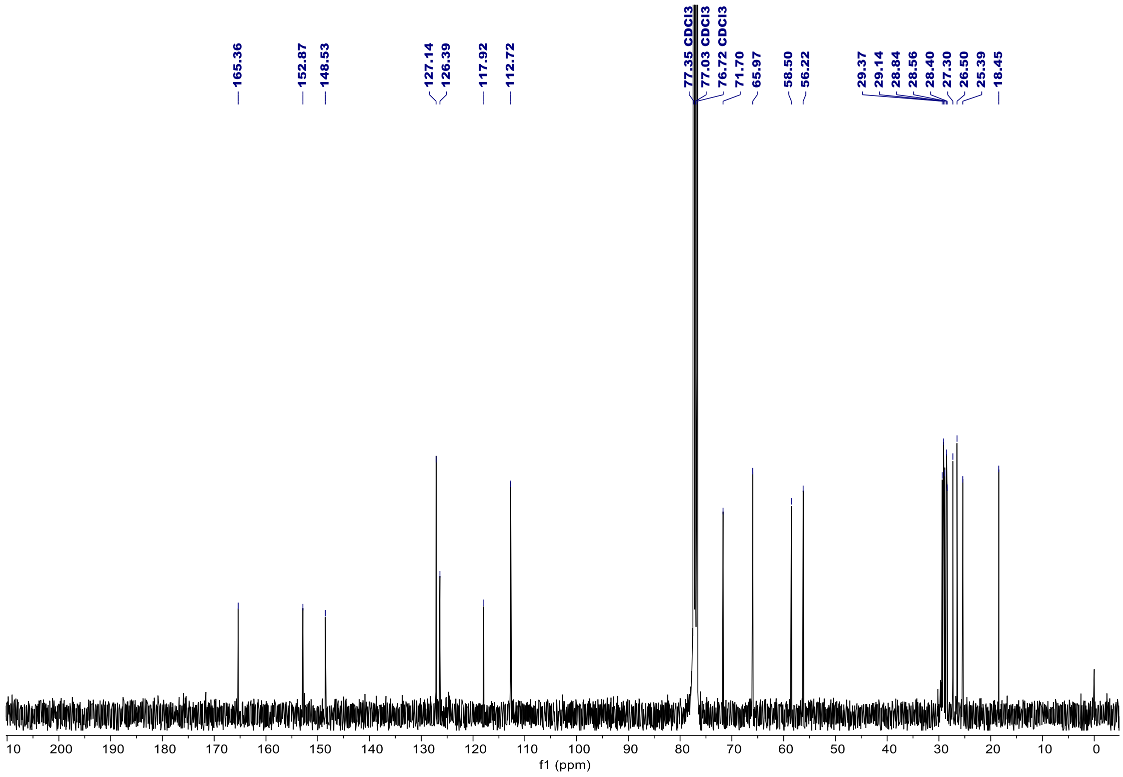

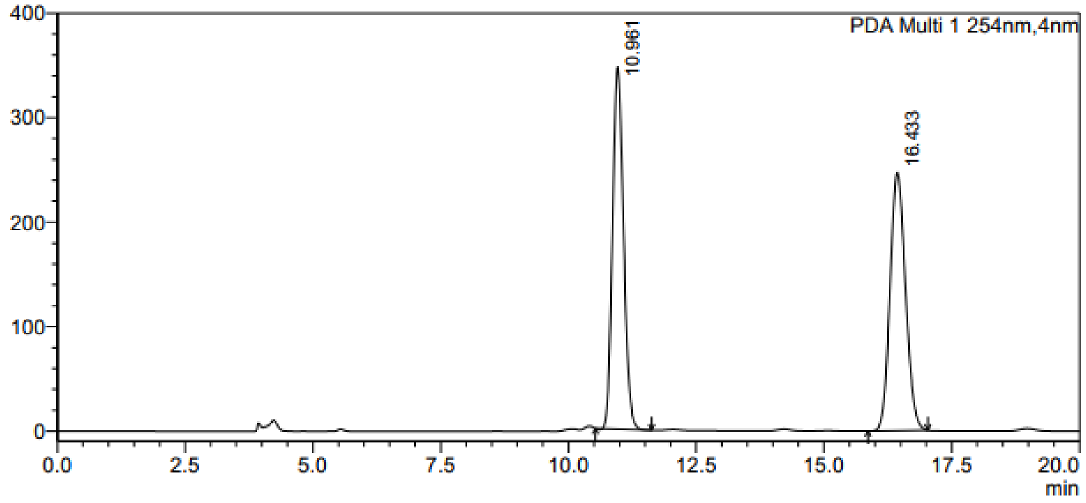


PDA Ch1 254nm

| Peak# | Ret. Time | Area | Area% | Height |
|---|---|---|---|---|
| 1 | 10.961 | 5199708 | 49.863 | 346231 |
| 2 | 16.433 | 5228338 | 50.137 | 246752 |
| Total | | 10428046 | 100.000 | 592983 |

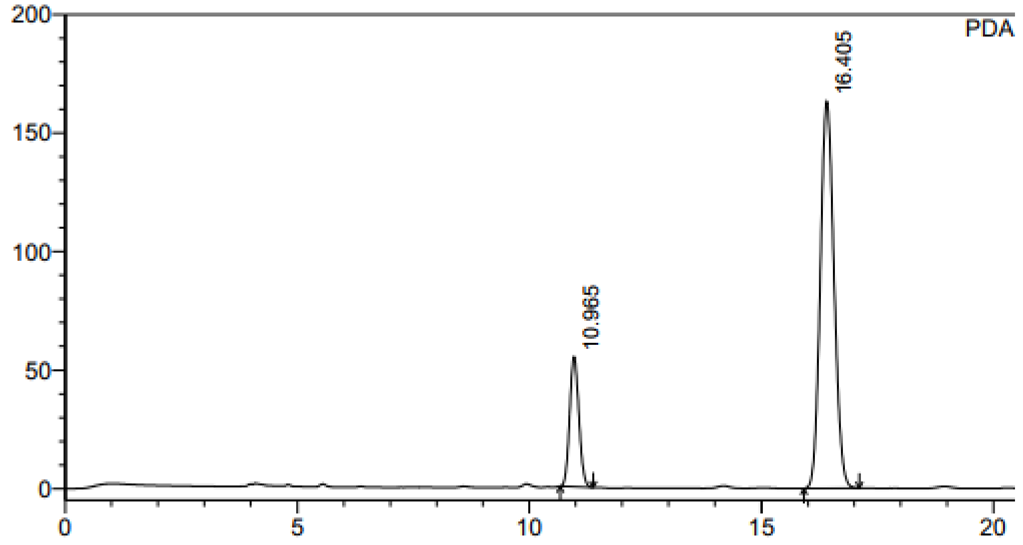


PDA Ch1 254nm

| Peak# | Ret. Time | Area | Area% | Height |
|---|---|---|---|---|
| 1 | 10.965 | 760136 | 18.471 | 54767 |
| 2 | 16.405 | 3355198 | 81.529 | 163041 |
| Total | | 4115334 | 100.000 | 217808 |

**Supplementary Figure 61.** $^{1}H$, $^{13}C$ and HPLC spectra of **2o**

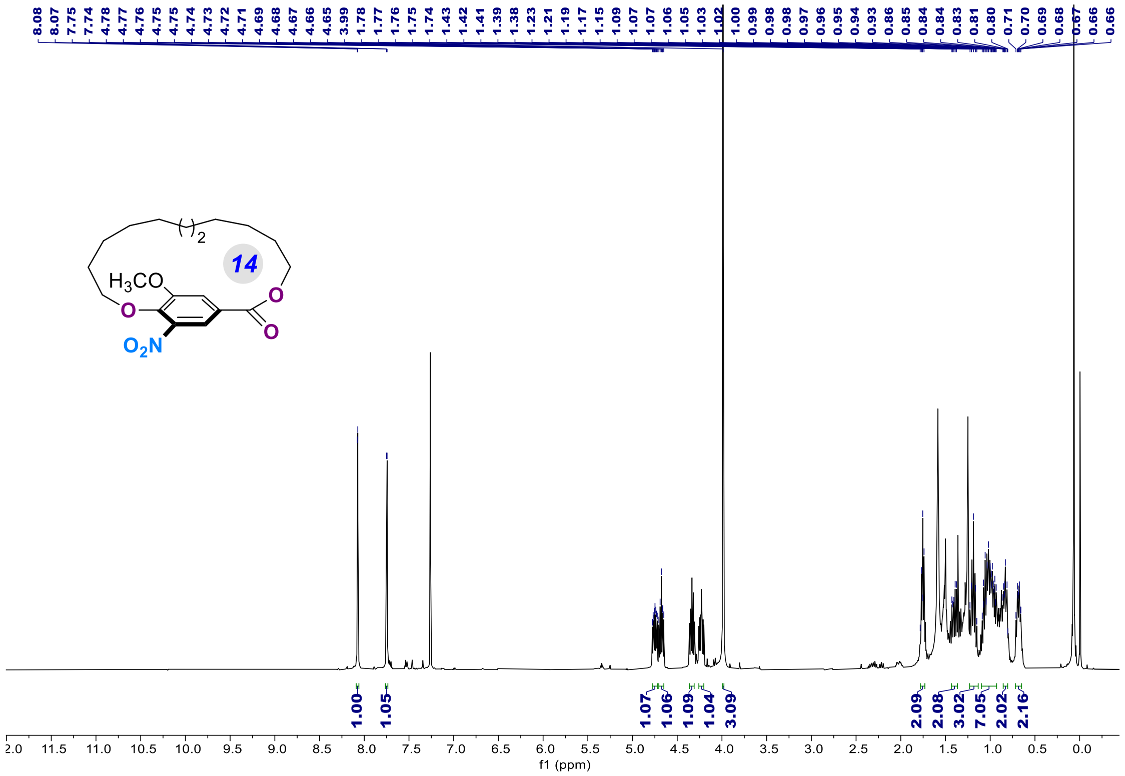


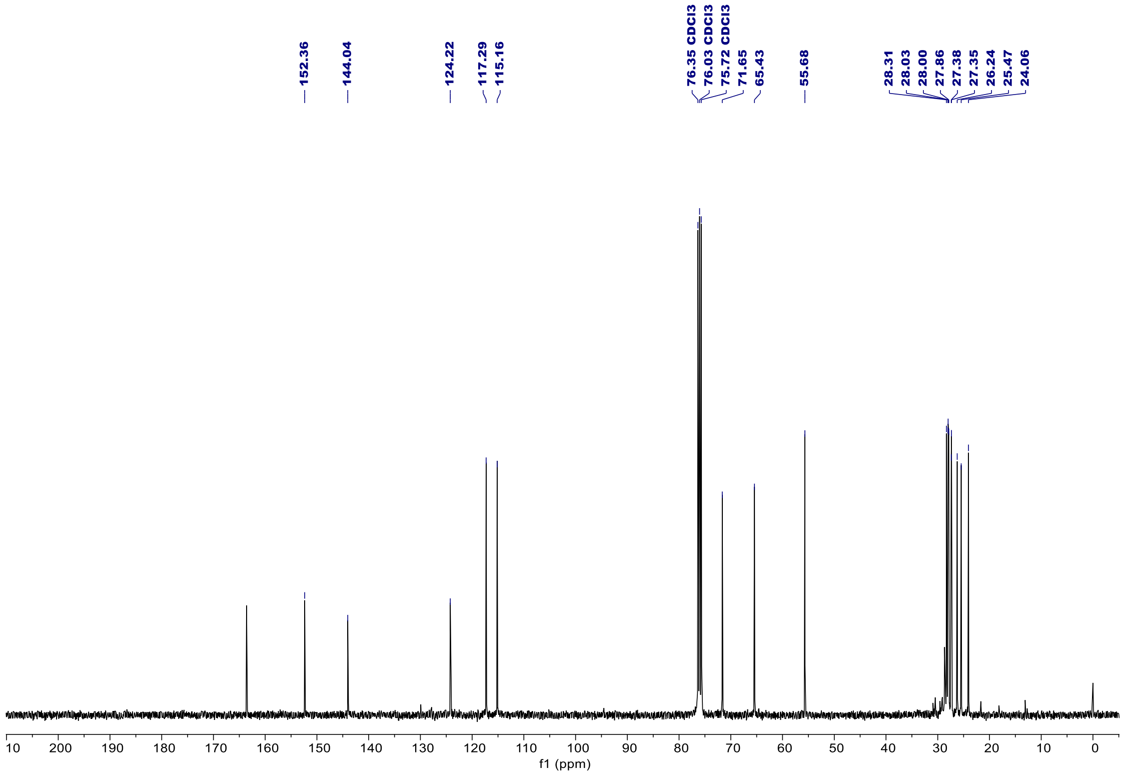

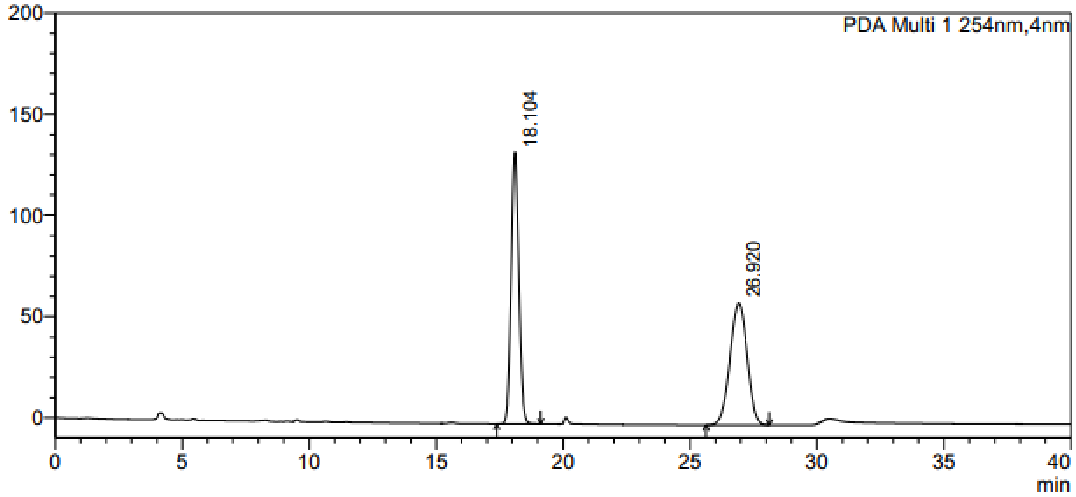


PDA Ch1 254nm

| Peak# | Ret. Time | Area | Area% | Height |
|---|---|---|---|---|
| 1 | 18.104 | 2842023 | 50.030 | 134109 |
| 2 | 26.920 | 2838600 | 49.970 | 60259 |
| Total | | 5680623 | 100.000 | 194368 |

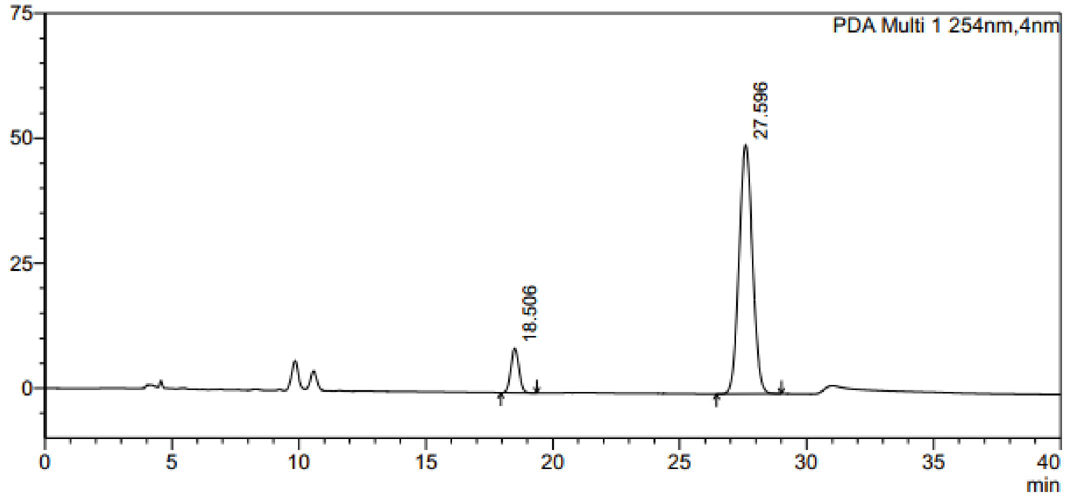


PDA Ch1 254nm

| Peak# | Ret. Time | Area | Area% | Height |
|---|---|---|---|---|
| 1 | 18.506 | 197844 | 9.736 | 8921 |
| 2 | 27.596 | 1834302 | 90.264 | 49747 |
| Total | | 2032147 | 100.000 | 58668 |

**Supplementary Figure 62.** $^{1}H$, $^{13}C$ and HPLC spectra of **2p**

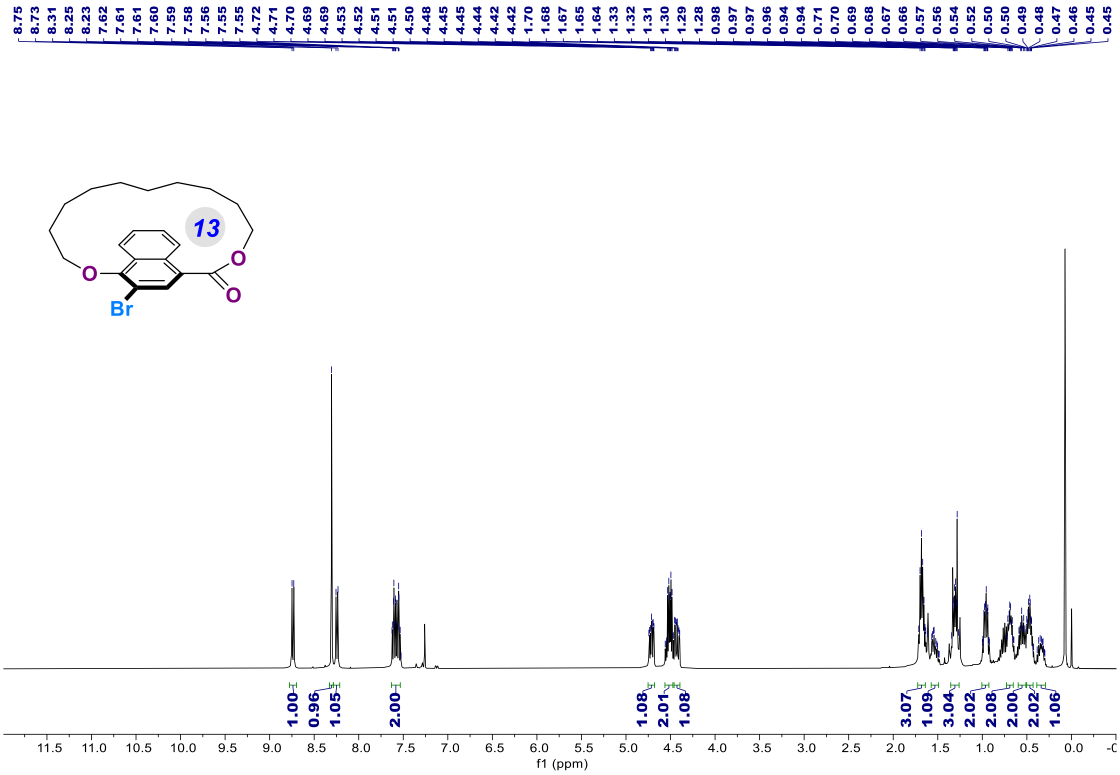


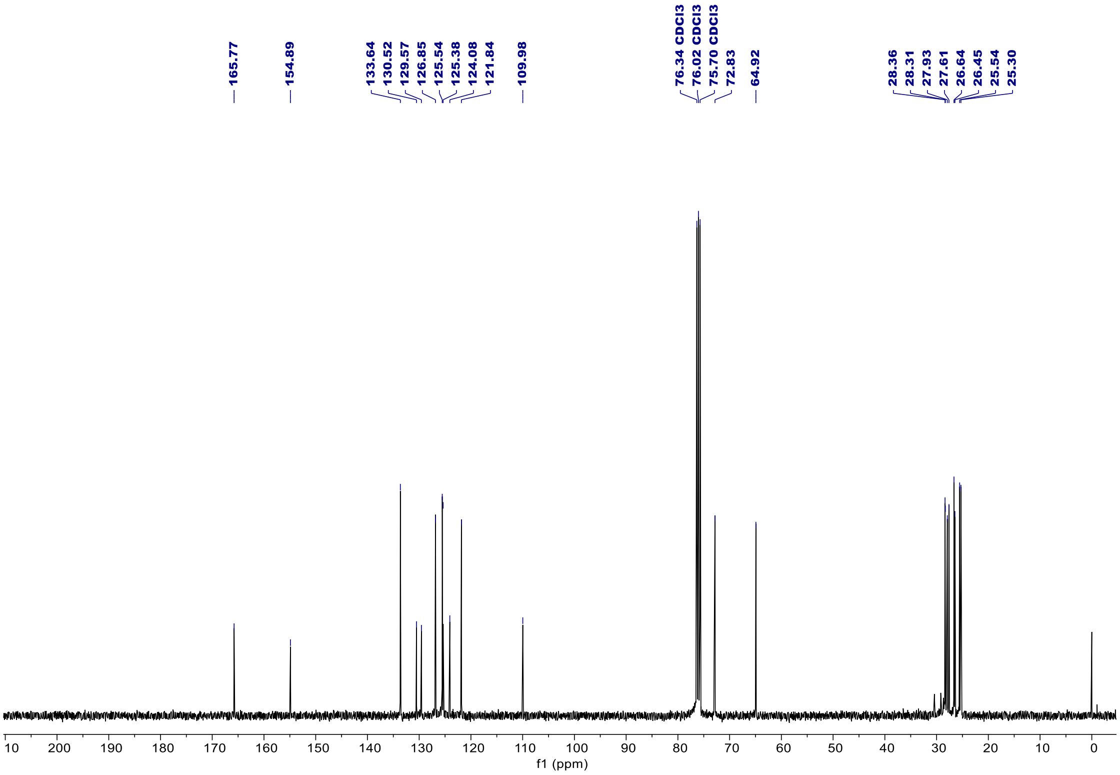

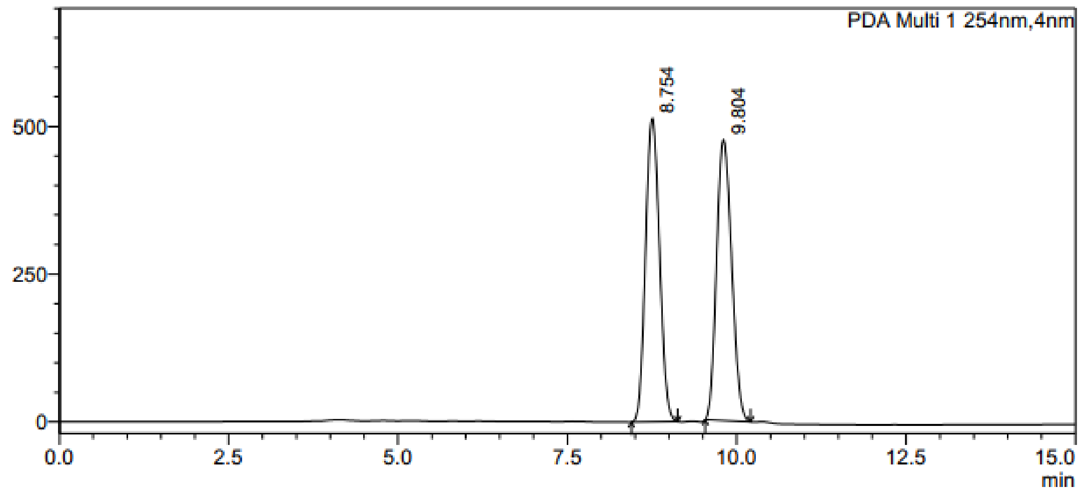


PDA Ch1 254nm

| Peak# | Ret. Time | Area | Area% | Height |
|---|---|---|---|---|
| 1 | 8.754 | 7370996 | 50.002 | 512996 |
| 2 | 9.804 | 7370444 | 49.998 | 475043 |
| Total | | 14741440 | 100.000 | 988039 |

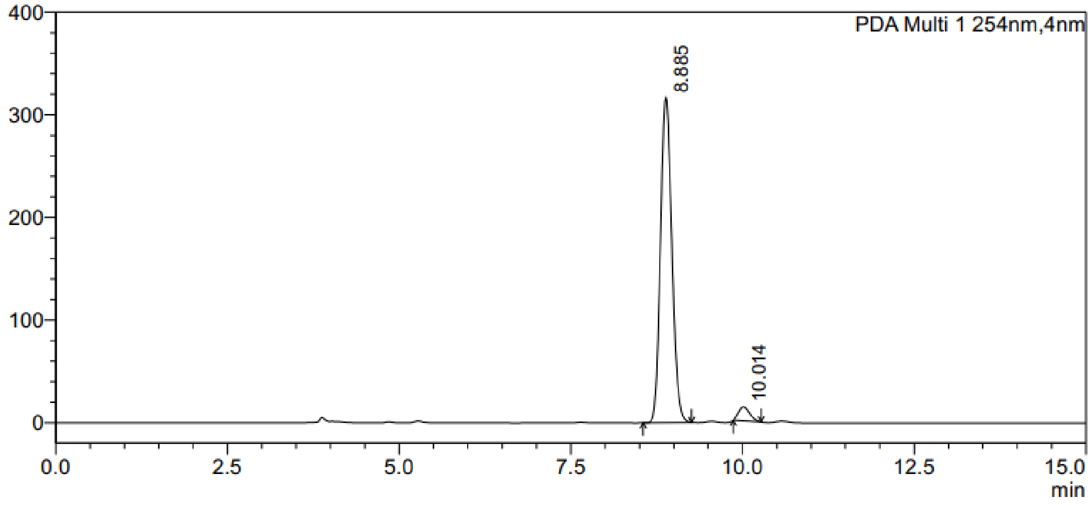


PDA Ch1 254nm

| Peak# | Ret. Time | Area | Area% | Height |
|---|---|---|---|---|
| 1 | 8.885 | 3642952 | 95.999 | 316372 |
| 2 | 10.014 | 151822 | 4.001 | 13477 |
| Total | | 3794774 | 100.000 | 329848 |

**Supplementary Figure 63.** $^{1}$H, $^{13}$C and HPLC spectra of **2q**

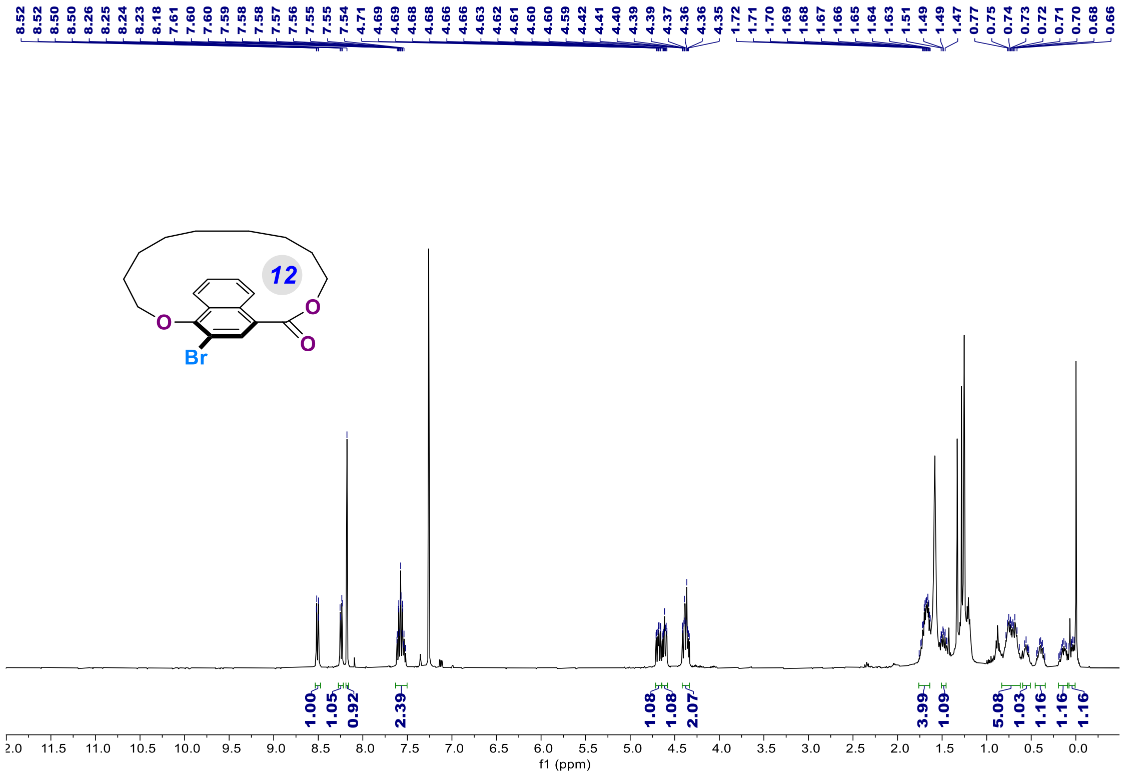


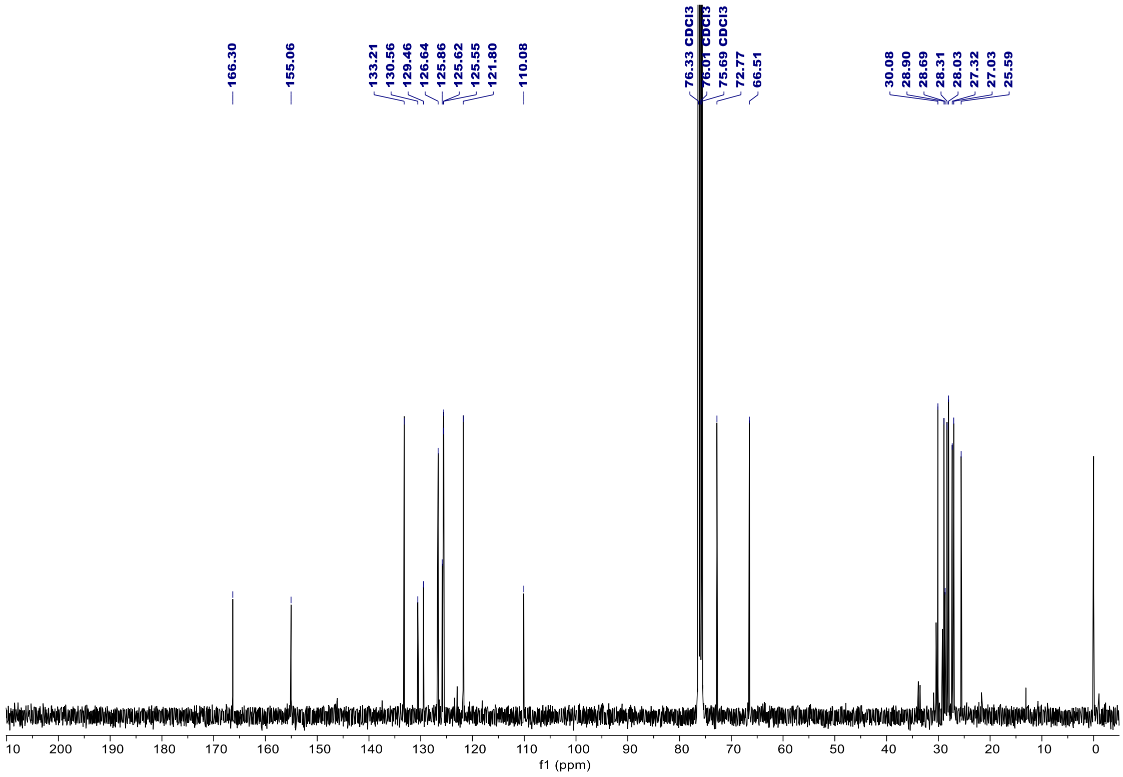

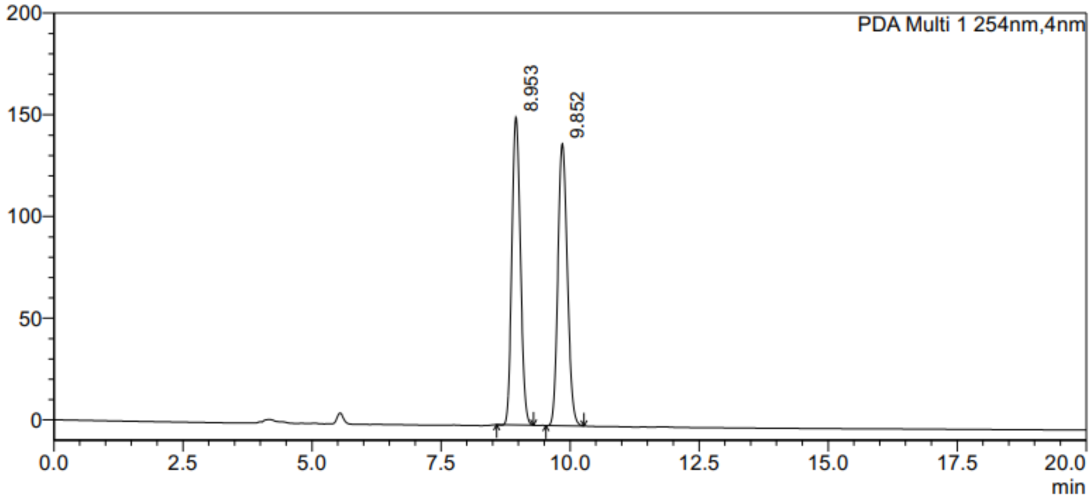


PDA Ch1 254nm

| Ret. Time | Area | Area% | Height |
|---|---|---|---|
| 8.953 | 1740309 | 49.693 | 151315 |
| 9.852 | 1761829 | 50.307 | 138628 |
| | 3502137 | 100.000 | 289943 |

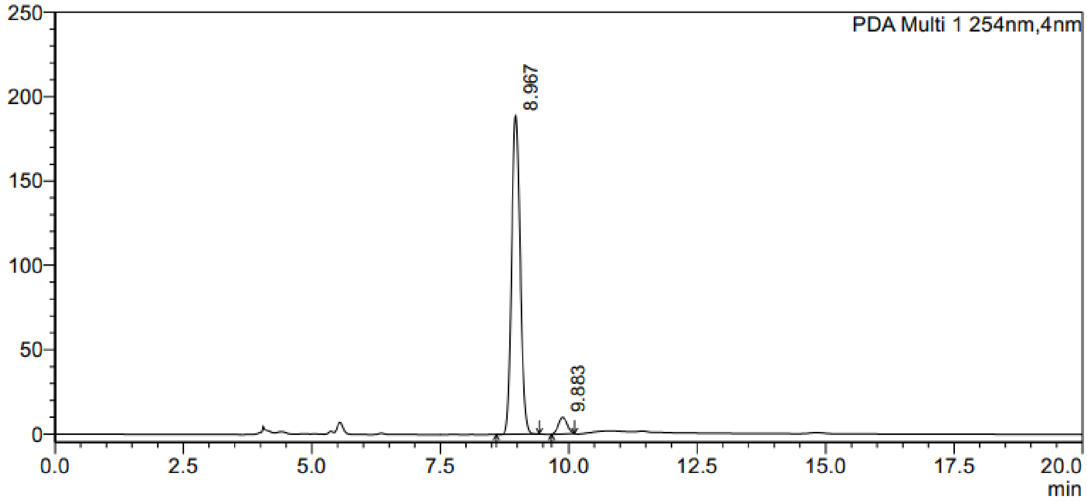


PDA Ch1 254nm

| Peak# | Ret. Time | Area | Area% | Height |
|---|---|---|---|---|
| 1 | 8.967 | 2171682 | 94.882 | 188801 |
| 2 | 9.883 | 117136 | 5.118 | 9874 |
| Total | | 2288818 | 100.000 | 198675 |

**Supplementary Figure 64.** $^{1}$H, $^{13}$C and HPLC spectra of **2s**

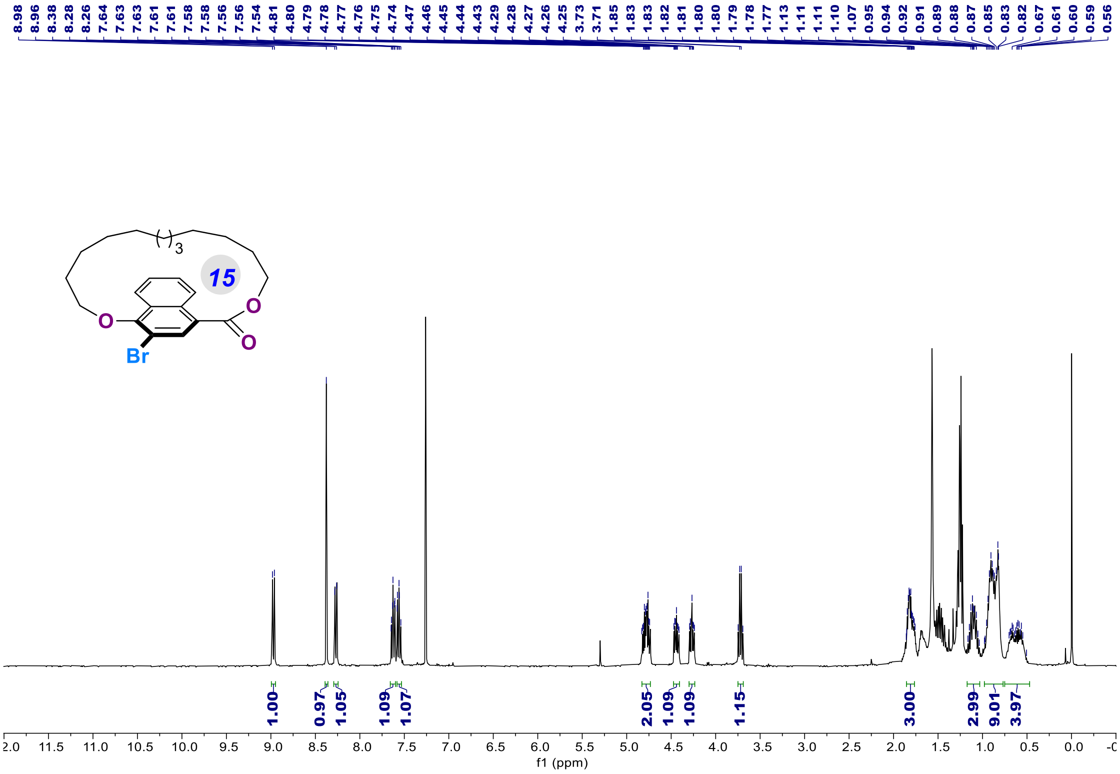


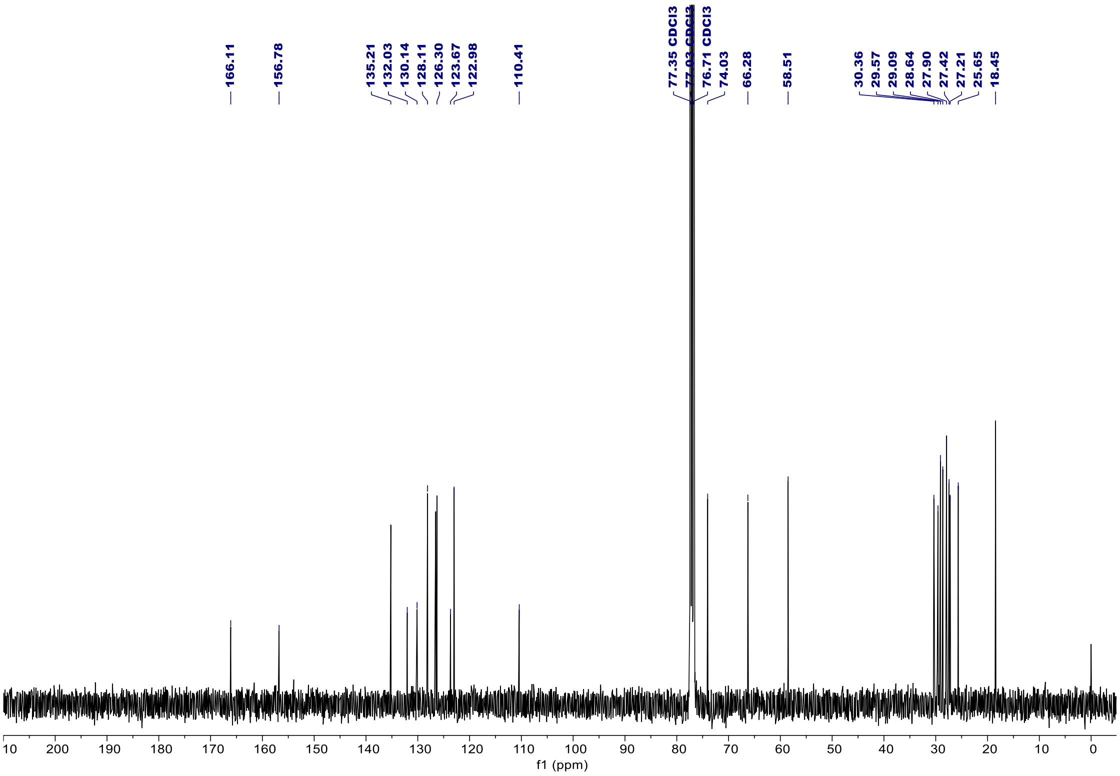

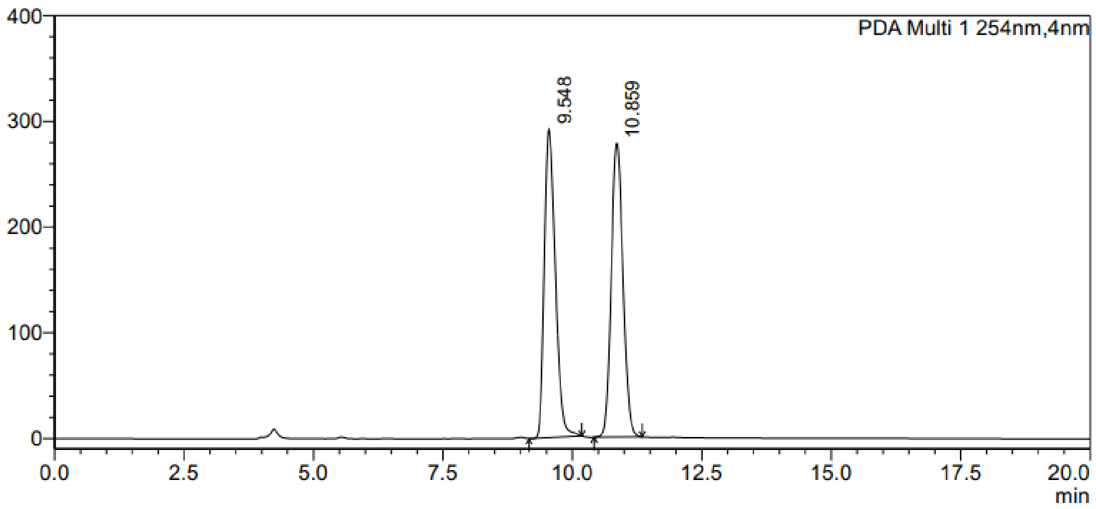


PDA Ch1 254nm

| Peak# | Ret. Time | Area | Area% | Height |
|---|---|---|---|---|
| 1 | 9.548 | 4334281 | 49.764 | 291367 |
| 2 | 10.859 | 4375420 | 50.236 | 277960 |
| Total | | 8709701 | 100.000 | 569328 |

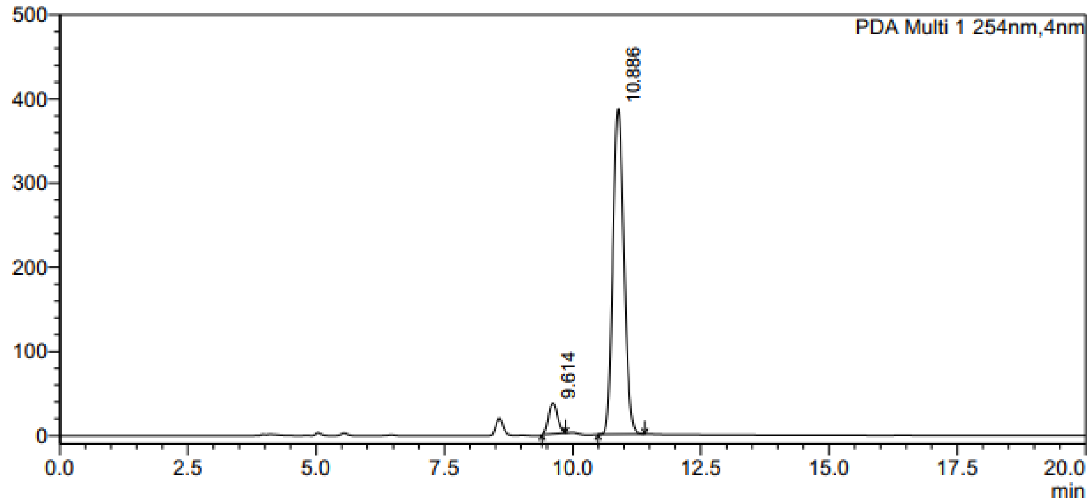


PDA Ch1 254nm

| Peak# | Ret. Time | Area | Area% | Height |
|---|---|---|---|---|
| 1 | 9.614 | 447962 | 7.126 | 36335 |
| 2 | 10.886 | 5838267 | 92.874 | 386093 |
| Total | | 6286229 | 100.000 | 422428 |

**Supplementary Figure 65.** $^{1}H$, $^{13}C$ and HPLC spectra of **2t**

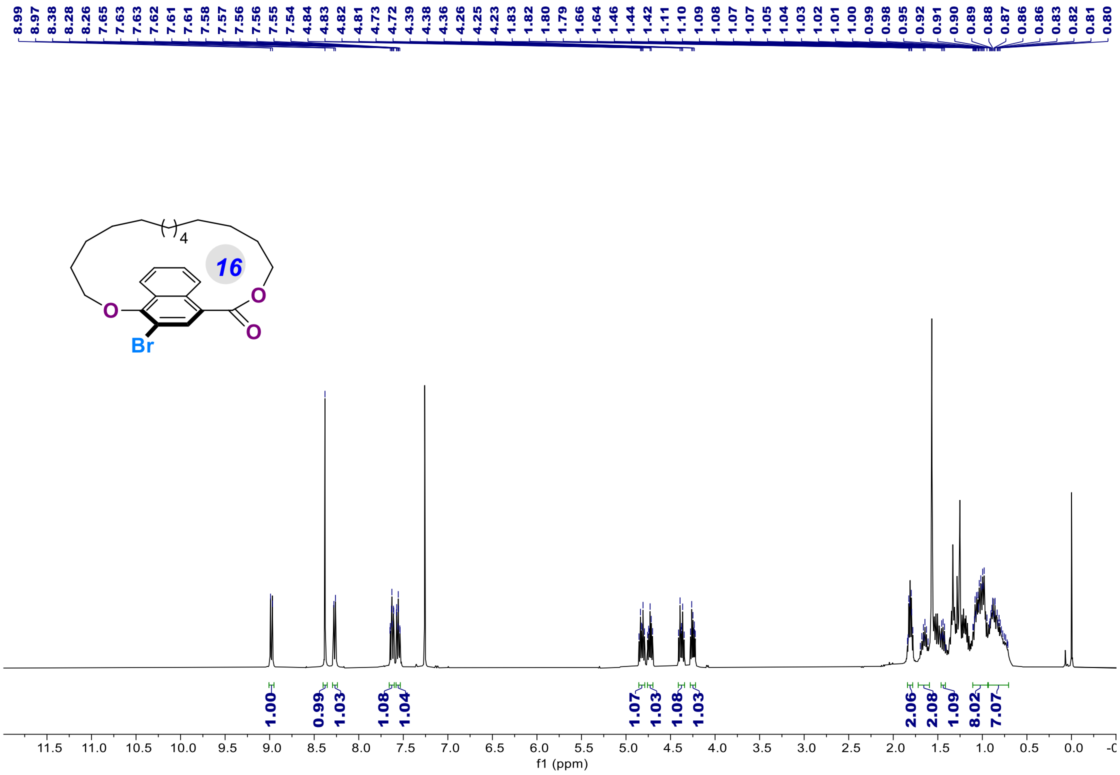

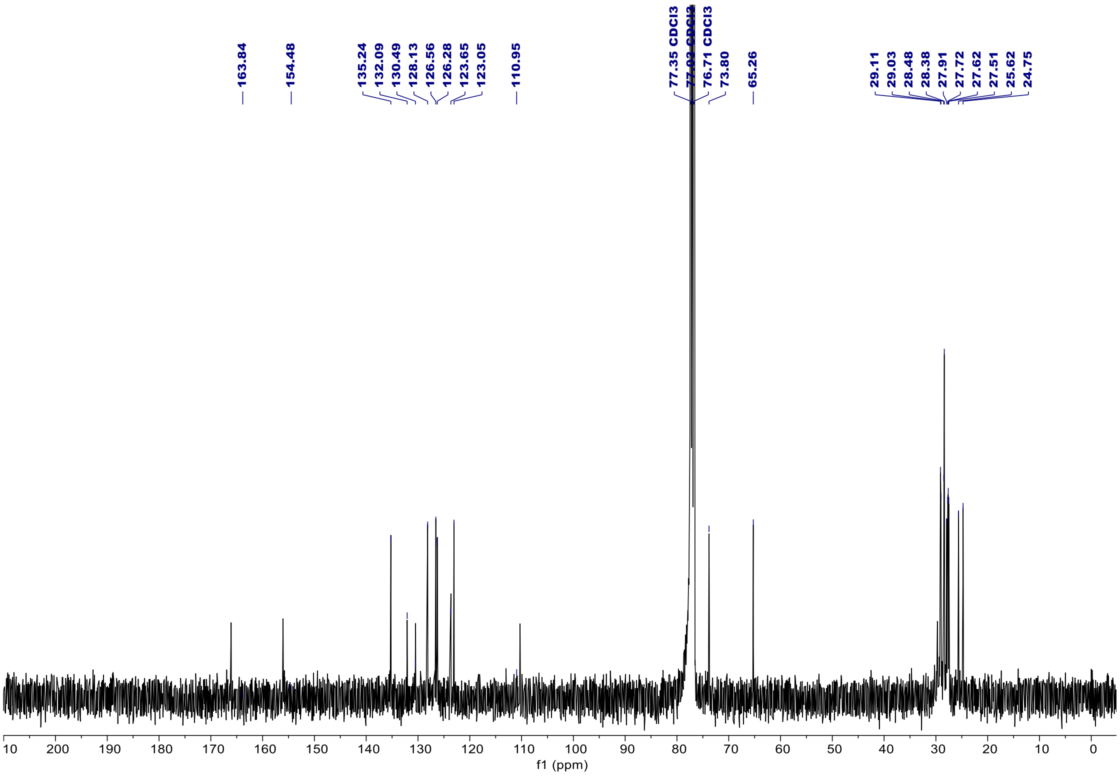

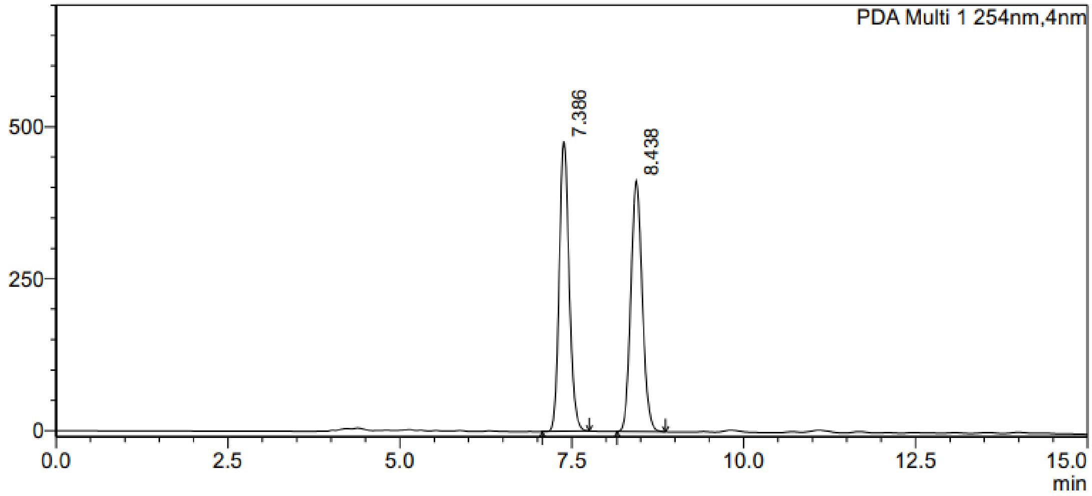


PDA Ch1 254nm

| Peak# | Ret. Time | Area | Area% | Height |
|---|---|---|---|---|
| 1 | 7.386 | 4811047 | 50.038 | 475747 |
| 2 | 8.438 | 4803791 | 49.962 | 412224 |
| Total | | 9614838 | 100.000 | 887971 |

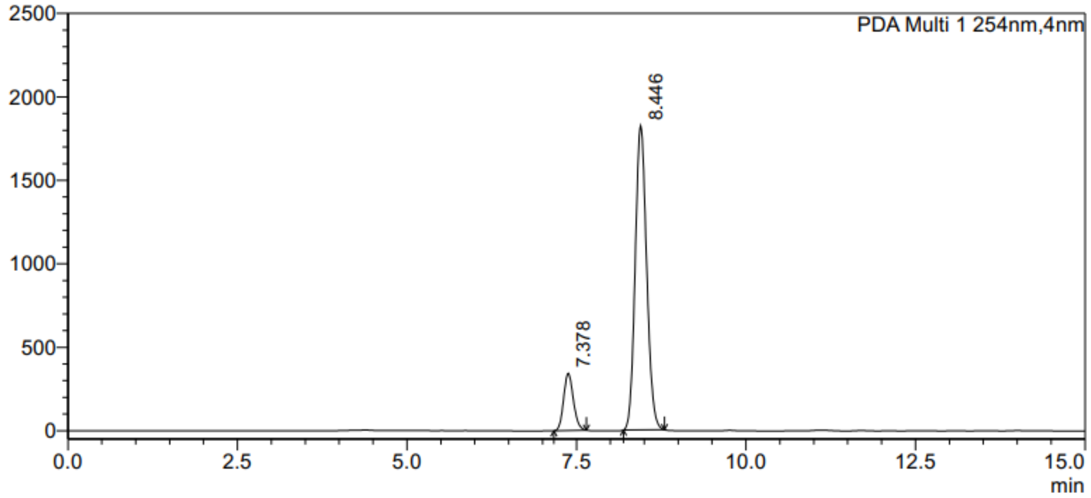


PDA Ch1 254nm

| Peak# | Ret. Time | Area | Area% | Height |
|---|---|---|---|---|
| 1 | 7.378 | 3445635 | 13.865 | 342702 |
| 2 | 8.446 | 21406395 | 86.135 | 1822823 |
| Total | | 24852030 | 100.000 | 2165524 |

**Supplementary Figure 66.** $^{1}H$, $^{13}C$ and HPLC spectra of **2u**

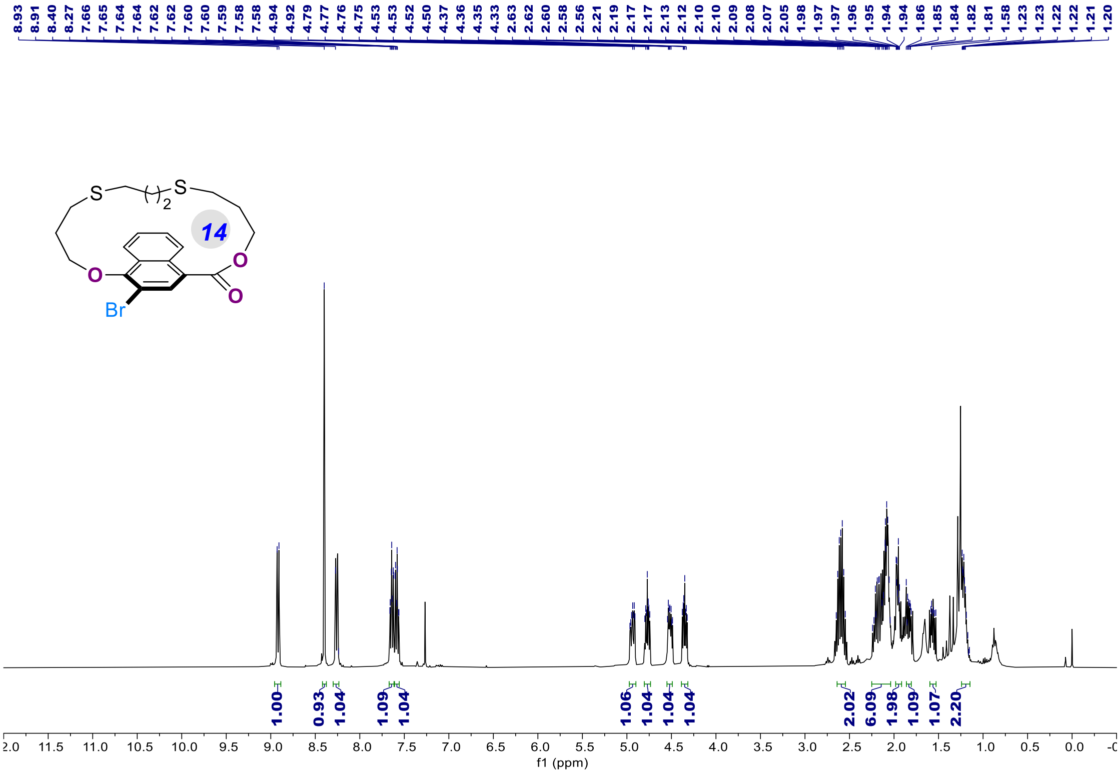

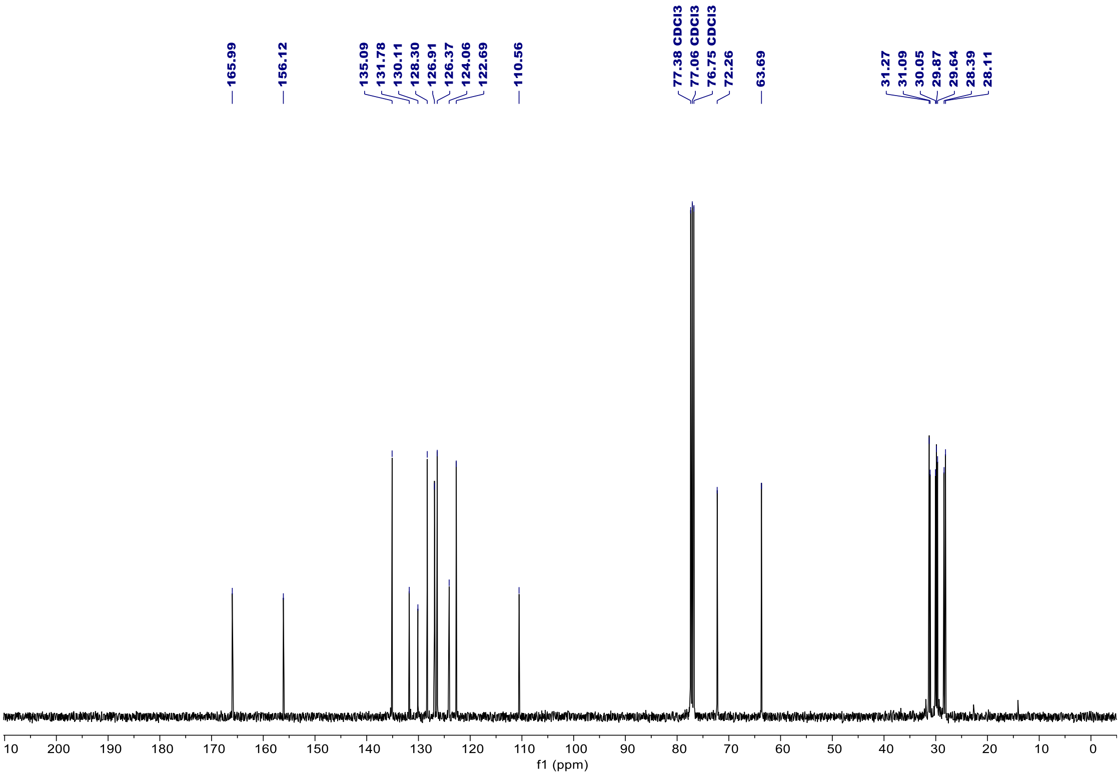

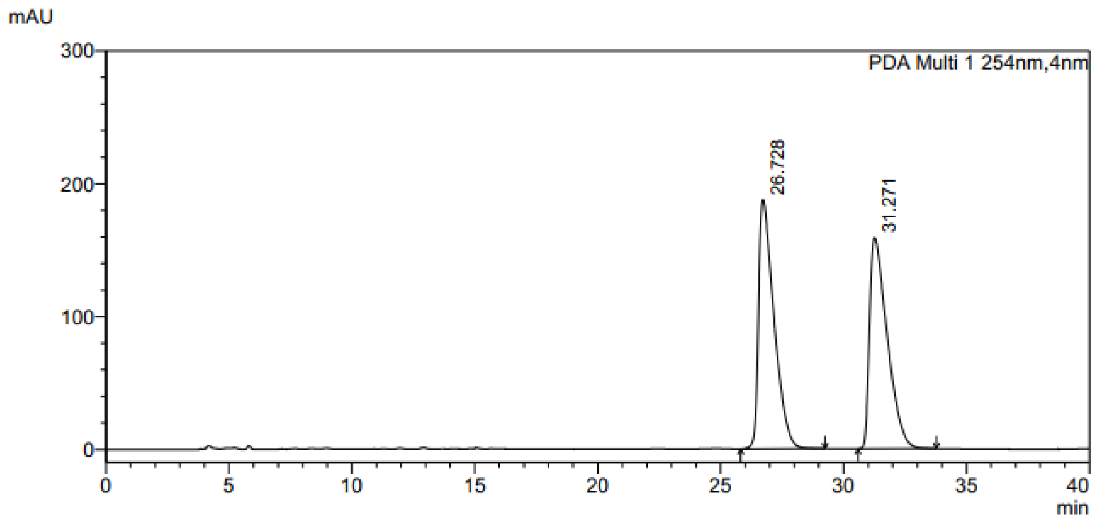


PDA Ch1 254nm

| Peak# | Ret. Time | Area | Area% | Height |
|---|---|---|---|---|
| 1 | 26.728 | 8217911 | 50.787 | 187514 |
| 2 | 31.271 | 7963188 | 49.213 | 158972 |
| Total | | 16181099 | 100.000 | 346486 |

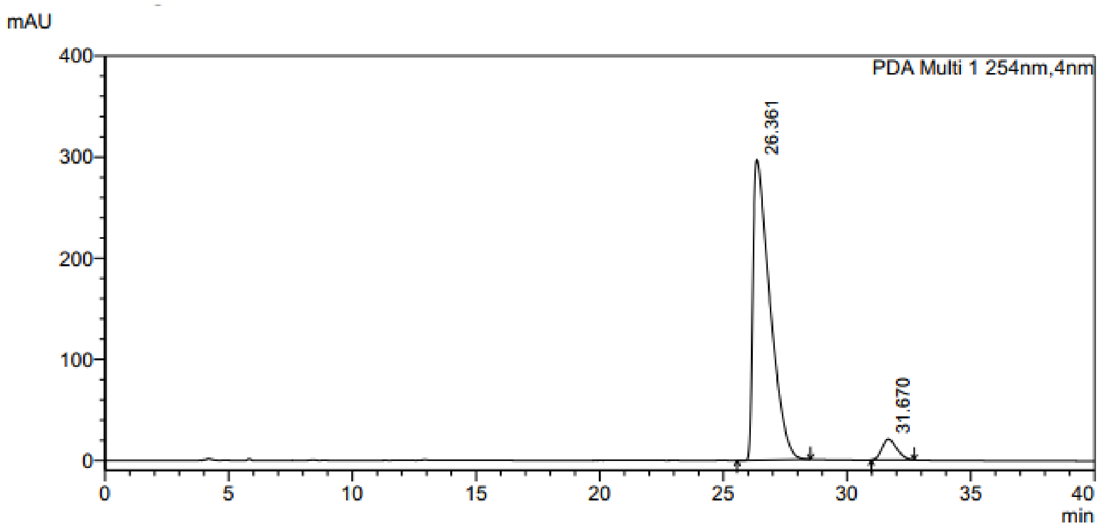


PDA Ch1 254nm

| Peak# | Ret. Time | Area | Area% | Height |
|---|---|---|---|---|
| 1 | 26.361 | 14213727 | 94.300 | 297125 |
| 2 | 31.670 | 859219 | 5.700 | 20446 |
| Total | | 15072945 | 100.000 | 317571 |

**Supplementary Figure 67.** $^{1}$H, $^{13}$C and HPLC spectra of **2v**

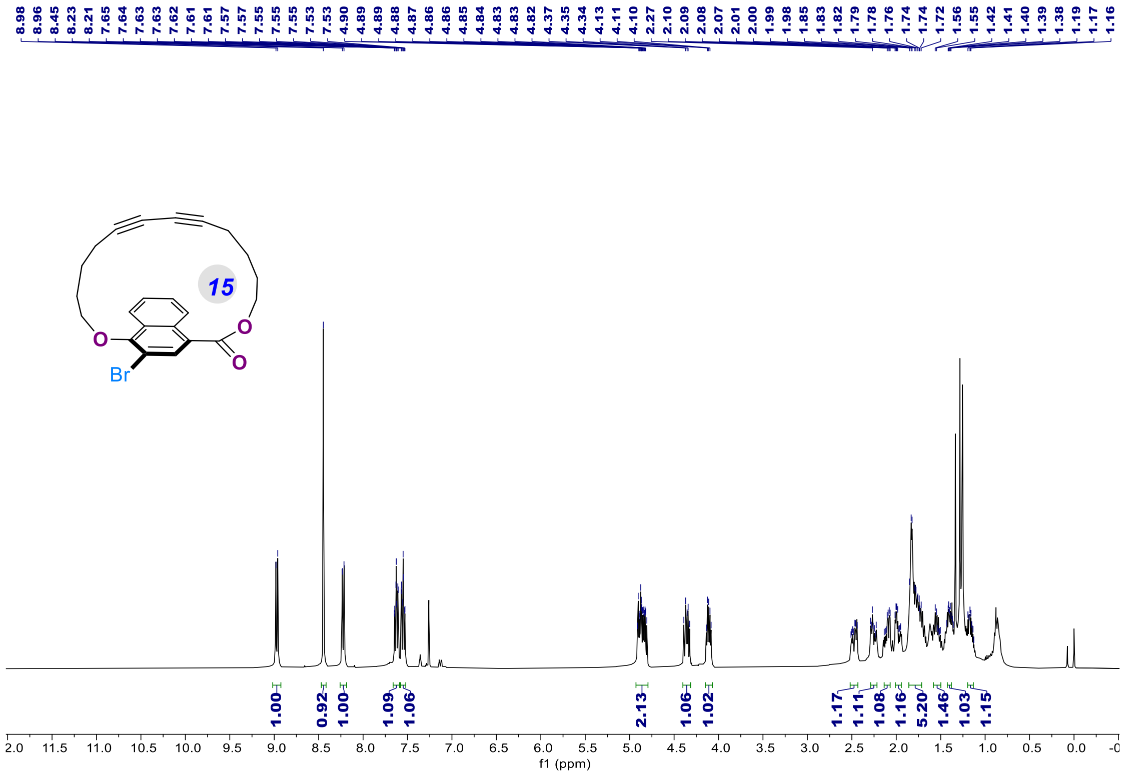


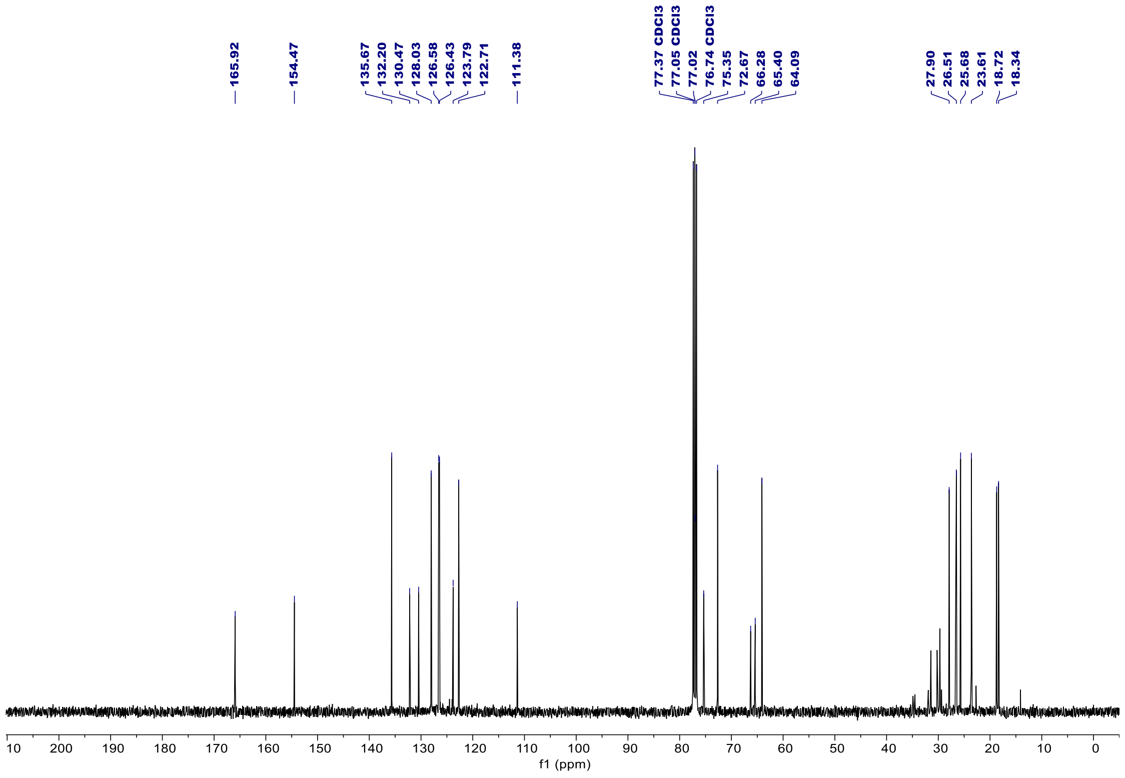

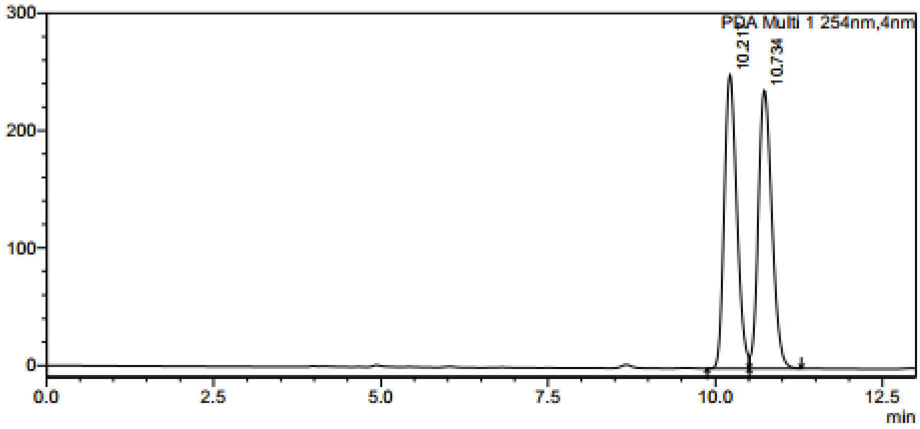


PDA Ch1 254nm

| Peak# | Ret. Time | Area | Area% | Height |
|---|---|---|---|---|
| 1 | 10.217 | 3290182 | 49.627 | 249836 |
| 2 | 10.734 | 3339646 | 50.373 | 236502 |
| Total | | 6629828 | 100.000 | 486338 |

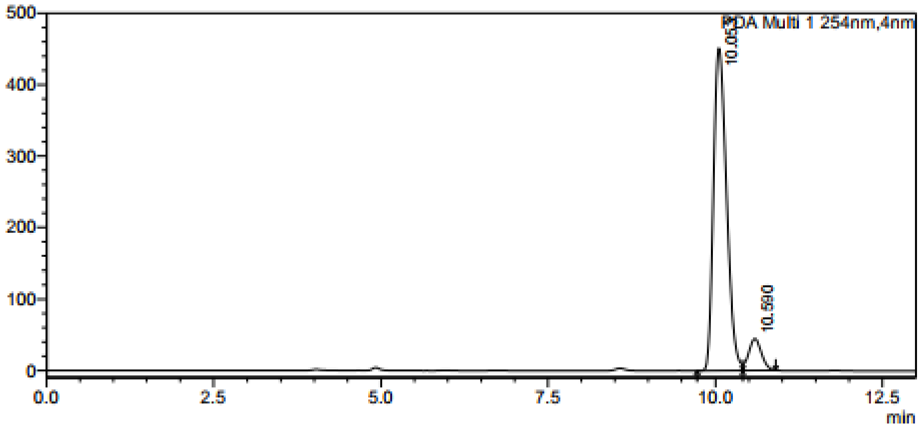


PDA Ch1 254nm

| Peak# | Ret. Time | Area | Area% | Height |
|---|---|---|---|---|
| 1 | 10.053 | 6118817 | 91.205 | 450865 |
| 2 | 10.590 | 590067 | 8.795 | 44368 |
| Total | | 6708885 | 100.000 | 495233 |

**Supplementary Figure 68.** $^{1}$H, $^{13}$C and HPLC spectra of **2w**

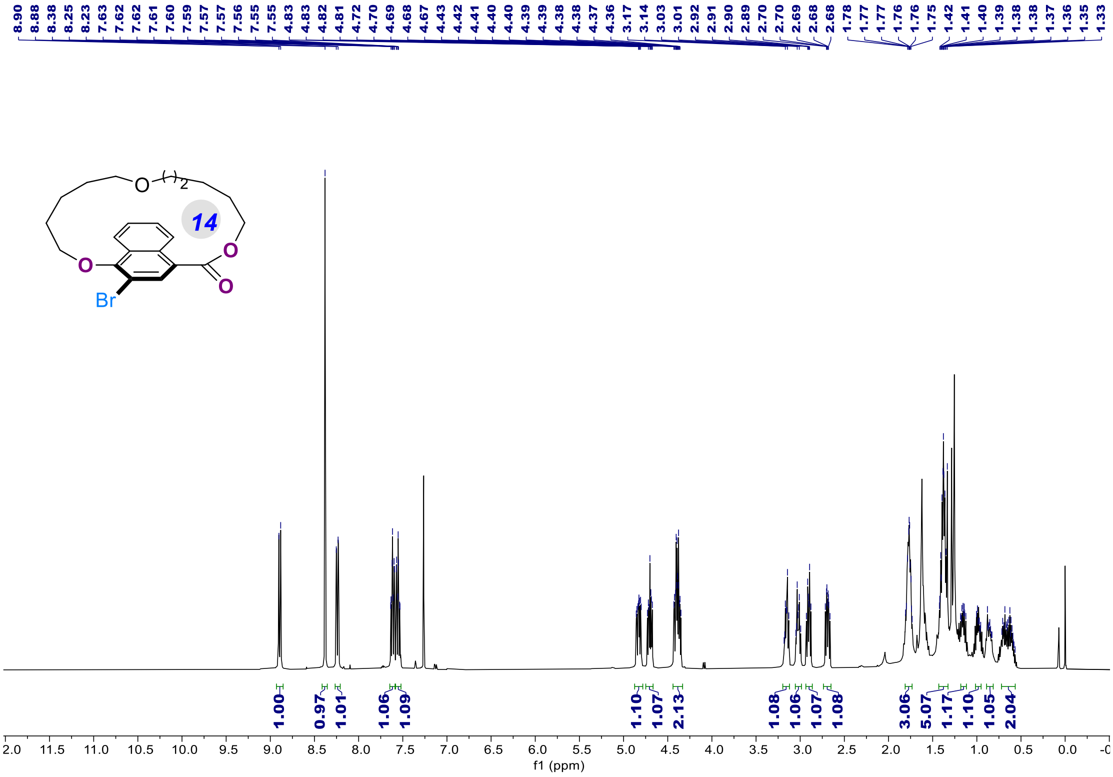


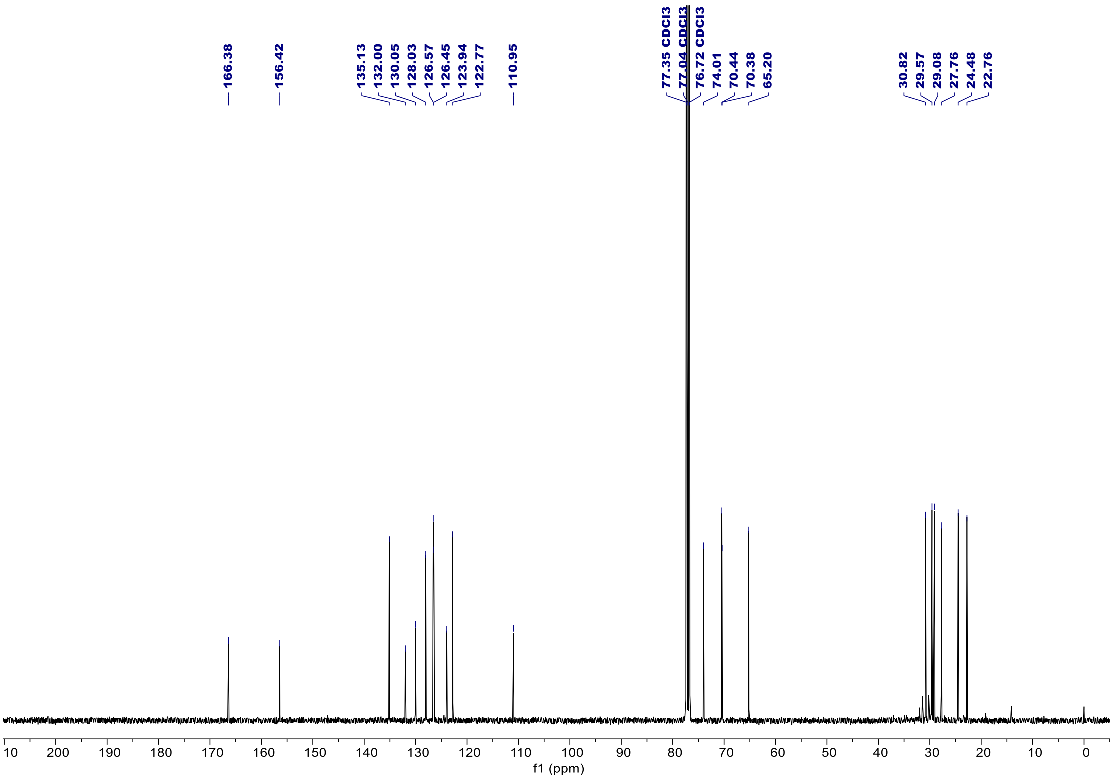

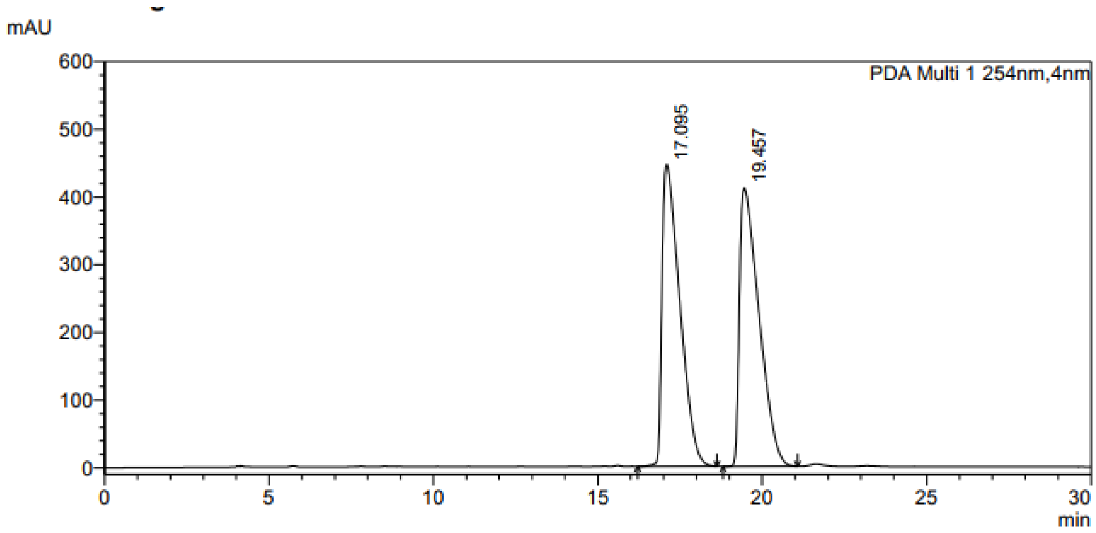


PDA Ch1 254nm

| Peak# | Ret. Time | Area | Area% | Height |
|---|---|---|---|---|
| 1 | 17.095 | 16777884 | 49.679 | 445792 |
| 2 | 19.457 | 16994876 | 50.321 | 410879 |
| Total | | 33772760 | 100.000 | 856670 |

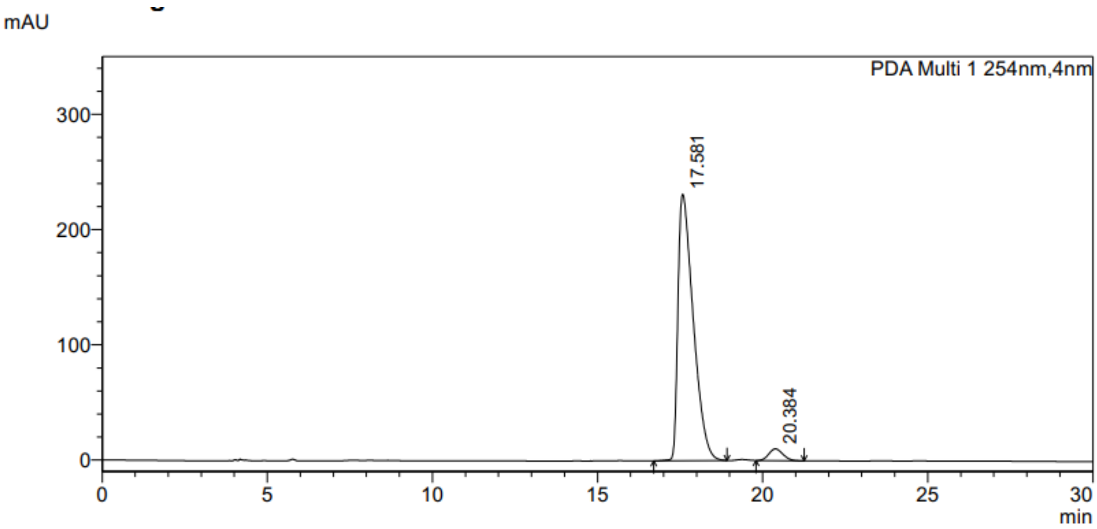


PDA Ch1 254nm

| Peak# | Ret. Time | Area | Area% | Height |
|---|---|---|---|---|
| 1 | 17.581 | 7436699 | 96.092 | 231255 |
| 2 | 20.384 | 302467 | 3.908 | 10210 |
| Total | | 7739165 | 100.000 | 241465 |

**Supplementary Figure 69.** $^{1}$H, $^{13}$C and HPLC spectra of **7**

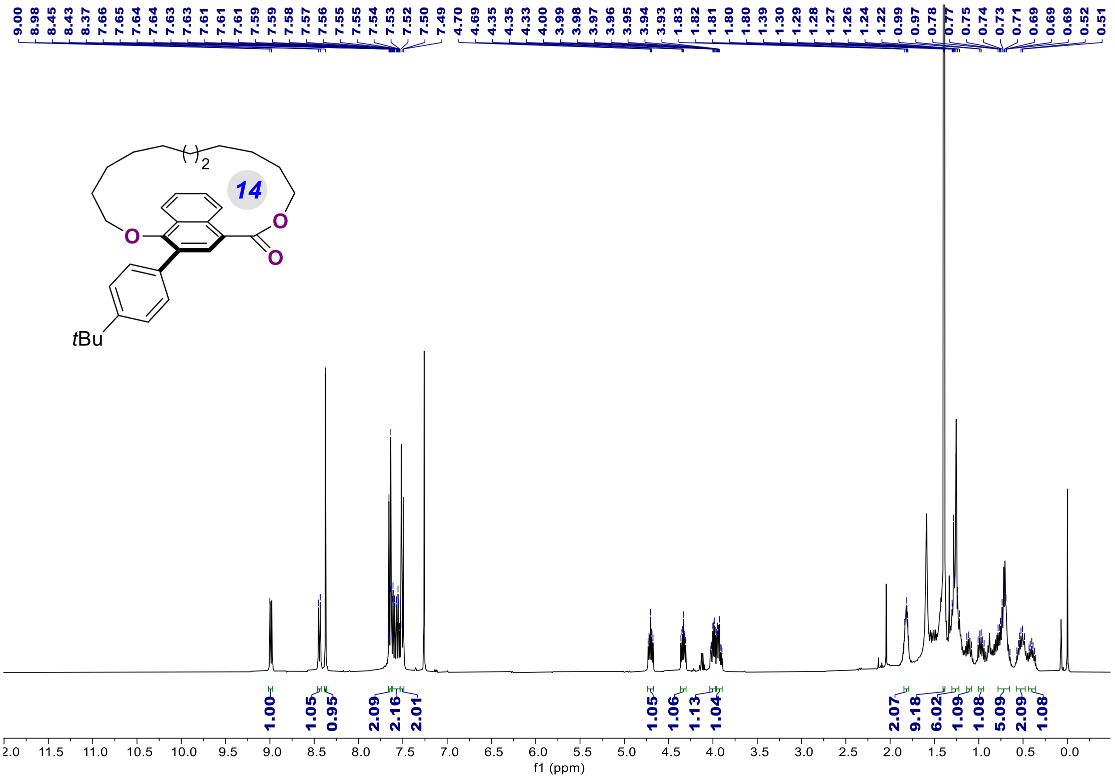


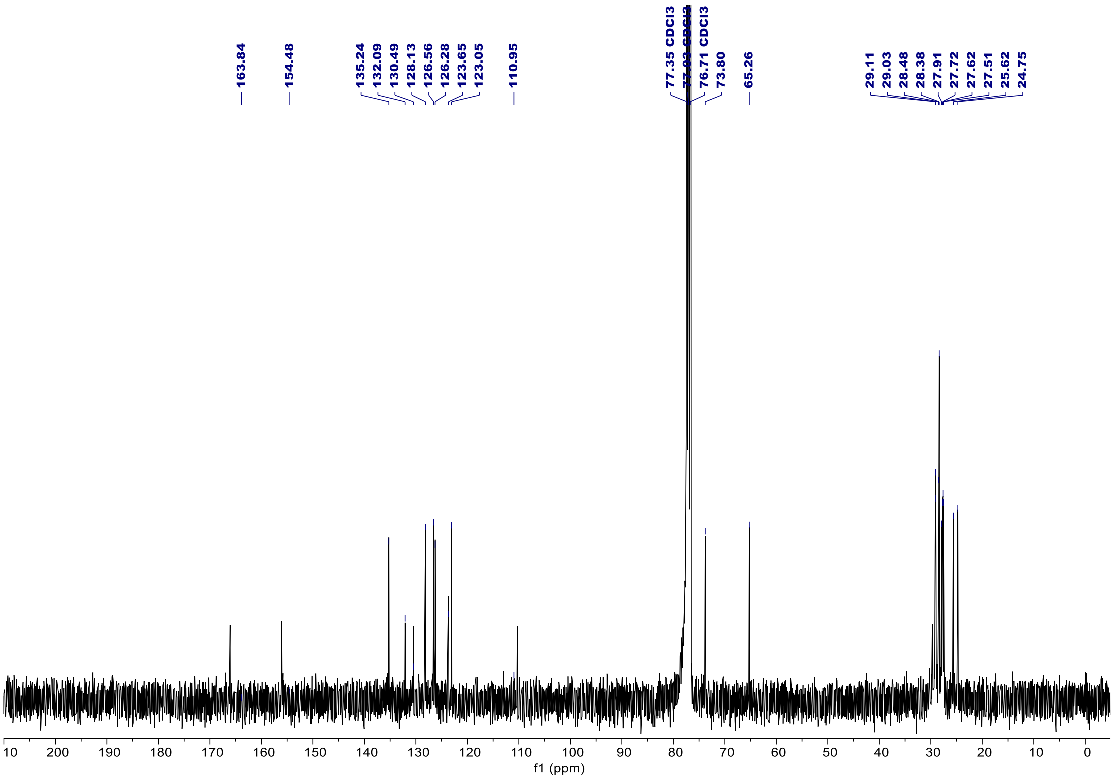

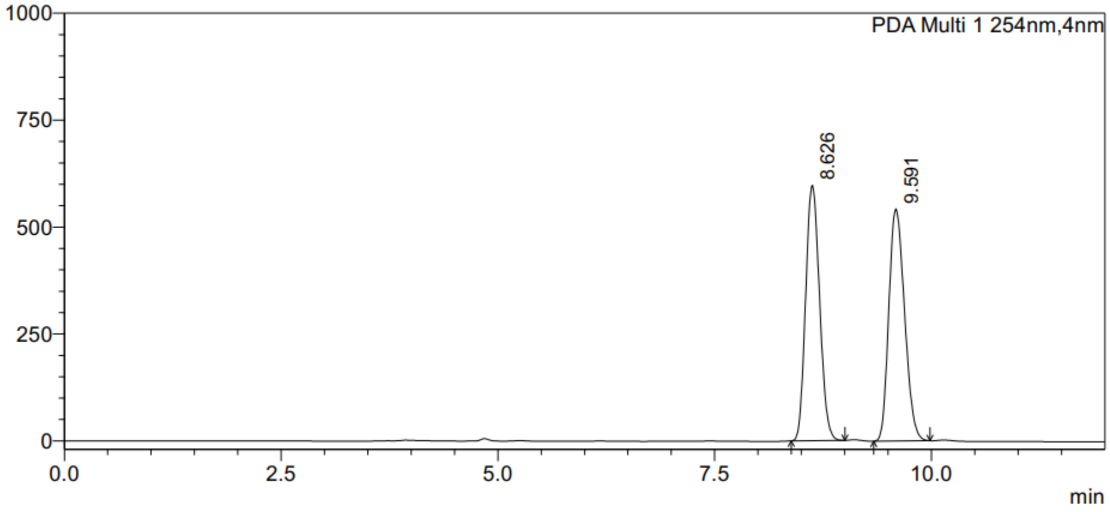


PDA Ch1 254nm

| Peak# | Ret. Time | Area | Area% | Height |
|---|---|---|---|---|
| 1 | 8.626 | 6677291 | 49.374 | 596775 |
| 2 | 9.591 | 6846697 | 50.626 | 542438 |
| Total | | 13523988 | 100.000 | 1139213 |

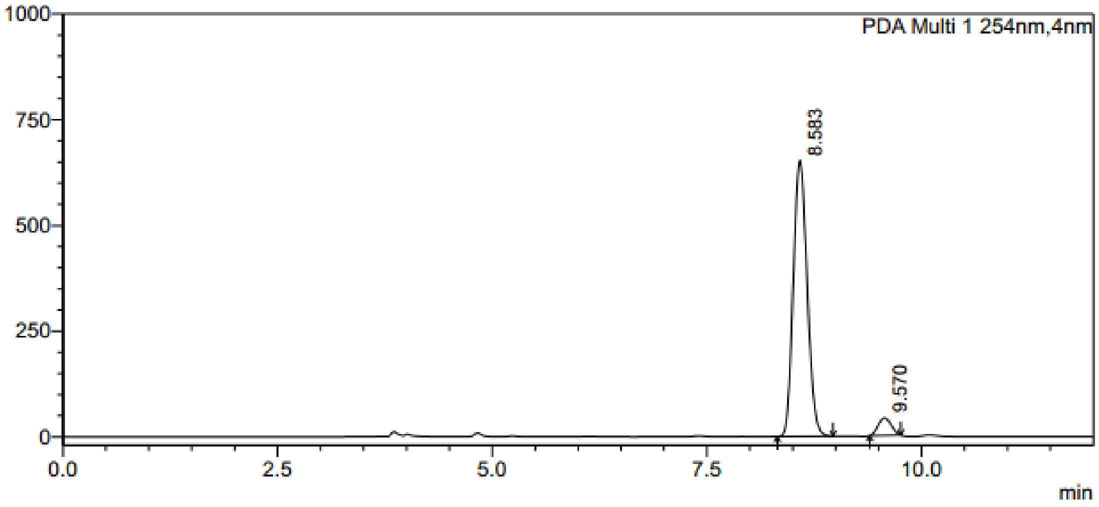


PDA Ch1 254nm

| Peak# | Ret. Time | Area | Area% | Height |
|---|---|---|---|---|
| 1 | 8.583 | 7327148 | 94.363 | 651287 |
| 2 | 9.570 | 437739 | 5.637 | 41386 |
| Total | | 7764887 | 100.000 | 692673 |

## **Supplementary Figure 70.** $^{1}$H, $^{13}$C and HPLC spectra of **9**

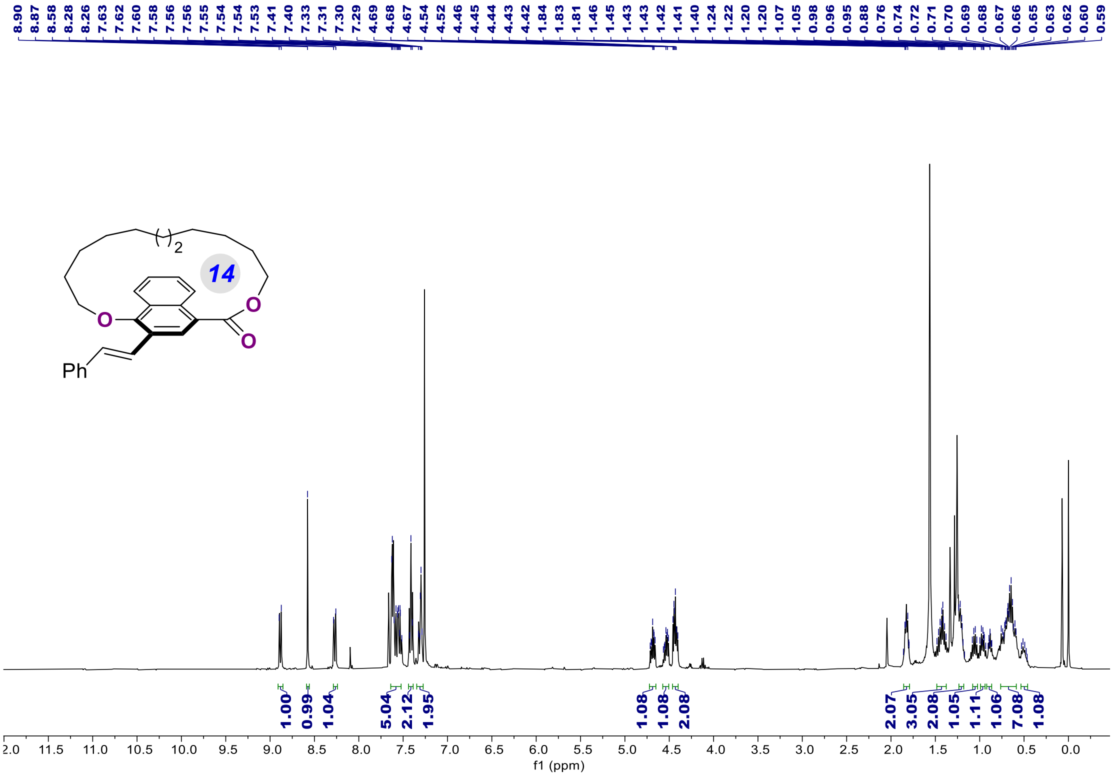


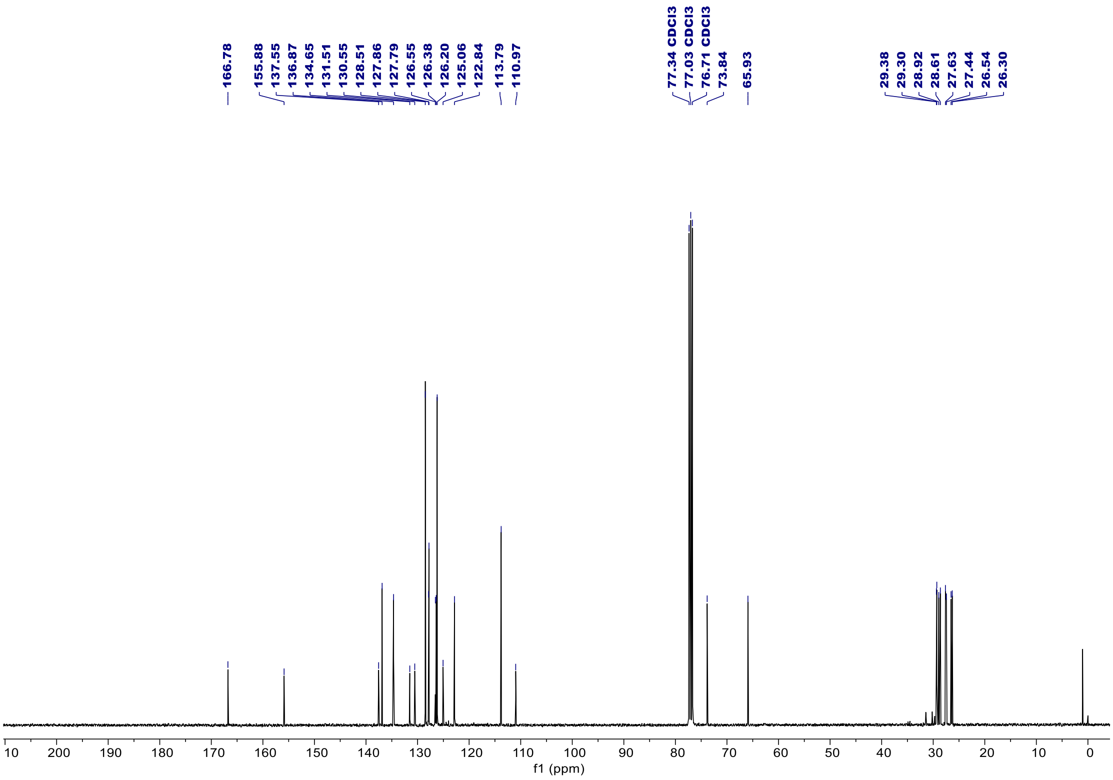

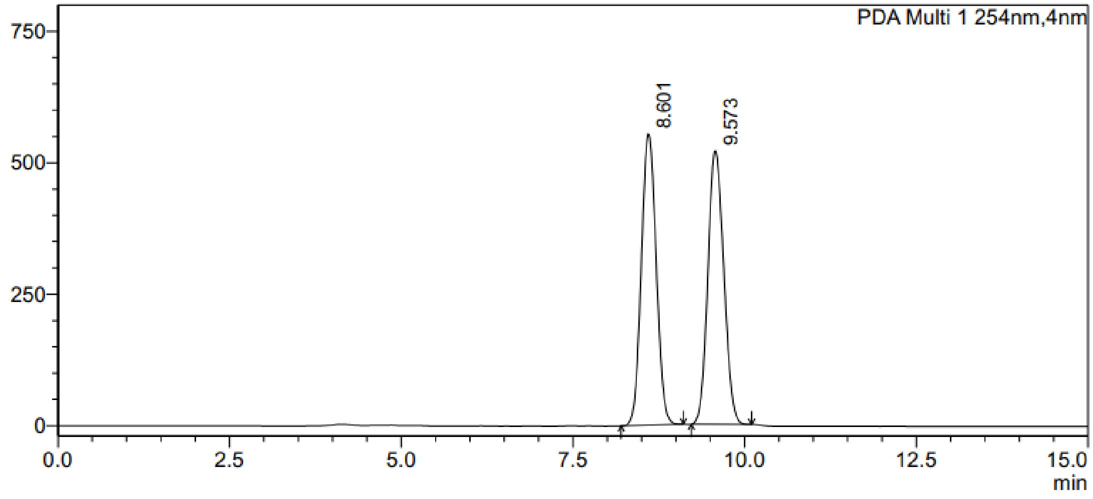


PDA Ch1 254nm

| Peak# | Ret. Time | Area | Area% | Height |
|---|---|---|---|---|
| 1 | 8.601 | 8360196 | 49.858 | 553741 |
| 2 | 9.573 | 8407831 | 50.142 | 519799 |
| Total | | 16768027 | 100.000 | 1073540 |

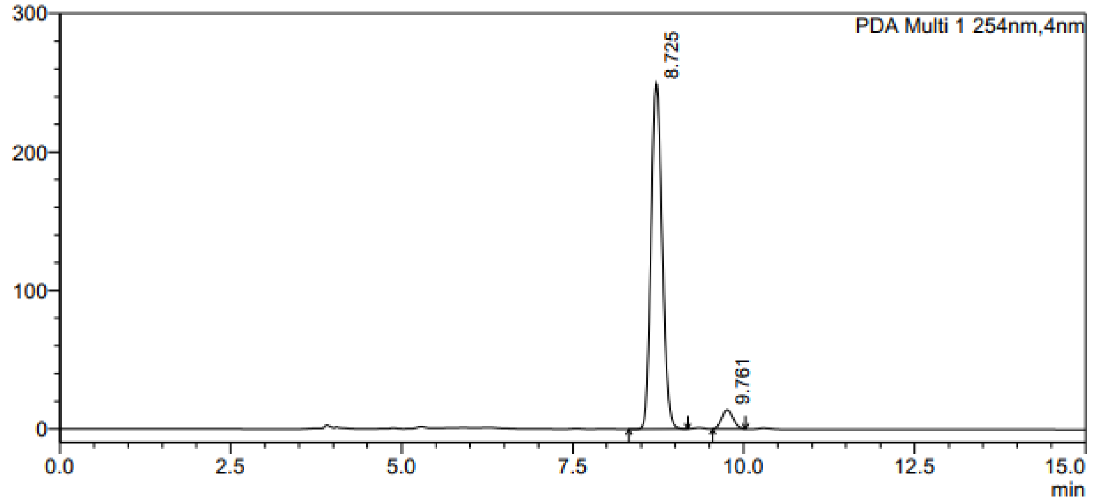


PDA Ch1 254nm

| Peak# | Ret. Time | Area | Area% | Height |
|---|---|---|---|---|
| 1 | 8.725 | 2799884 | 94.591 | 249232 |
| 2 | 9.761 | 160111 | 5.409 | 13637 |
| Total | | 2959996 | 100.000 | 262869 |

**Supplementary Figure 71.** $^{1}$H, $^{13}$C and HPLC spectra of **11**

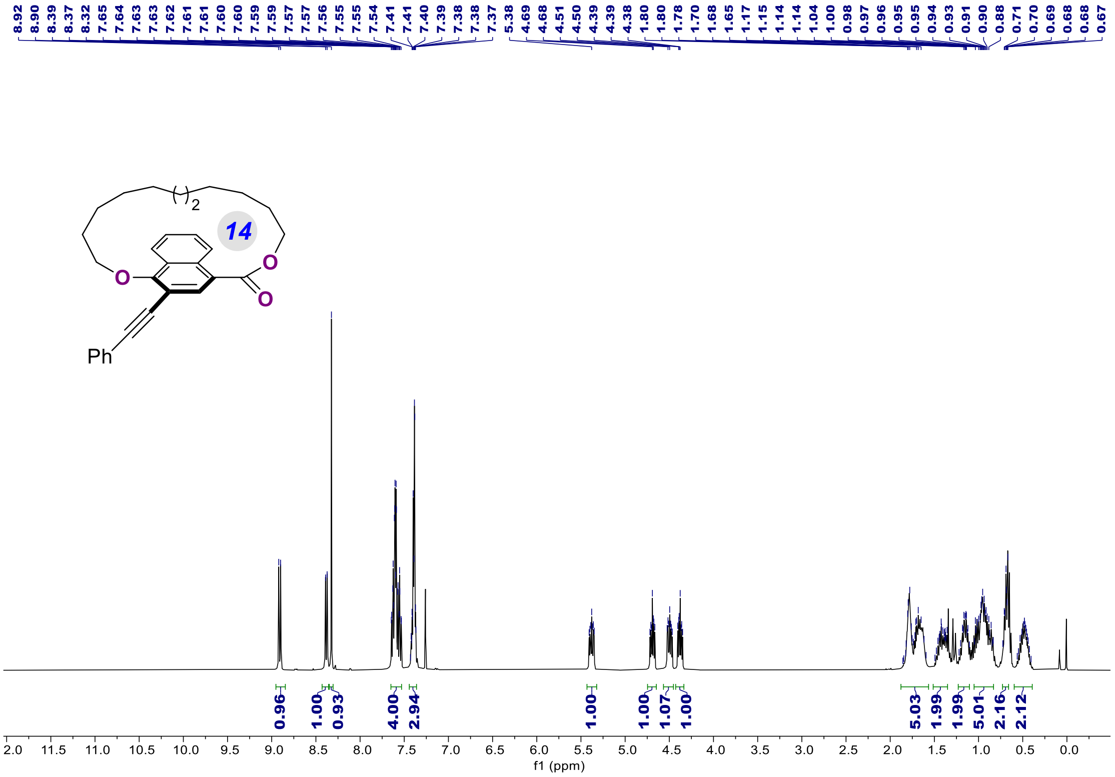


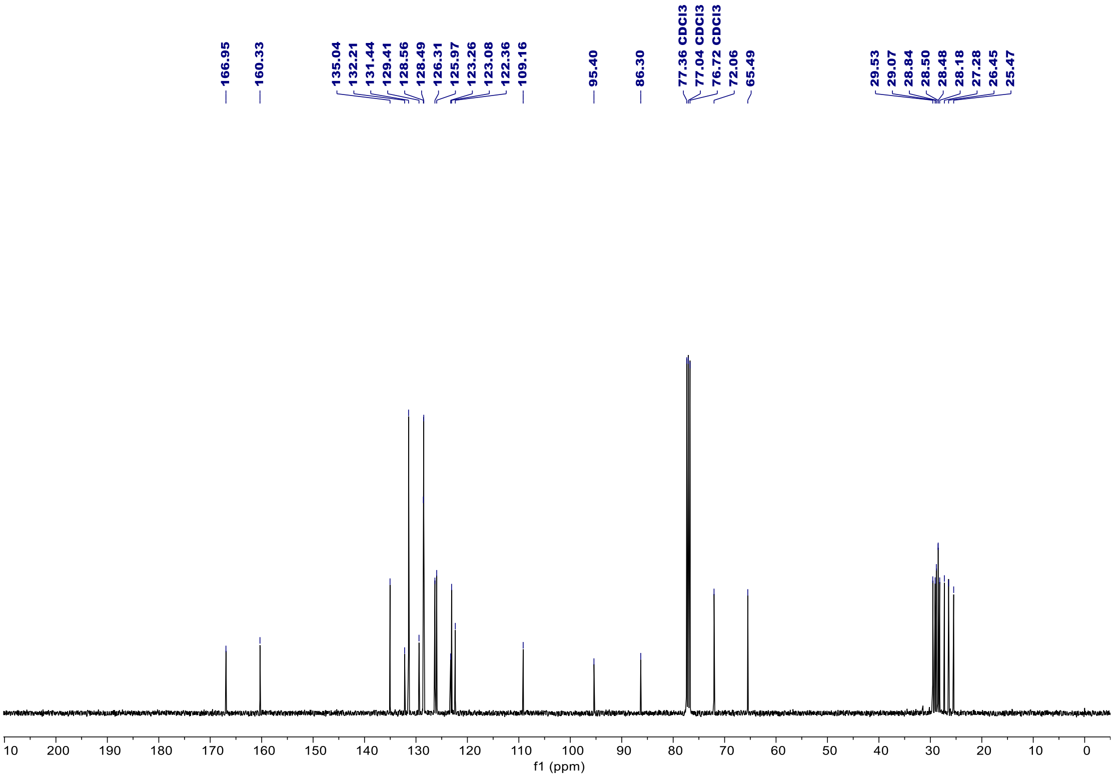

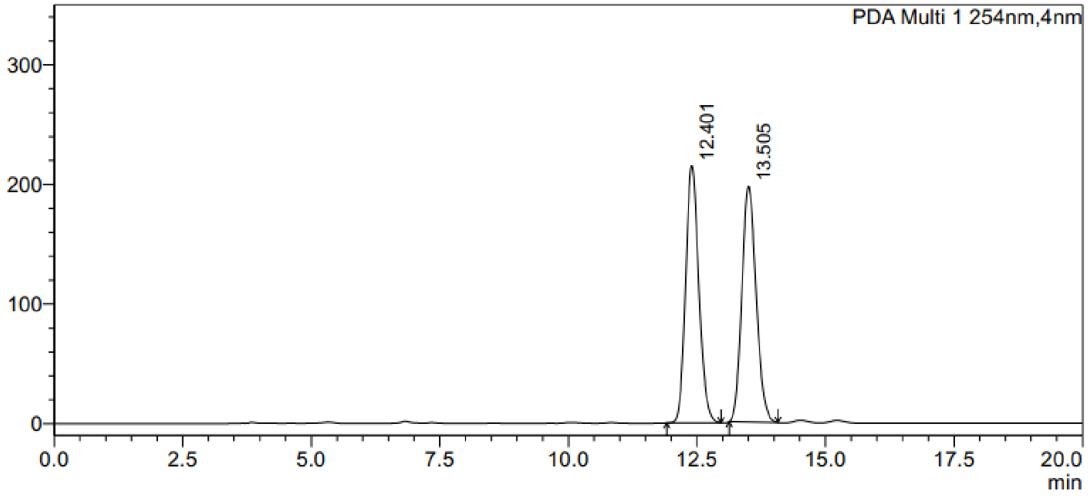


PDA Ch1 254nm

| Peak# | Ret. Time | Area | Area% | Height |
|---|---|---|---|---|
| 1 | 12.401 | 3851668 | 50.204 | 214832 |
| 2 | 13.505 | 3820376 | 49.796 | 196800 |
| Total | | 7672043 | 100.000 | 411633 |

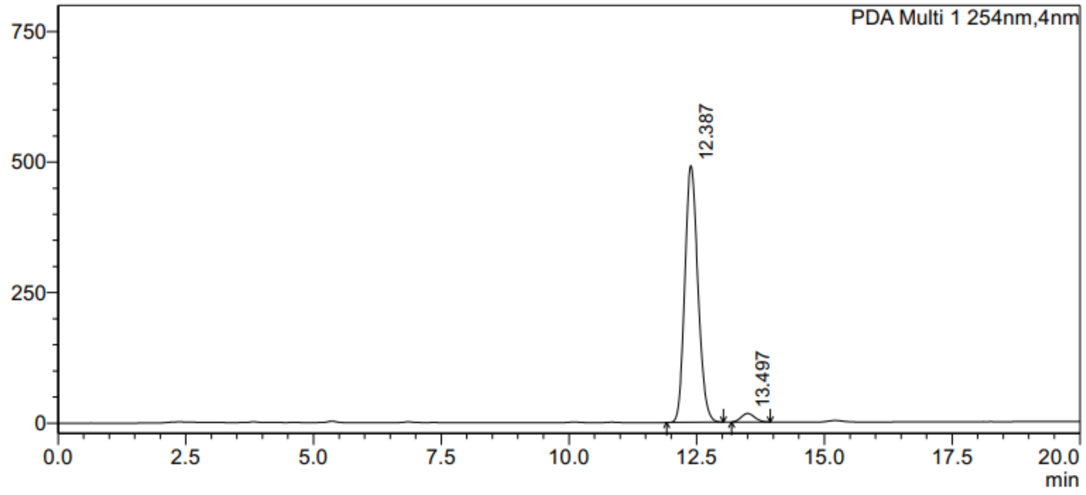


PDA Ch1 254nm

| Peak# | Ret. Time | Area | Area% | Height |
|---|---|---|---|---|
| 1 | 12.387 | 8774488 | 96.616 | 491763 |
| 2 | 13.497 | 307337 | 3.384 | 16533 |
| Total | | 9081825 | 100.000 | 508297 |

# IV. Supplementary References


1. Chen, X., Yan, L., Liu, Y., Yang, Y. & You, J. Switchable cascade C-H annulation to polycyclic pyryliums and pyridiniums: discovering mitochondria-targeting fluorescent probes. *Chem. Commun.* **56**, 15080-15083 (2020).
2. Velder, J. et al. Modular synthesis of chiral phosphine-phosphite-pigands from phenolic precursors: a new approach to bidentate chelateligands exploiting a P-O to P-C migration rearrangement. *Adv. Syn. Cata.* **350**, 1309-1315 (2008).
3. Epifano, F. et al. Topical anti-inflammatory activity of boropinic acid and its natural and semi-synthetic derivatives. *Bio.Med. Chem. Lett.* **21**, 769-772 (2011).
4. Sdahl, M., Conrad, J., Braunberger, C. & Beifuss, U. Efficient and sustainable laccase-catalyzed iodination of p-substituted phenols using KI as iodine source and aerial O(2) as oxidant. *RSC Adv.* **9**, 19549-19559 (2019).
5. Ren, T., Zhang, G. & Liu, D. Synthesis of bifunctional cationic compound for gene delivery. *Tetrahedron Lett.* **42**, 1007-1010 (2001).
6. Yadav, J. S., Ganganna, B., Dutta, P. & Singarapu, K. K. Synthesis and determination of absolute configuration of alpha-pyrones isolated from Penicillium corylophilum. *J. Org. Chem.* **79**, 10762-10771 (2014).
7. Yadav, J. S., Suresh, B. & Srihari, P. Expedient synthesis of large-ringtrans-enamide macrolides by CuI-mediated intramolecular coupling of vinyl iodide with amide: total synthesis of palmyrolide A. *Eur. J. Org. Chem.* **2016**, 2509-2513 (2016).
8. Gundoju, N. R. et al. Total syntheses of surinone B, alatanones A-B, and trineurone A. *J. Asian. Nat. Prod. Res.* **21**, 262-269 (2019).
9. Tonoi, T. et al. 4-(Dimethylamino)pyridine N-Oxide-catalyzed macrolactamization using 2-Methyl-6-nitrobenzoic anhydride in the synthesis of the depsipeptidic analogue of FE399. *ACS Omega* **6**, 3571-3577 (2021).
10. Aida, K. et al. Catalytic reductive ring opening of epoxides enabled by zirconocene and photoredox catalysis. *Chem* **8**, 1762-1774 (2022).
11. Sargent, B. T. & Alexanian, E. J. Cobalt-catalyzed aminocarbonylation of alkyl tosylates: stereospecific synthesis of amides. *Angew. Chem. Int. Ed.* **58**, 9533-9536 (2019).

12. Trost, B. M., Harrington, P. E., Chisholm, J. D. & Wrobleski, S. T. Total synthesis of (+)-amphidinolide A. structure elucidation and completion of the synthesis. *J. Am. Chem. Soc.* **127**, 13598-13610 (2005).
13. Garg, N. K., Hiebert, S. & Overman, L. E. Total synthesis of (-)-sarain A. *Angew. Chem. Int. Ed.* **45**, 2912-2915 (2006).
14. Menche, D., Hassfeld, J. & Li, J. Rudolph, S.,Total synthesis of archazolid A. *J. Am. Chem. Soc.* **129**, 6100-6101 (2007).
15. Bihelovic, F. & Saicic, R. N. Total synthesis of (-)-atrop-abyssomicin C. *Angew. Chem. Int. Ed.* **51**, 5687-5691 (2012).
16. Tripathi, C. B., Kayal, S. & Mukherjee, S. Catalytic asymmetric synthesis of α,β-disubstituted α,γ-diaminophosphonic acid precursors by Michael addition of α-substituted nitrophosphonates to nitroolefins. *Org. Lett.* **14**, 3296-3299 (2012).
17. Yu, F., Hu, H., Gu, X. & Ye, J. Asymmetric Michael addition of substituted rhodanines to α,β-unsaturated ketones catalyzed by bulky primary amines. *Org. Lett.* **14**, 2038-2041 (2012).
18. Gagnon, C. et al. Biocatalytic synthesis of planar chiral macrocycles. *Science* **367**, 917–921 (2020).
